# Supplementary material for: How Robust Are Genomic Offset Predictions to Methodological Choices? Insights From Perennial Ryegrass
Source: Mol Ecol. 2026 Jul 13;35(14):e70463. doi: 10.1111/mec.70463 (PMC13361167; doi:10.1111/mec.70463)
Supplement: Supplementary file 1 — Appendix S1: Supplementary Material Description. [file MEC-35-e70463-s010.docx]

Supplementary Material Description

The supplementary material comprises eight tables and three figures. Supplementary Tables S1–S4 describe the plant accessions, the concordance of SNP positions between genome assemblies, and the environmental and phenotypic data used in the analyses. Supplementary Tables S5–S8 report the outlier loci detected by Gradient Forest and CANCOR, their genomic annotation, and their associations with phenotypic traits. Supplementary Figures S1–S3 illustrate the relationships between phenotypic traits and experienced genomic offset under both models, together with the predicted spatial variation in adaptive genomic composition.
