# Supplementary material for: How Robust Are Genomic Offset Predictions to Methodological Choices? Insights From Perennial Ryegrass
Source: Mol Ecol. 2026 Jul 13;35(14):e70463. doi: 10.1111/mec.70463 (PMC13361167; doi:10.1111/mec.70463)
Supplement: Supplementary file 2 — Figure S1: Relationship between phenotypic trait values measured in the common gardens and the genomic offset experienced at each site during the growth period preceding trait measurement, based on the GFGF model. Each panel corresponds to a single phenotypic trait, with the trait label (e.g., ADF_04_me17) indicated on the y‐axis; the trait name encodes the trait, the measurement period, and the common garden and year of measurement. Each point represents one population, coloured according to the adaptive genetic composition of its location of origin (as in Figure 3, derived from the first three principal components of the GF‐transformed environmental variables mapped onto an RGB colour palette). The red line shows the linear regression of the trait on genomic offset, and the grey band its 95% confidence interval. [file MEC-35-e70463-s001.pdf]

Figure S1 : Relationship between phenotypic trait values measured in the common gardens and the genomic offset experienced at each site during the growth period preceding trait measurement, based on the GF_{GF} model. Each panel corresponds to a single phenotypic trait, with the trait label (e.g., ADF_04_me17) indicated on the y-axis; the trait name encodes the trait, the measurement period, and the common garden and year of measurement. Each point represents one population, coloured according to the adaptive genetic composition of its location of origin (as in Figure 3, derived from the first three principal components of the GF-transformed environmental variables mapped onto an RGB colour palette). The red line shows the linear regression of the trait on genomic offset, and the grey band its 95% confidence interval.

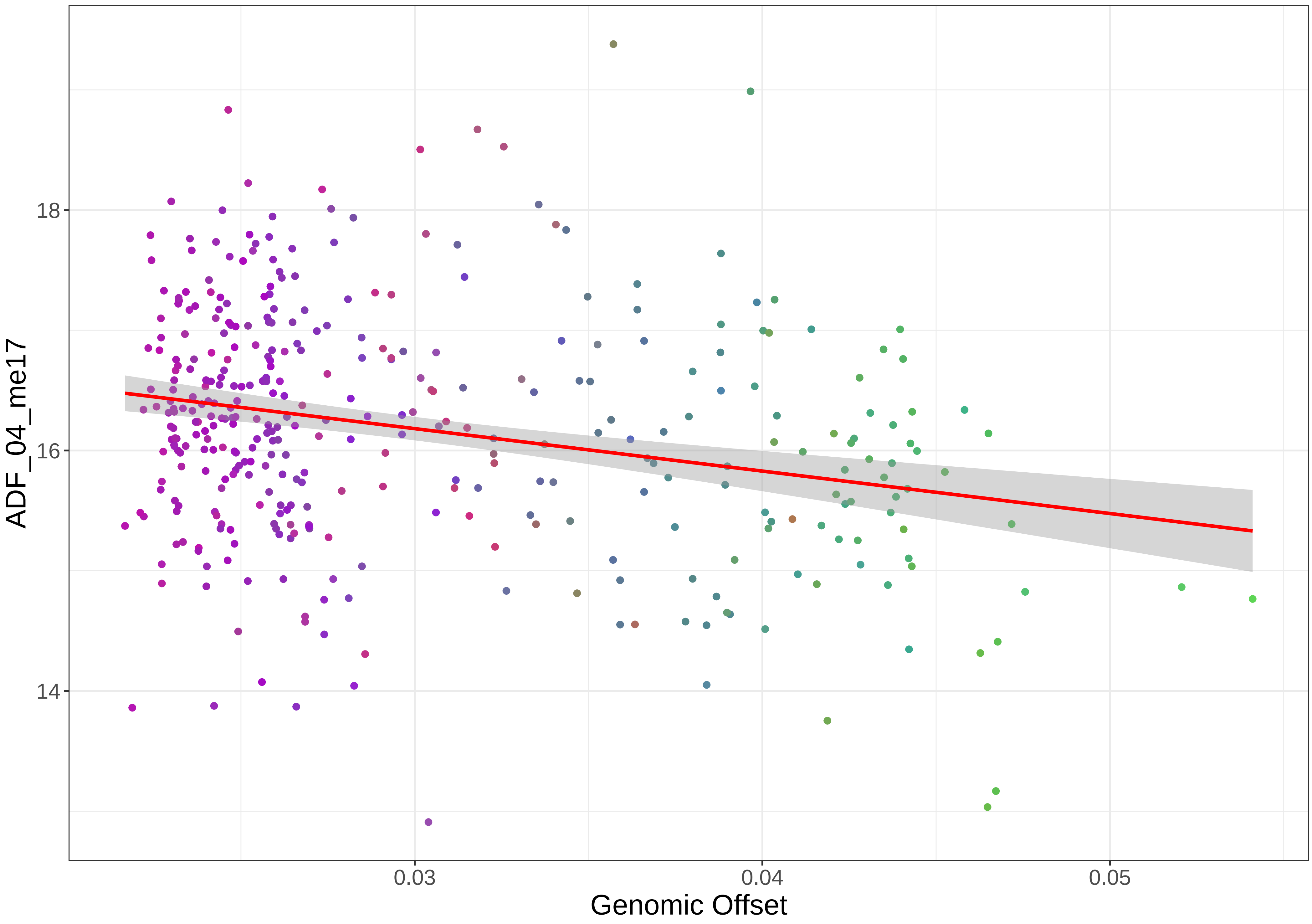

ADF_04_me17
18
16
14
0.03
0.04
0.05
Genomic Offset

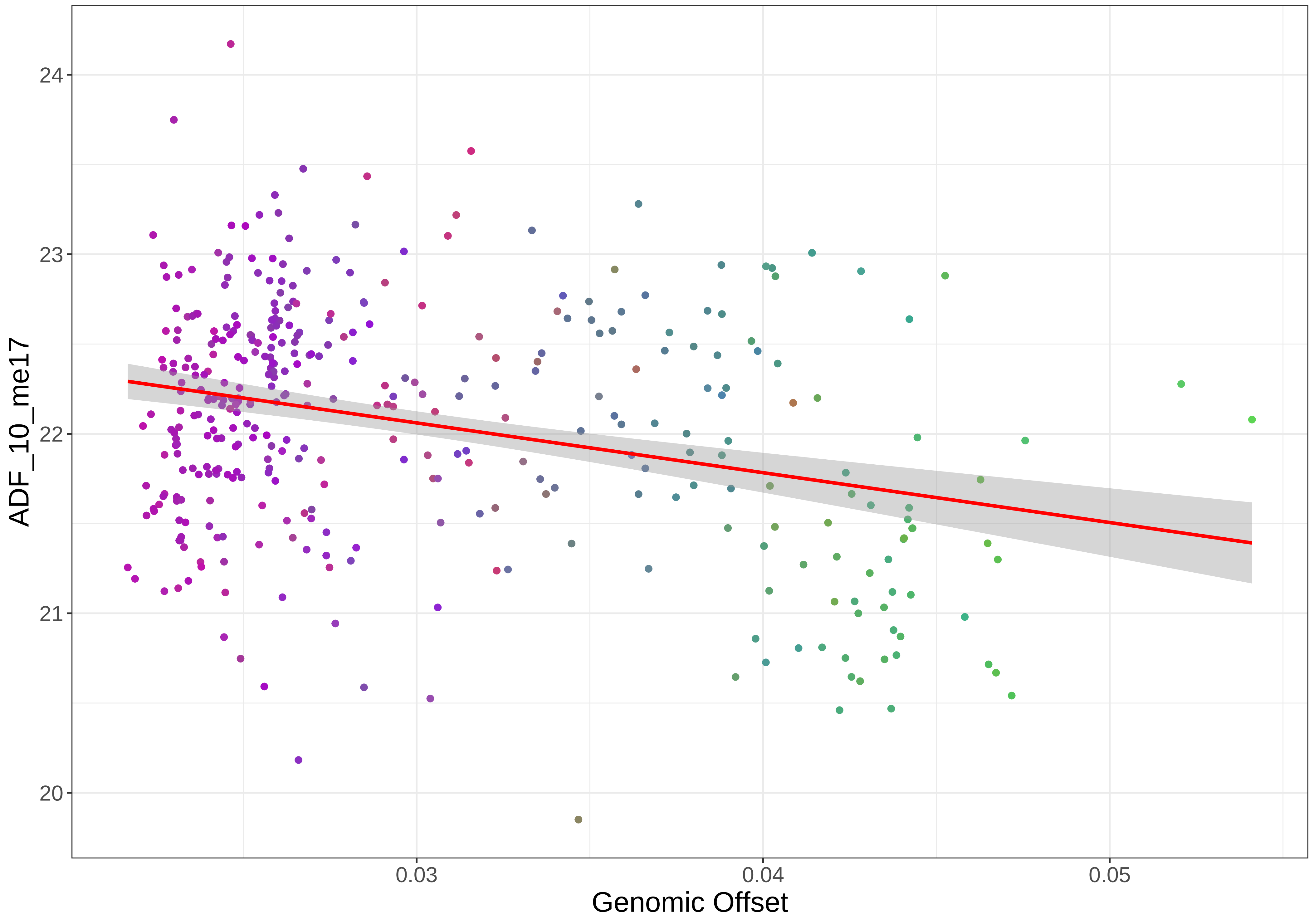

24
23
22
21
20
ADF_10_me17
0.03
0.04
0.05
Genomic Offset

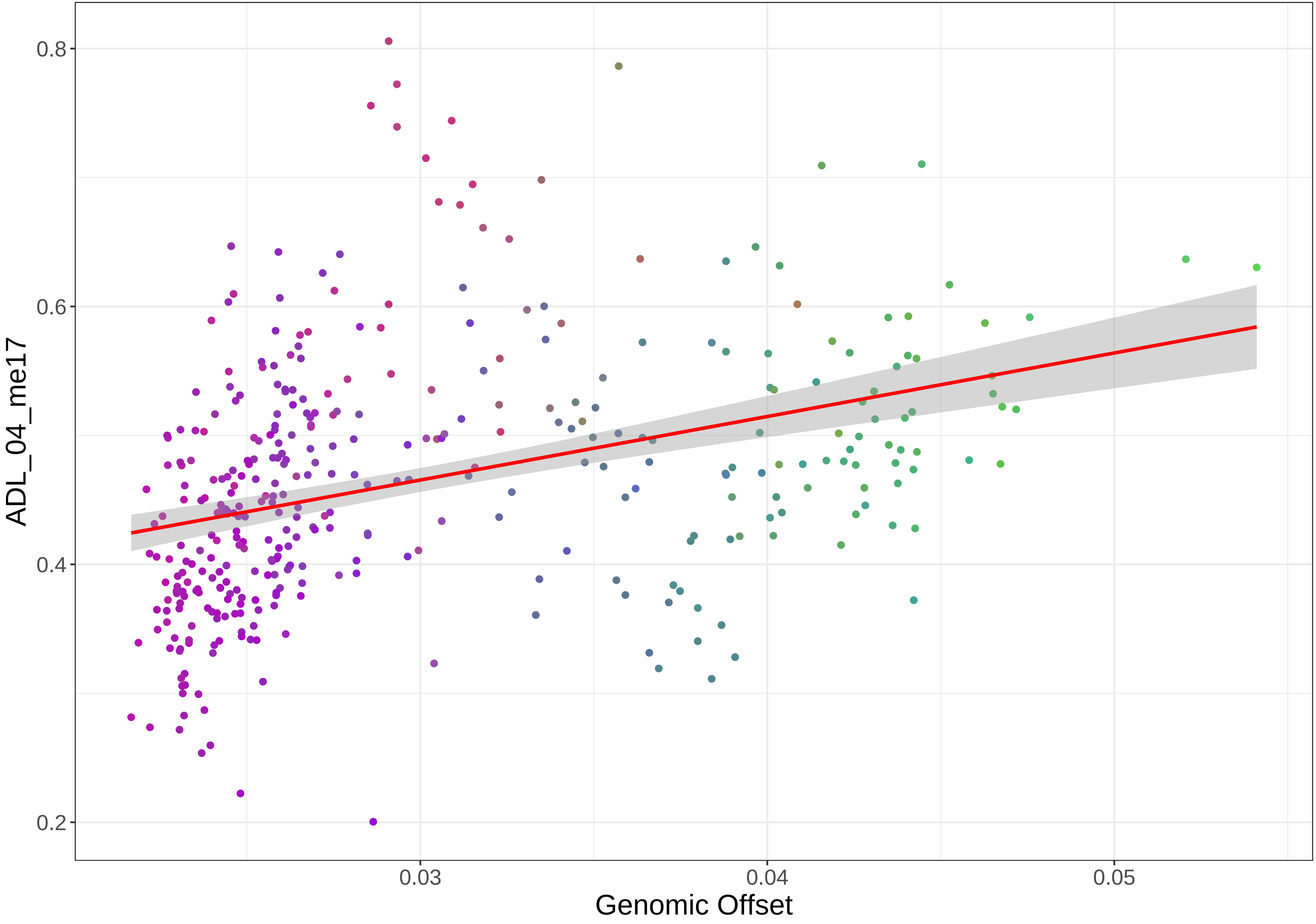
0.8
0.6
0.4
0.2
ADL_04_me17
0.03
0.04
0.05
Genomic Offset

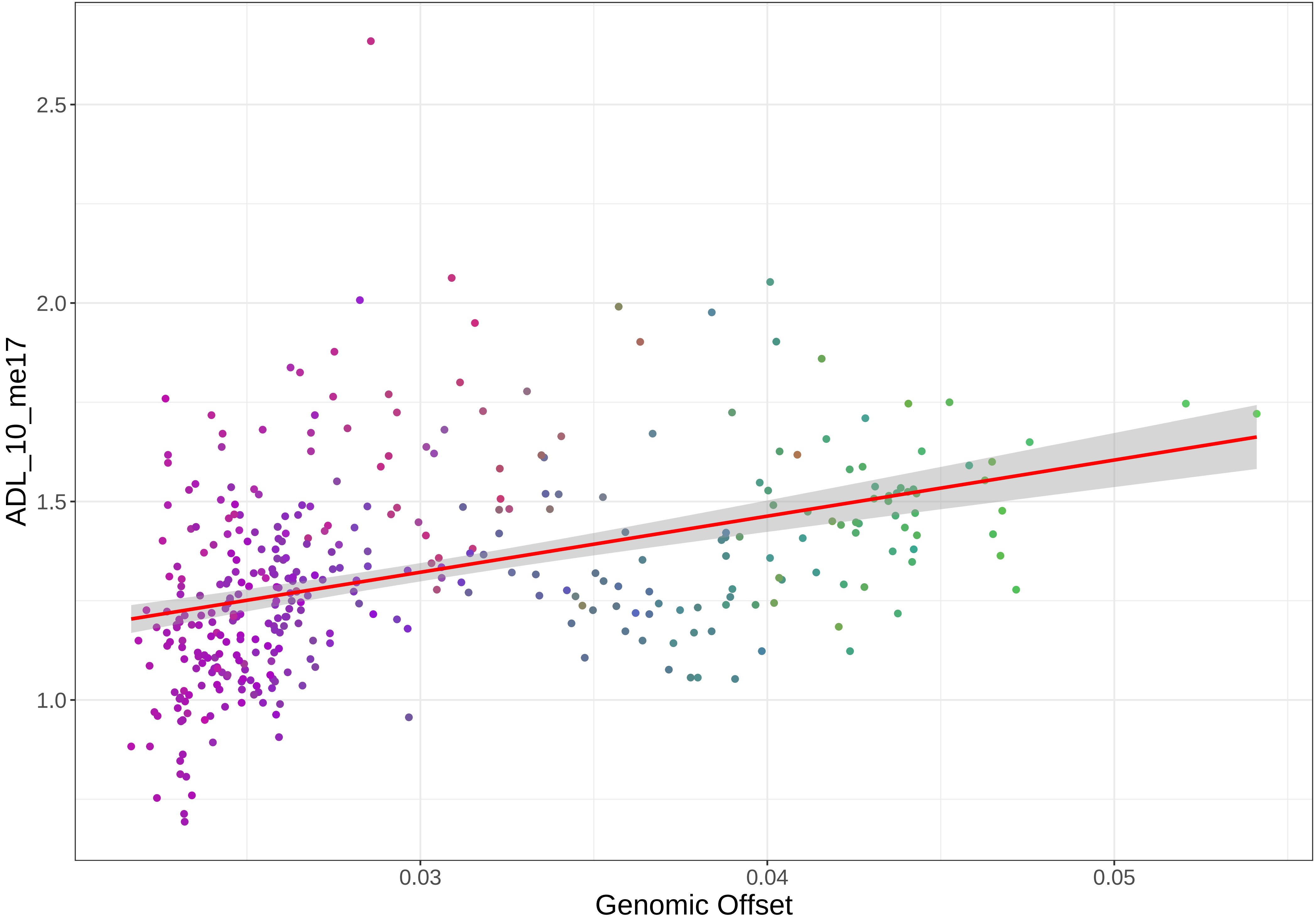

ADL_10_me17
2.5
2.0
1.5
1.0
0.03
0.04
0.05
Genomic Offset

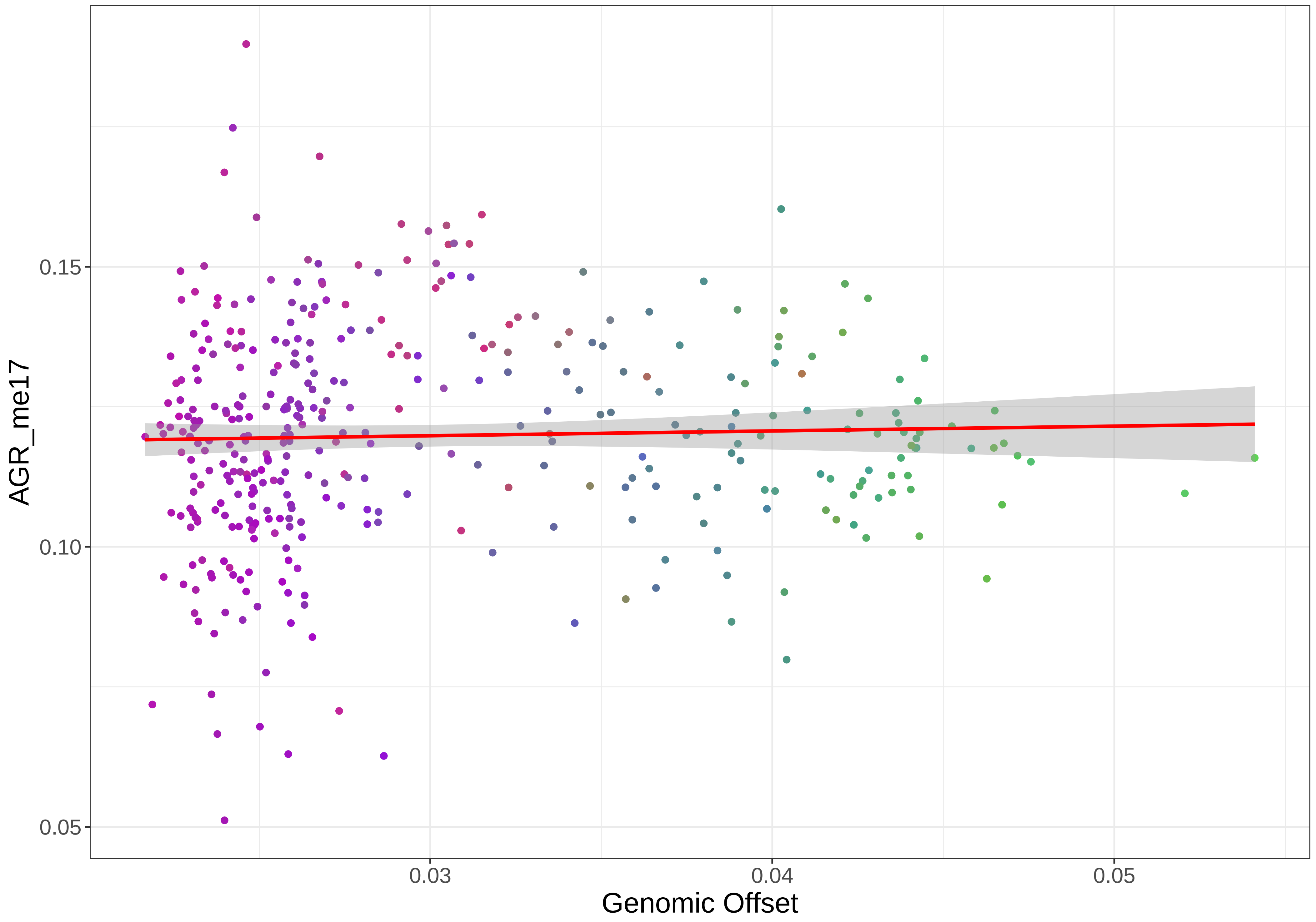

AGR_me17
0.15
0.10
0.05
0.03
0.04
0.05
Genomic Offset

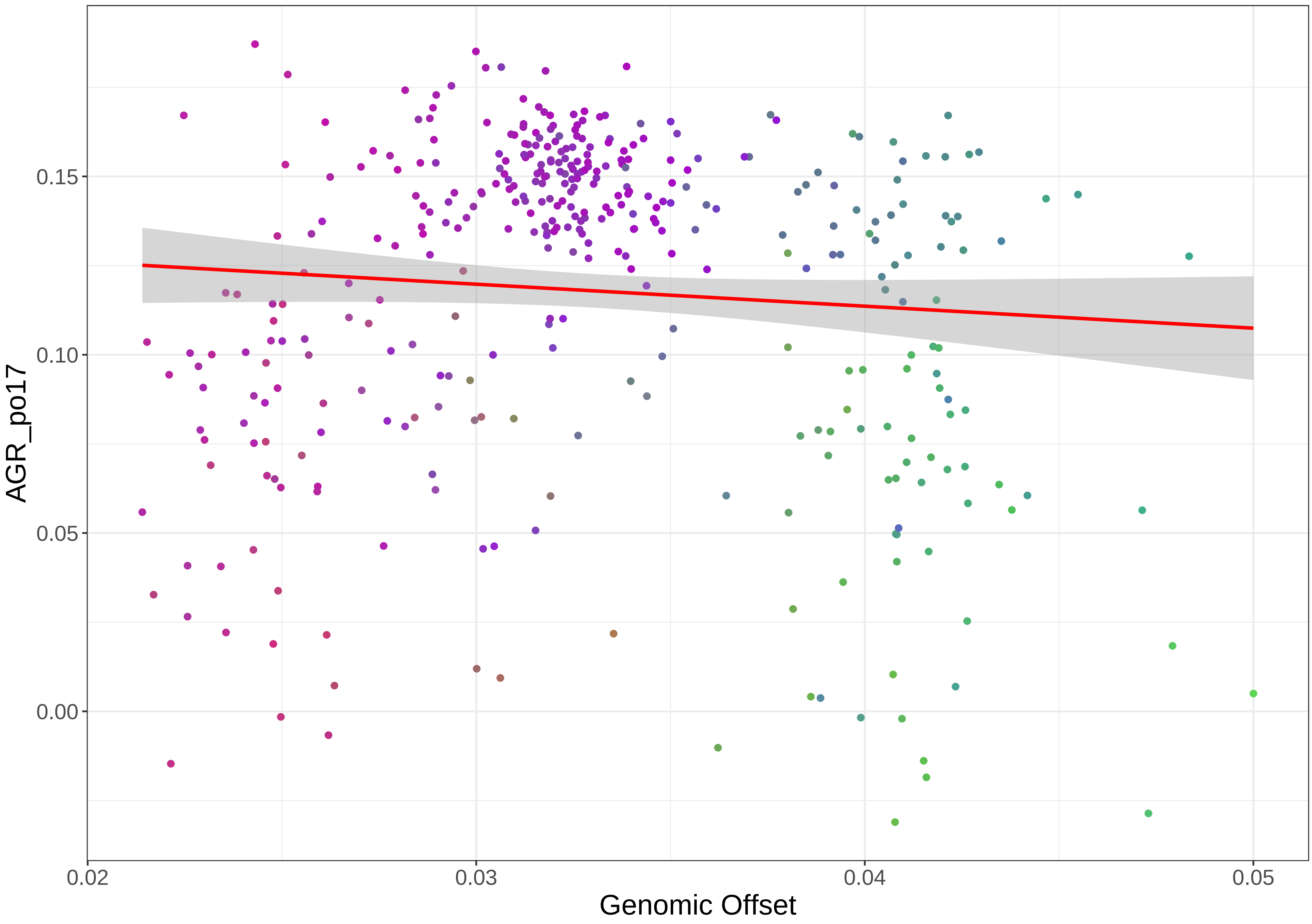
AGR_po17
0.15
0.10
0.05
0.00
0.02
0.03
0.04
0.05
Genomic Offset

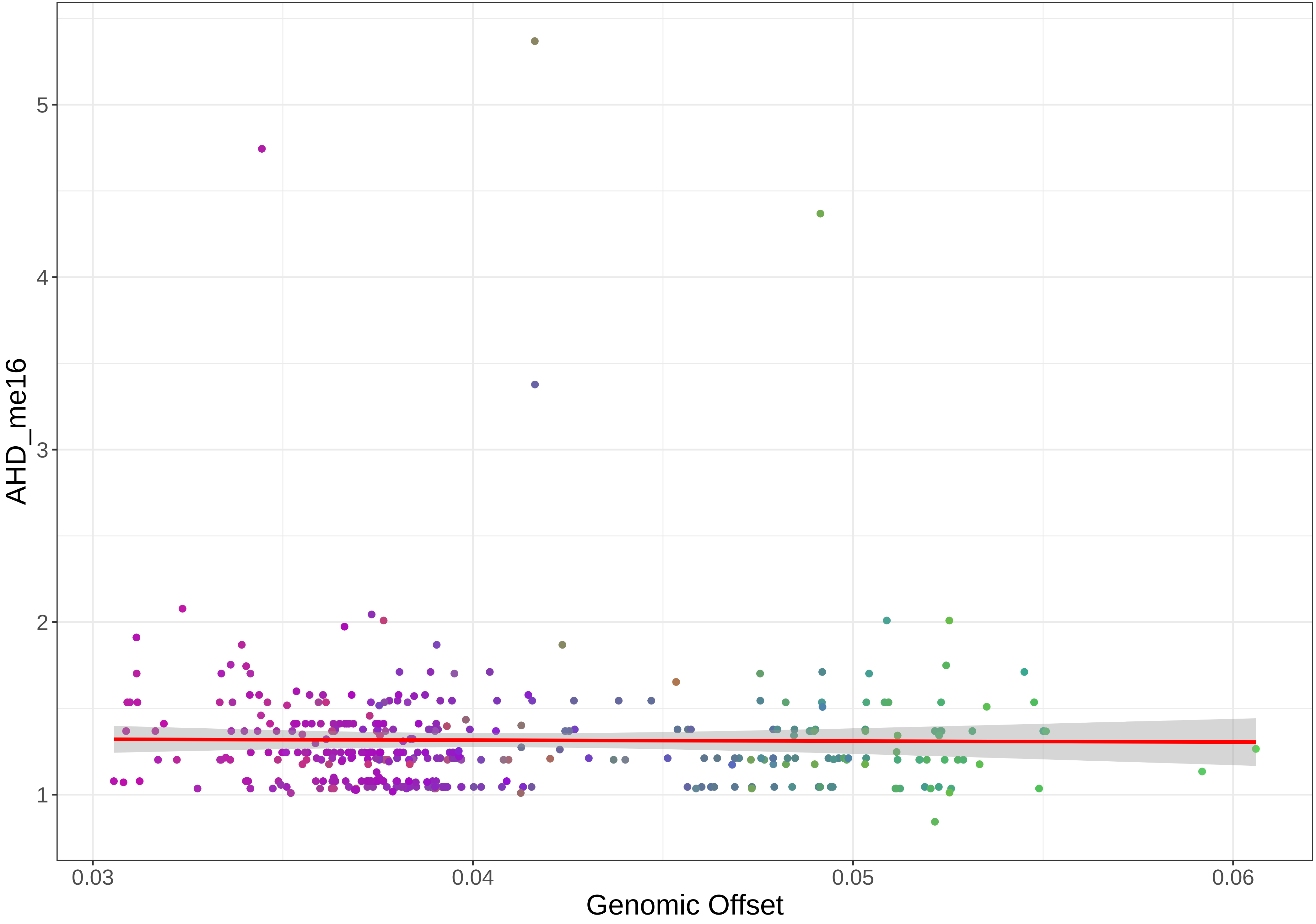

AHD_me16
5
4
3
2
1
0.03
0.04
0.05
0.06
Genomic Offset

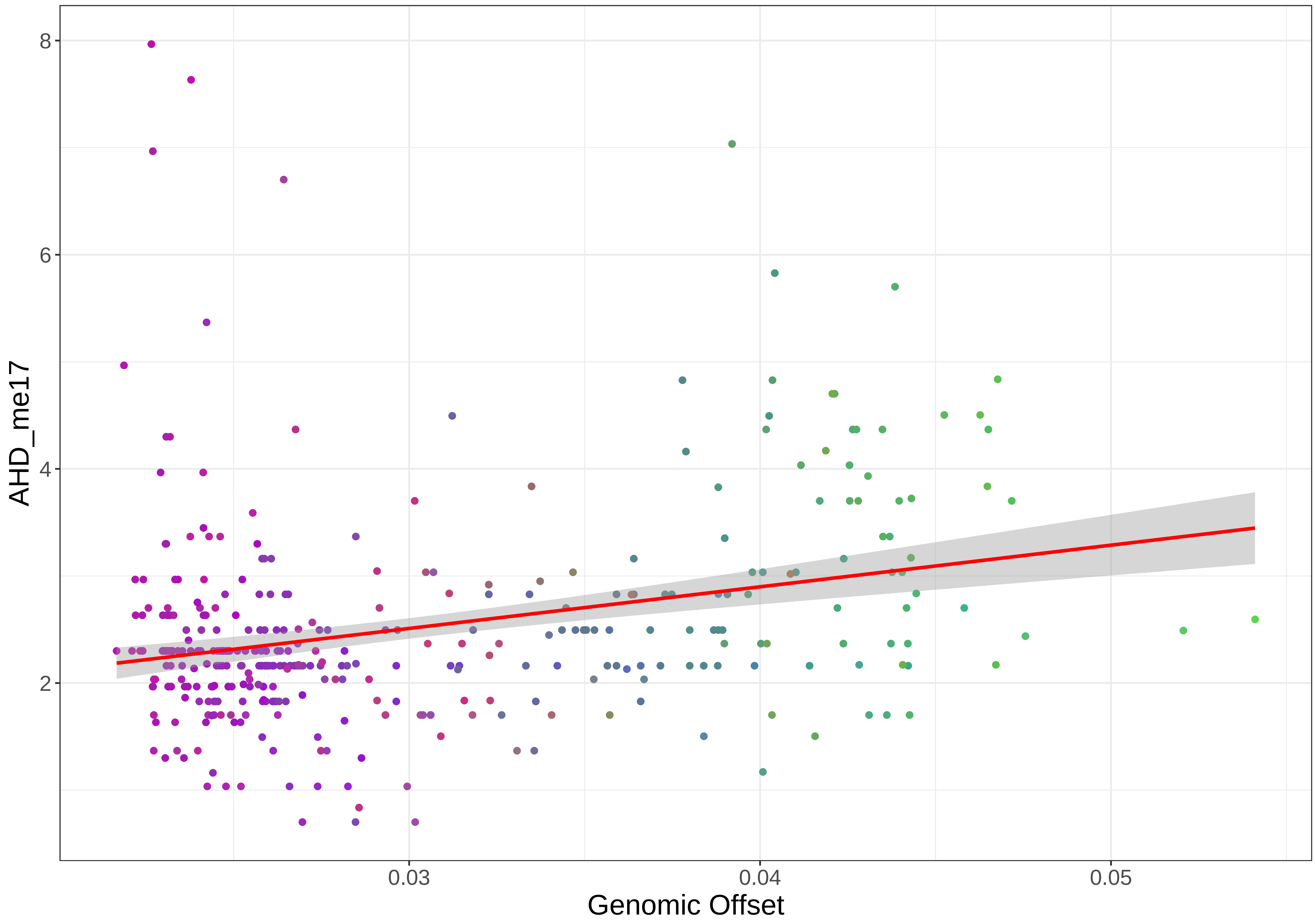
AHD_me17
8
6
4
2
0.03
0.04
0.05
Genomic Offset

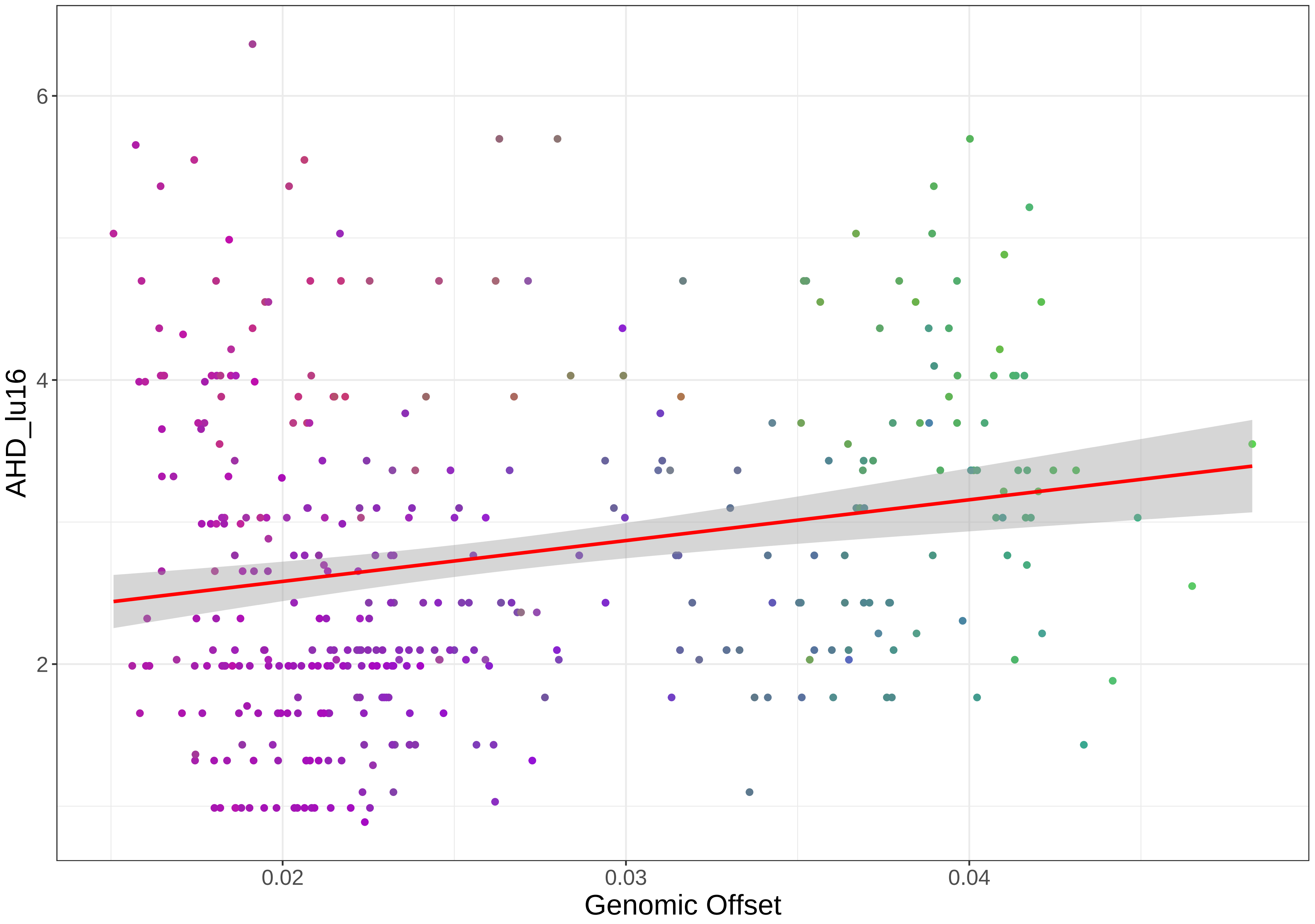
6
4
2
AHD_lu16
0.02
0.03
0.04
Genomic Offset

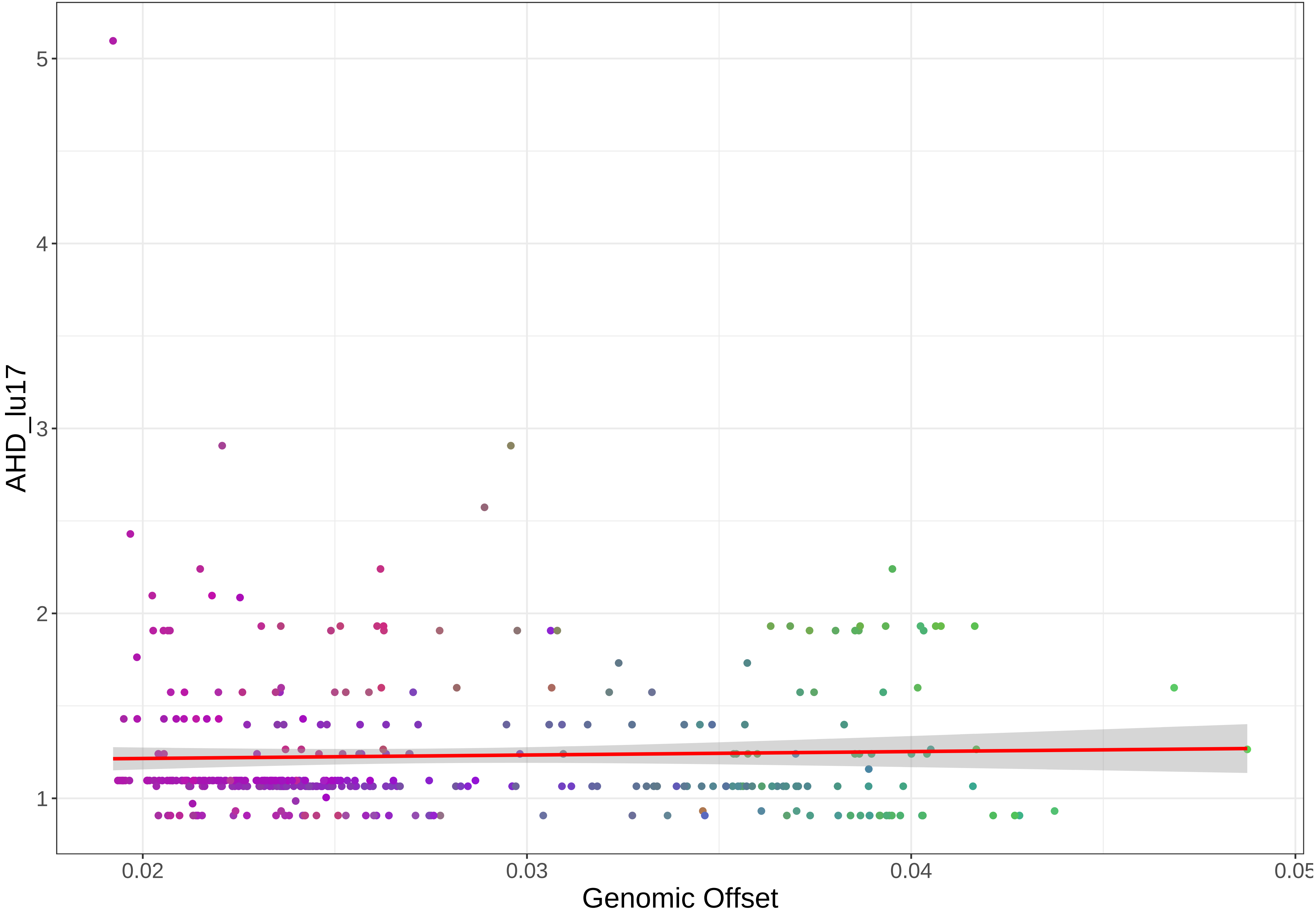

AHD_lu17
1
2
3
4
5
0.02
0.03
0.04
0.05
Genomic Offset

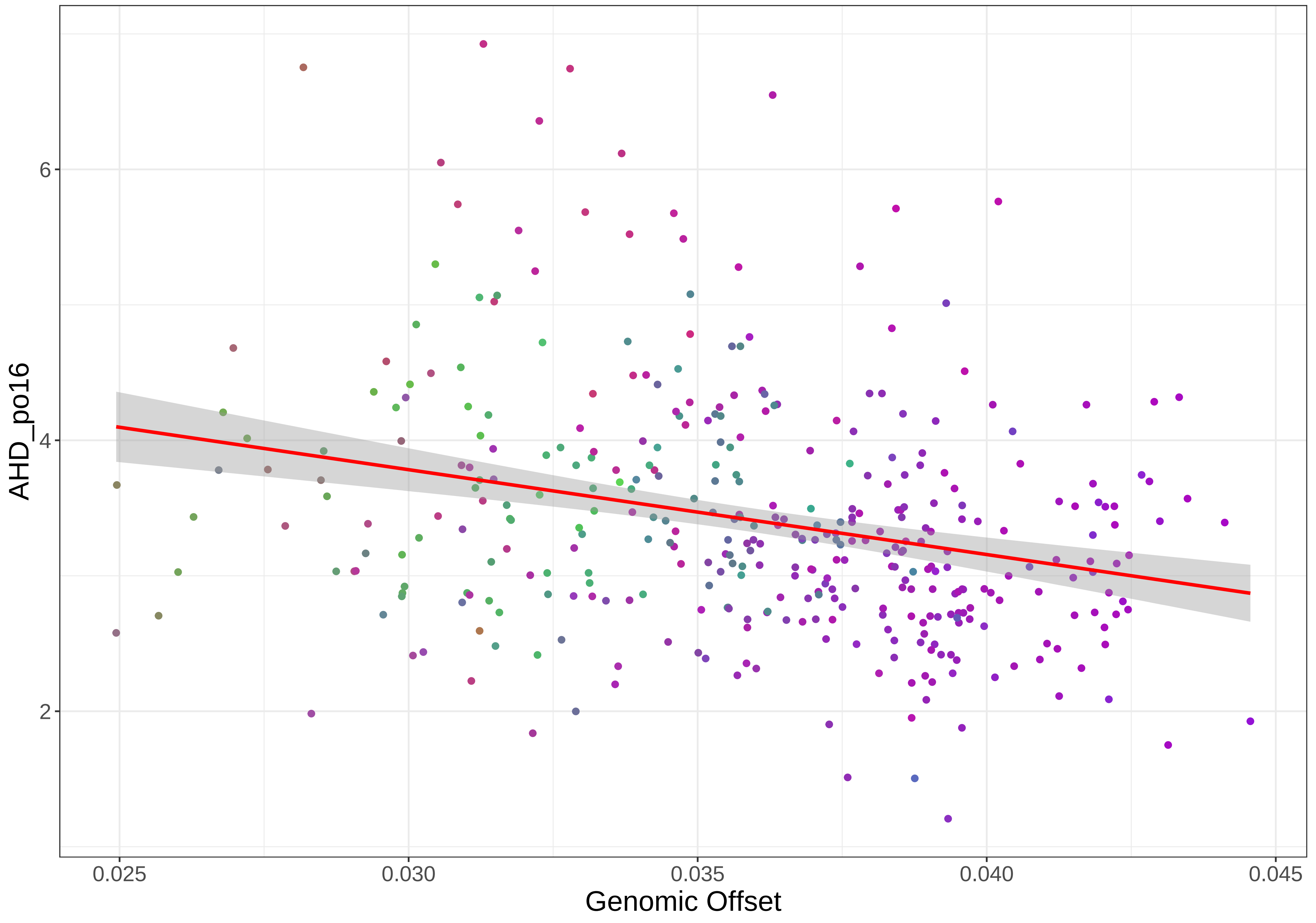

AHD_po16
6
4
2
0.025
0.030
0.035
0.040
0.045
Genomic Offset

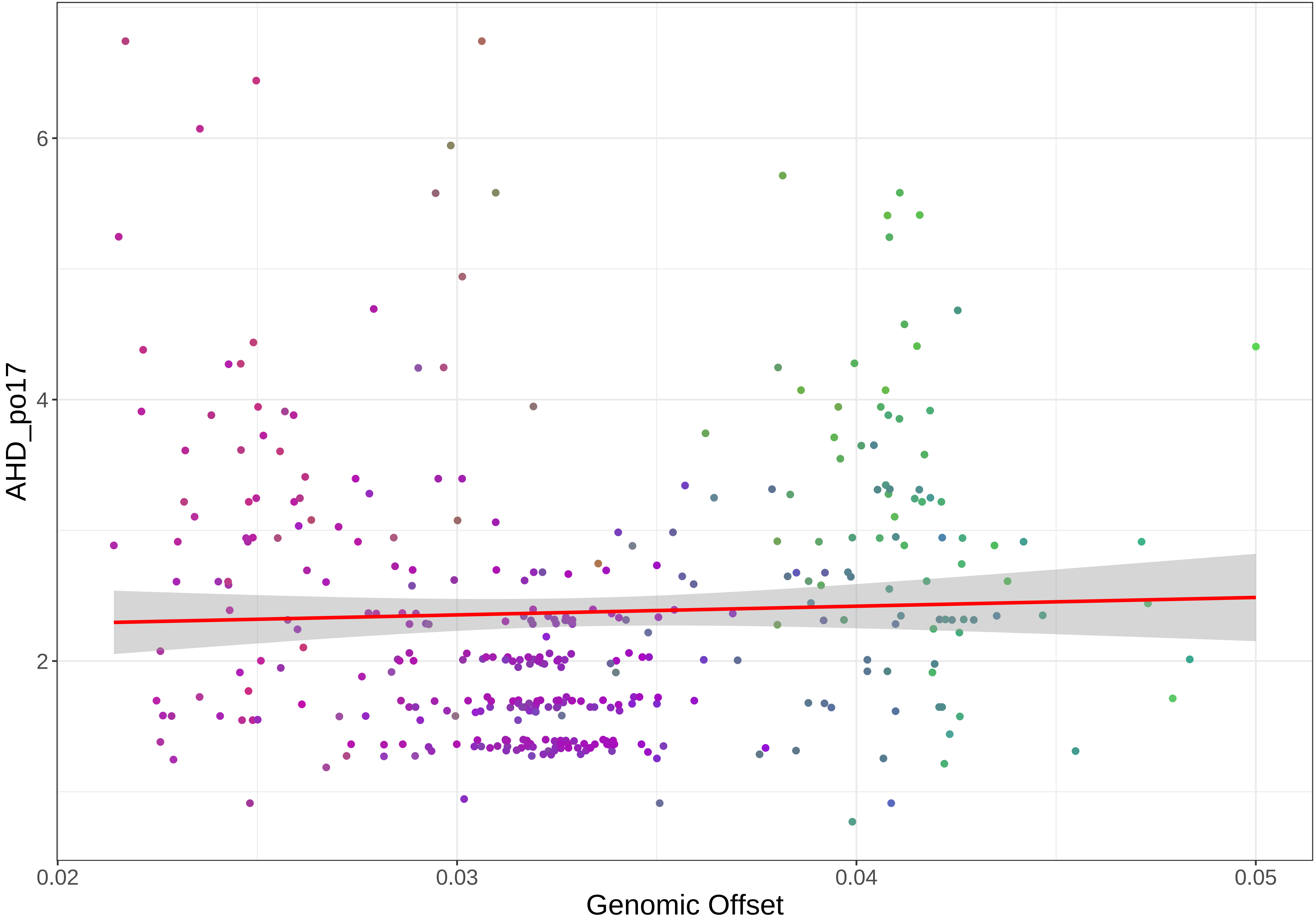

AHD_po17
6
4
2
0.02
0.03
0.04
0.05
Genomic Offset

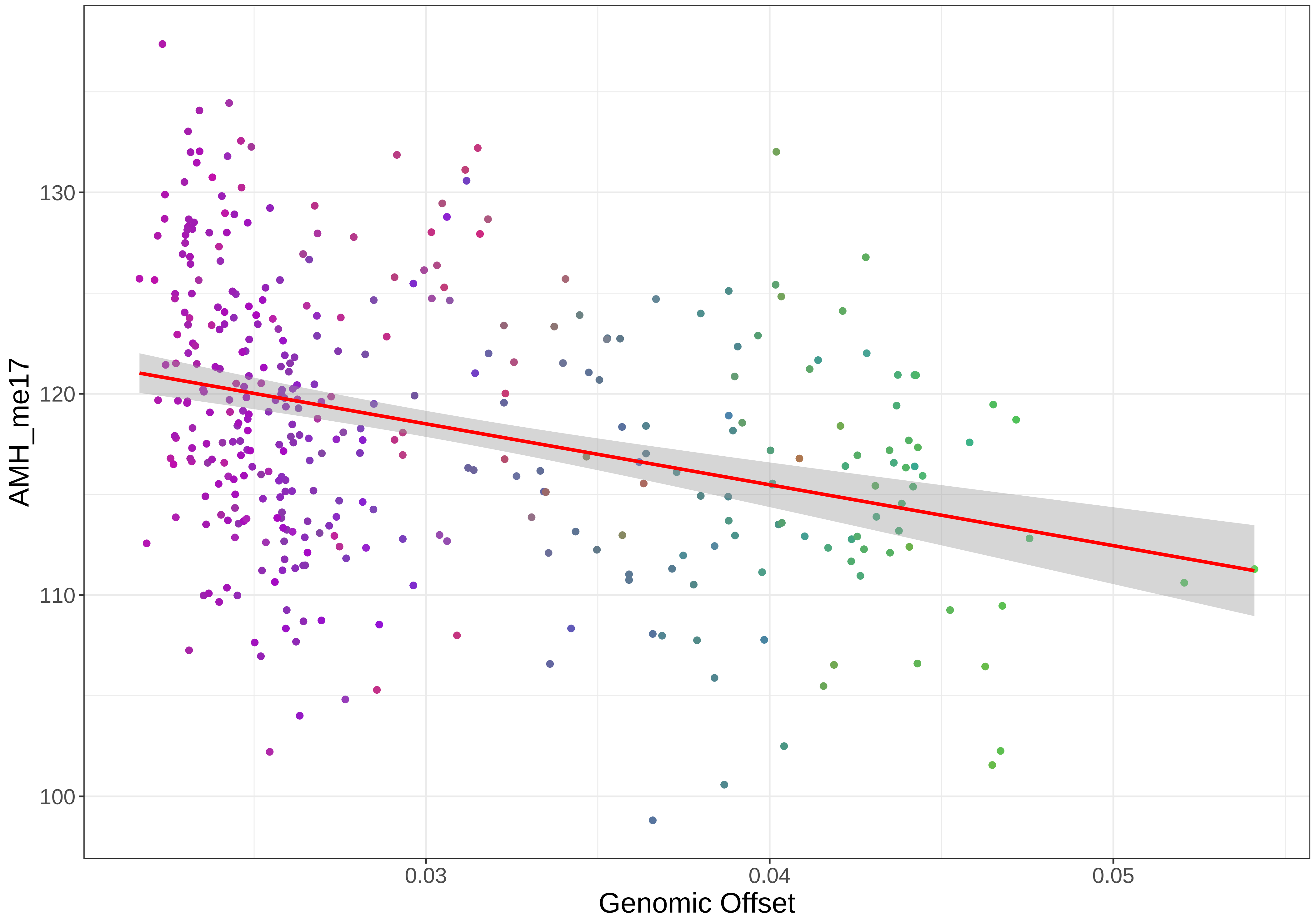
AMH_me17
130
120
110
100
0.03
0.04
0.05
Genomic Offset

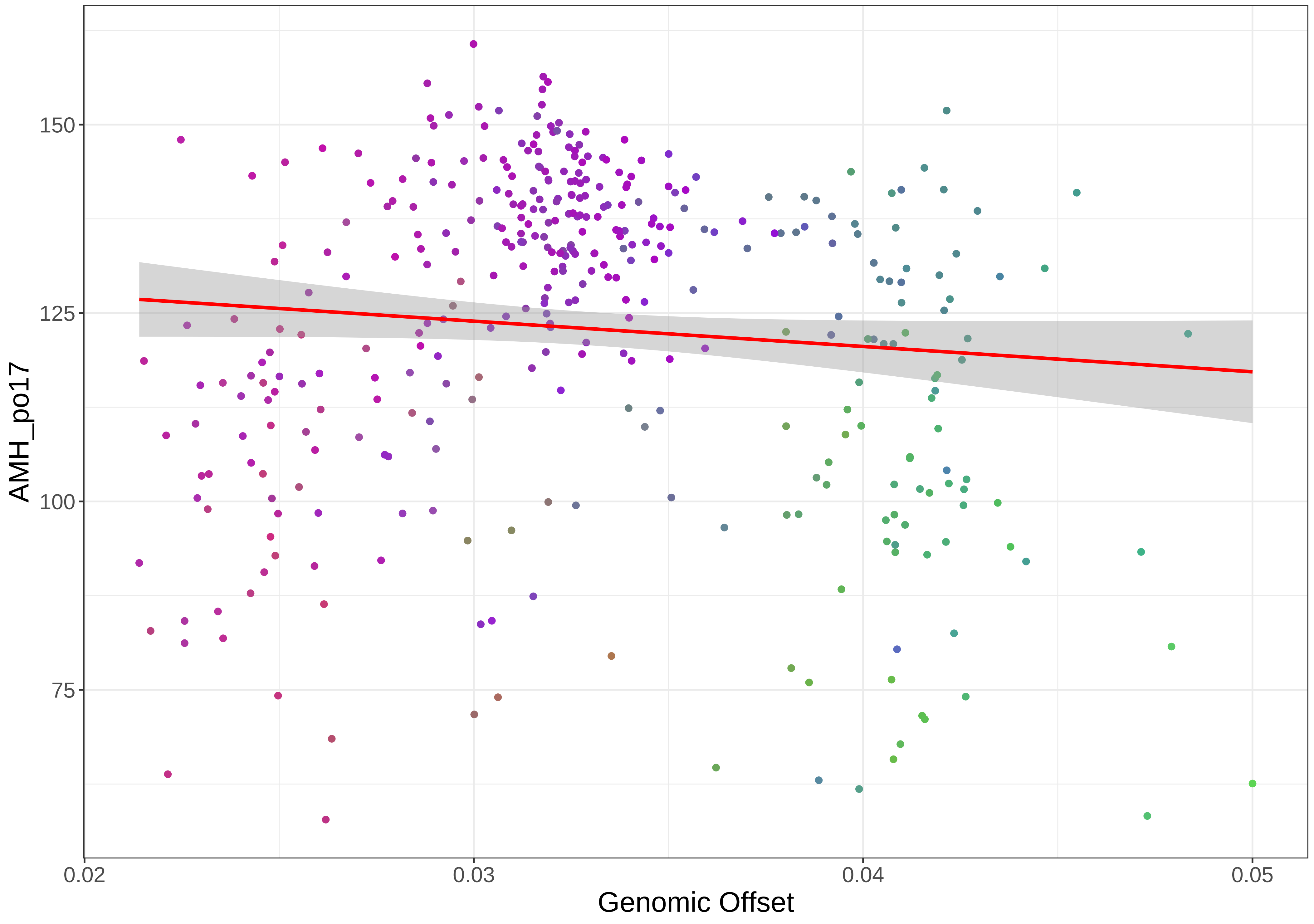

AMH_po17
150
125
100
75
0.02
0.03
0.04
0.05
Genomic Offset

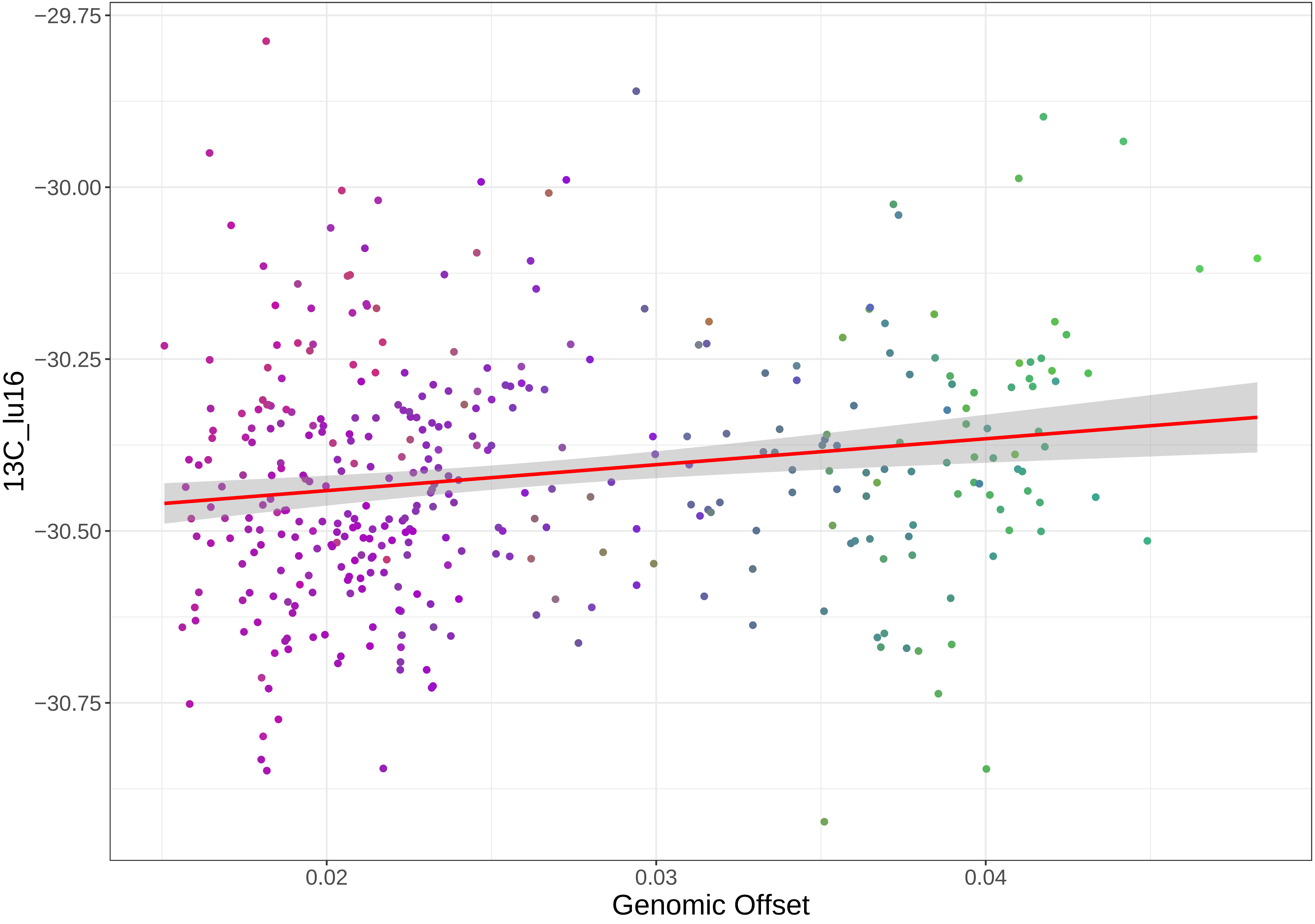

−29.75
−30.00
−30.25
−30.50
−30.75
13C_lu16
0.02
0.03
0.04
Genomic Offset

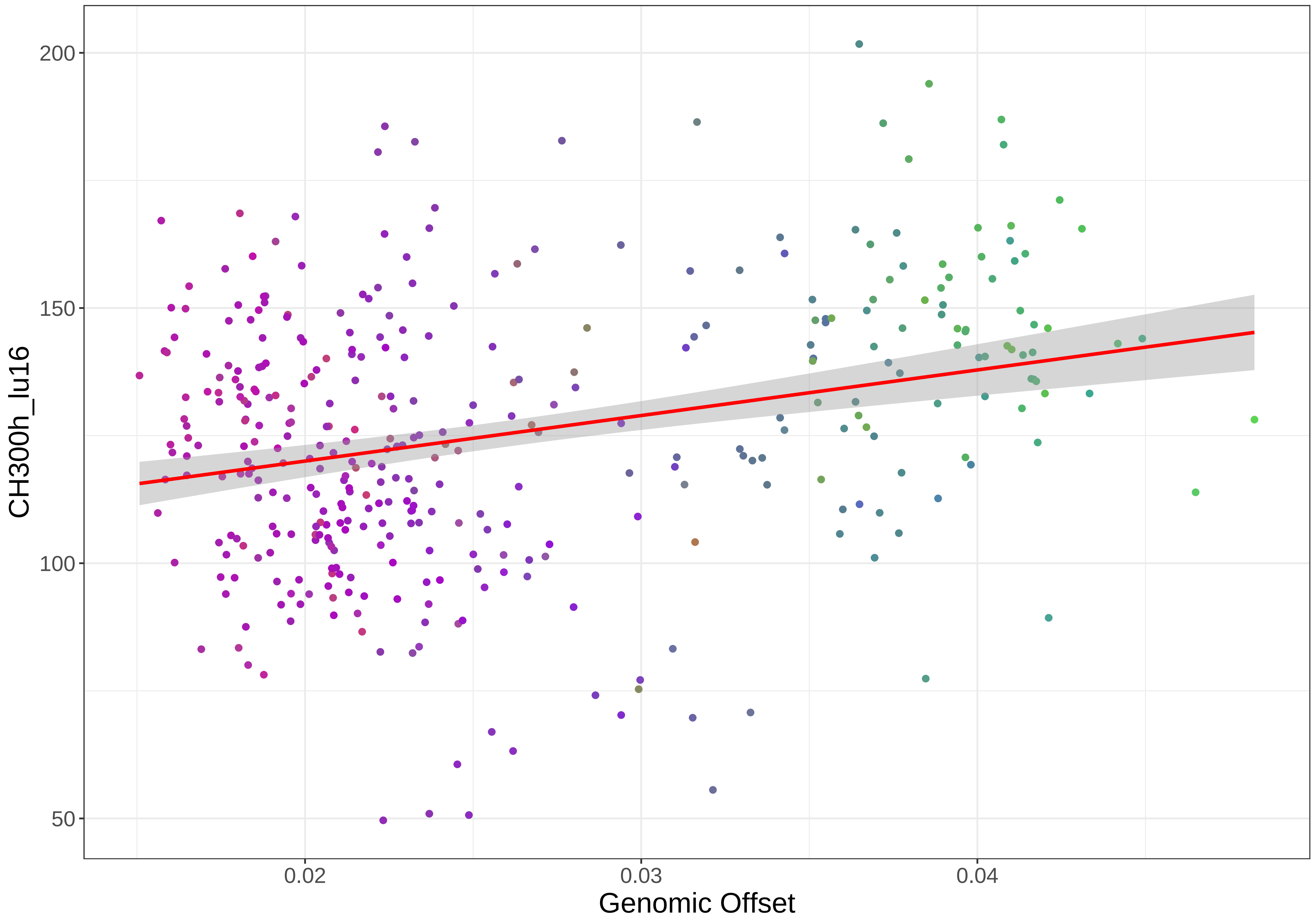

200
150
100
50
CH300h_lu16
0.02
0.03
0.04
Genomic Offset

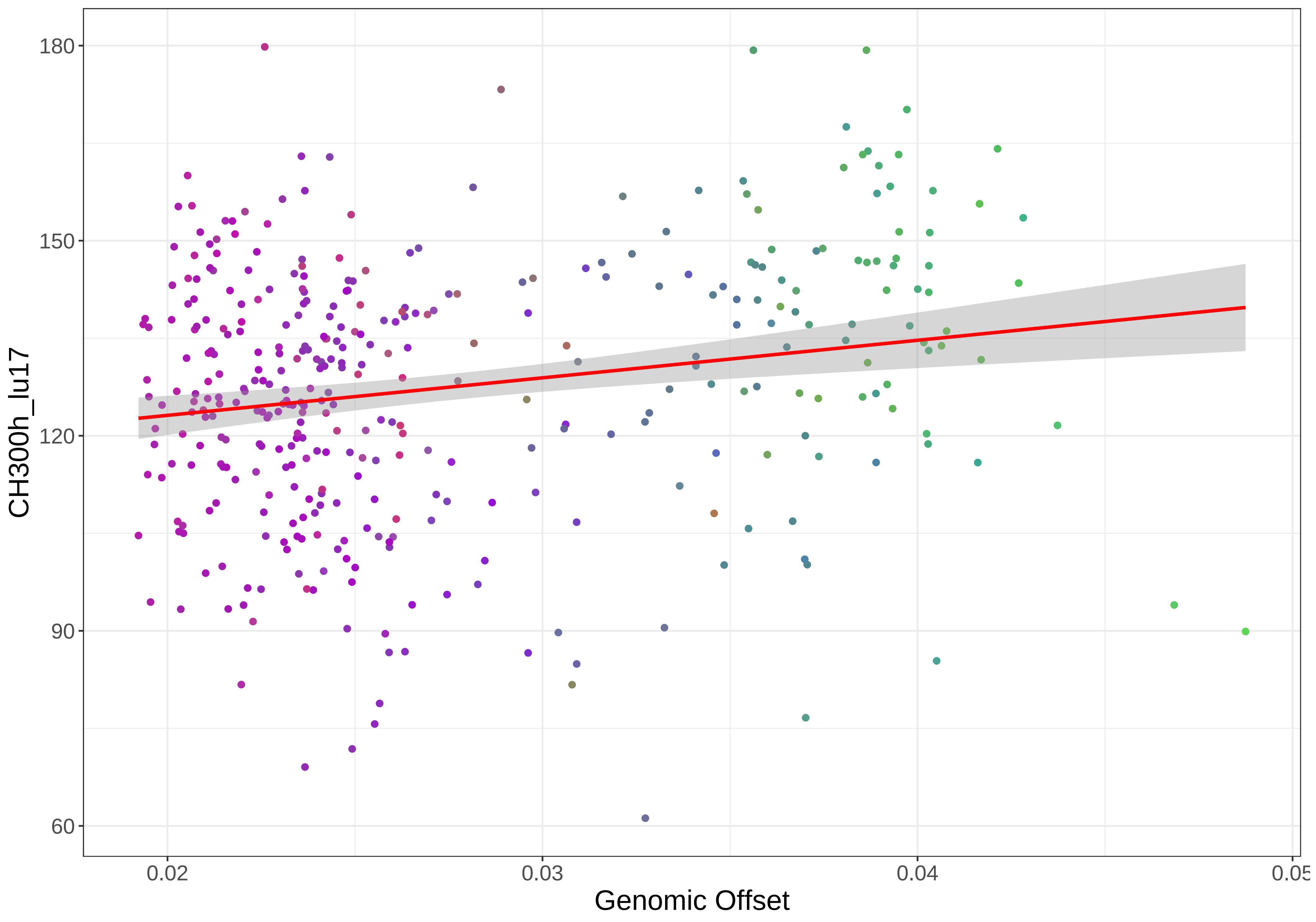

180
150
120
90
60
0.02
0.03
0.04
0.05
CH300h_lu17
Genomic Offset

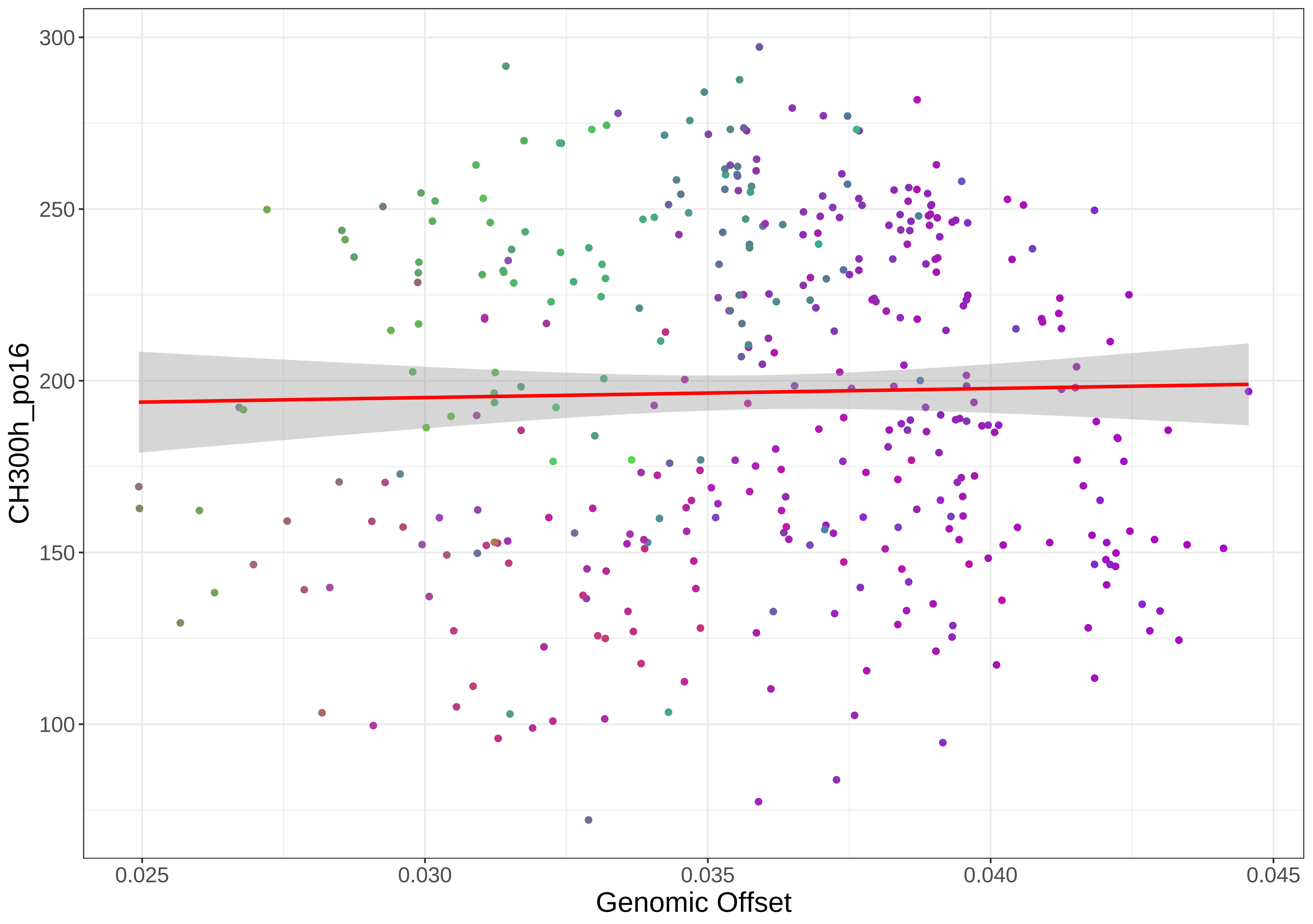

300
250
200
150
100
CH300h_po16
0.025
0.030
0.035
0.040
0.045
Genomic Offset

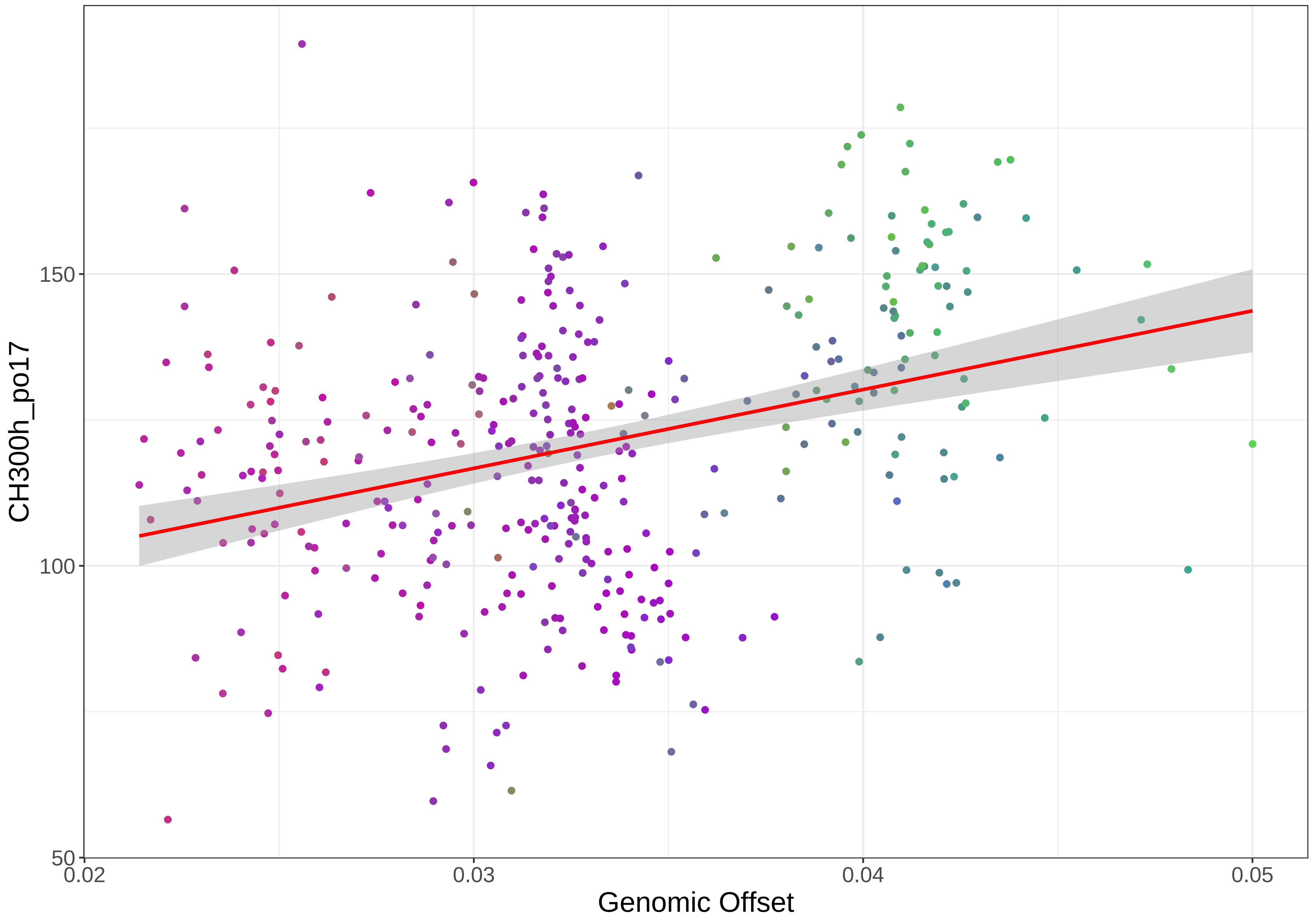

CH300h_po17
150
100
50
0.02
0.03
0.04
0.05
Genomic Offset

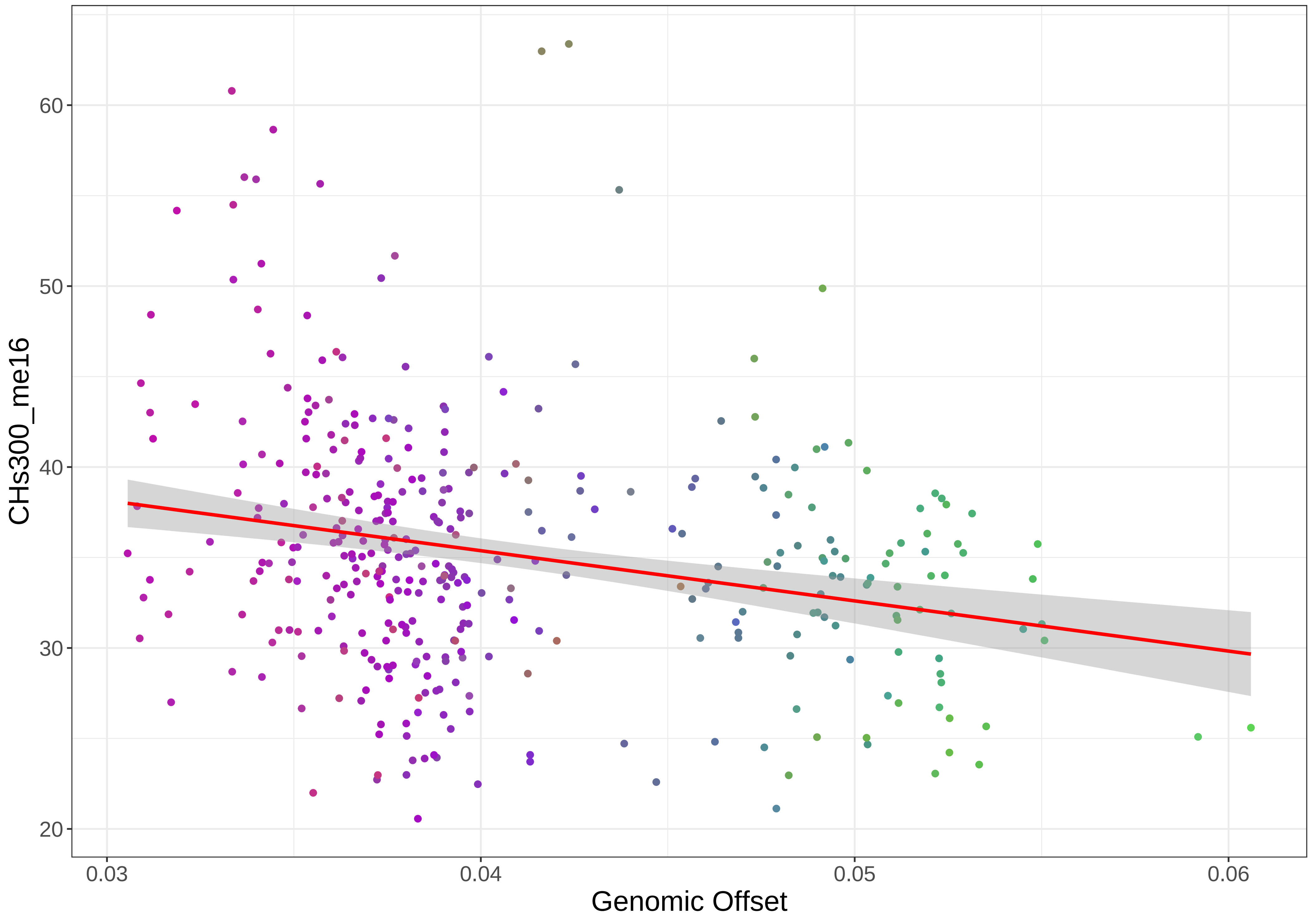

CHs300_me16
Genomic Offset
60
50
40
30
20
0.03
0.04
0.05
0.06

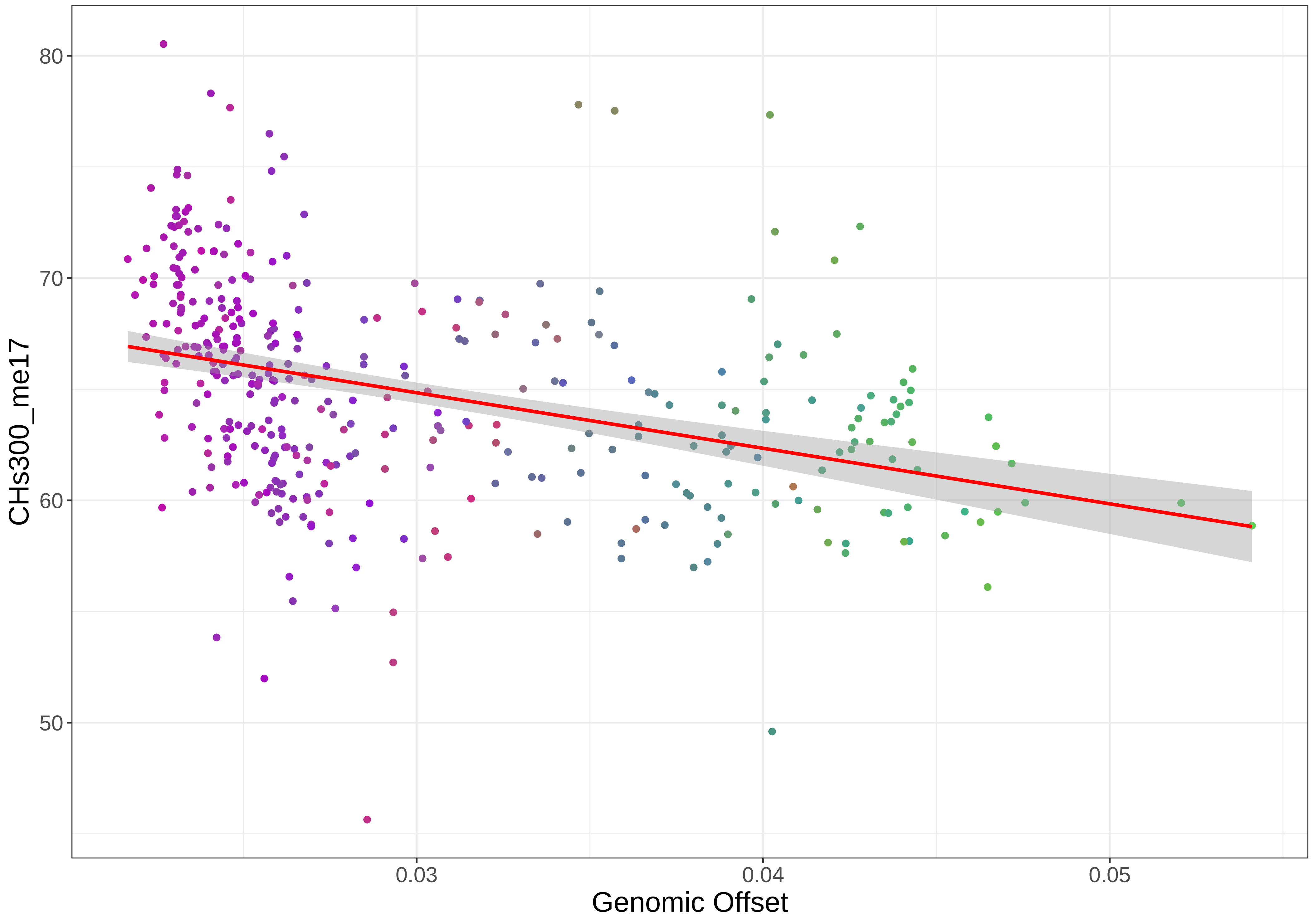

80
70
60
50
CHs300_me17
0.03
0.04
0.05
Genomic Offset

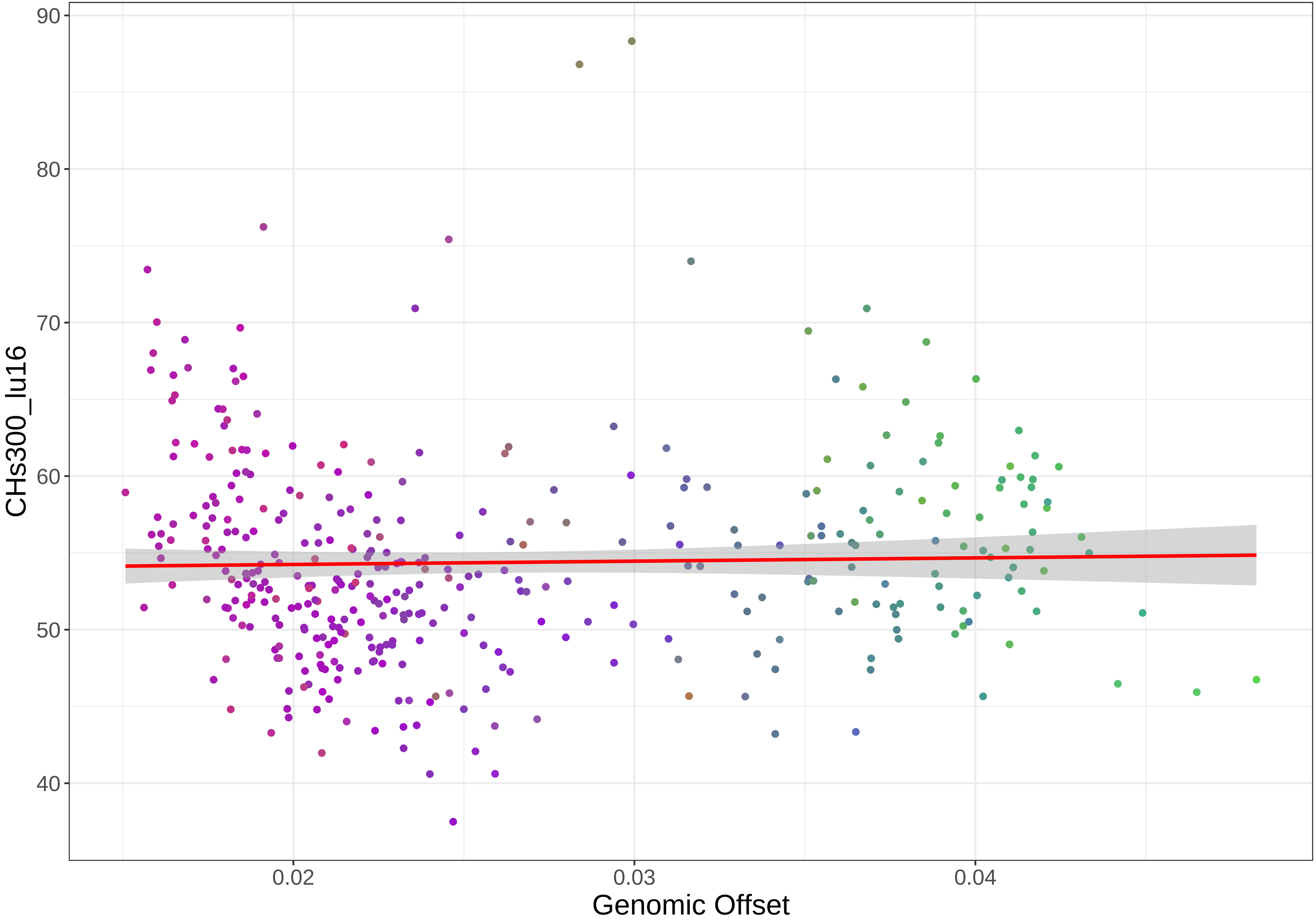

90
80
70
60
50
40
CHs300_lu16
0.02
0.03
0.04
Genomic Offset

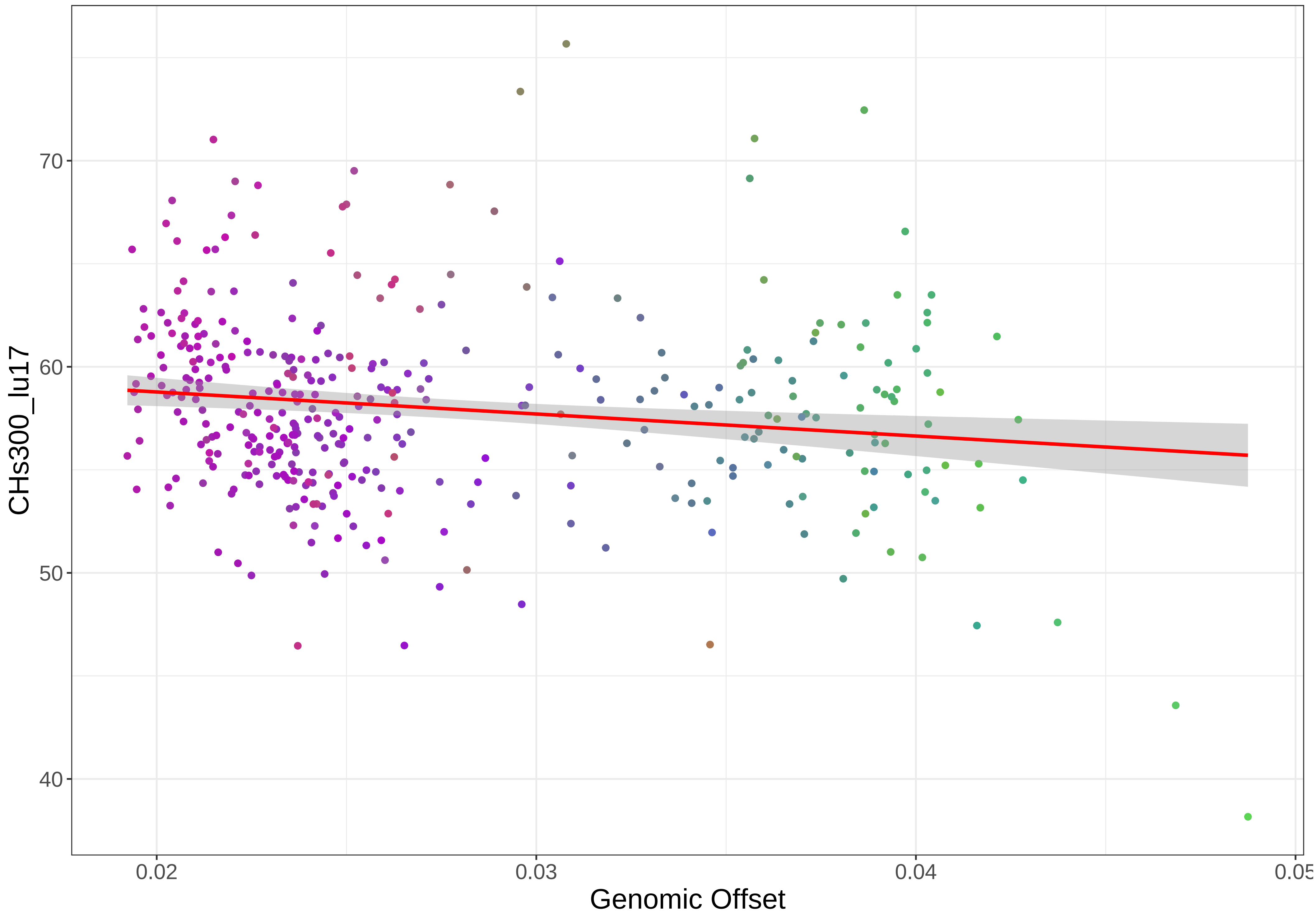

CHs300_lu17
70
60
50
40
0.02
0.03
0.04
0.05
Genomic Offset

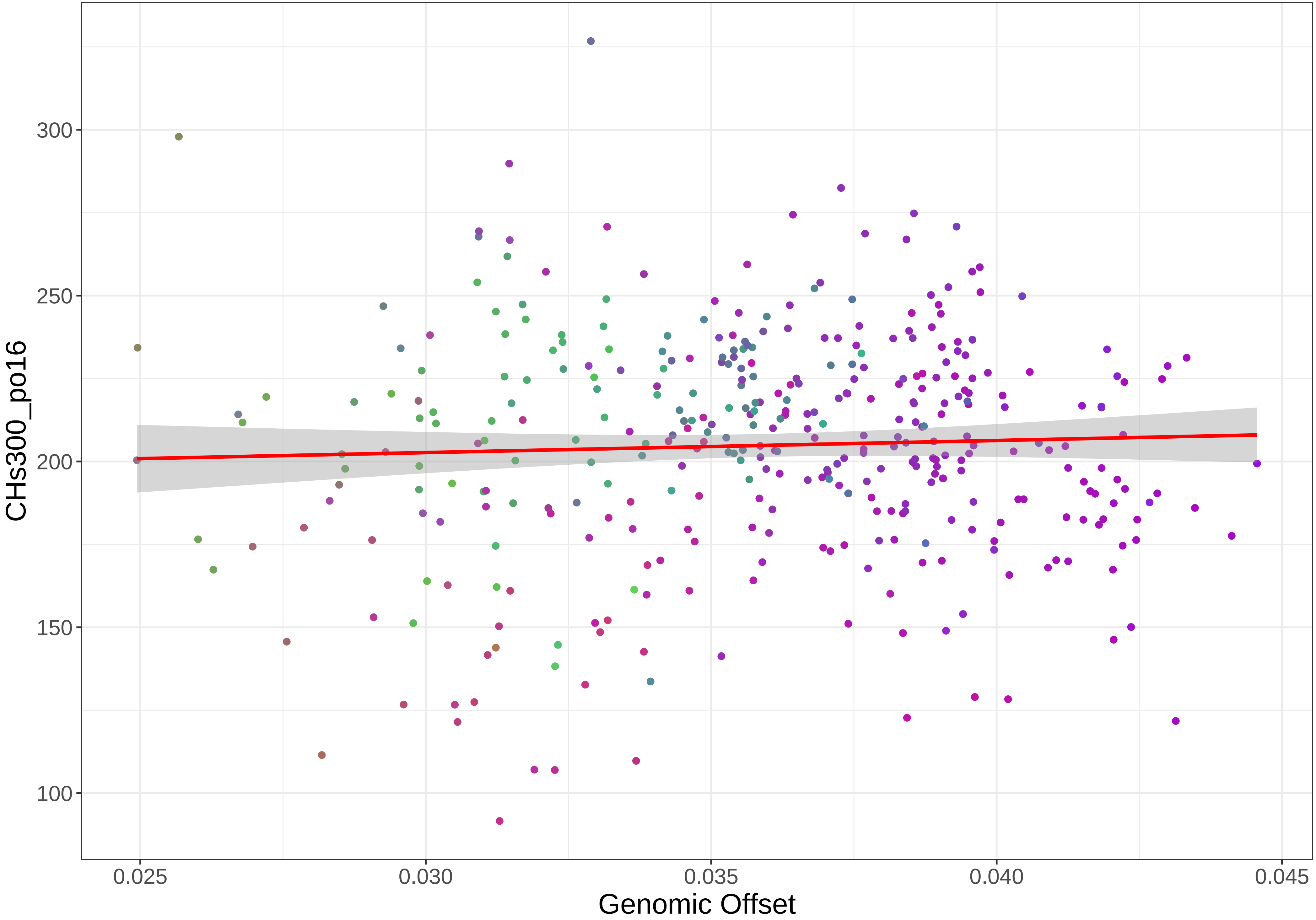

CHs300_po16
300
250
200
150
100
0.025
0.030
0.035
0.040
0.045
Genomic Offset

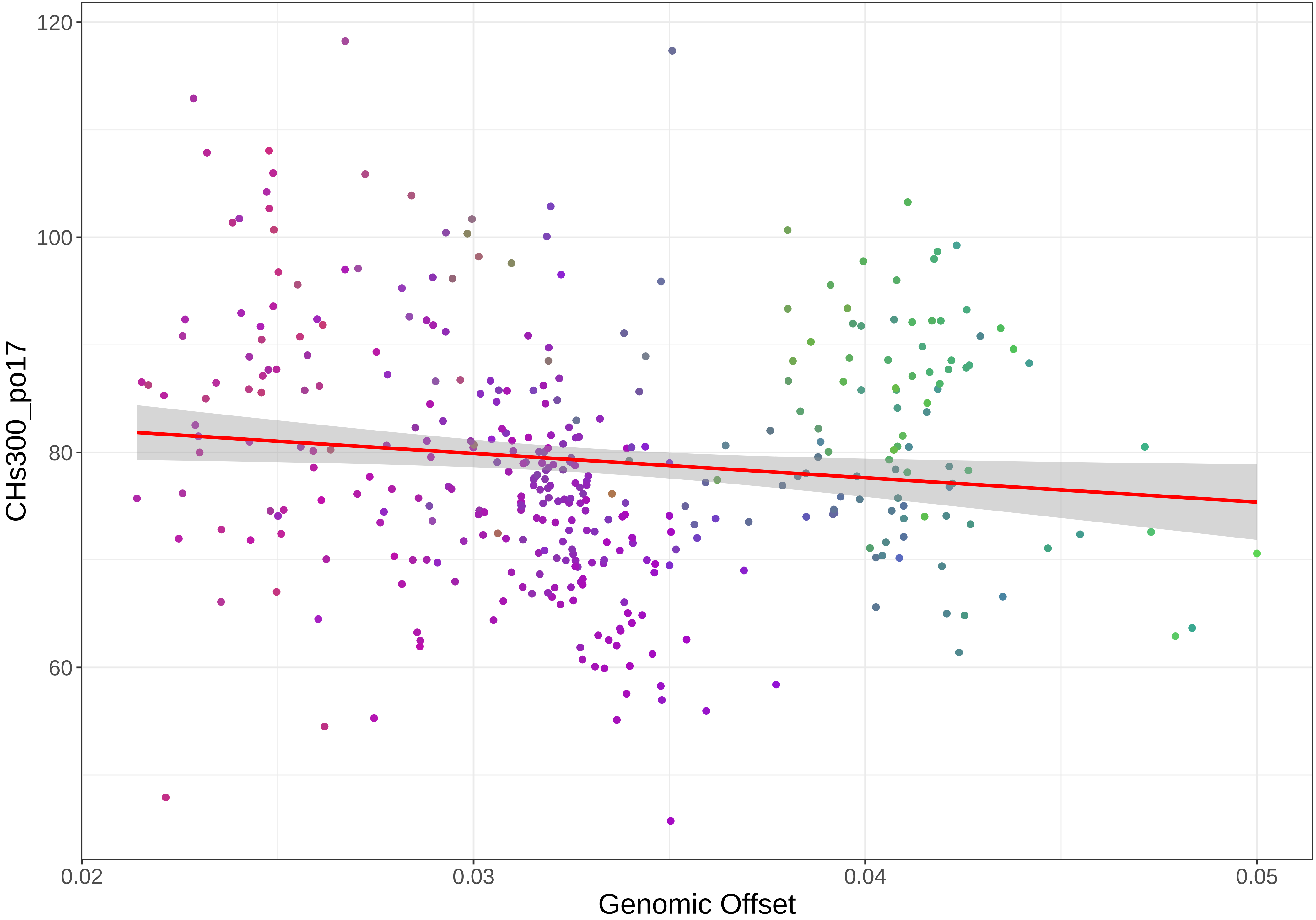

120
100
80
60
CHs300_po17
0.02
0.03
0.04
0.05
Genomic Offset

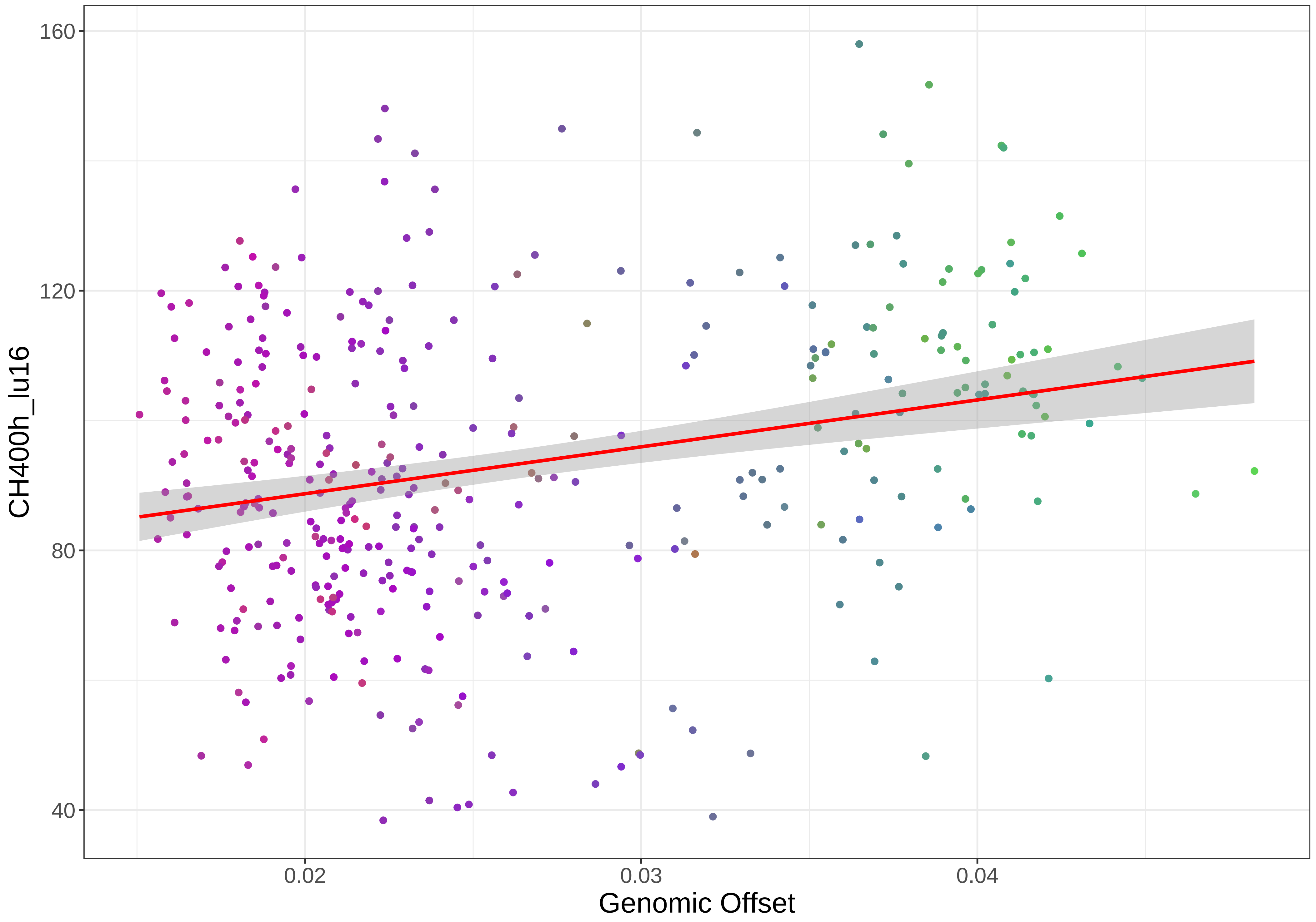

160
120
80
40
0.02
0.03
0.04
Genomic Offset
CH400h_lu16

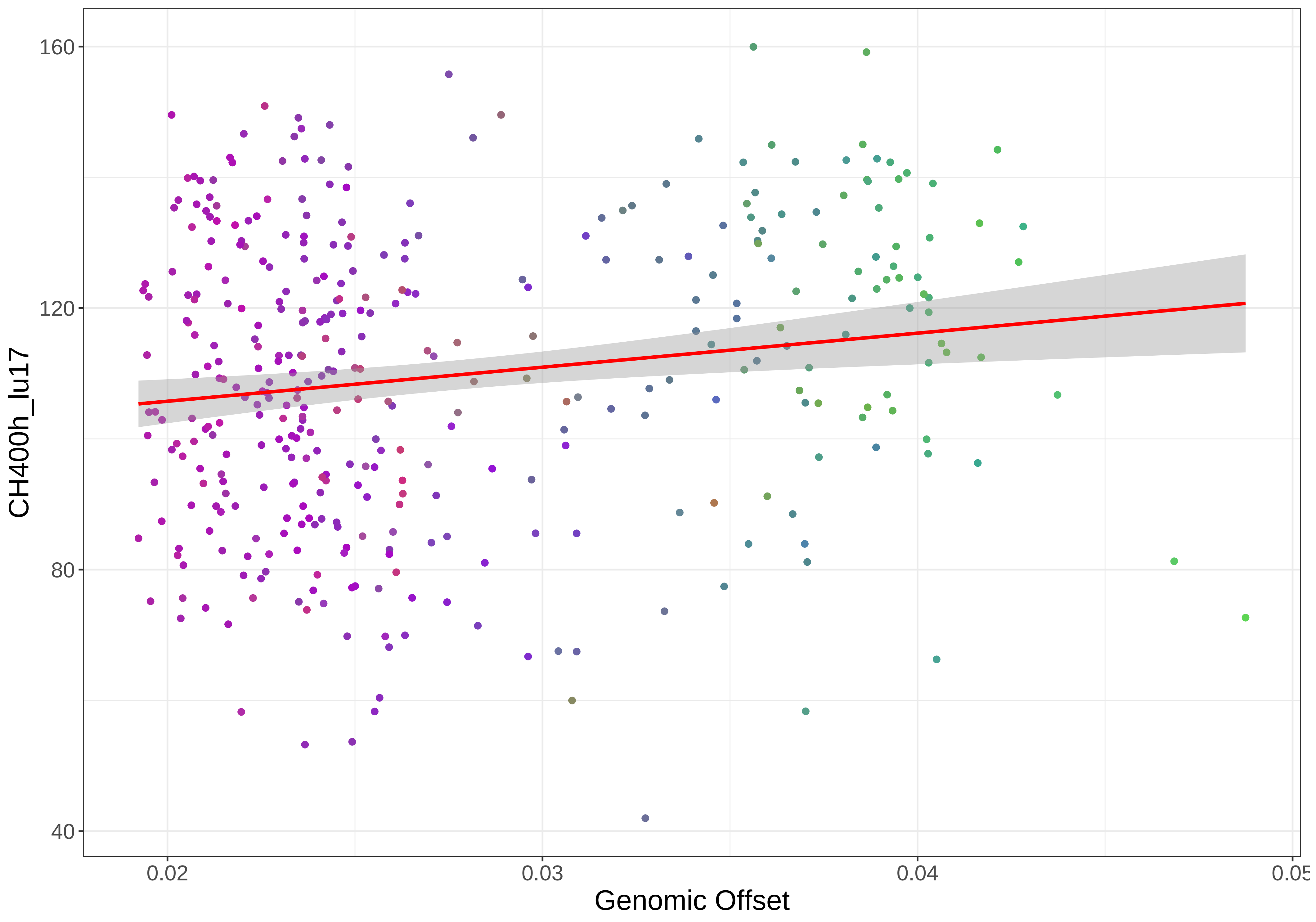

160
120
80
40
CH400h_lu17
0.02
0.03
0.04
0.05
Genomic Offset

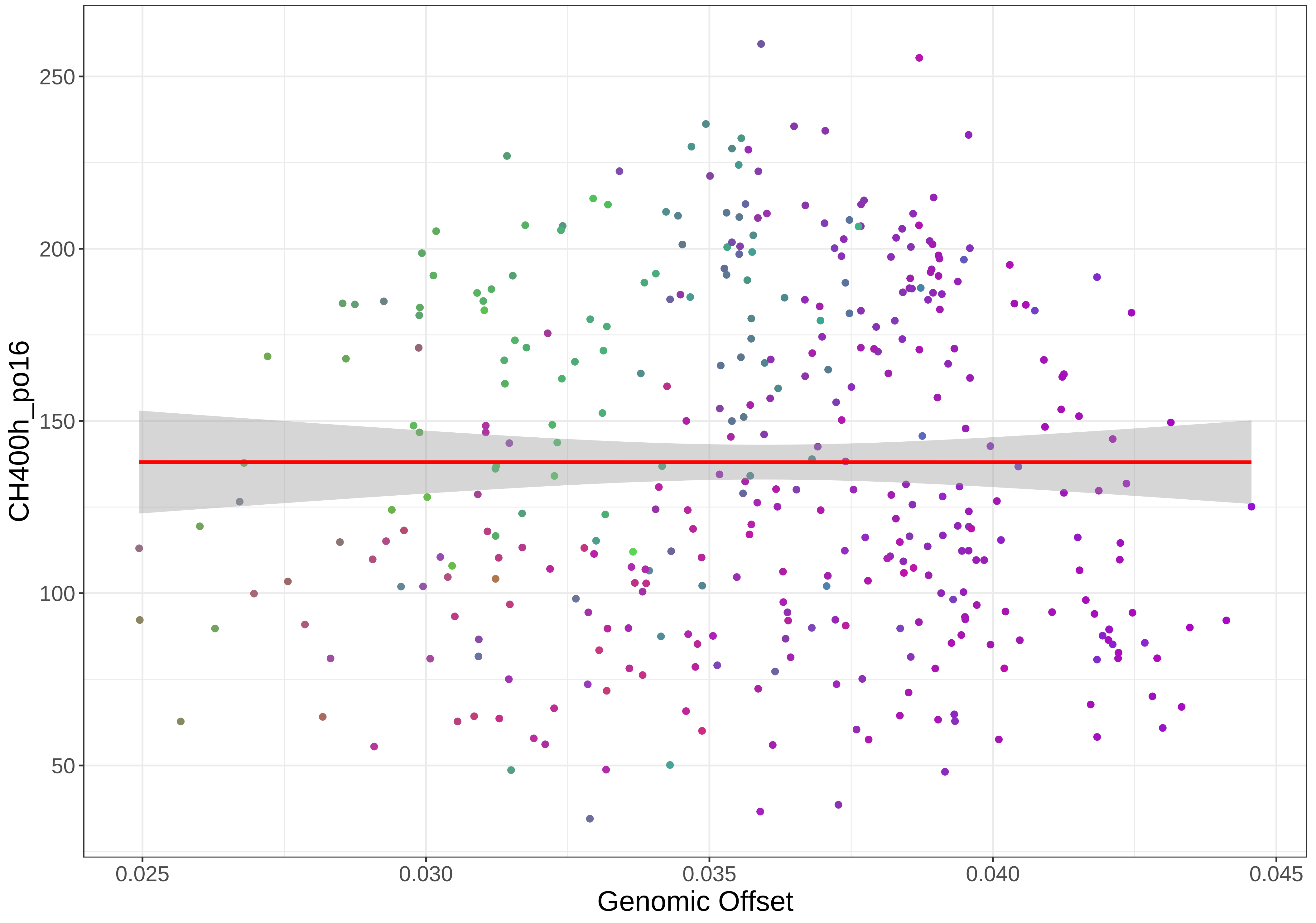
CH400h_po16
Genomic Offset
250
200
150
100
50
0.025
0.030
0.035
0.040
0.045

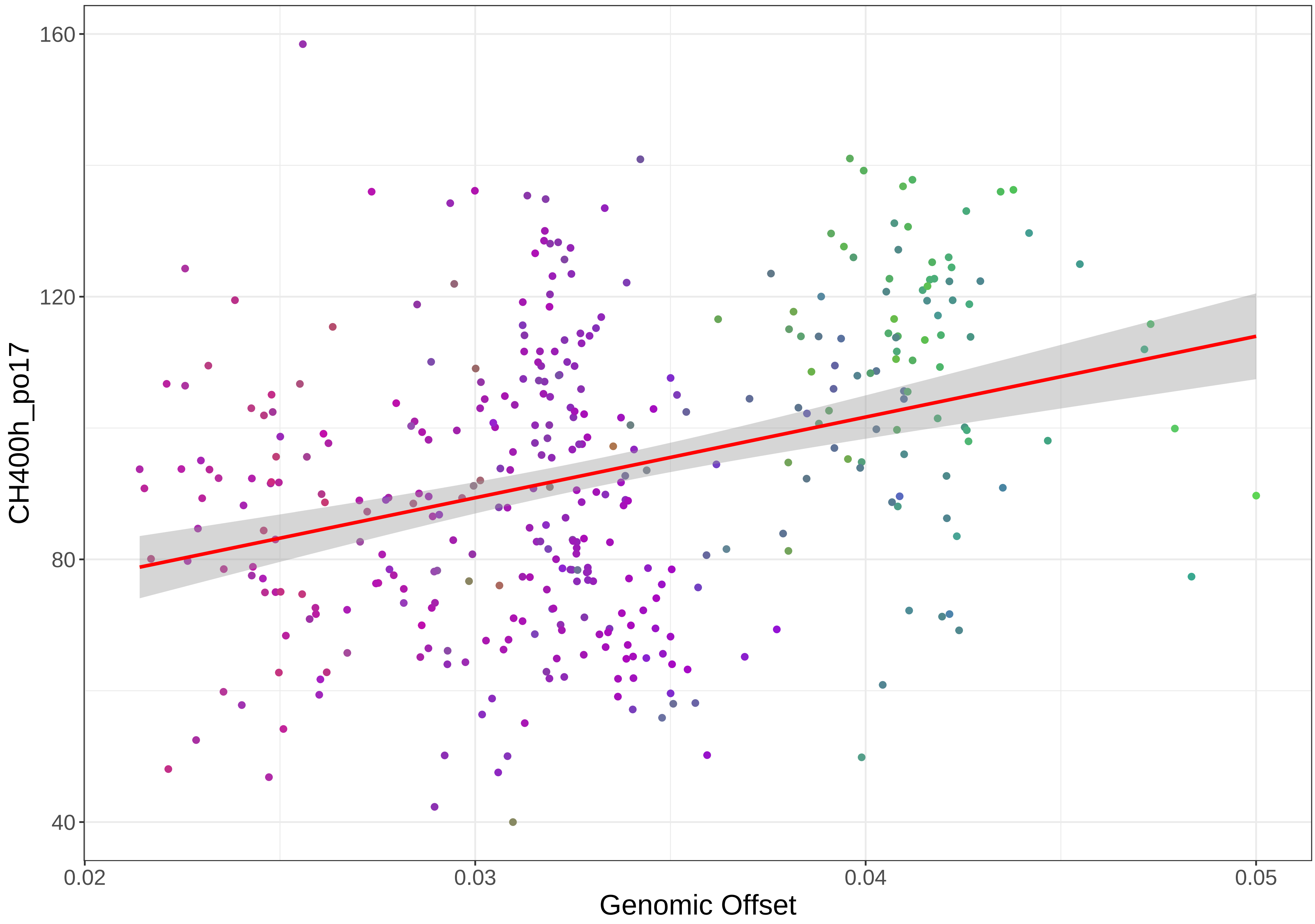

160
120
80
40
CH400h_po17
0.02
0.03
0.04
0.05
Genomic Offset

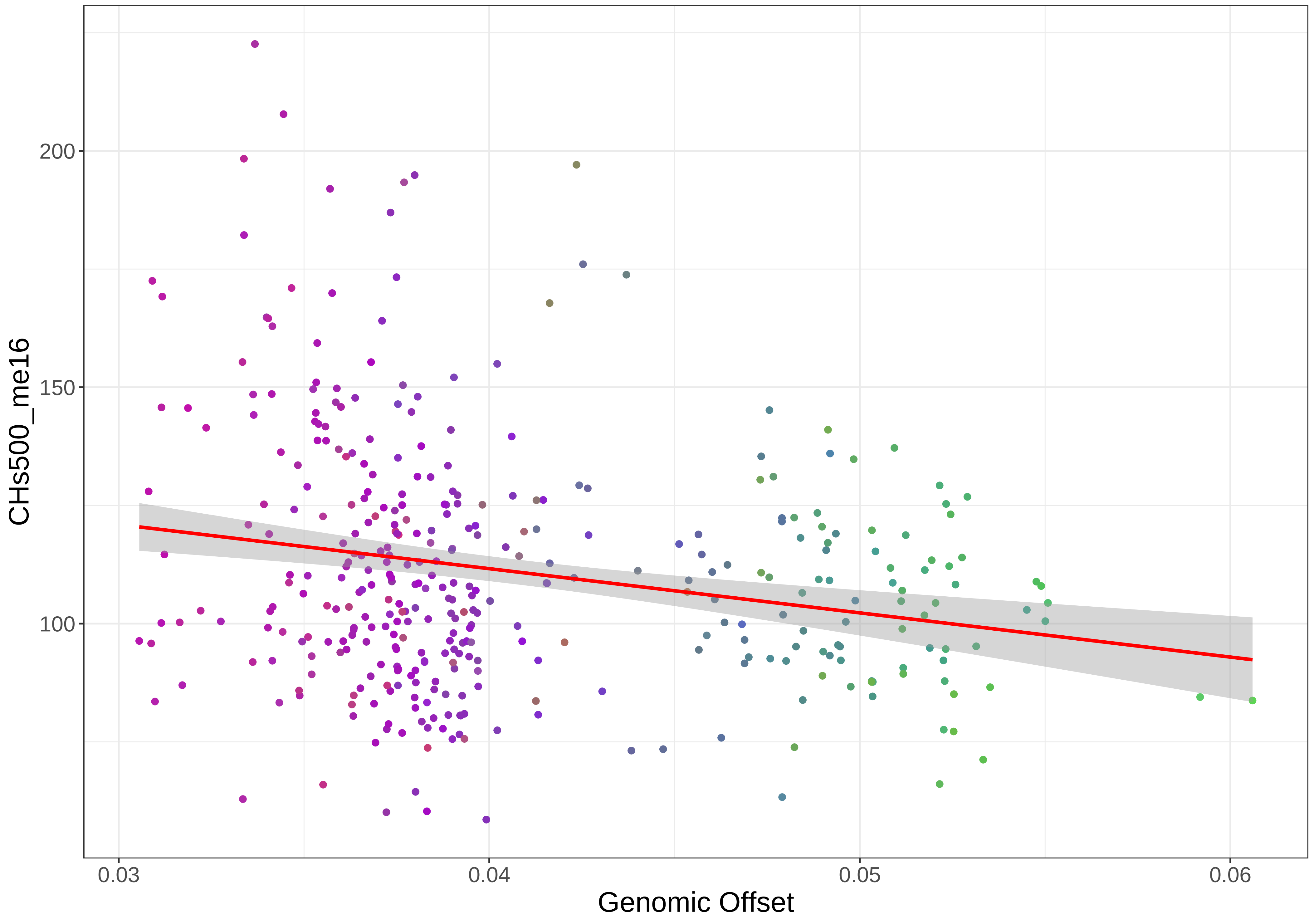
CHs500_me16
200
150
100
Genomic Offset
0.03
0.04
0.05
0.06

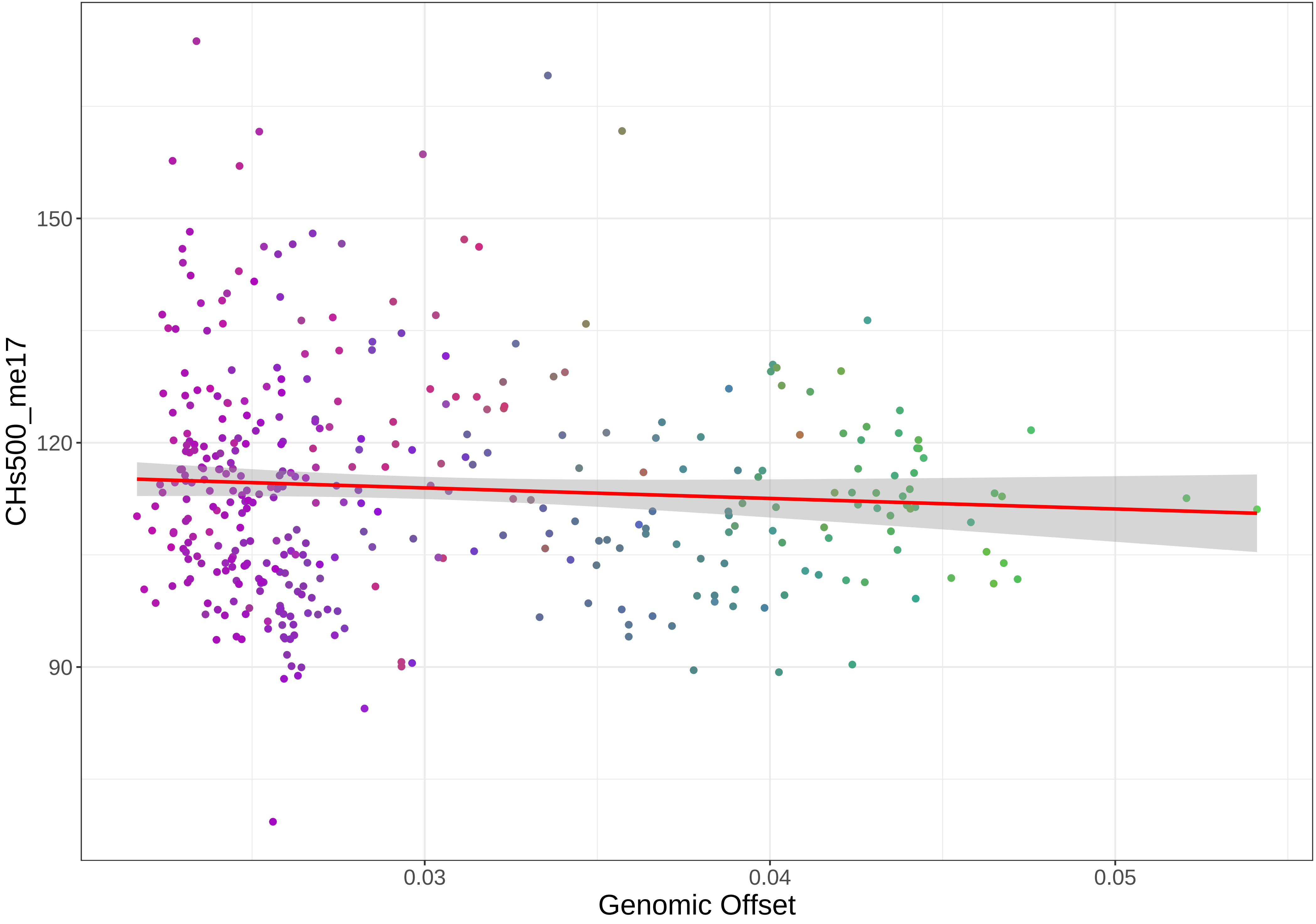

150
120
90
CHs500_me17
0.03
0.04
0.05
Genomic Offset

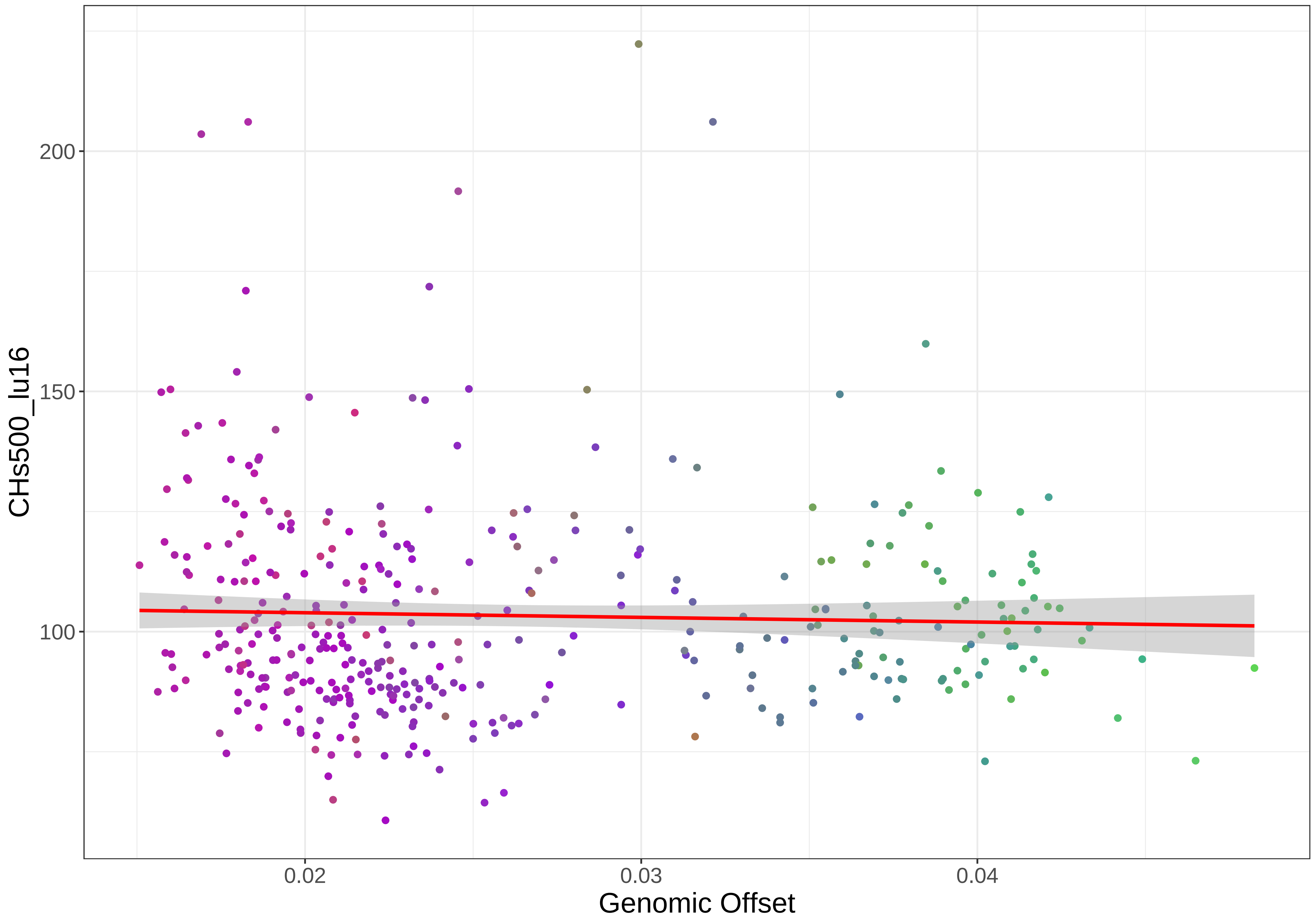

200
150
100
CHs500_lu16
0.02
0.03
0.04
Genomic Offset

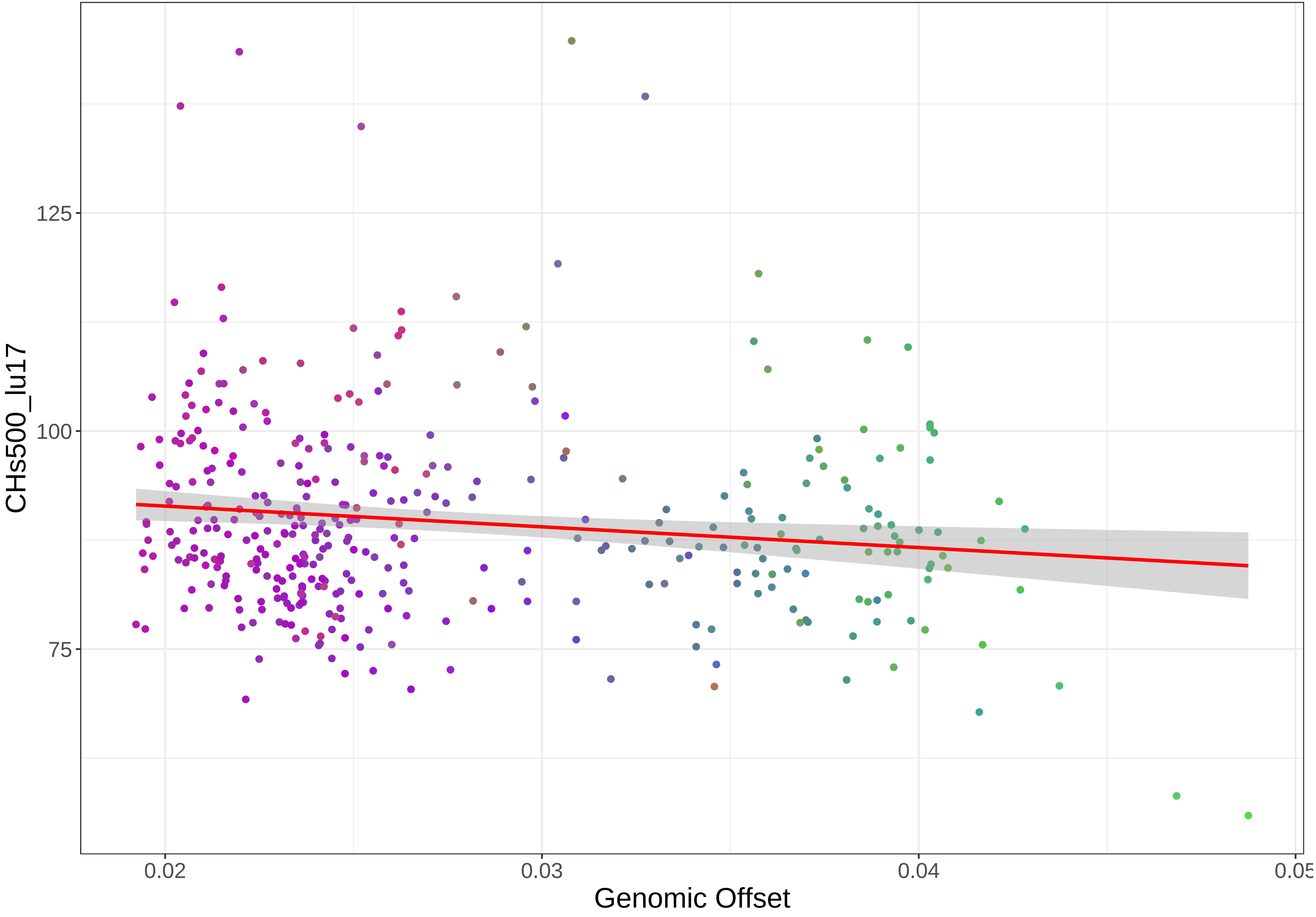

CHs500_lu17
125
100
75
0.02
0.03
0.04
Genomic Offset

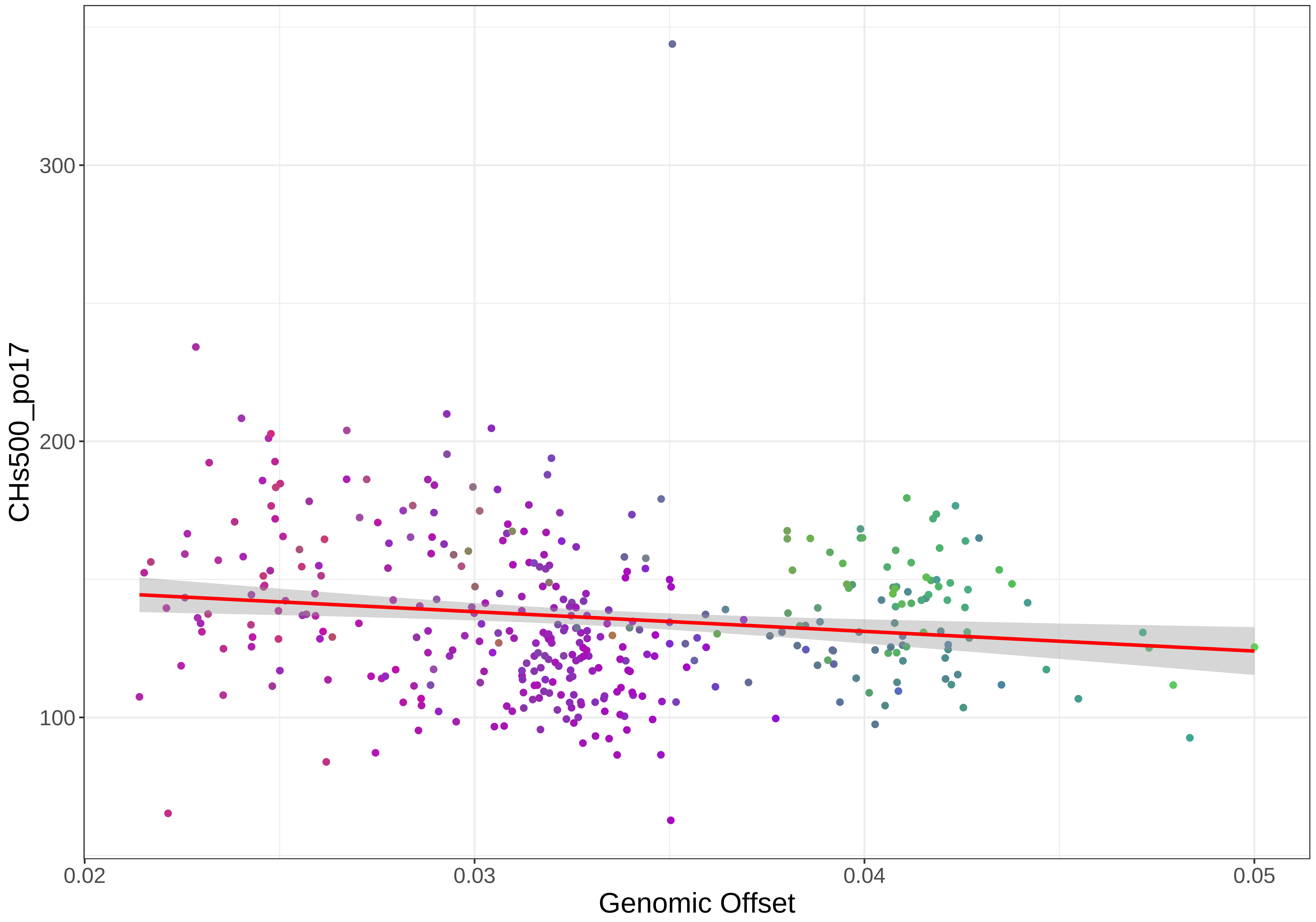

CHs500_po17
300
200
100
0.02
0.03
0.04
0.05
Genomic Offset

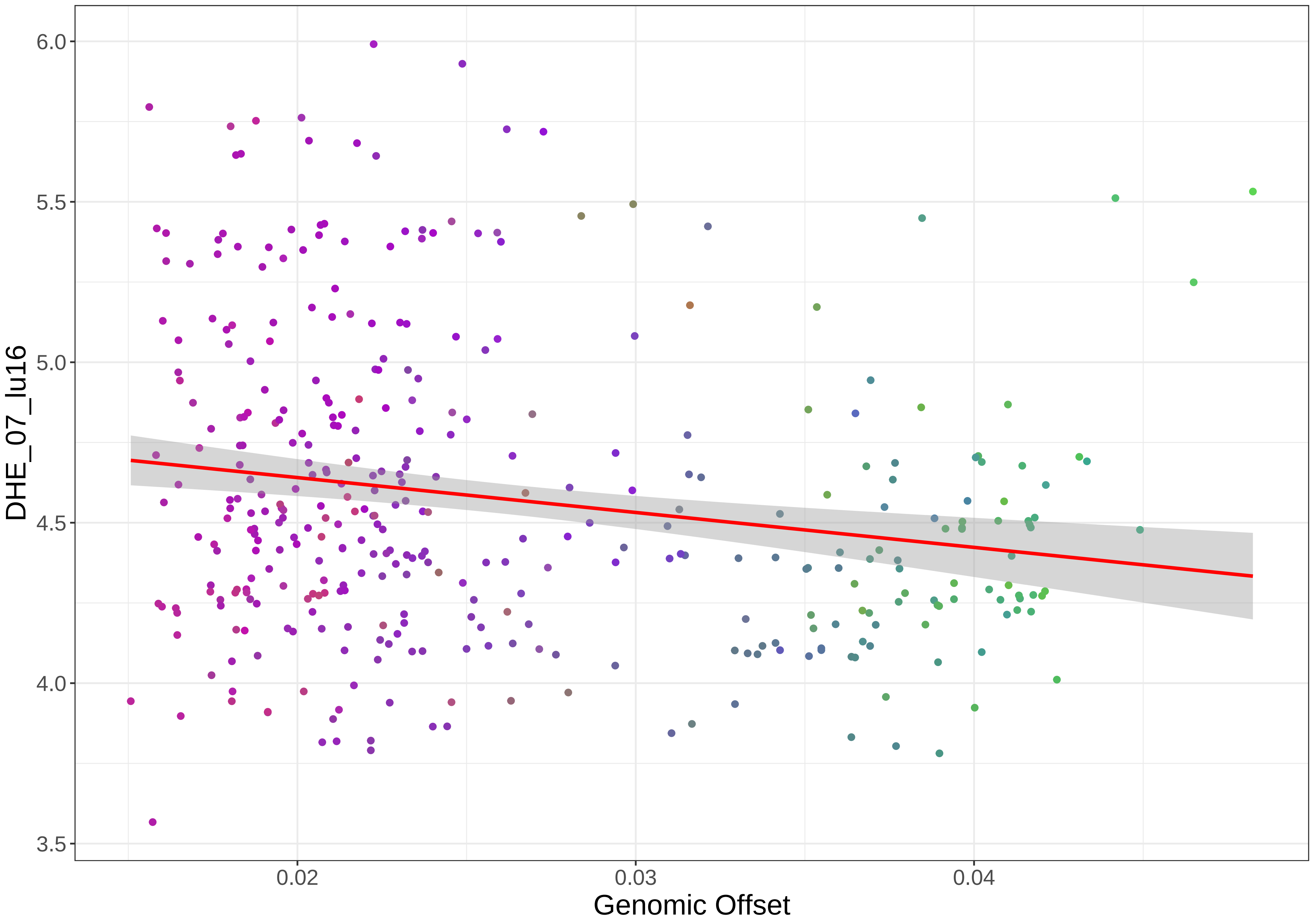
6.0
5.5
5.0
4.5
4.0
3.5
DHE_07_lu16
0.02
0.03
0.04
Genomic Offset

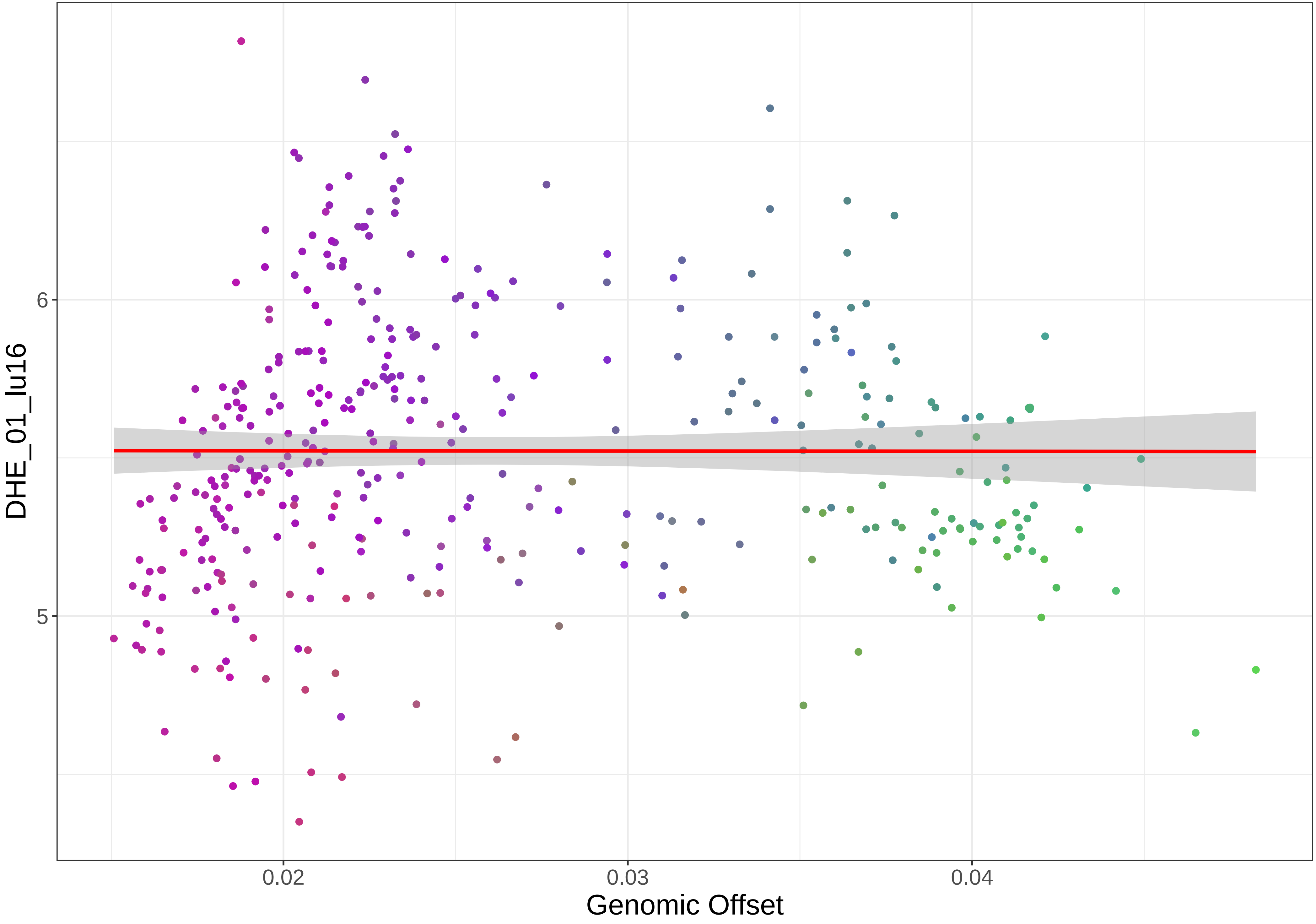
DHE_01_lu16
6
5
0.02
0.03
0.04
Genomic Offset

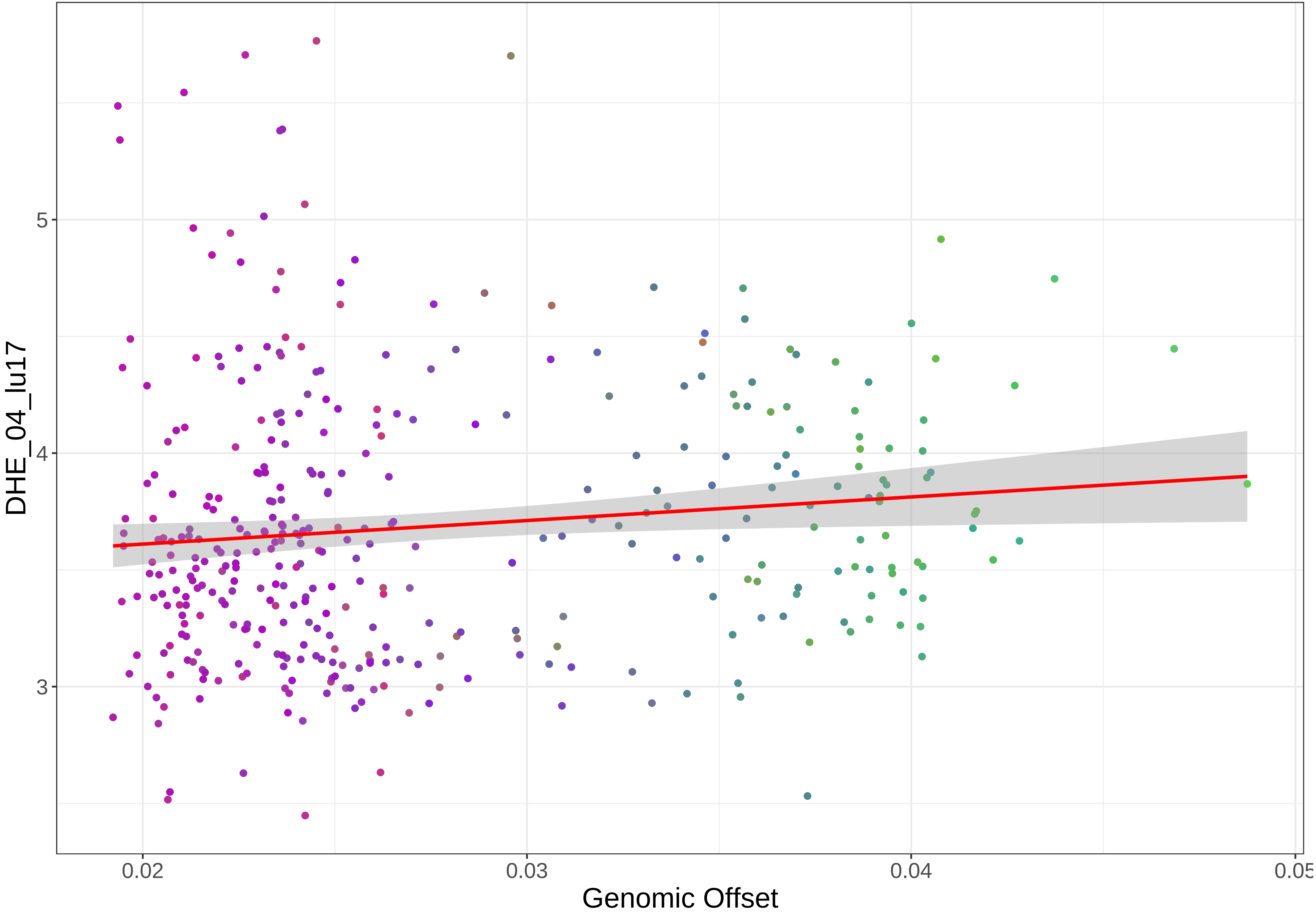

DHE_04_lu17
5
4
3
0.02
0.03
0.04
0.05
Genomic Offset

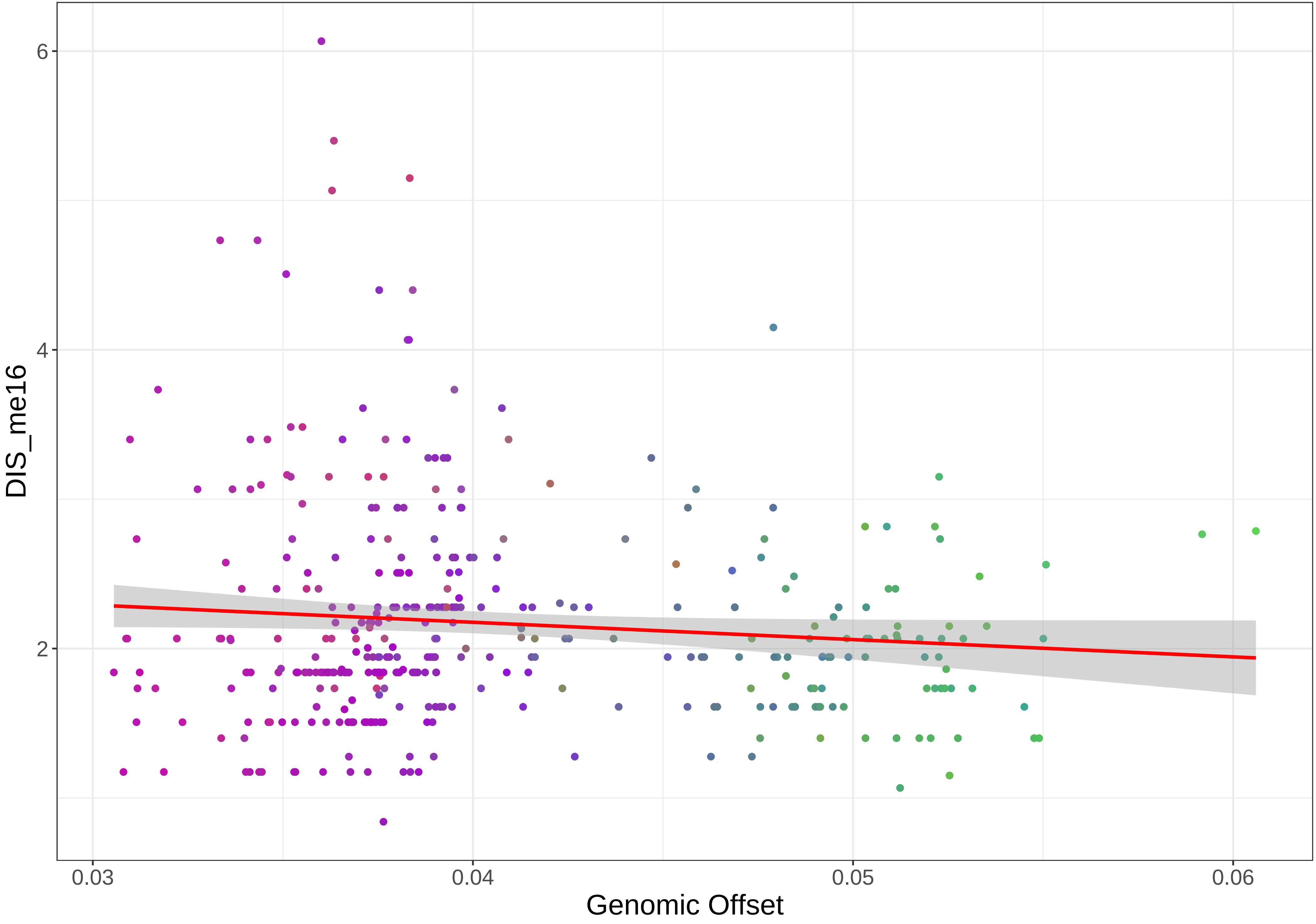
DIS_me16
6
4
2
0.03
0.04
0.05
0.06
Genomic Offset

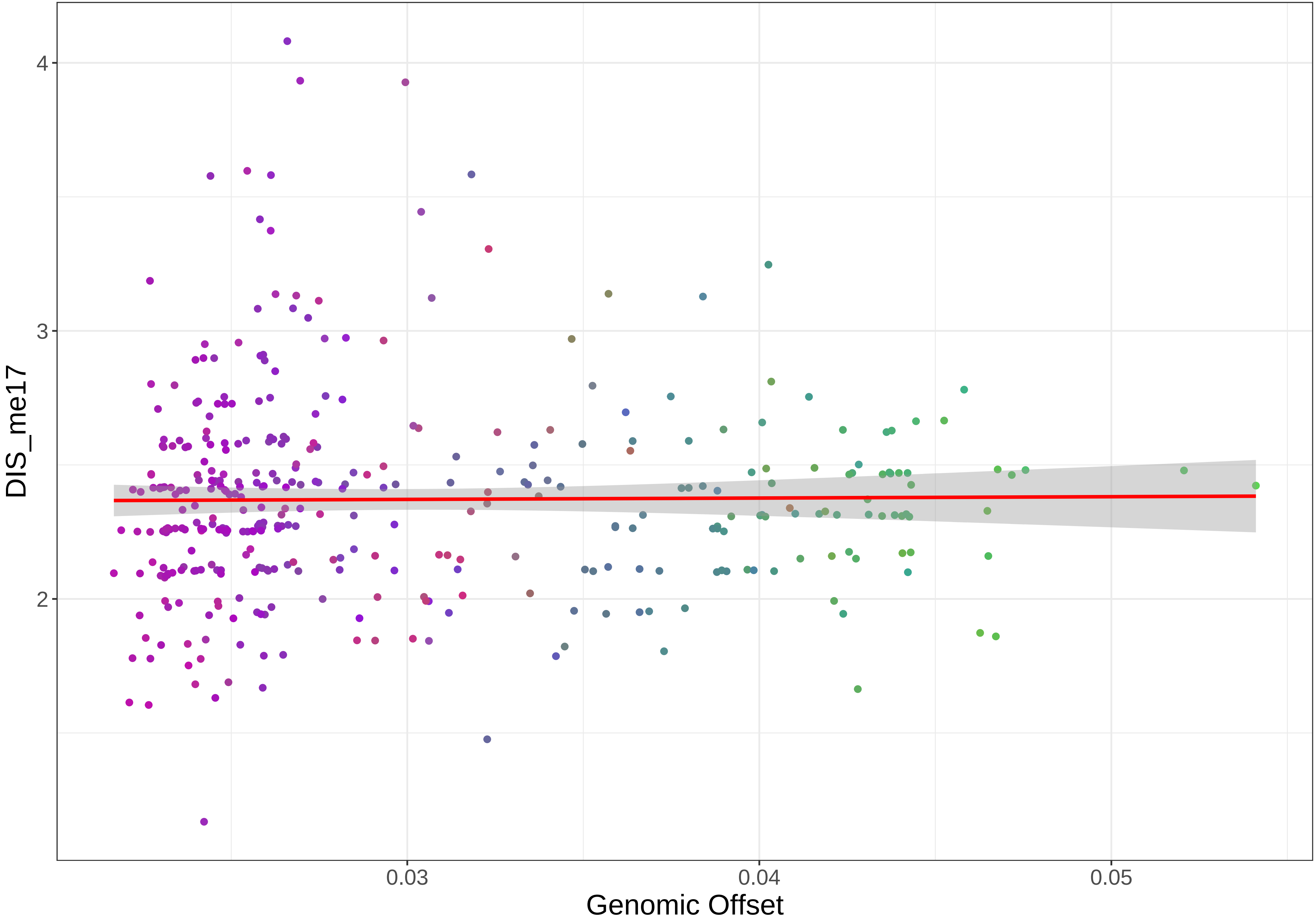

4
3
2
DIS_me17
0.03
0.04
0.05
Genomic Offset

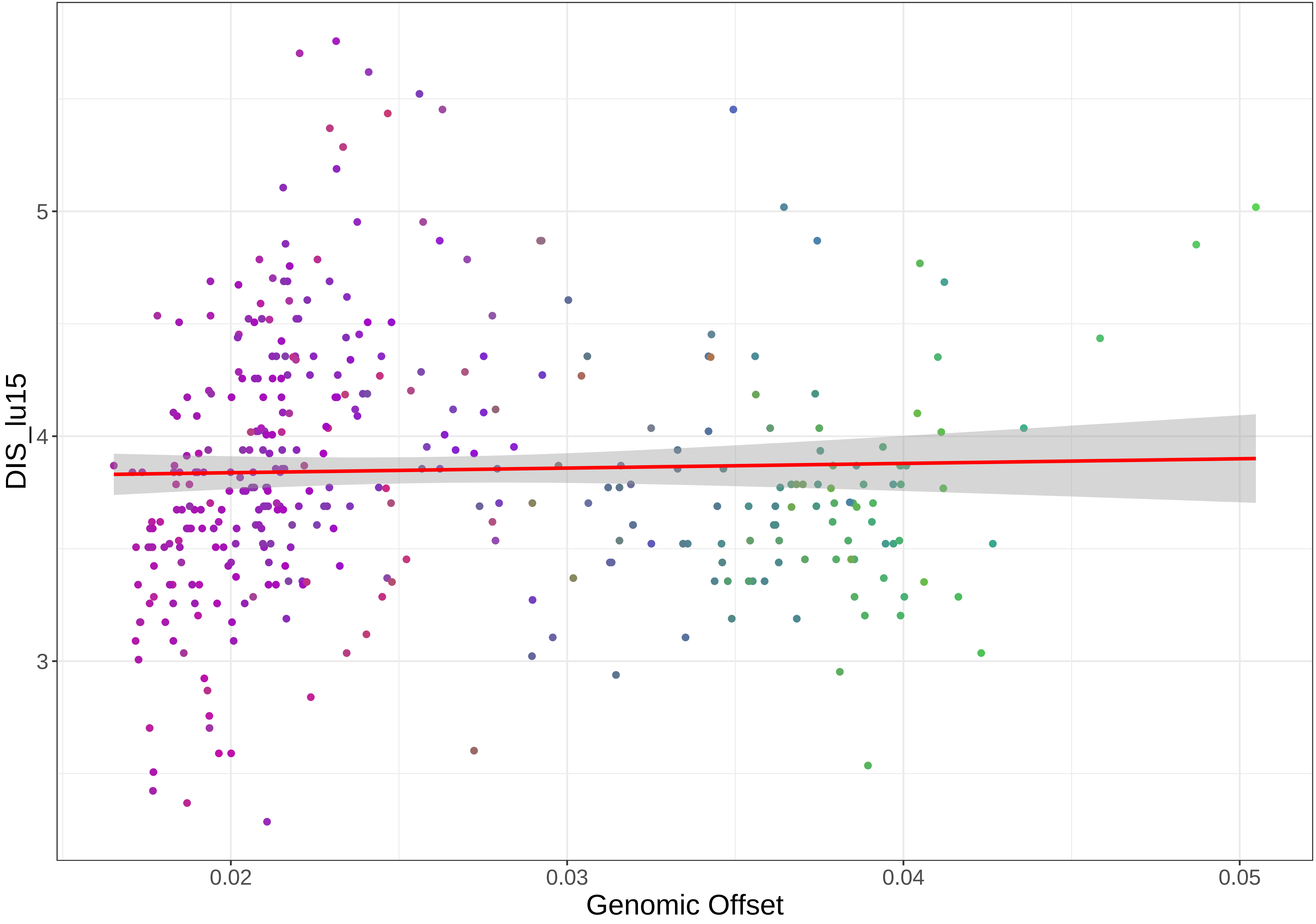
DIS_lu15
5
4
3
0.02
0.03
0.04
0.05
Genomic Offset

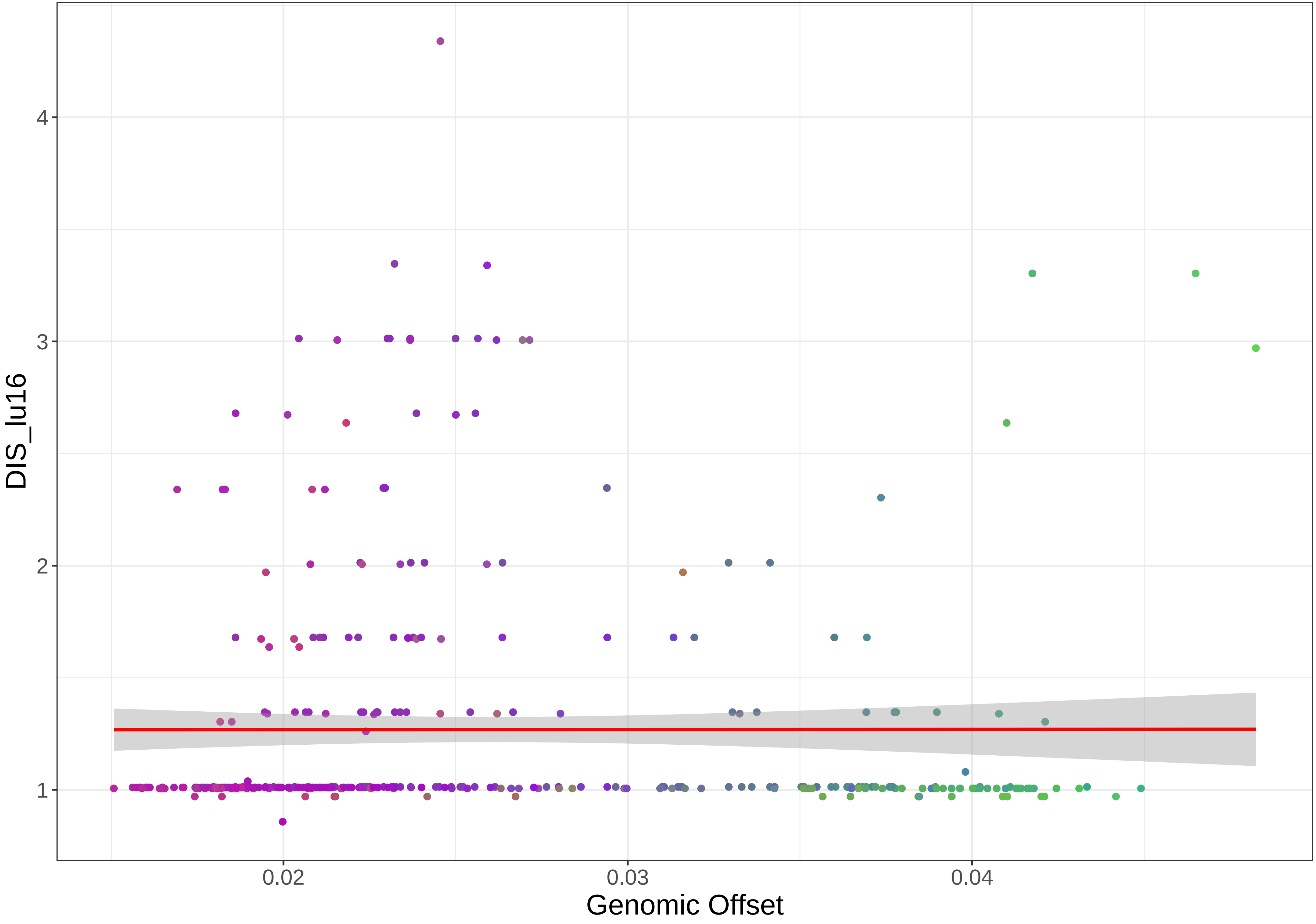

4
3
2
1
0.02
0.03
0.04
DIS_lu16
Genomic Offset

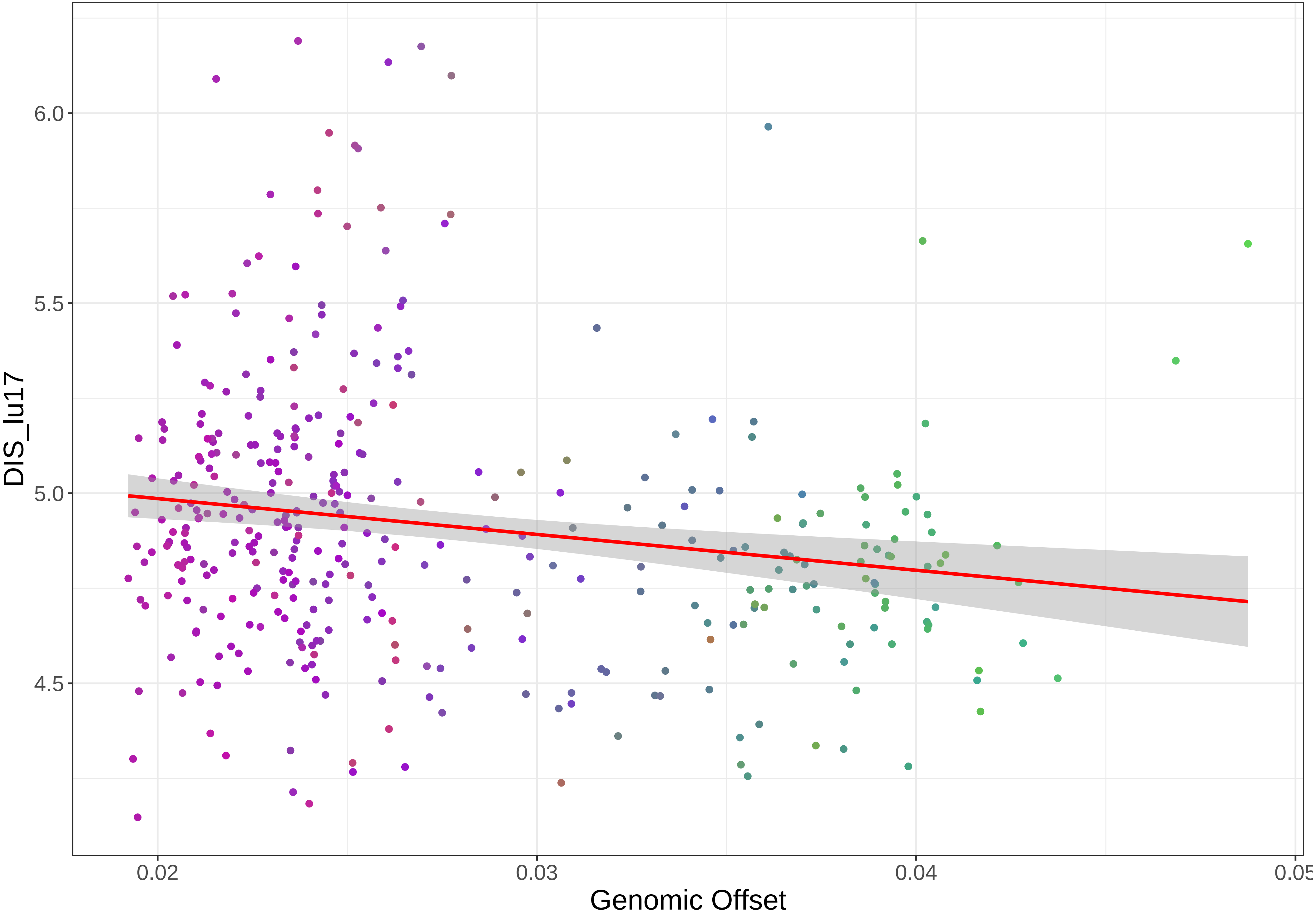
6.0
5.5
5.0
4.5
DIS_lu17
0.02
0.03
0.04
0.05
Genomic Offset

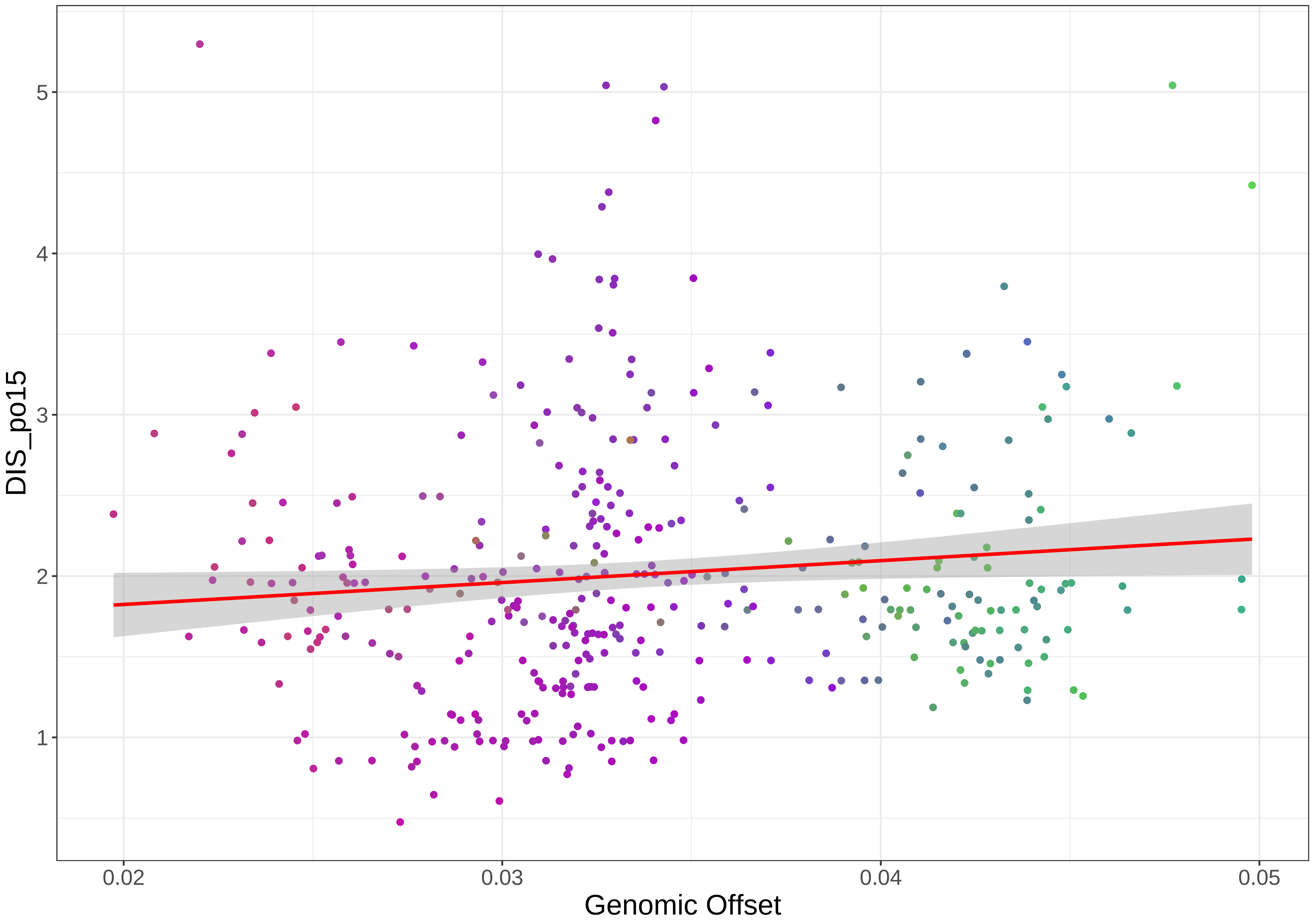
DIS_po15
5
4
3
2
1
0.02
0.03
0.04
0.05
Genomic Offset

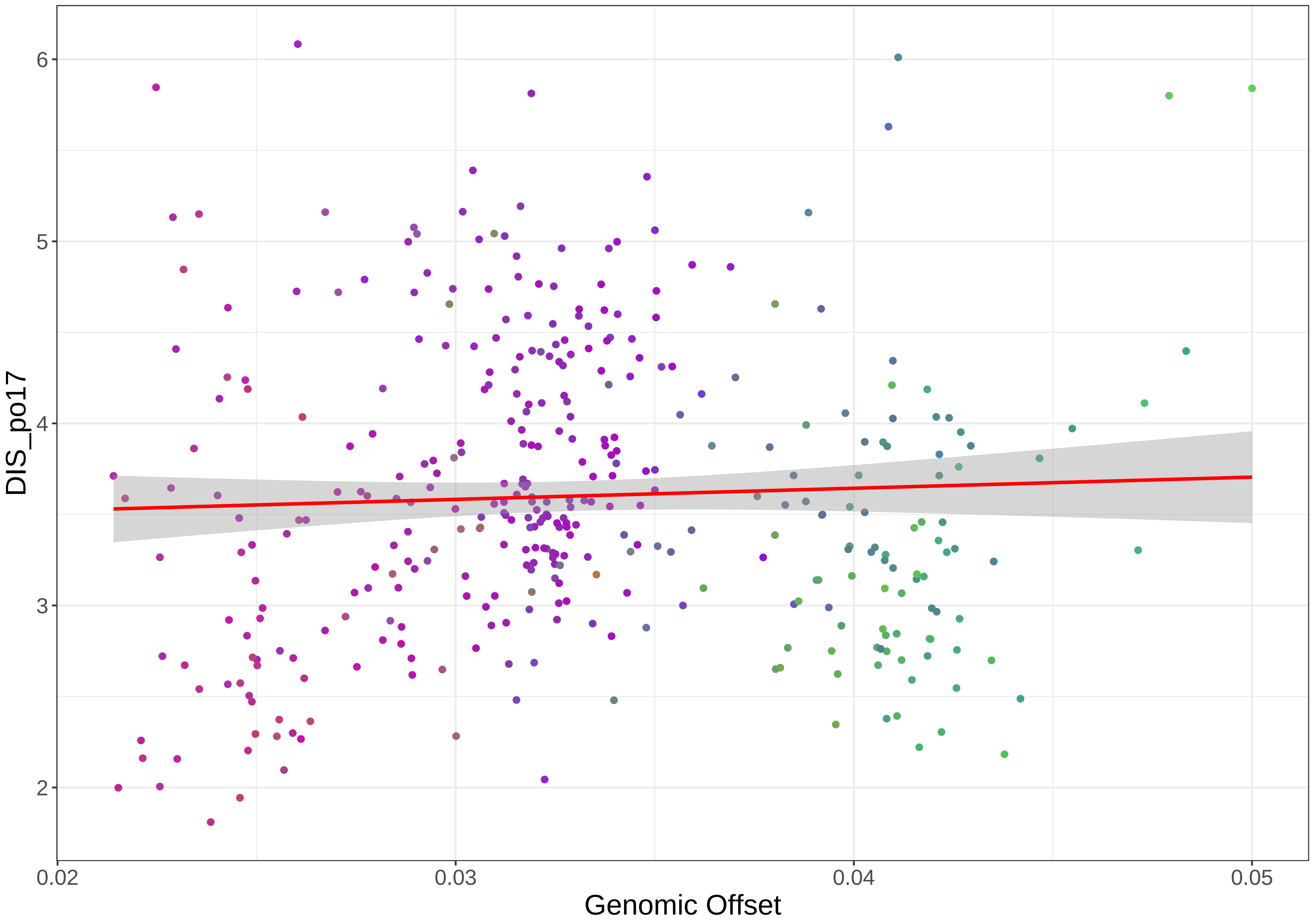

6
5
4
3
2
DIS_po17
0.02
0.03
0.04
0.05
Genomic Offset

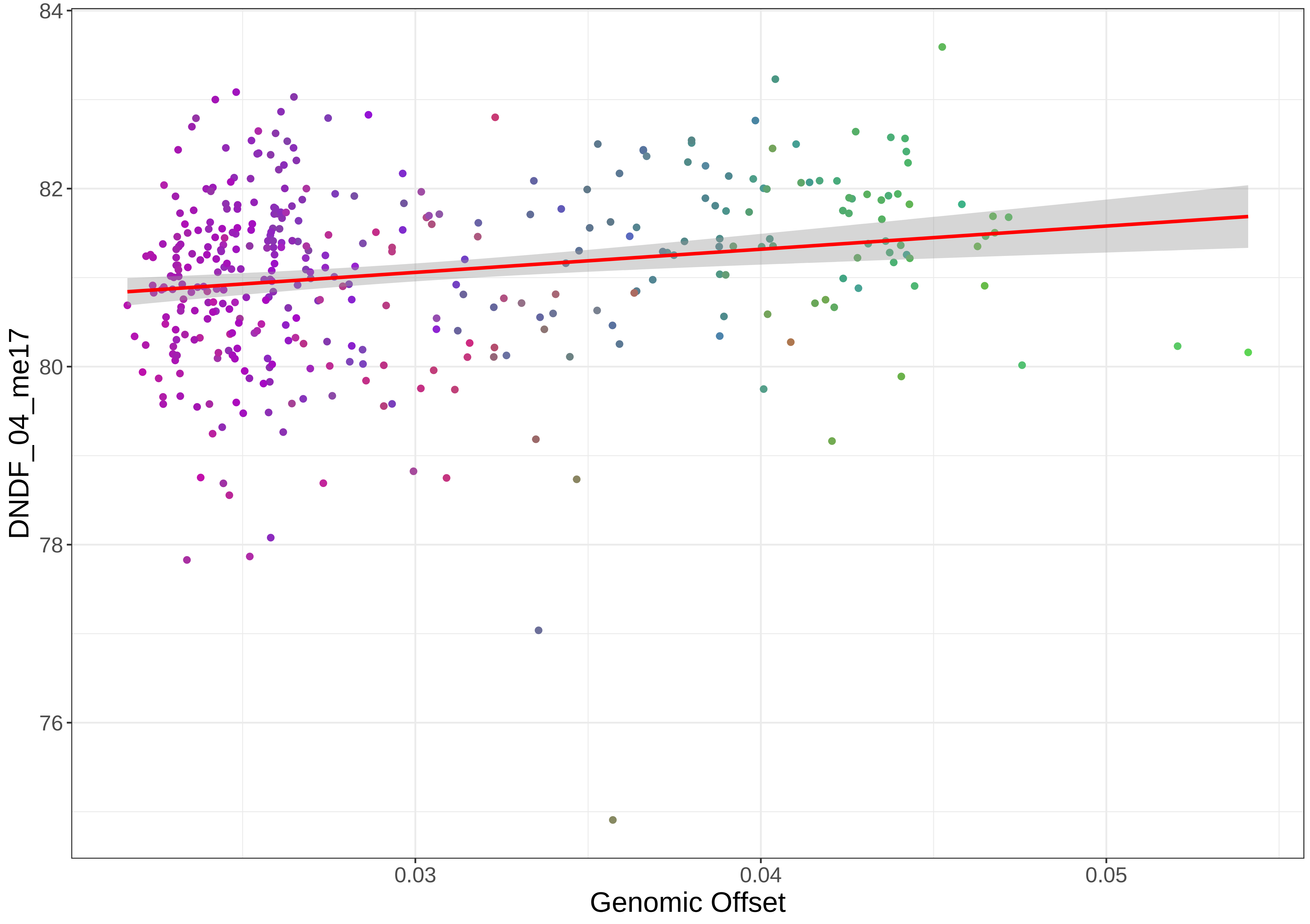

84
82
80
78
76
DNDF_04_me17
0.03
0.04
0.05
Genomic Offset

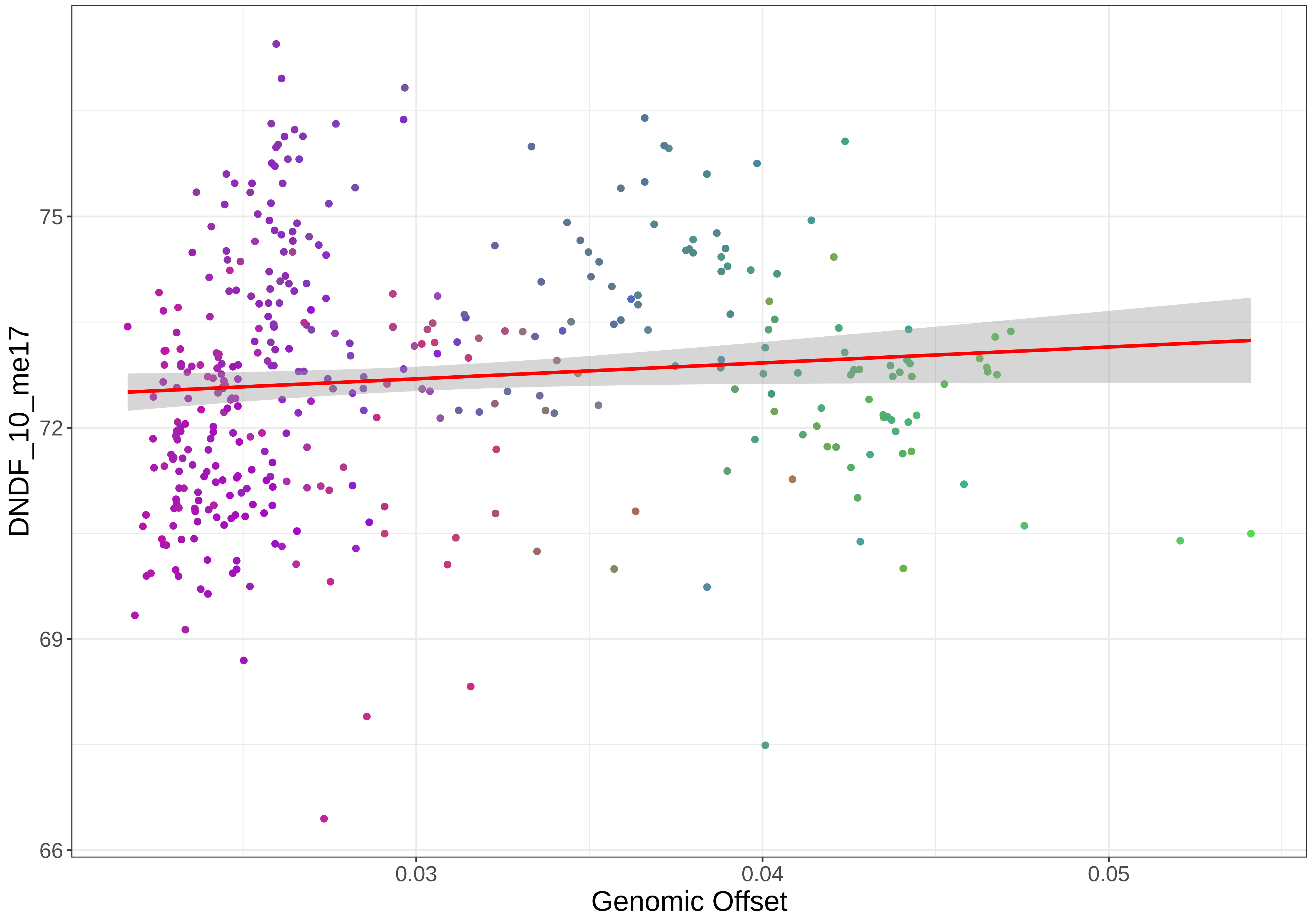

DNDF_10_me17
75
72
69
66
0.03
0.04
0.05
Genomic Offset

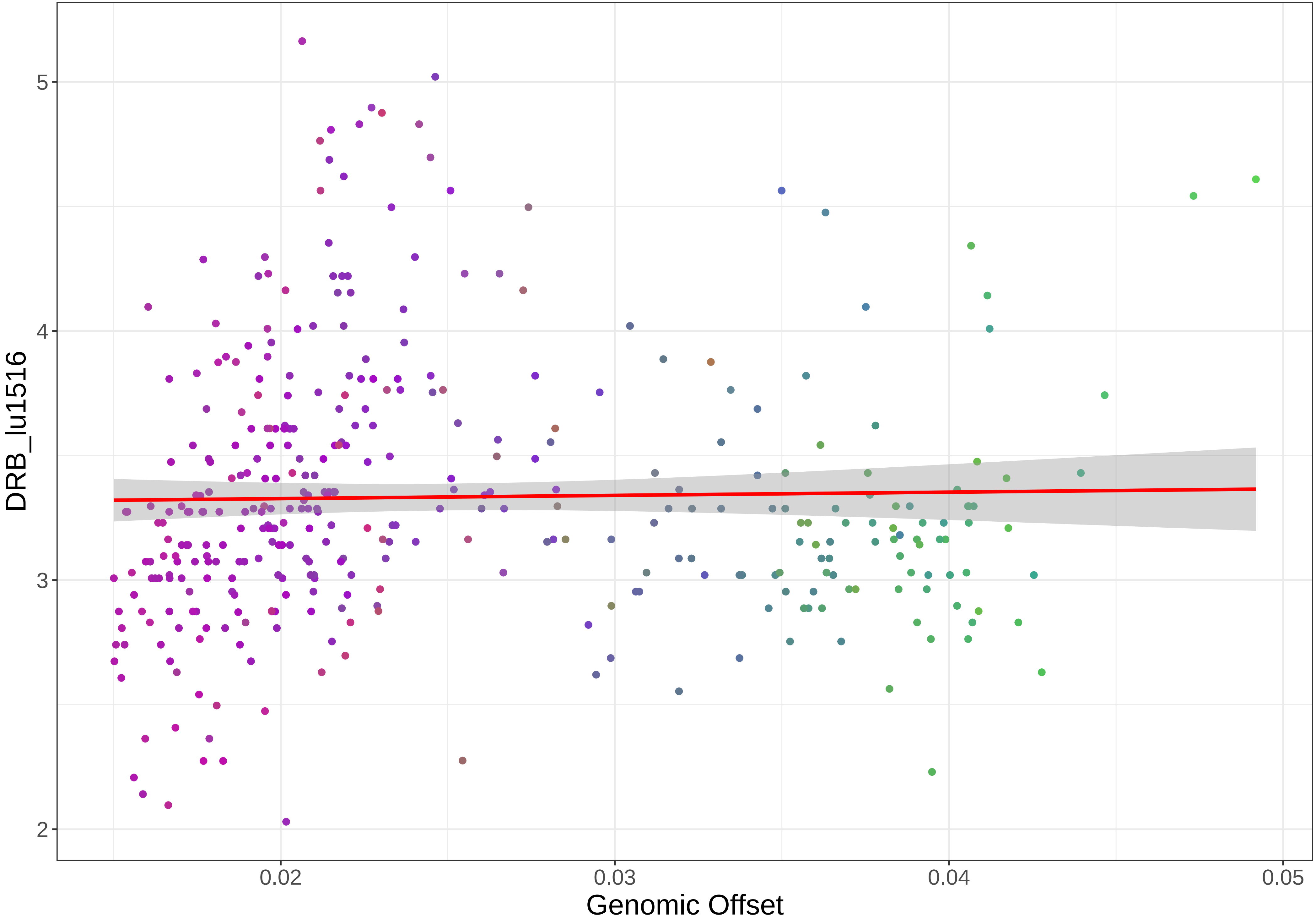

DRB_lu1516
5
4
3
2
0.02
0.03
0.04
0.05
Genomic Offset

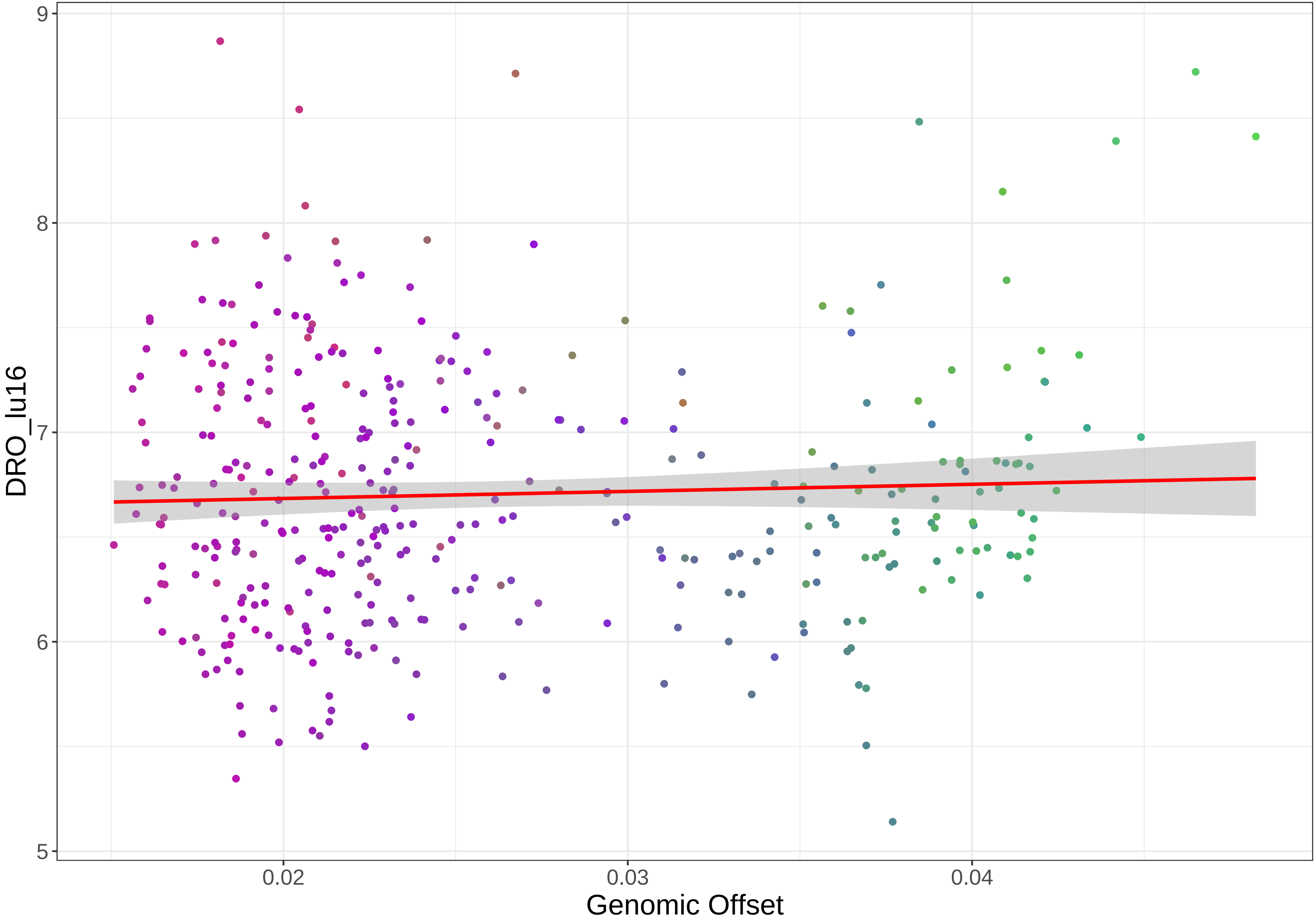

DRO_lu16
9
8
7
6
5
0.02
0.03
0.04
Genomic Offset

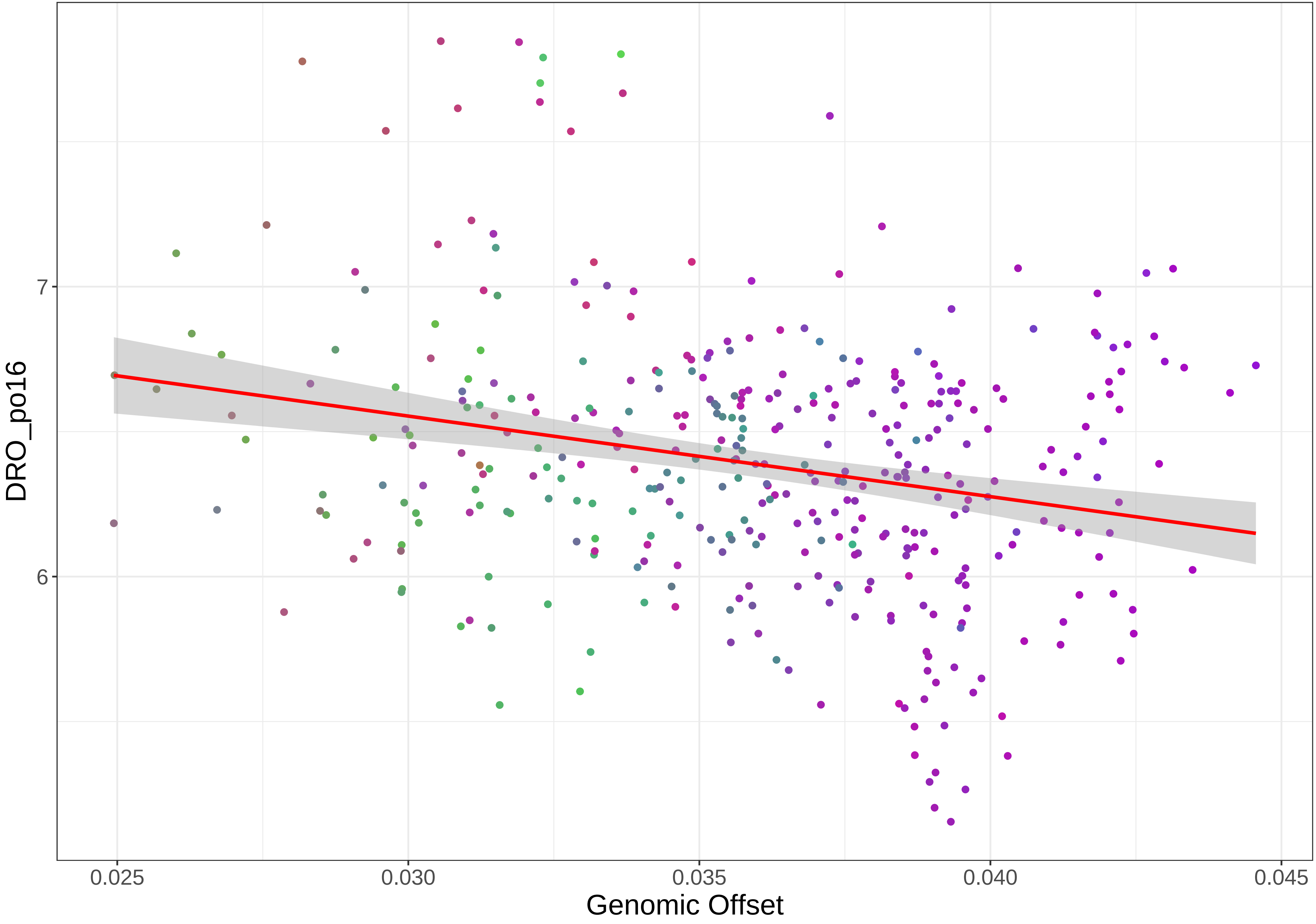

DRO_po16
7
6
0.025
0.030
0.035
0.040
0.045
Genomic Offset

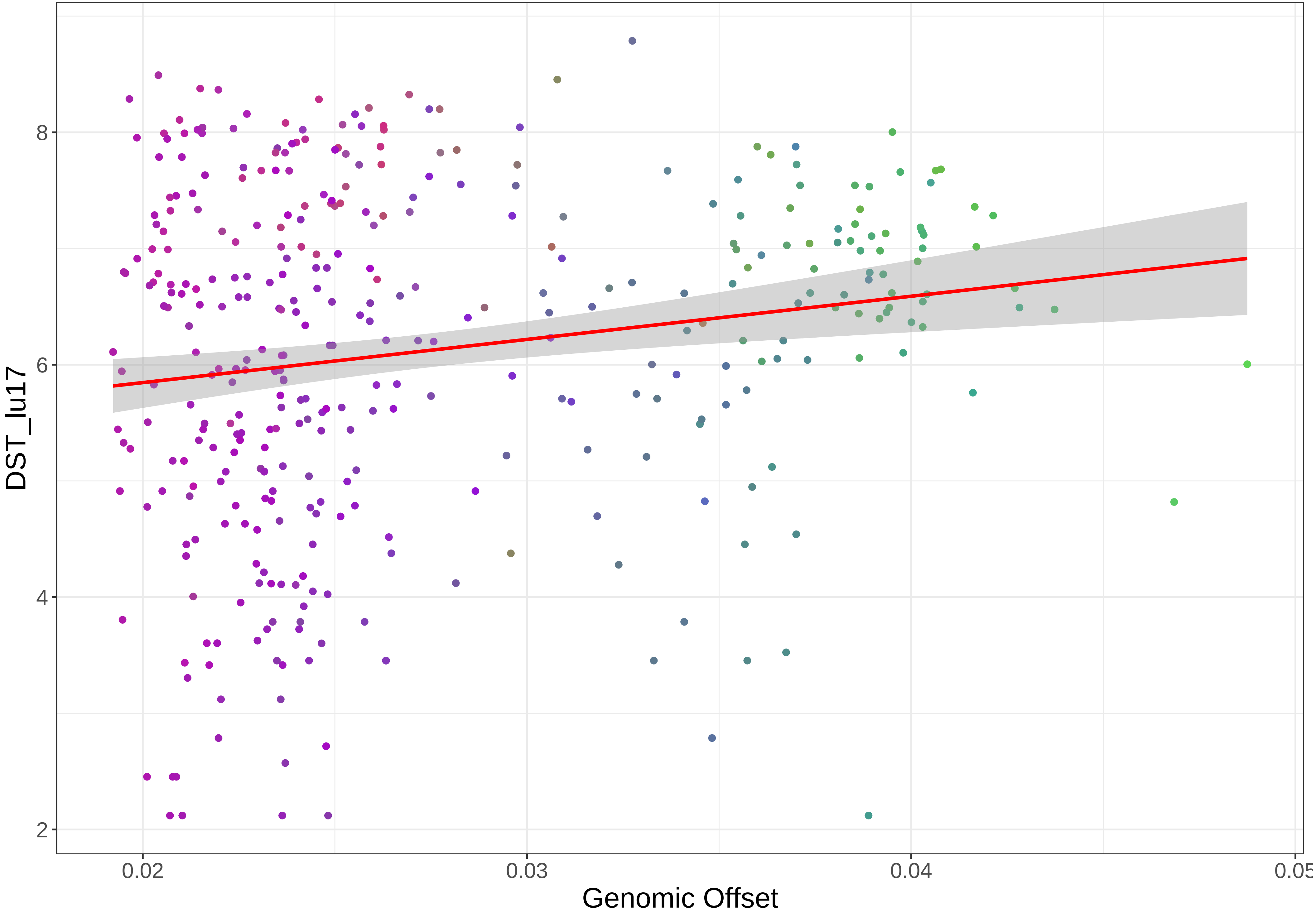
DST_lu17
8
6
4
2
0.02
0.03
0.04
0.05
Genomic Offset

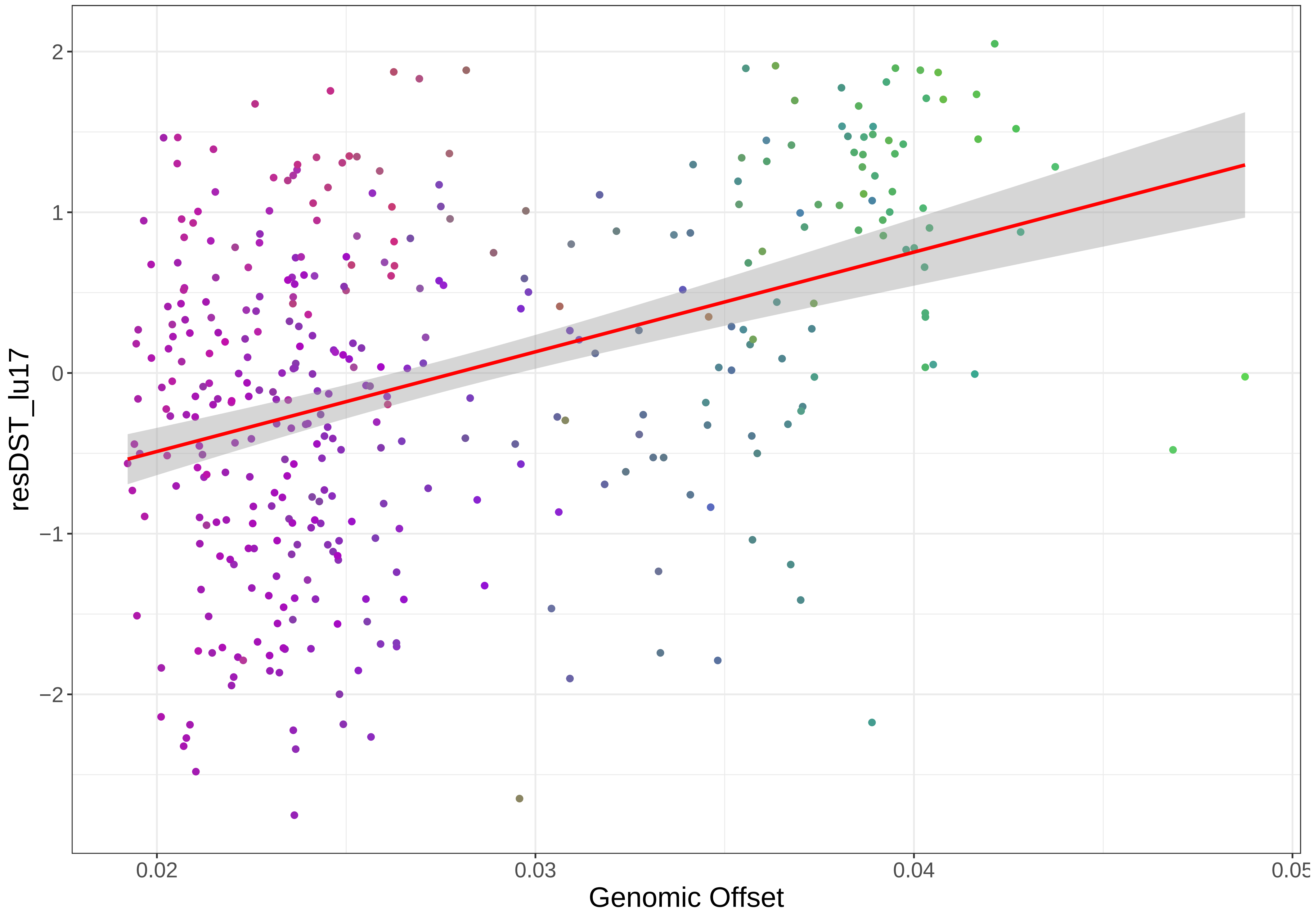

resDST_lu17
Genomic Offset
2
1
0
−1
−2
0.02
0.03
0.04
0.05

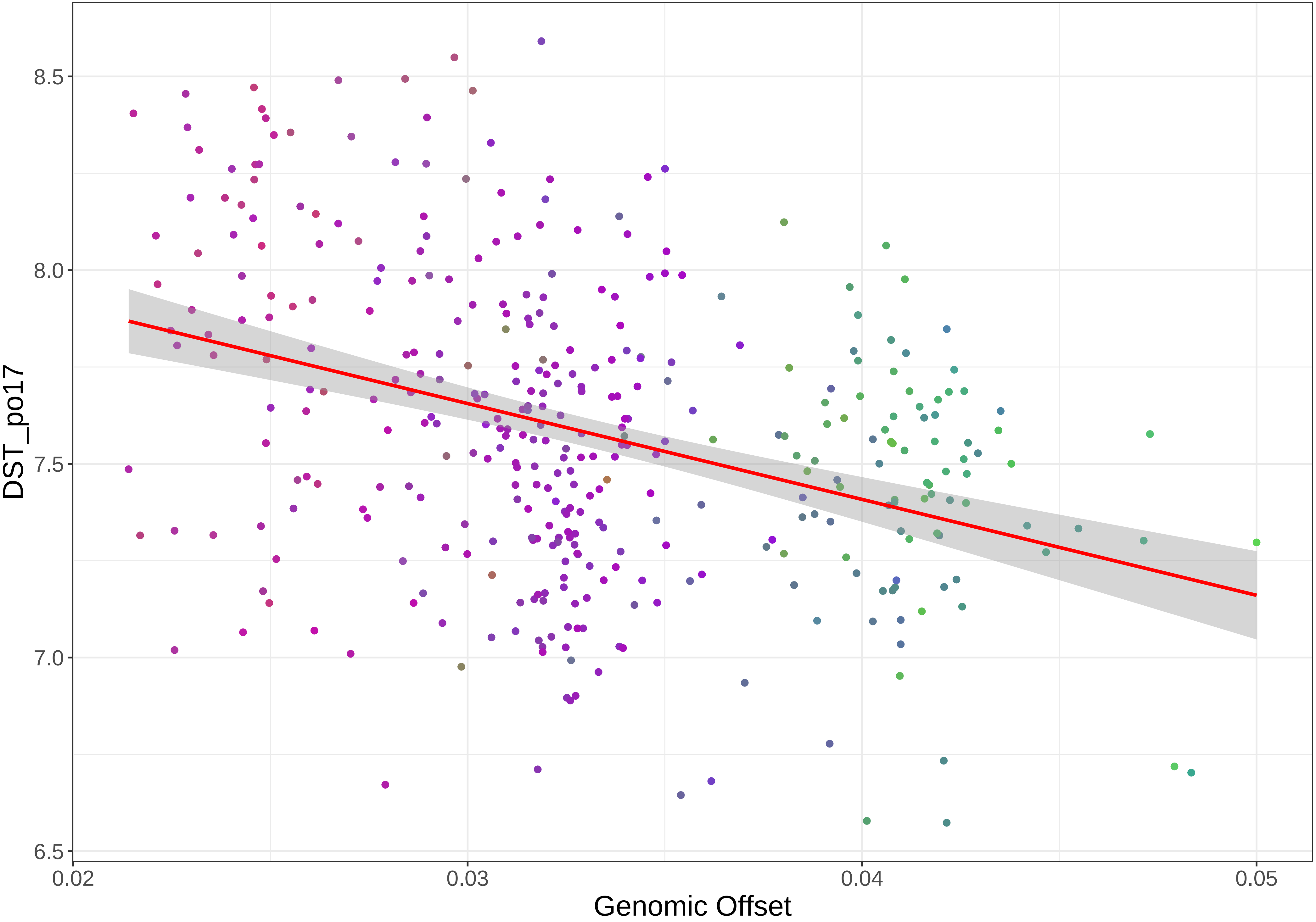

8.5
8.0
7.5
7.0
6.5
DST_po17
0.02
0.03
0.04
0.05
Genomic Offset

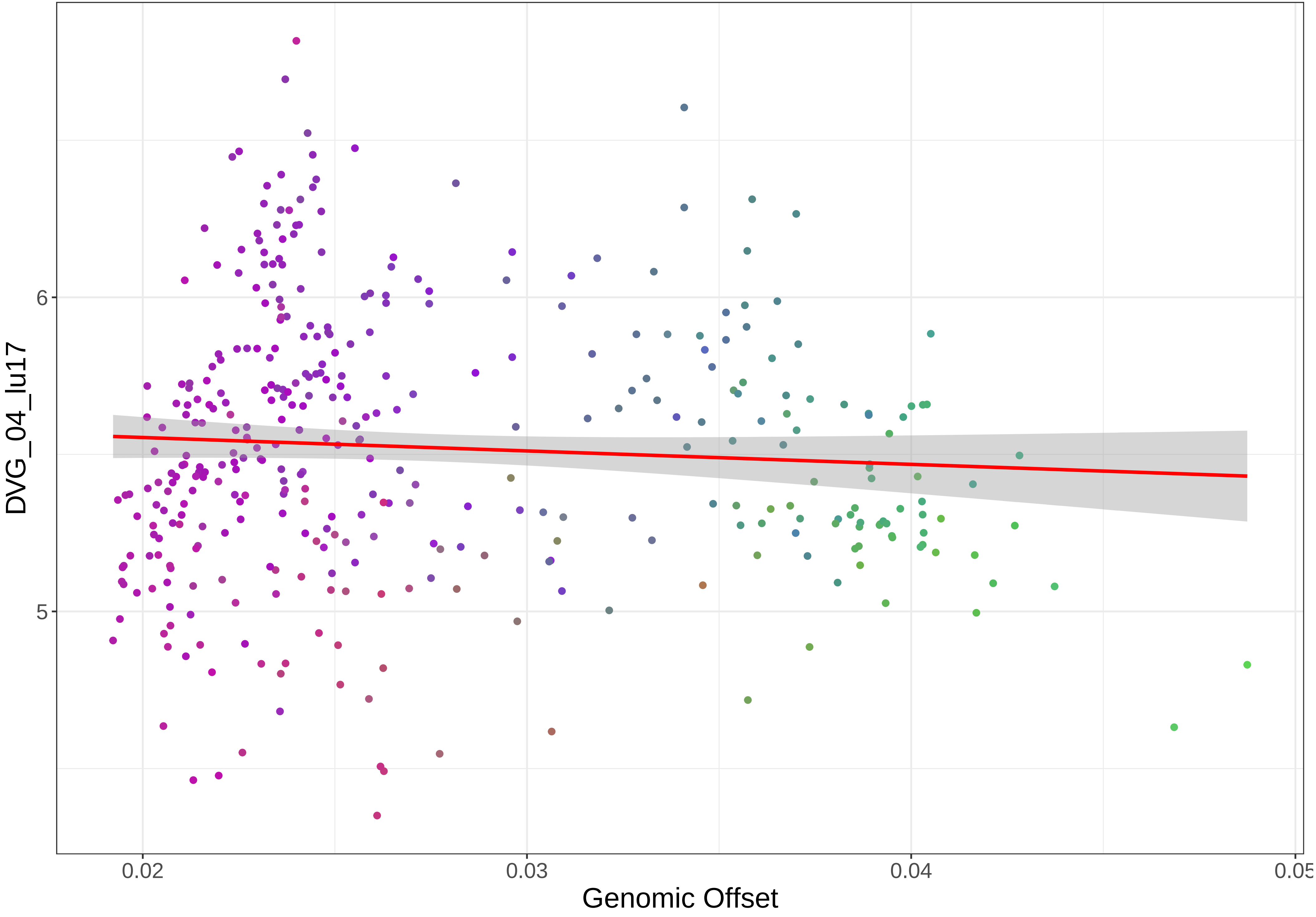

DVG_04_lu17
6
5
0.02
0.03
0.04
0.05
Genomic Offset

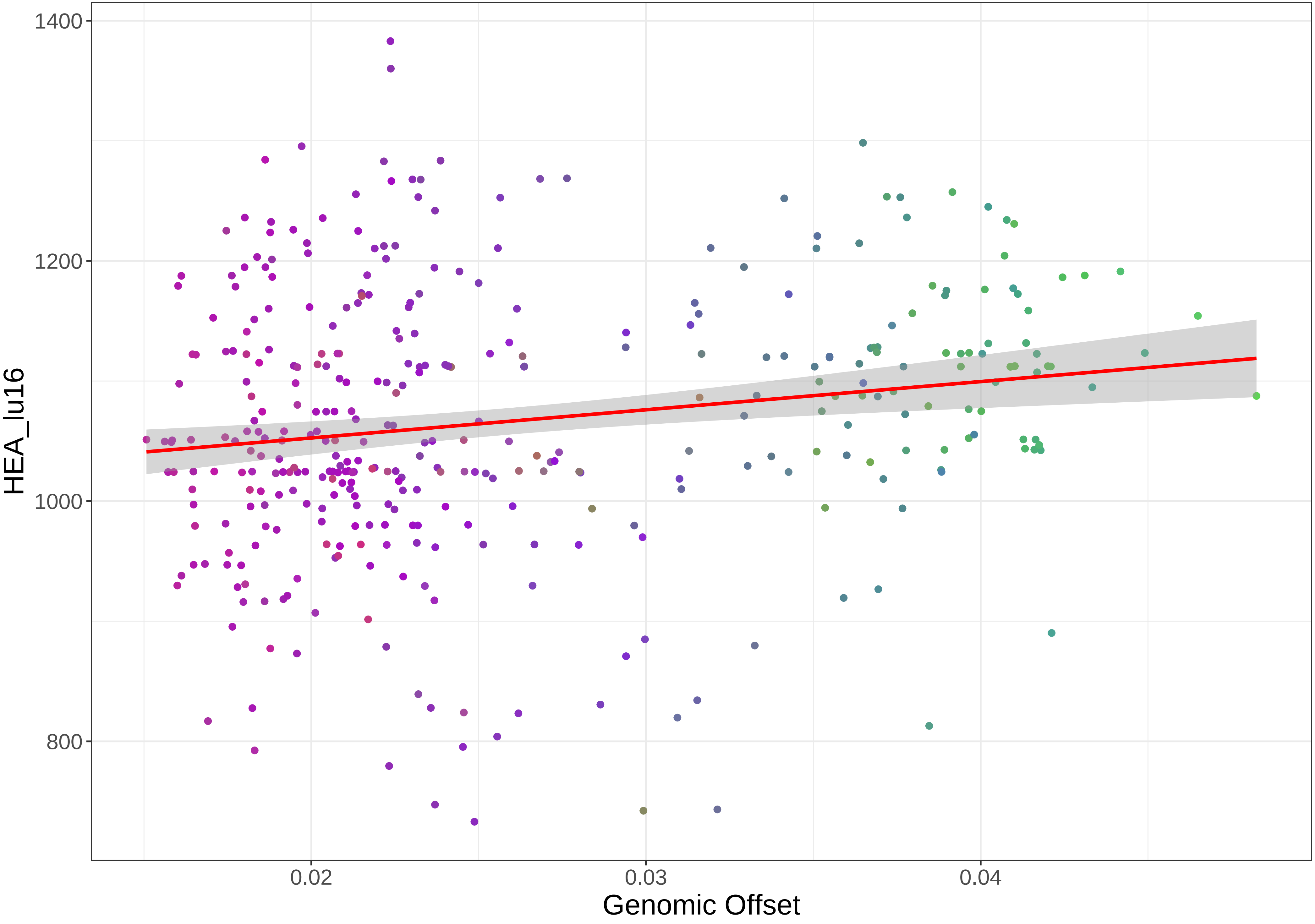

1400
1200
1000
800
HEA_lu16
0.02
0.03
0.04
Genomic Offset

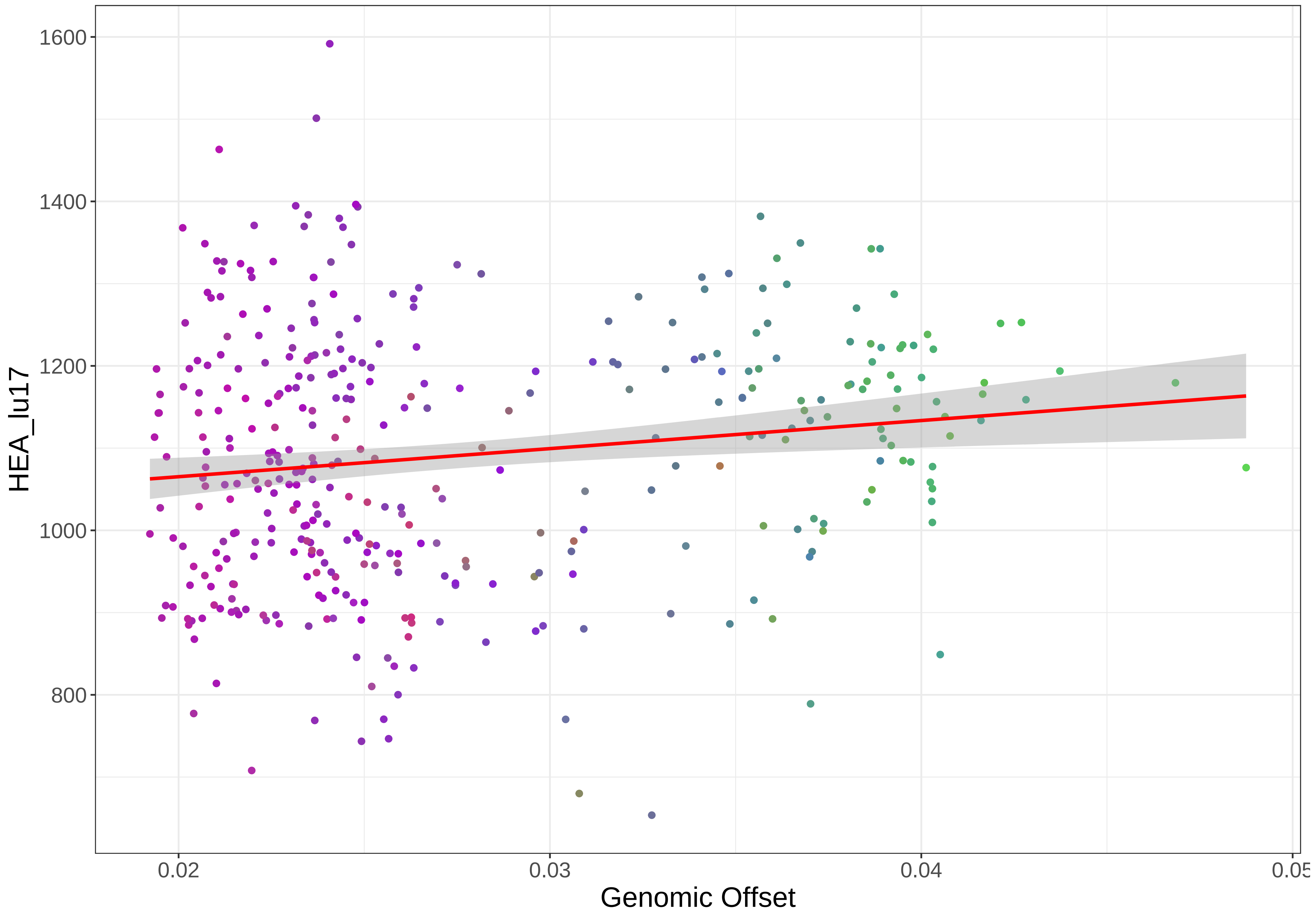

1600
1400
1200
1000
800
HEA_lu17
0.02
0.03
0.04
0.05
Genomic Offset

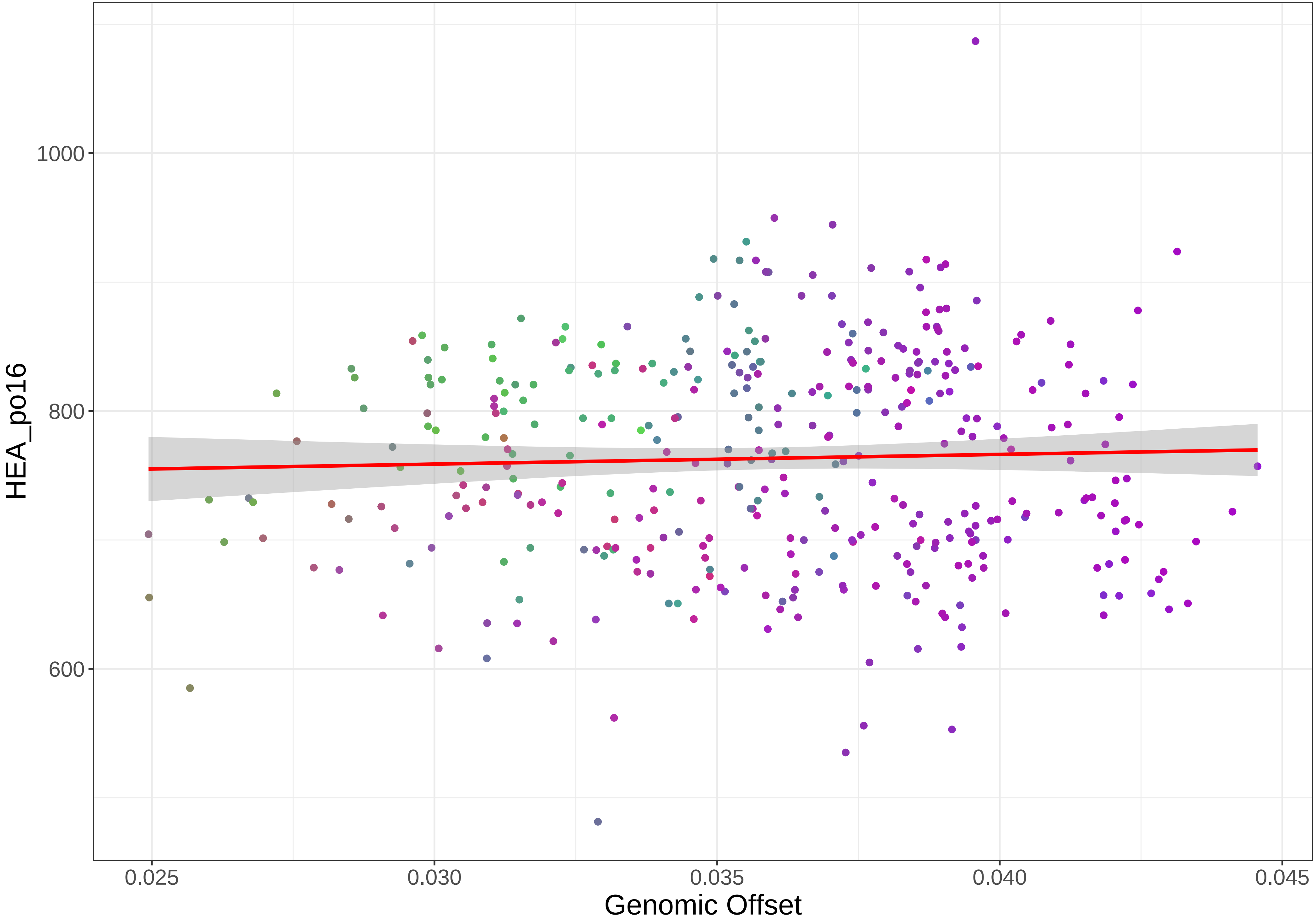
1000
800
600
HEA_po16
0.025
0.030
0.035
0.040
0.045
Genomic Offset

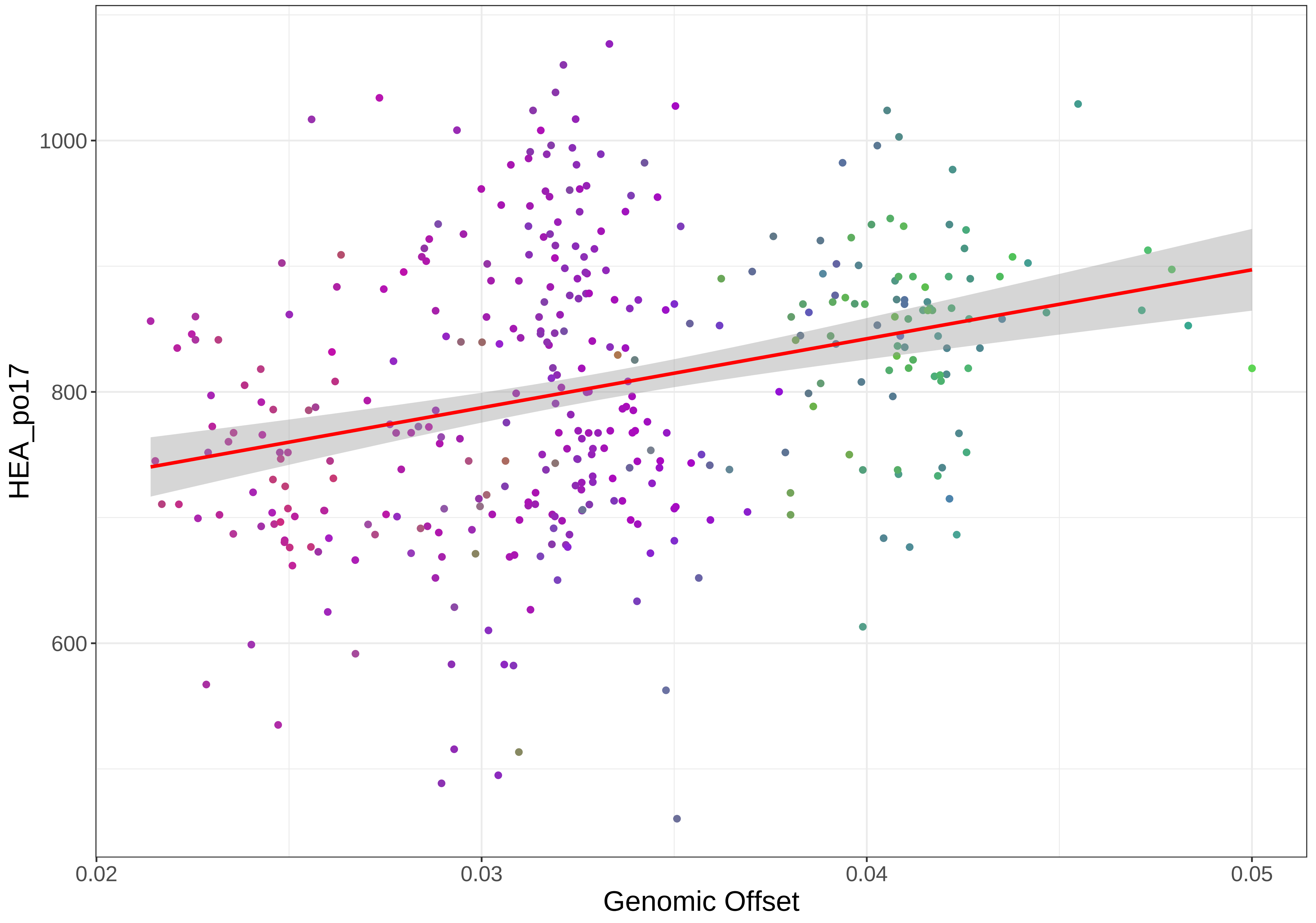
HEA_po17
600
800
1000
0.02
0.03
0.04
0.05
Genomic Offset

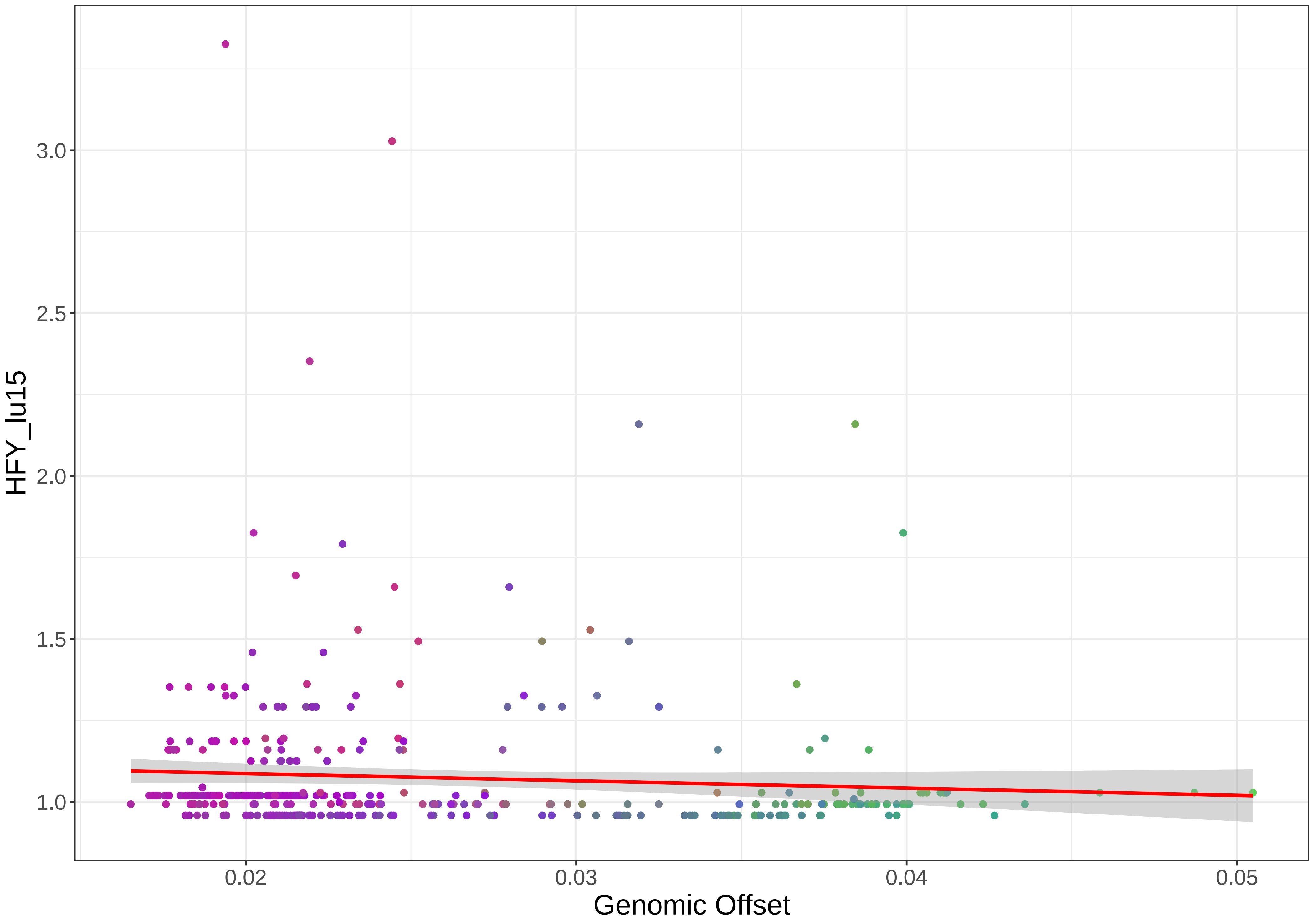
HFY_lu15
3.0
2.5
2.0
1.5
1.0
0.02
0.03
0.04
0.05
Genomic Offset

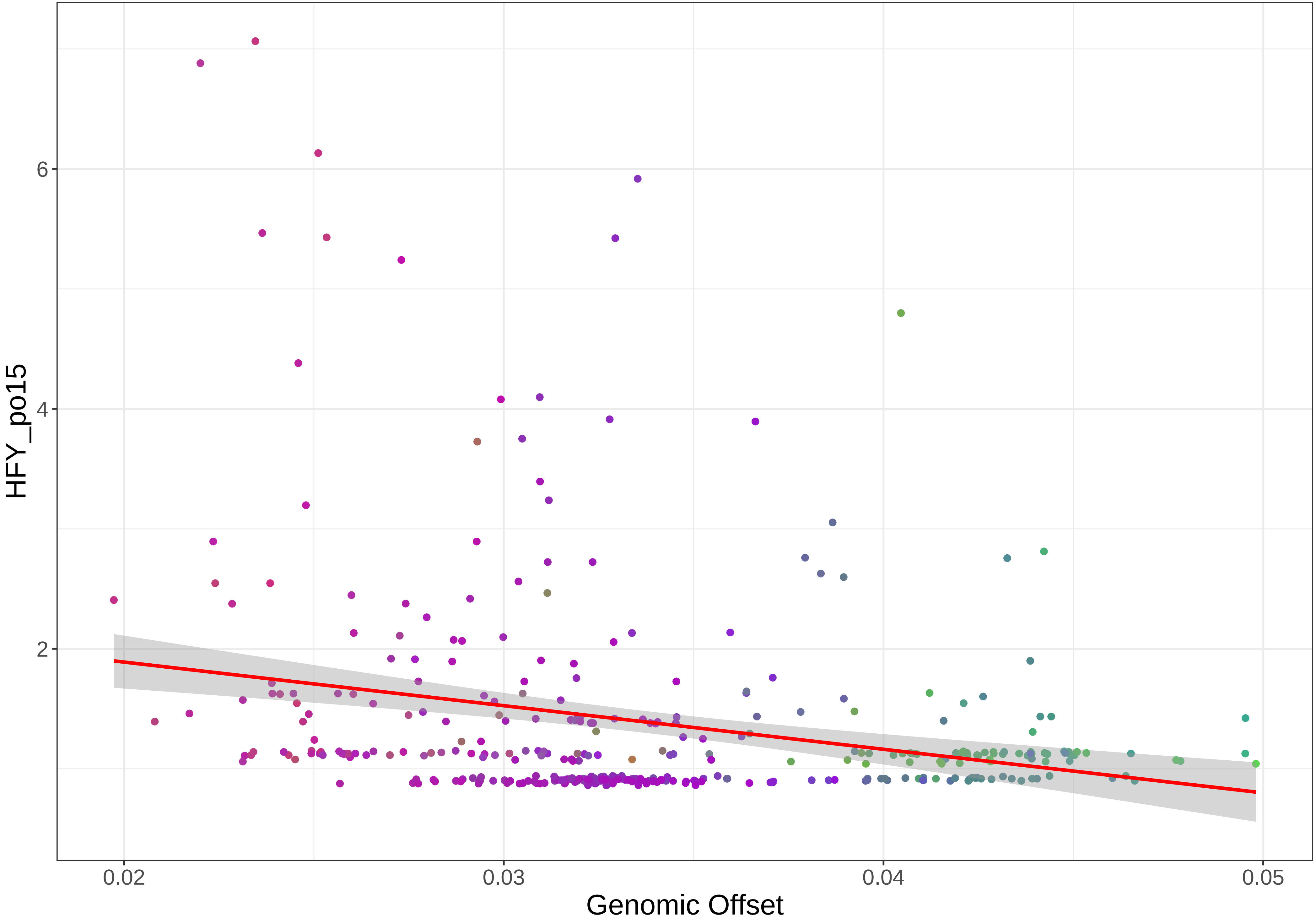

HFY_po15
6
4
2
0.02
0.03
0.04
0.05
Genomic Offset

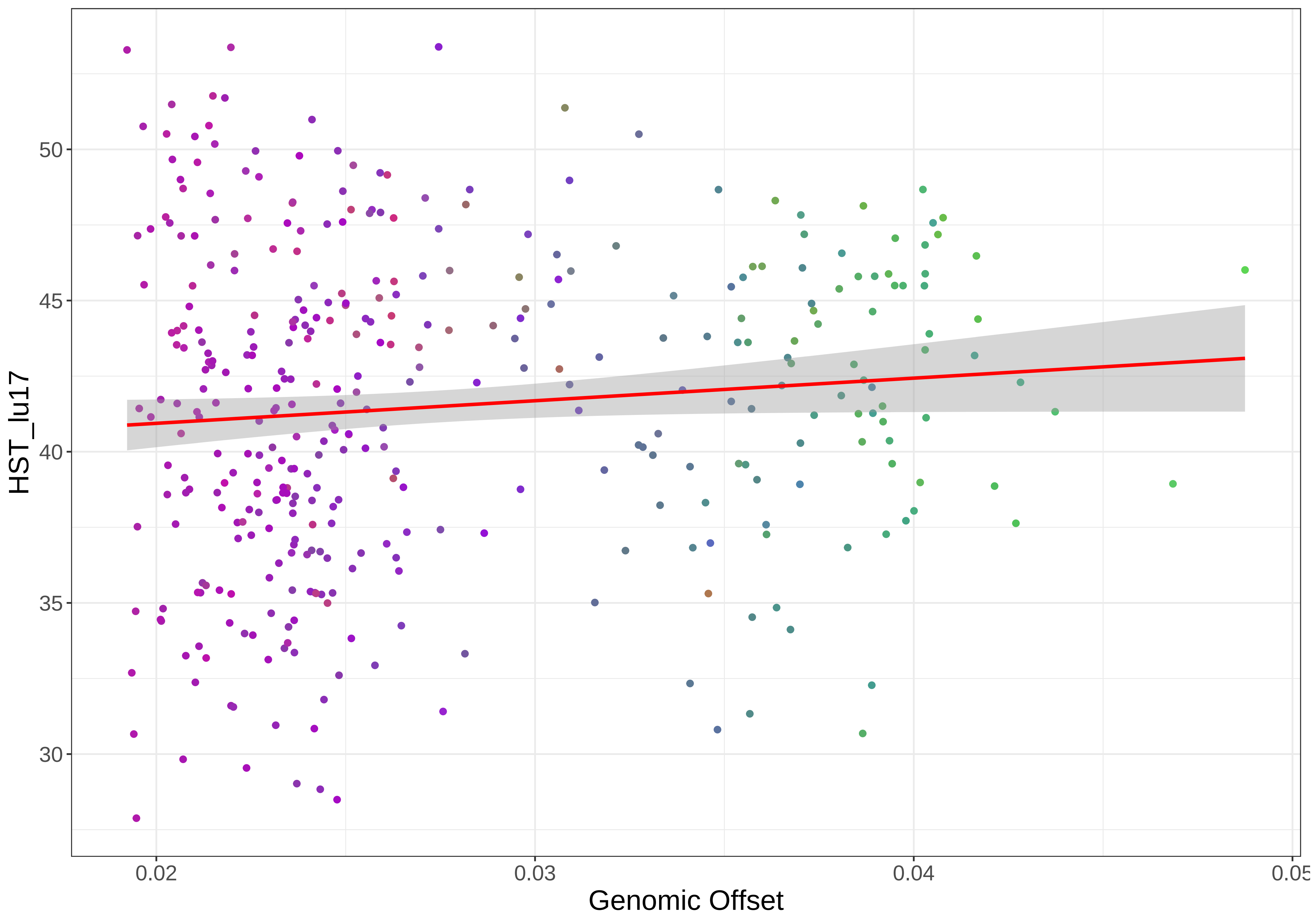

HST_lu17
50
45
40
35
30
0.02
0.03
0.04
0.05
Genomic Offset

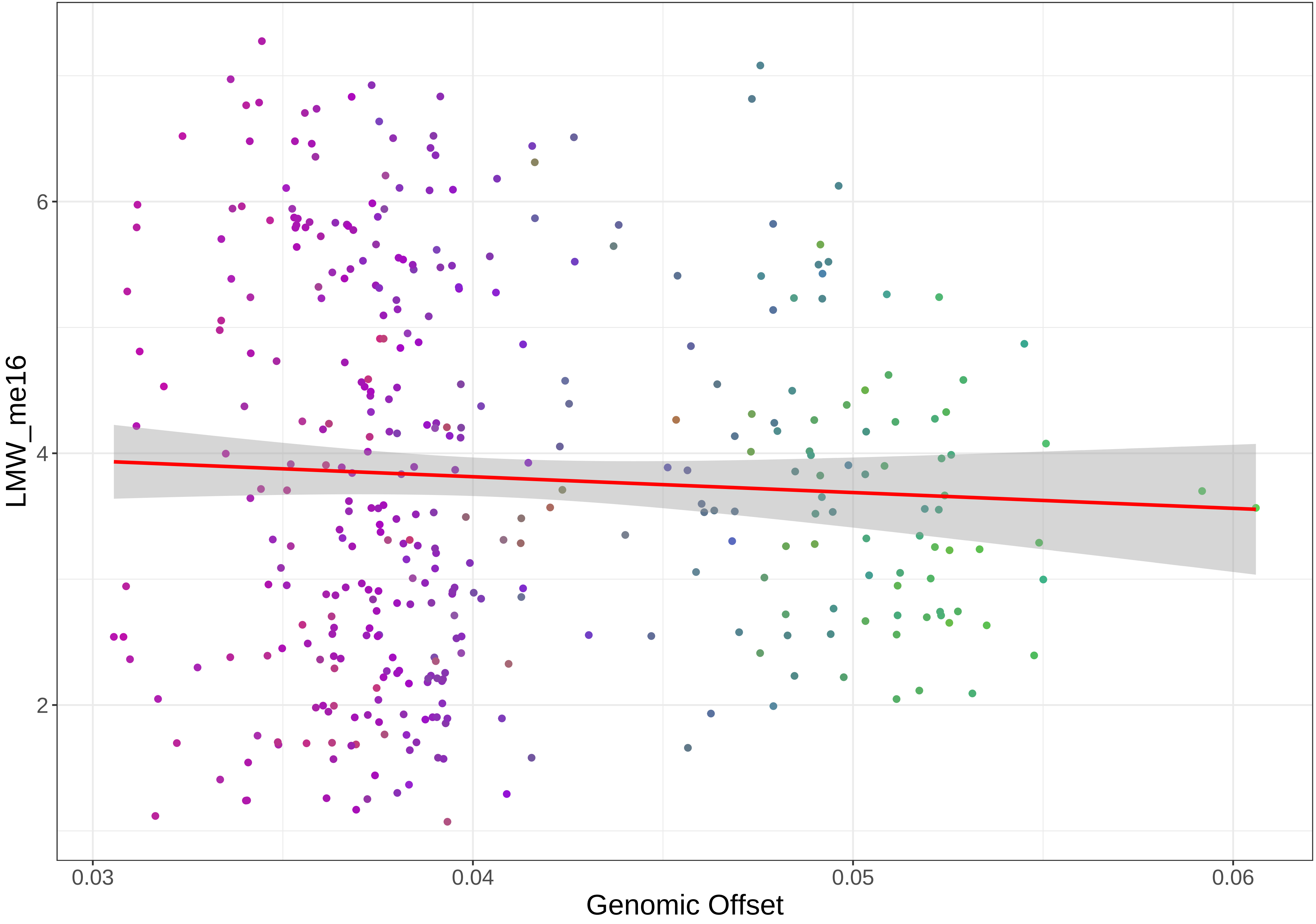

LMW_me16
6
4
2
0.03
0.04
0.05
0.06
Genomic Offset

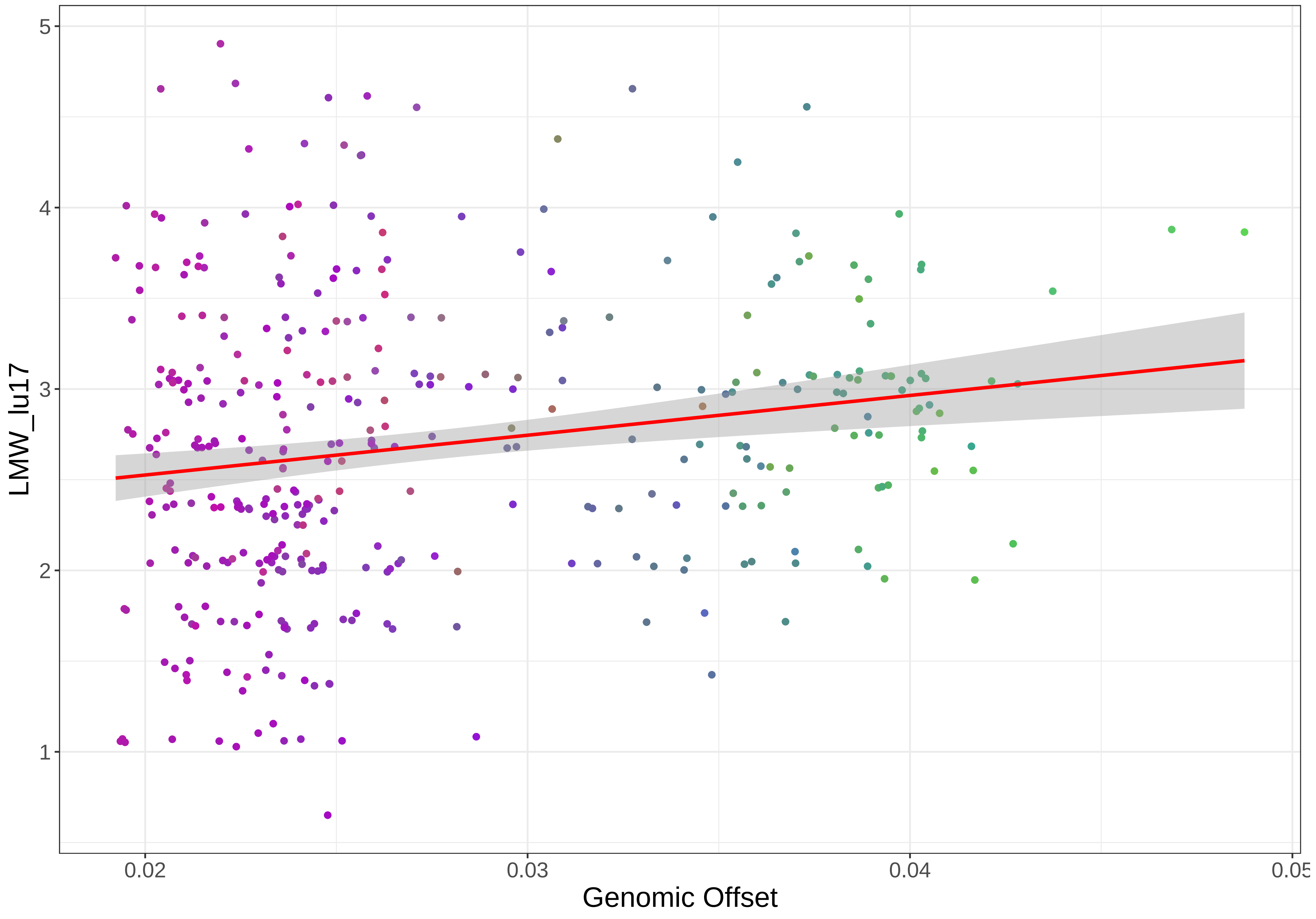

5
4
3
2
1
LMW_lu17
0.02
0.03
0.04
0.05
Genomic Offset

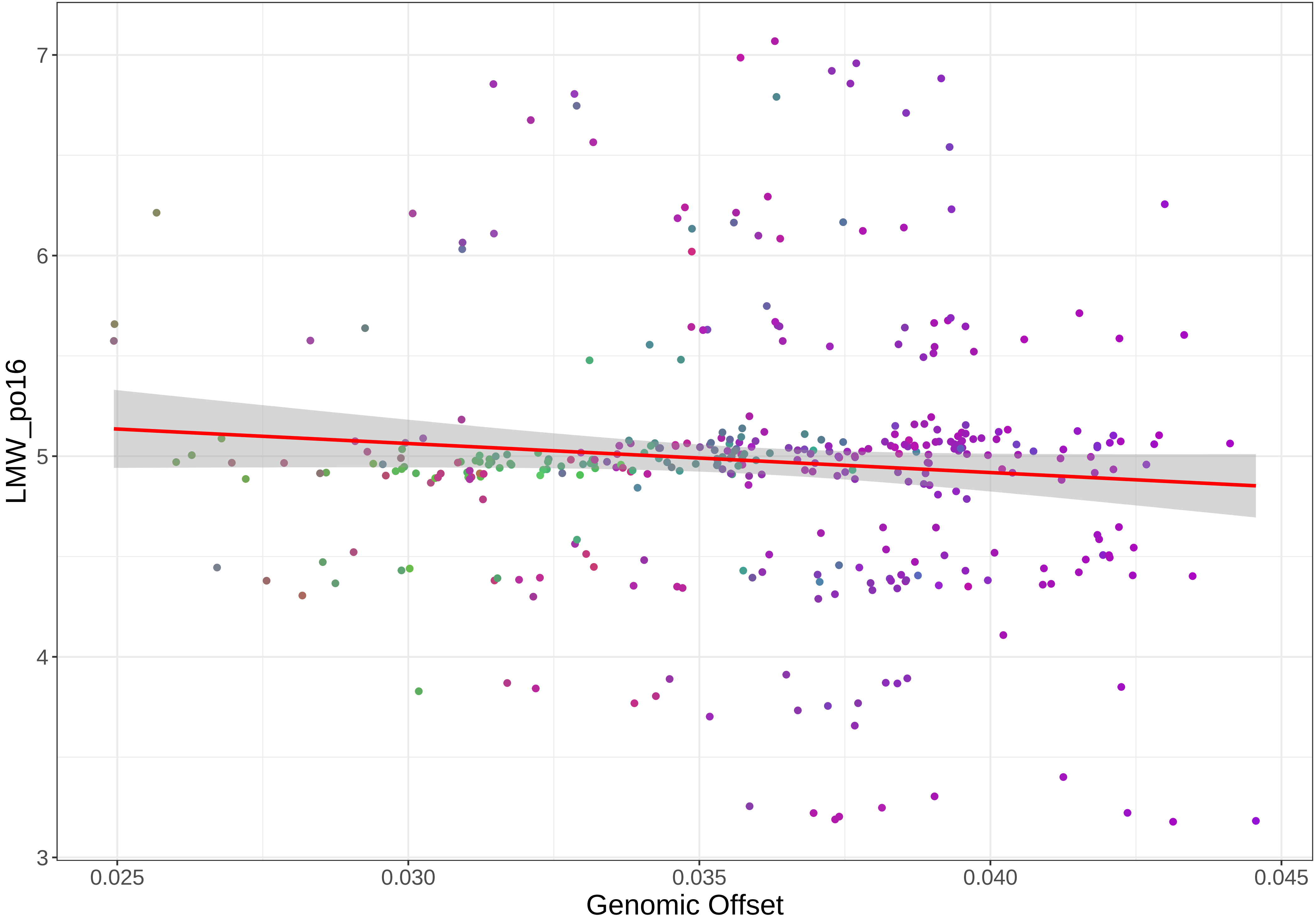

7
6
5
4
3
LMW_po16
0.025
0.030
0.035
0.040
0.045
Genomic Offset

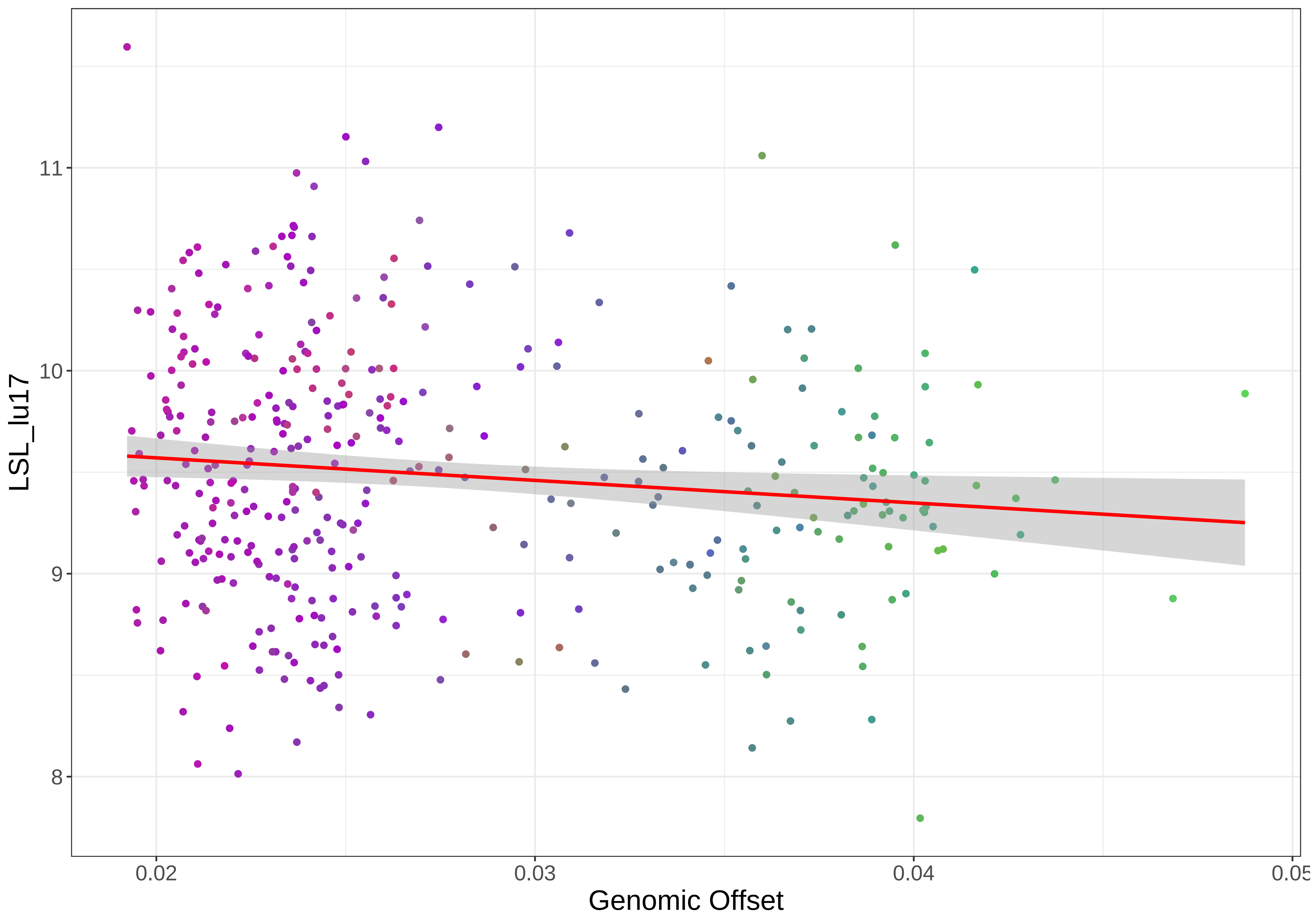

11
10
9
8
LSL_lu17
0.02
0.03
0.04
0.05
Genomic Offset

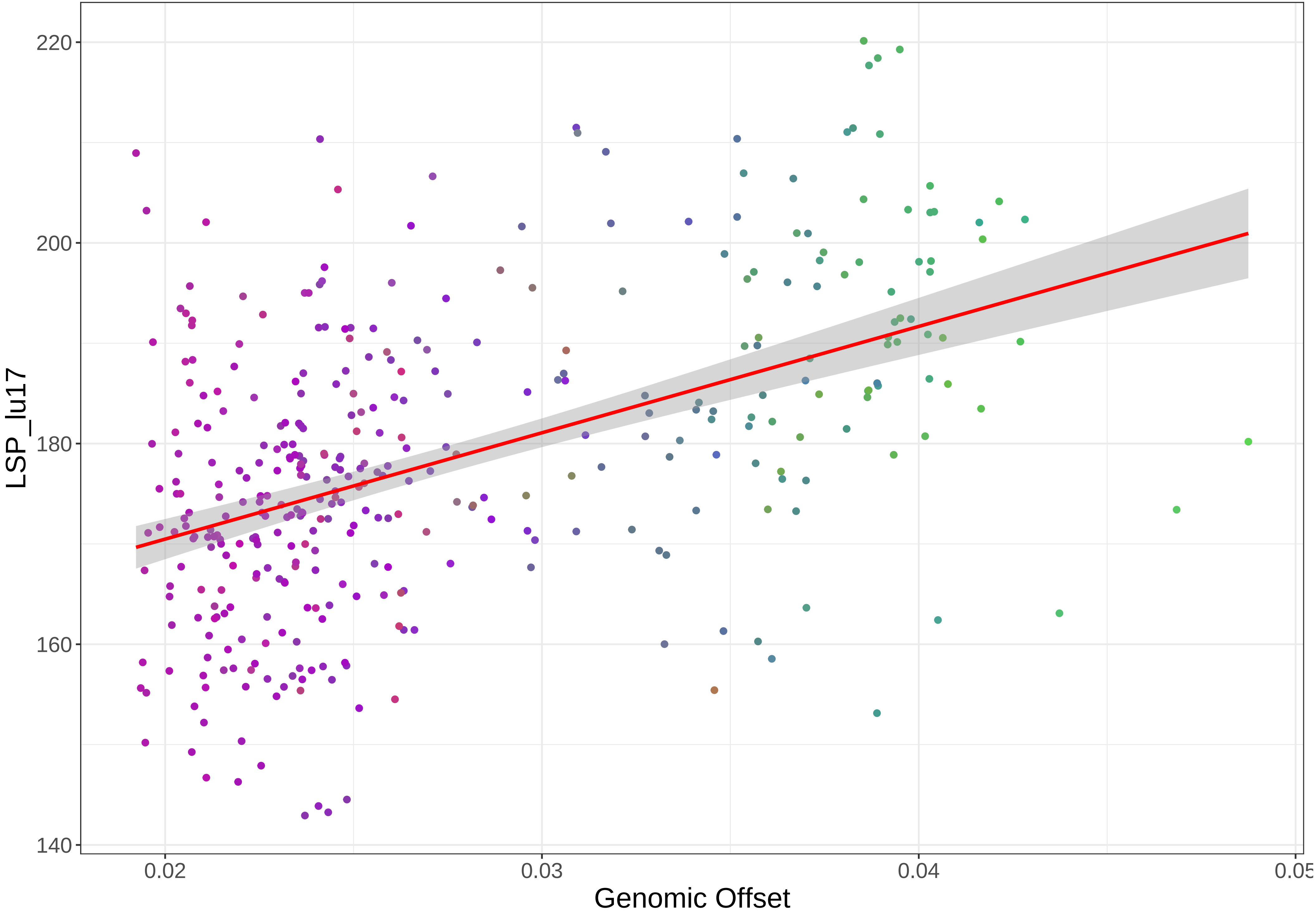

220
200
180
160
140
LSP_lu17
0.02
0.03
0.04
0.05
Genomic Offset

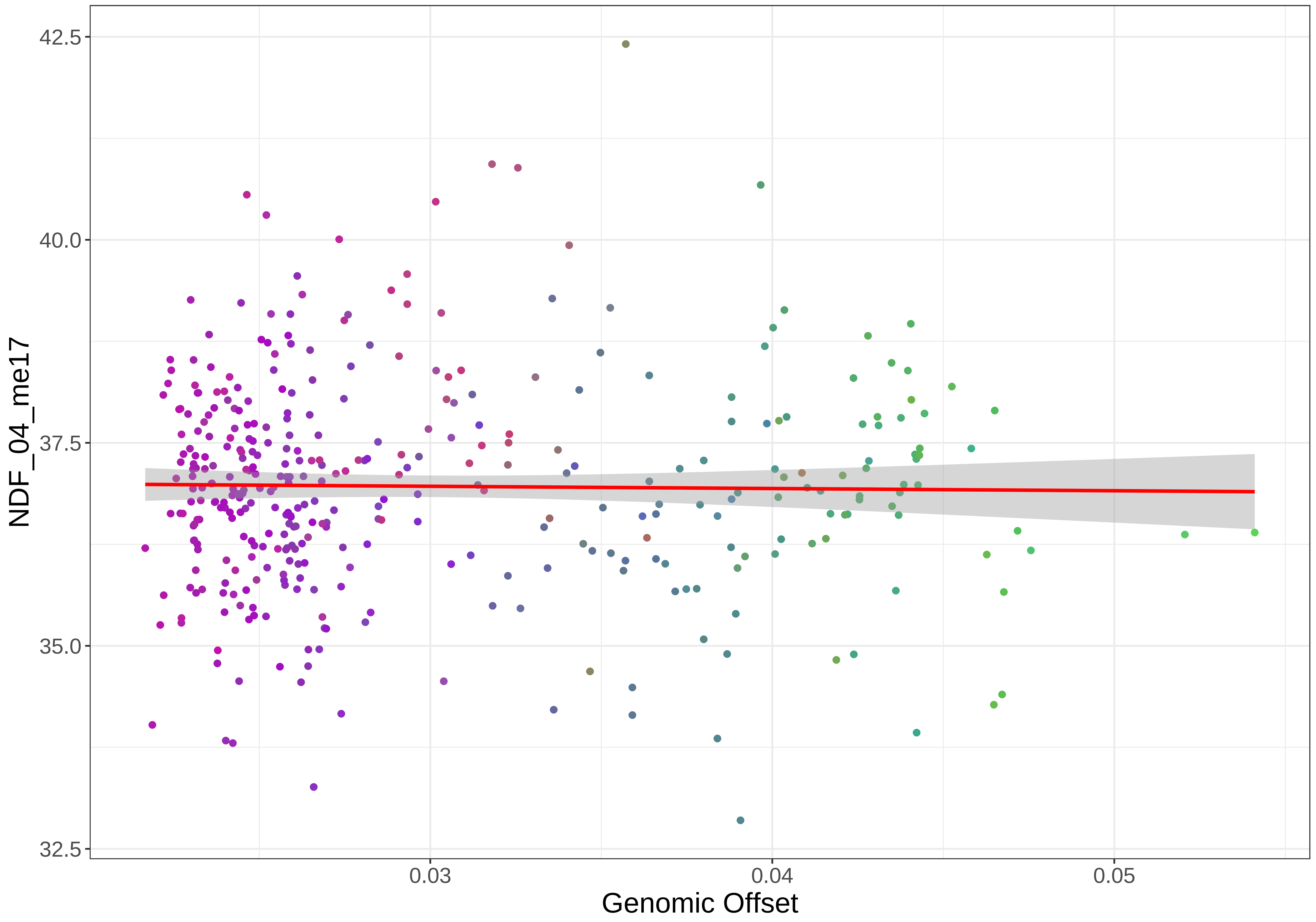
42.5
40.0
37.5
35.0
32.5
NDF_04_me17
0.03
0.04
0.05
Genomic Offset

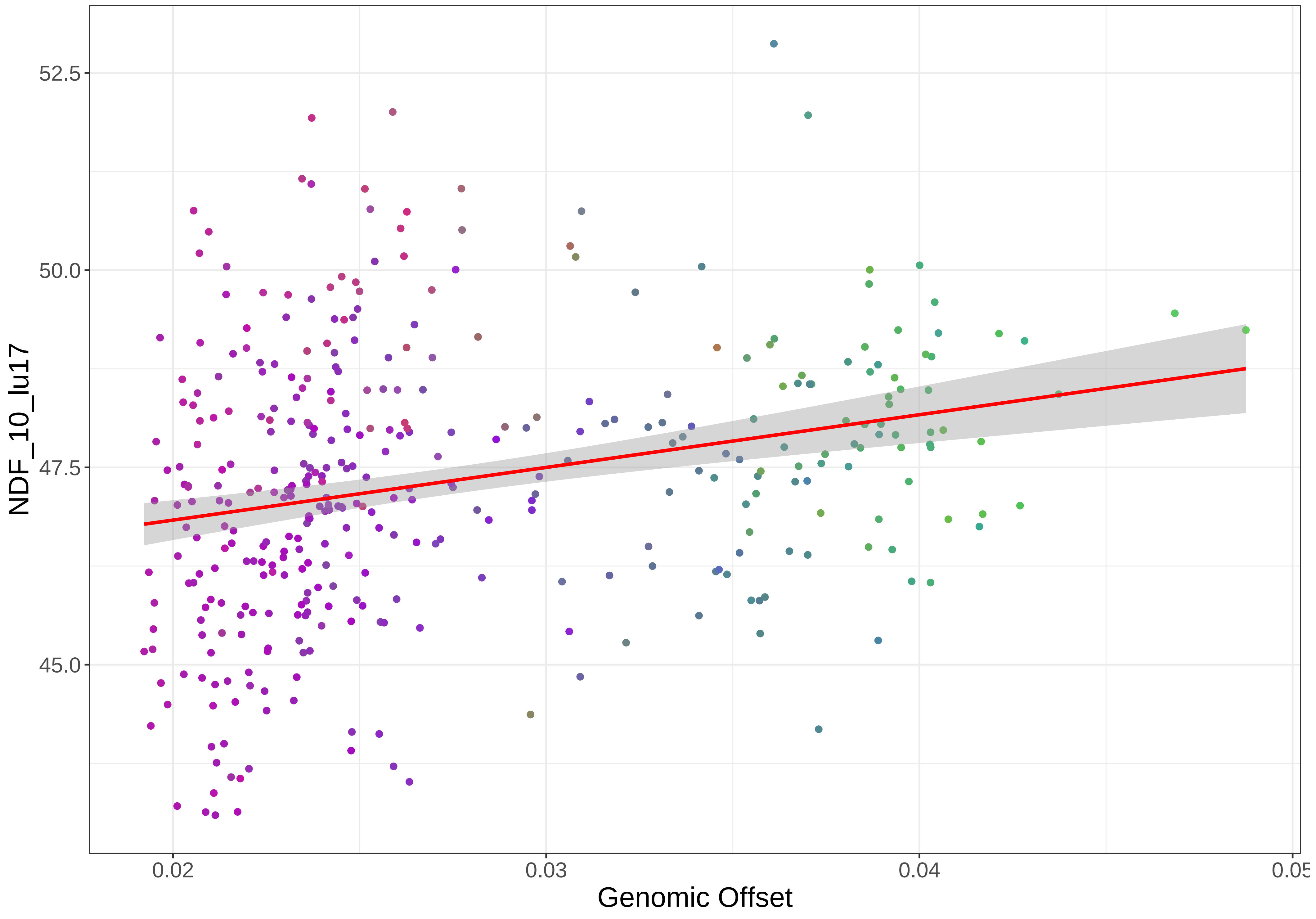

NDF_10_lu17
52.5
50.0
47.5
45.0
0.02
0.03
0.04
0.05
Genomic Offset

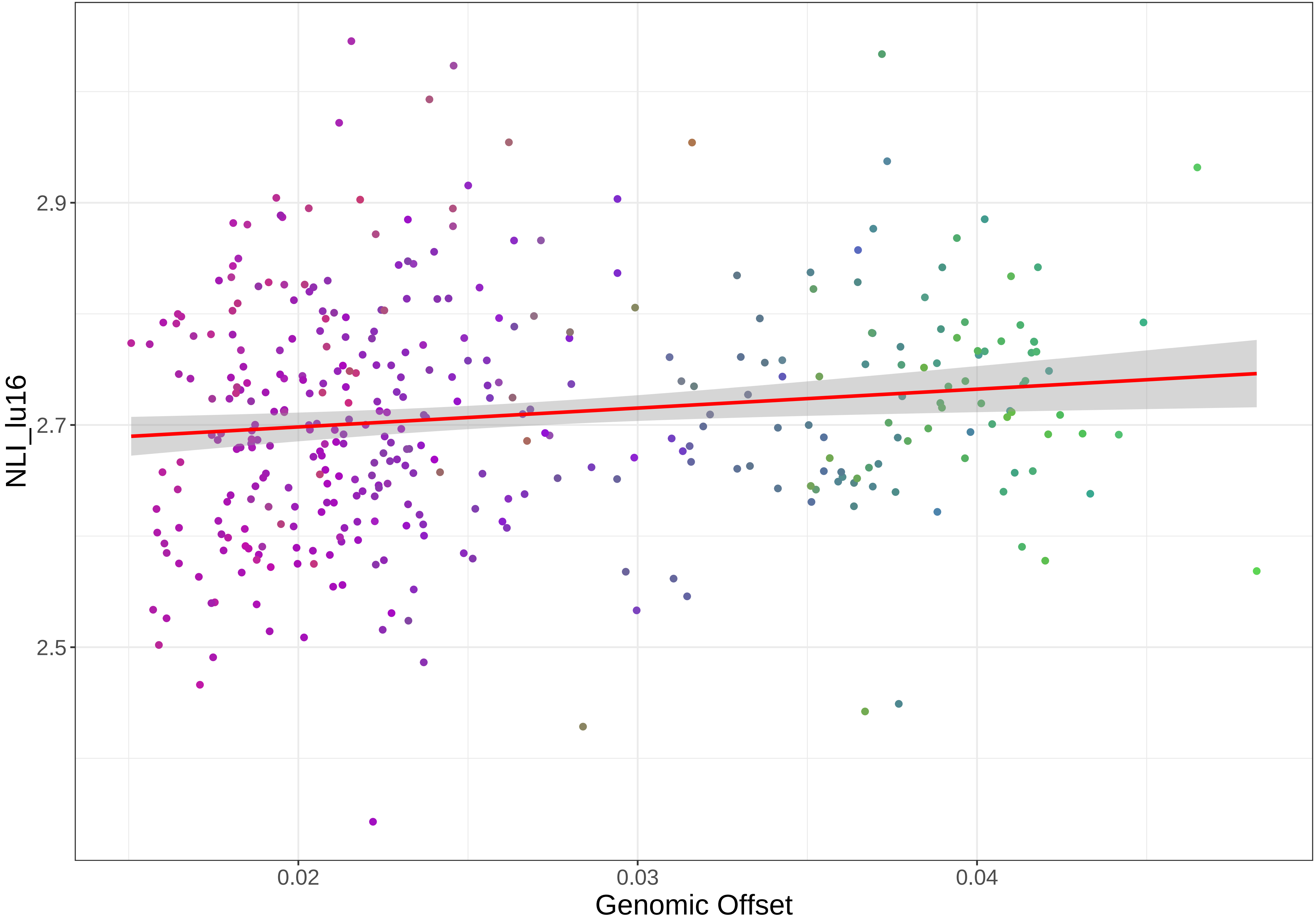

NLI_lu16
2.9
2.7
2.5
0.02
0.03
0.04
Genomic Offset

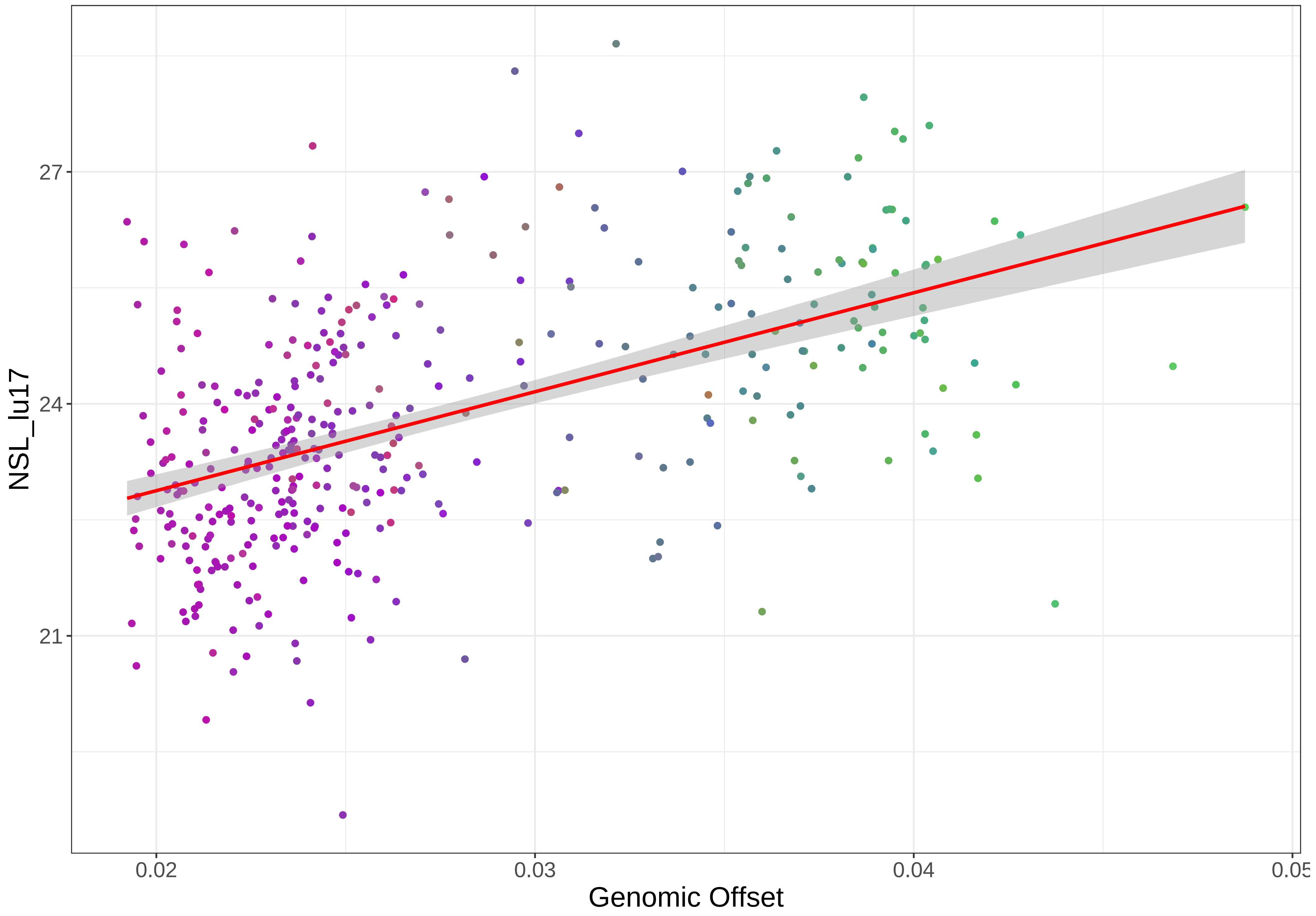

NSL_lu17
27
24
21
0.02
0.03
0.04
Genomic Offset

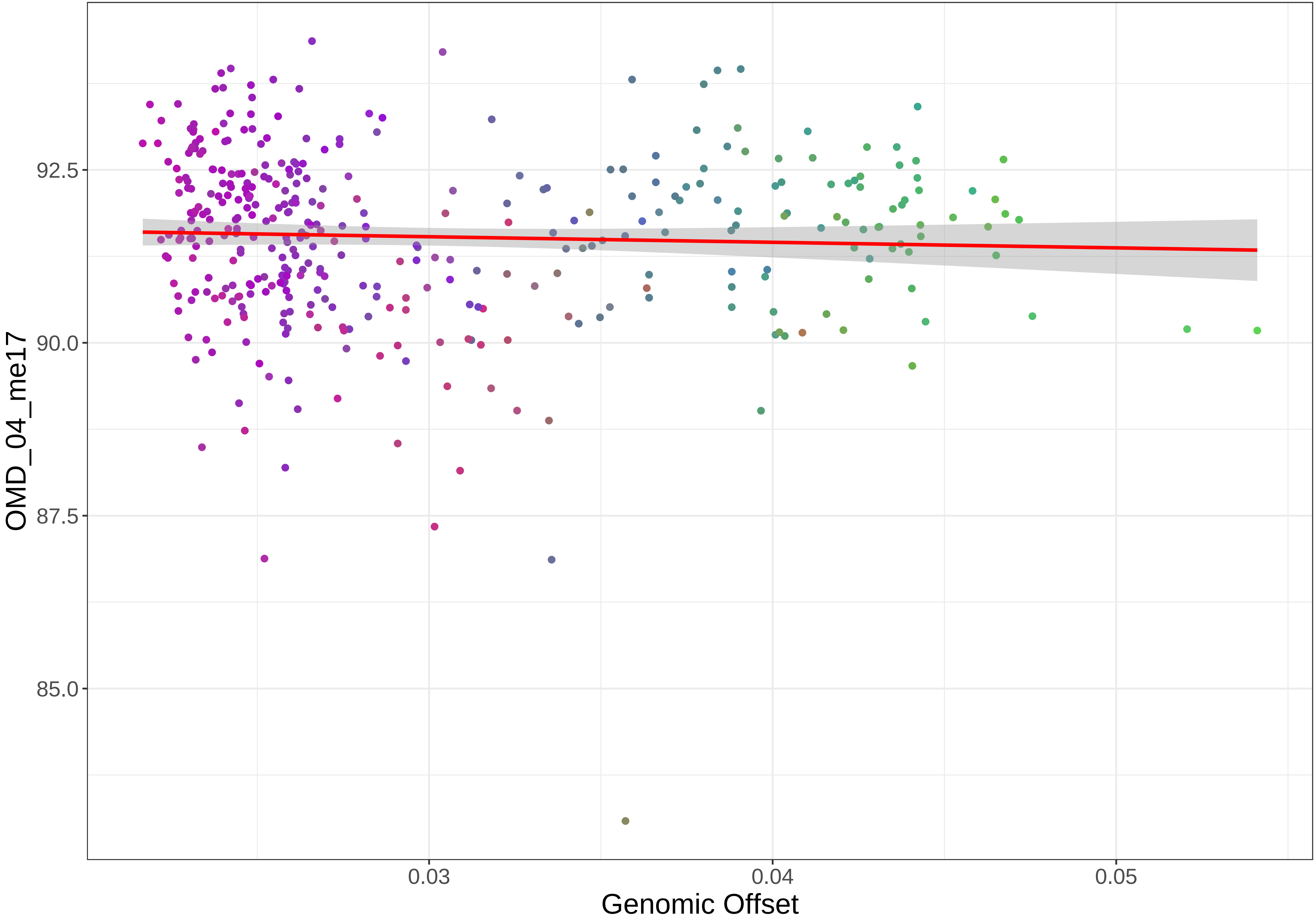

92.5
90.0
87.5
85.0
OMD_04_me17
0.03
0.04
0.05
Genomic Offset

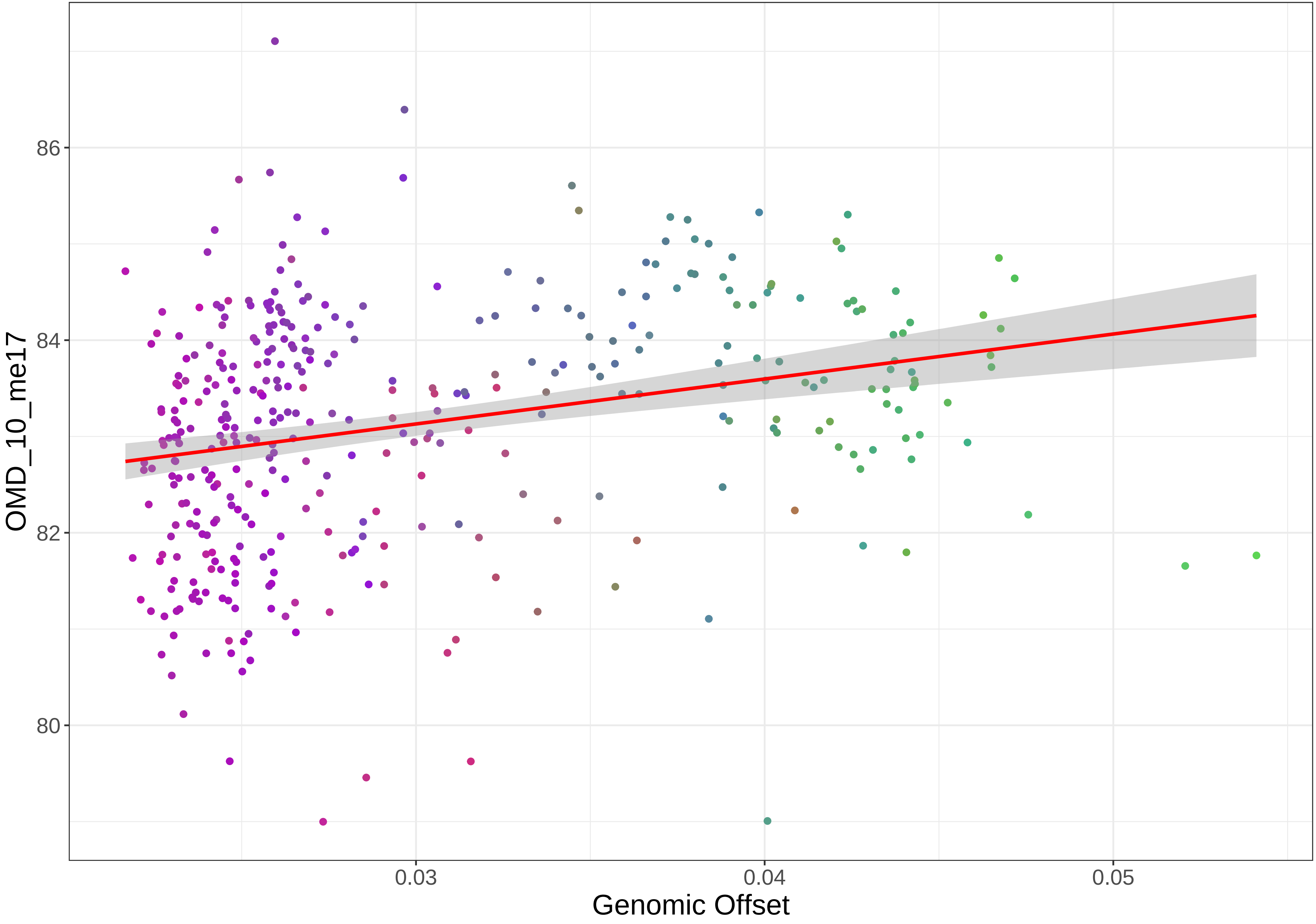

86
84
82
80
OMD_10_me17
0.03
0.04
0.05
Genomic Offset

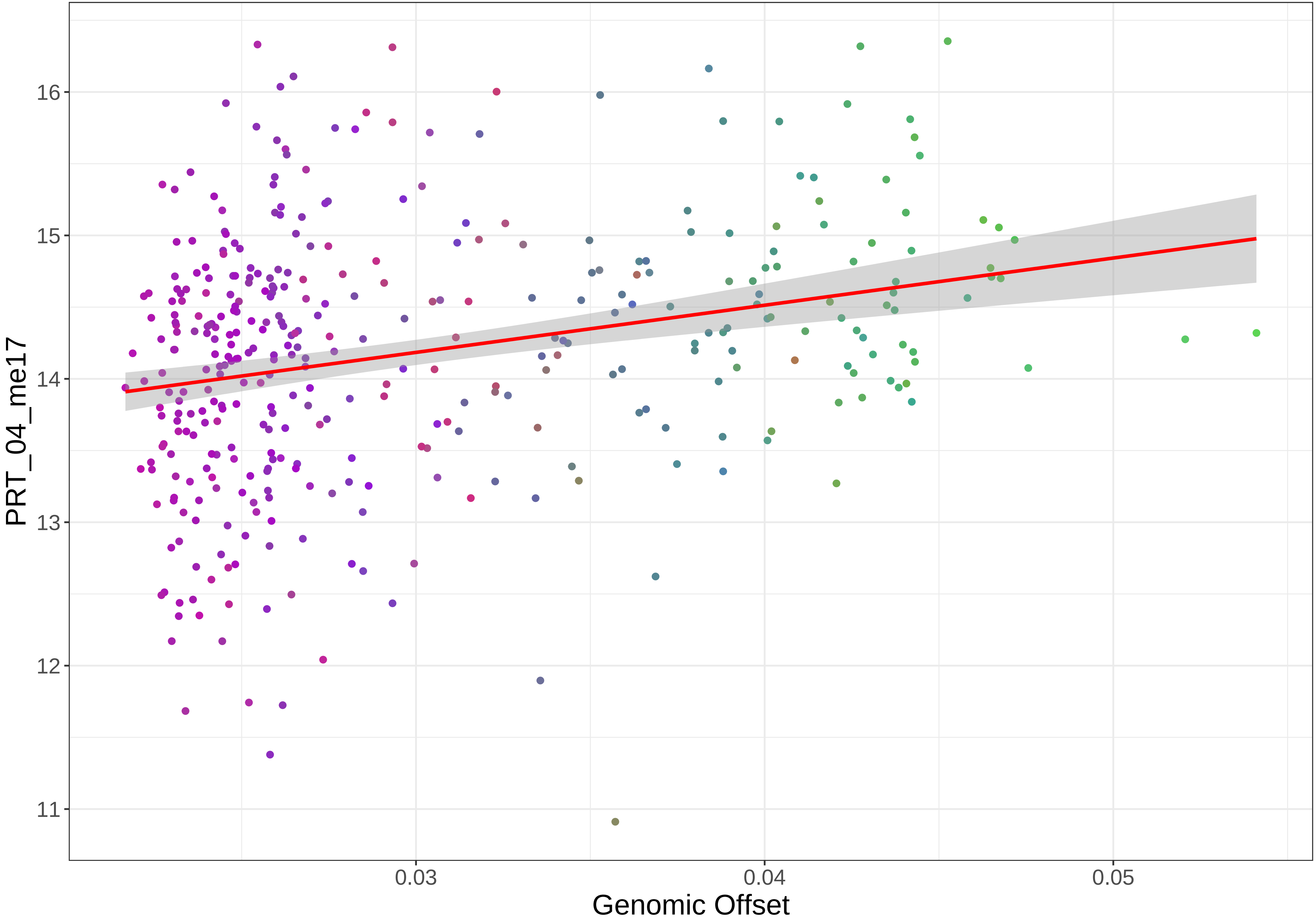

PRT_04_me17
16
15
14
13
12
11
0.03
0.04
0.05
Genomic Offset

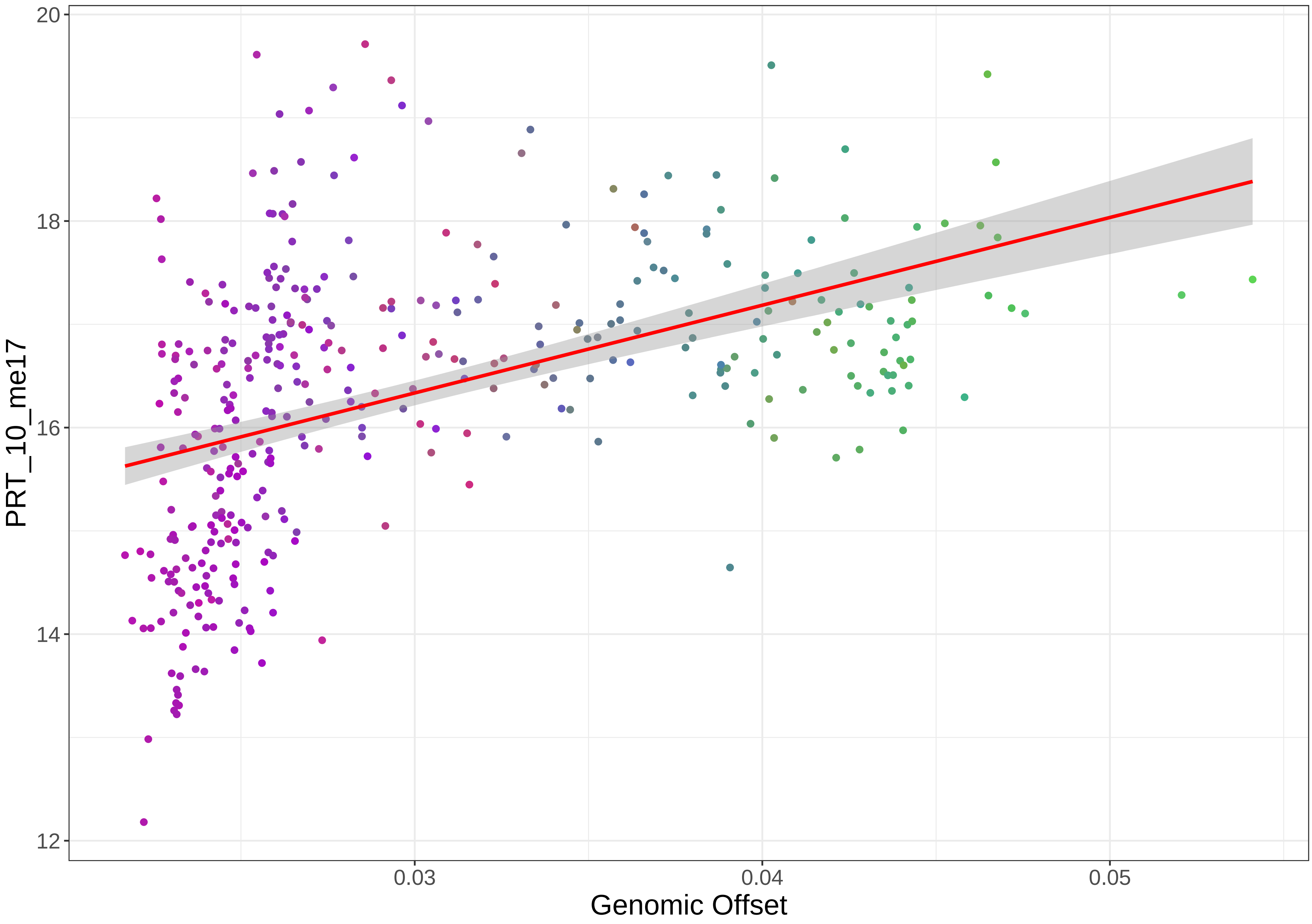

20
18
16
14
12
PRT_10_me17
0.03
0.04
0.05
Genomic Offset

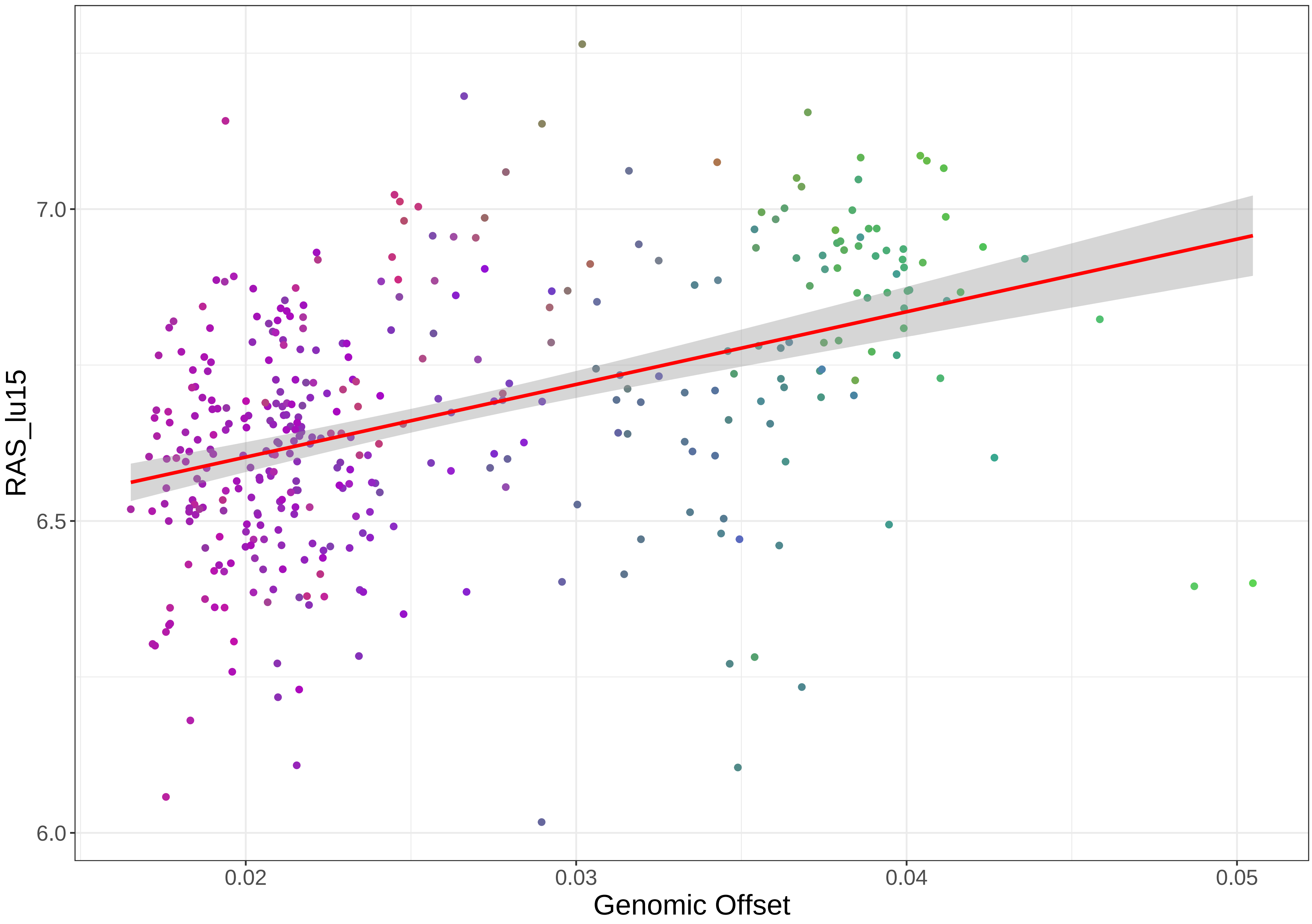

RAS_lu15
7.0
6.5
6.0
0.02
0.03
0.04
0.05
Genomic Offset

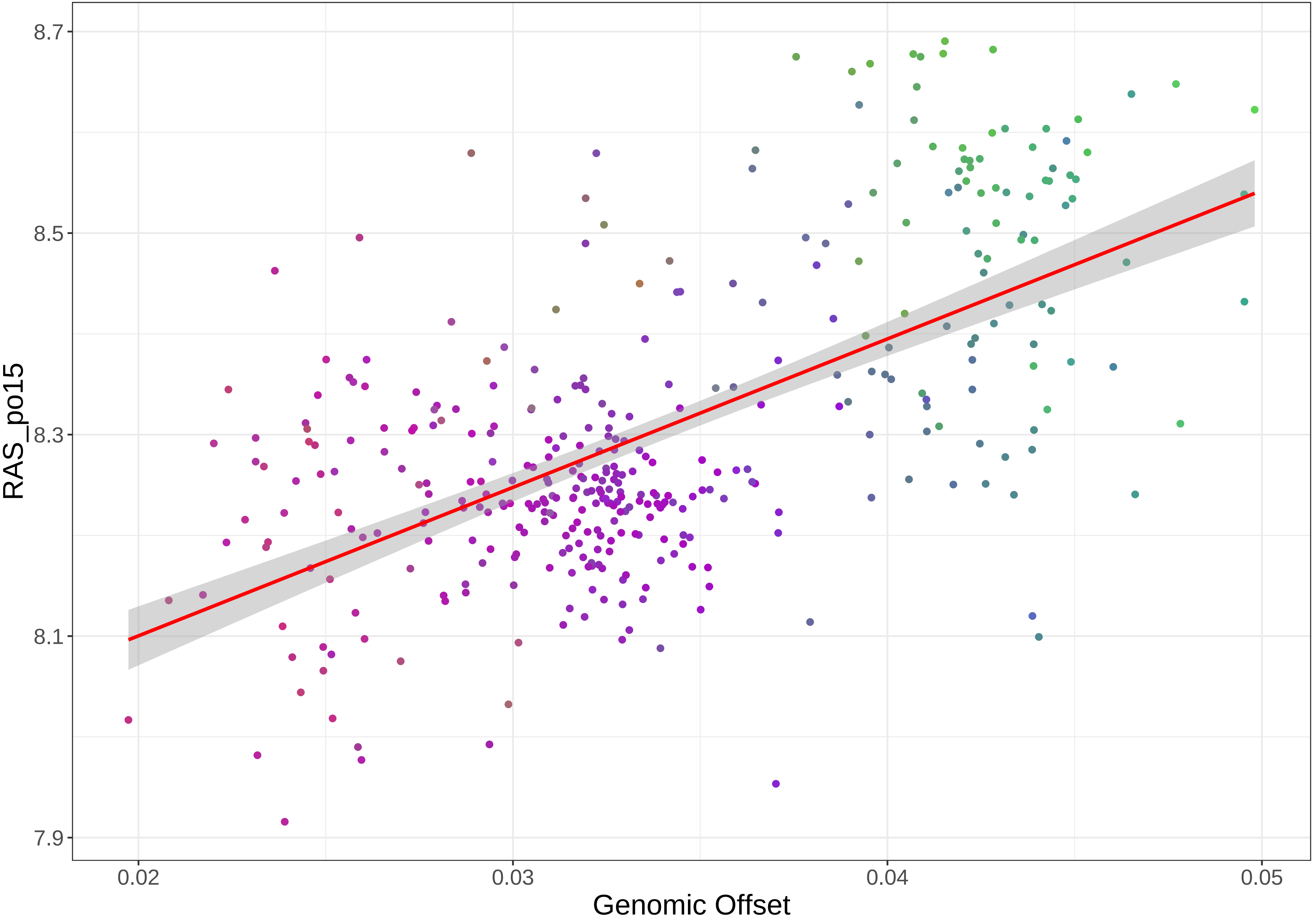
8.7
8.5
8.3
8.1
7.9
RAS_po15
0.02
0.03
0.04
0.05
Genomic Offset

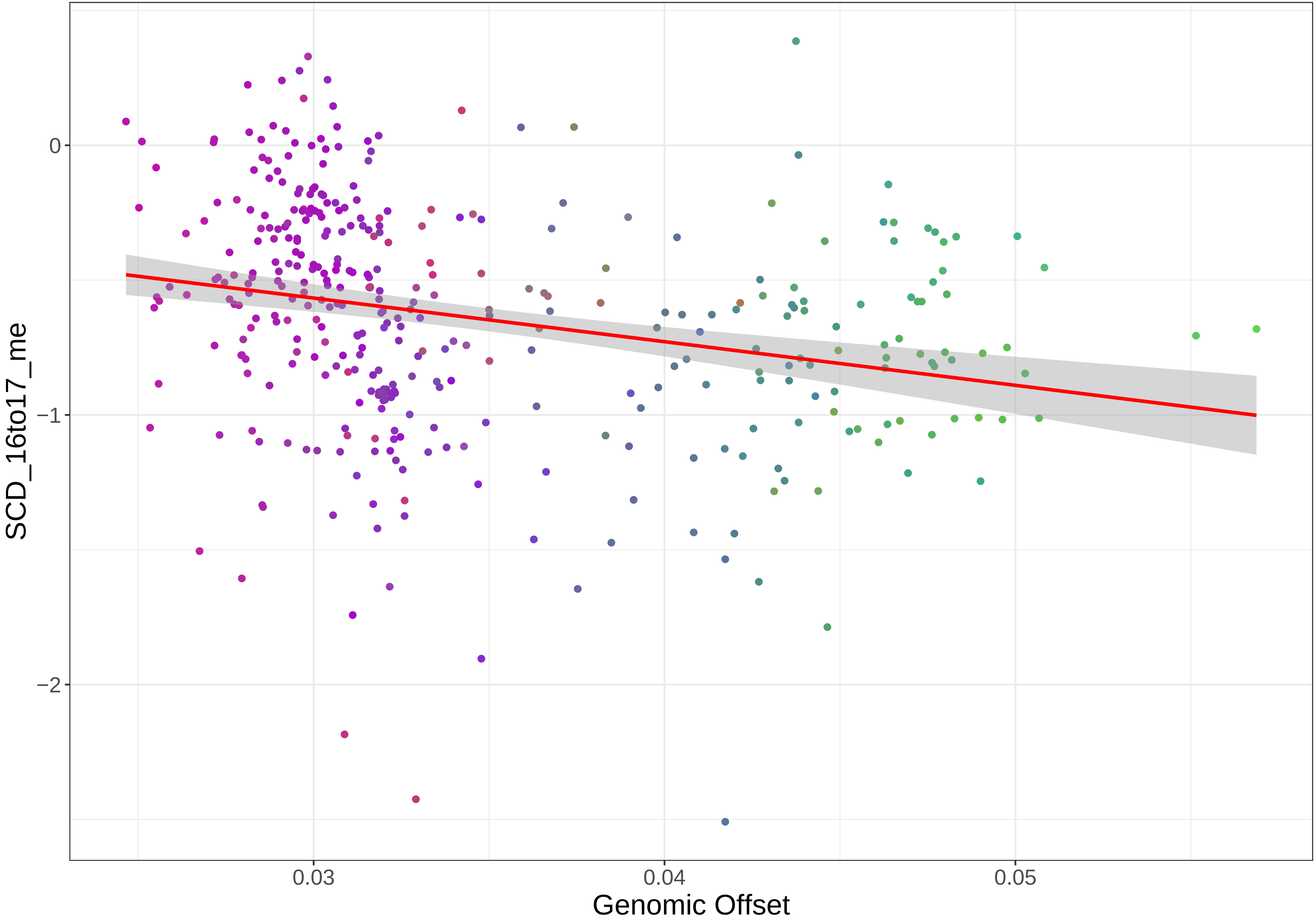
0
−1
−2
SCD_16to17_me
0.03
0.04
0.05
Genomic Offset

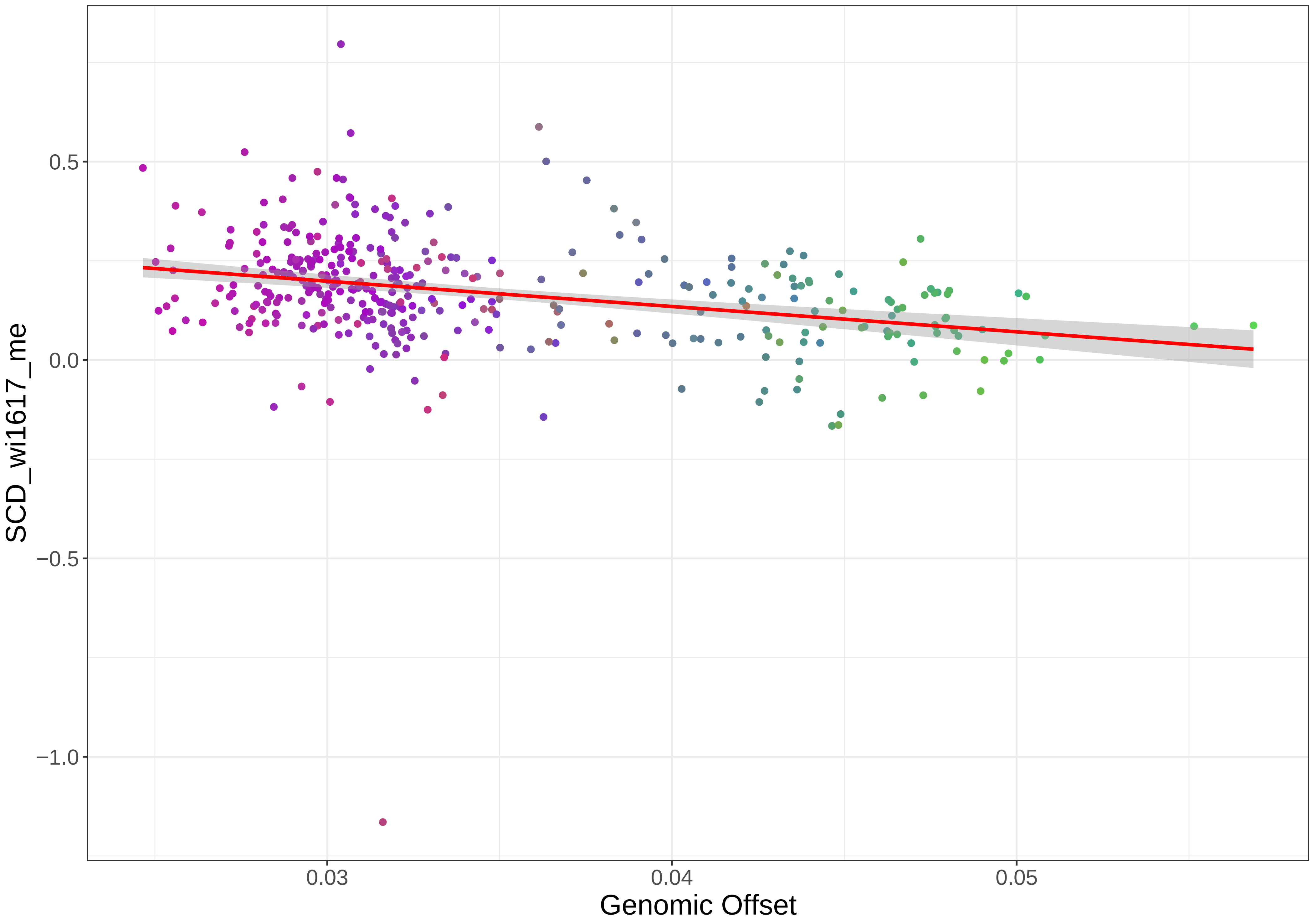

SCD_wi1617_me
0.5
0.0
−0.5
−1.0
0.03
0.04
0.05
Genomic Offset

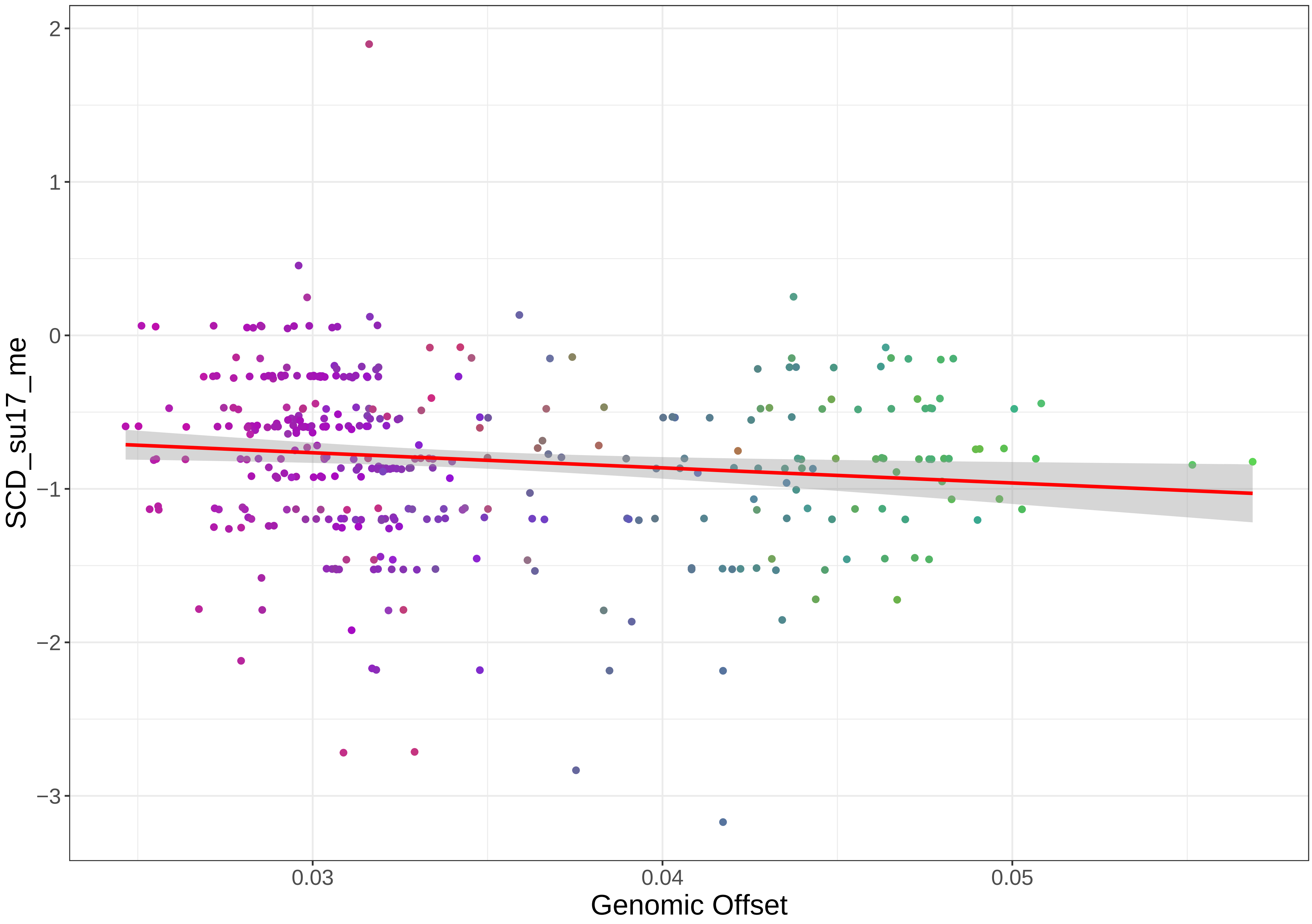

2
1
0
−1
−2
−3
SCD_su17_me
0.03
0.04
0.05
Genomic Offset

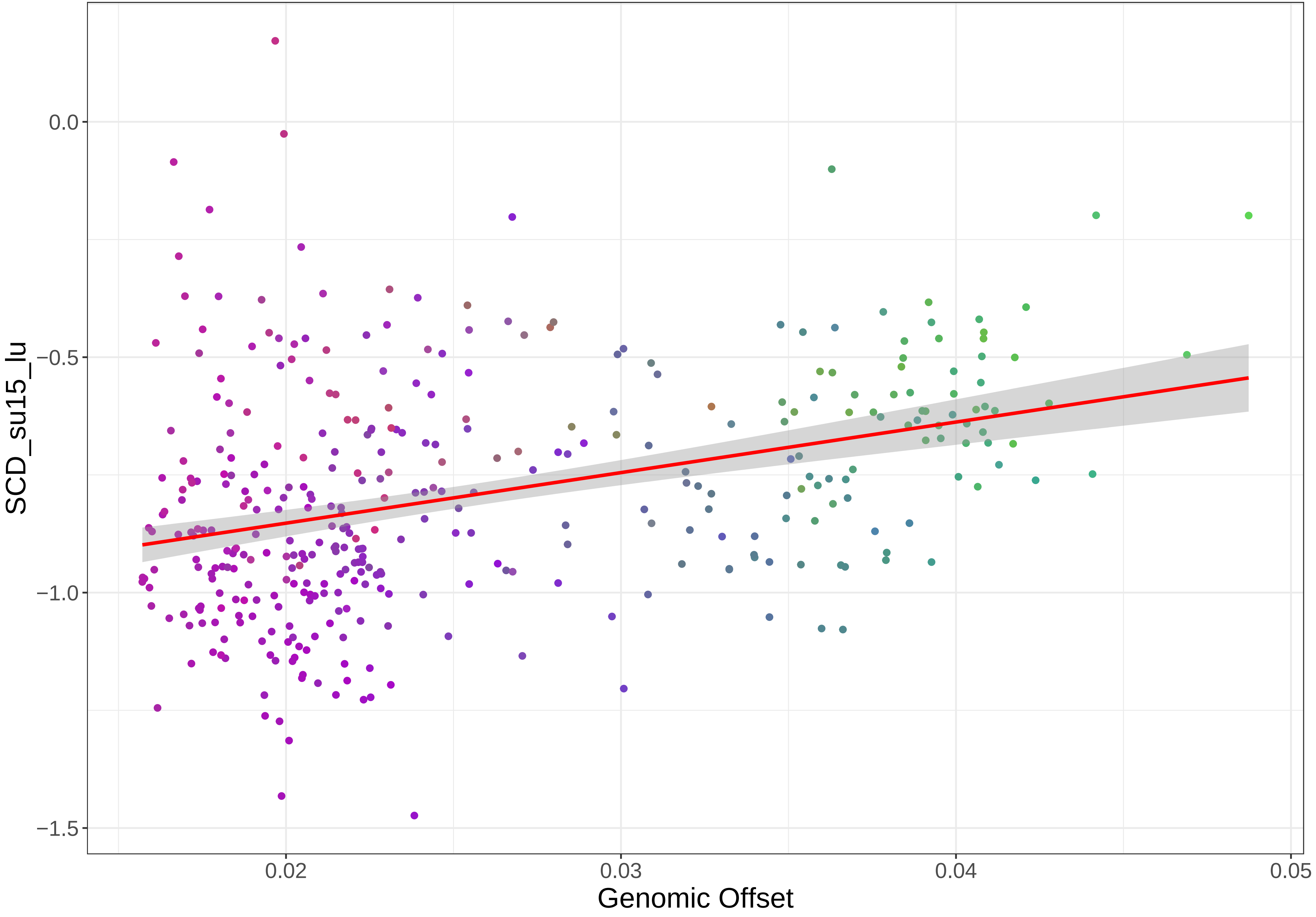

0.0
−0.5
−1.0
−1.5
SCD_su15_lu
0.02
0.03
0.04
0.05
Genomic Offset

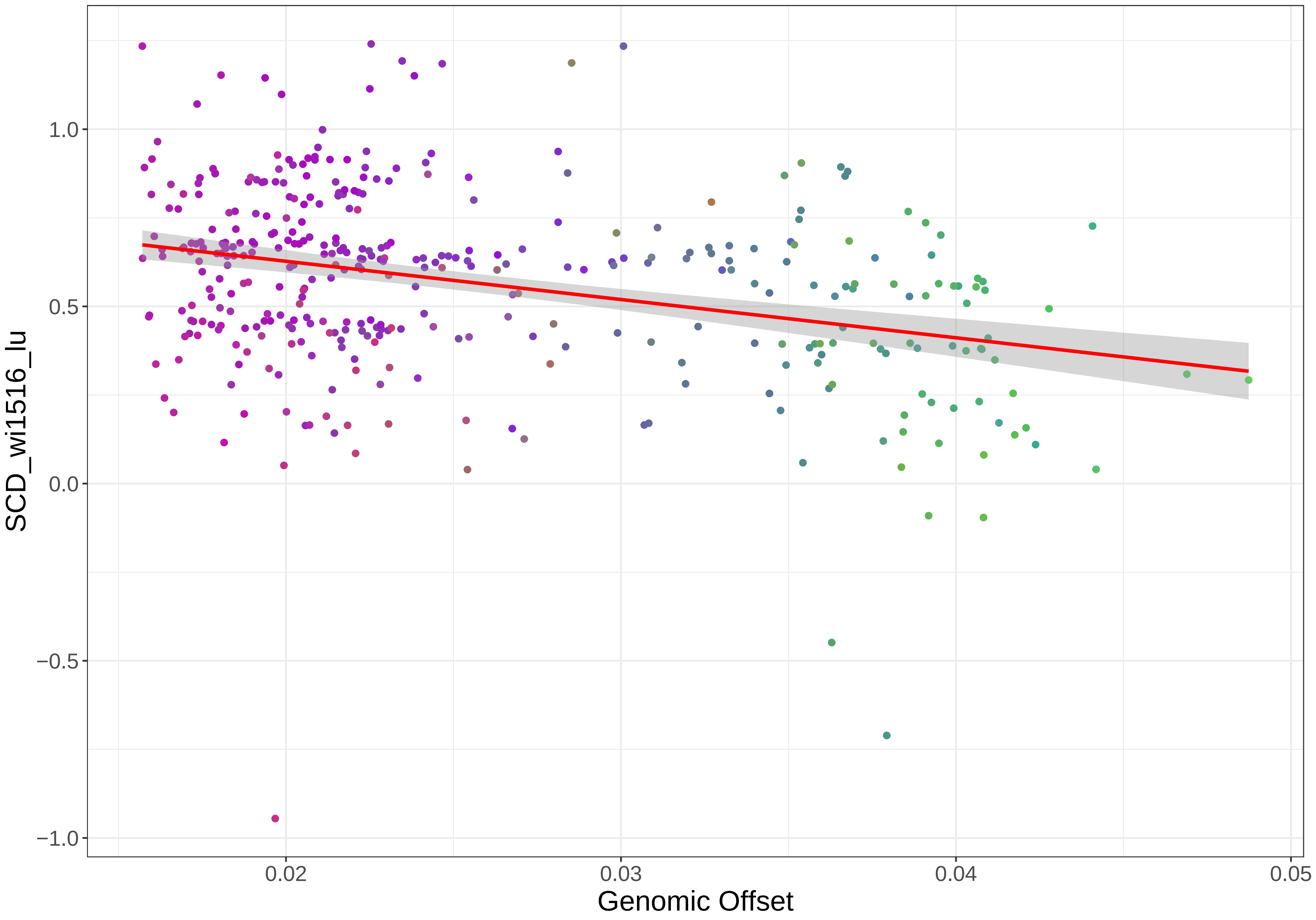

1.0
0.5
0.0
−0.5
−1.0
SCD_wi1516_lu
0.02
0.03
0.04
0.05
Genomic Offset

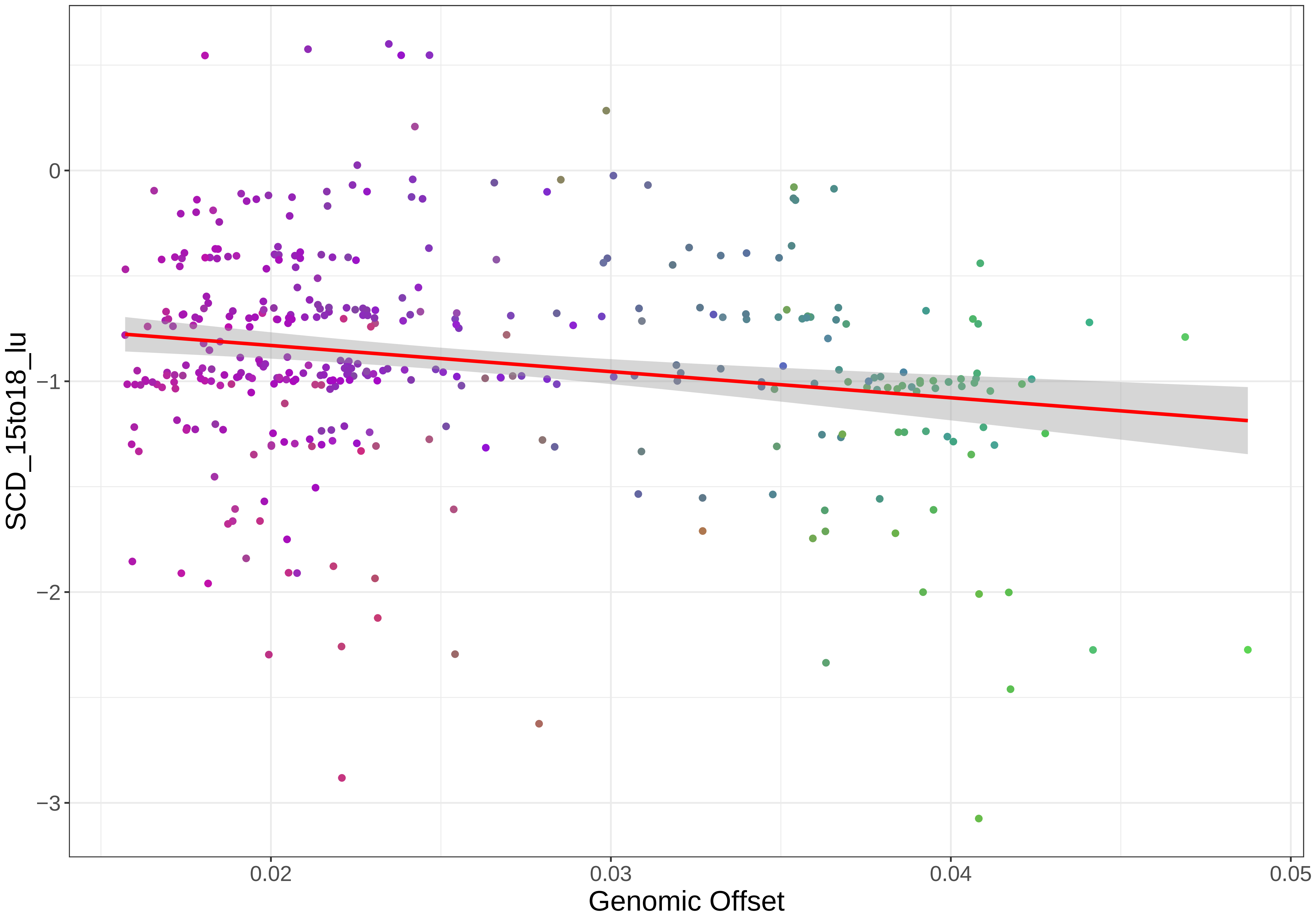

SCD_15to18_lu
0
−1
−2
−3
0.02
0.03
0.04
0.05
Genomic Offset

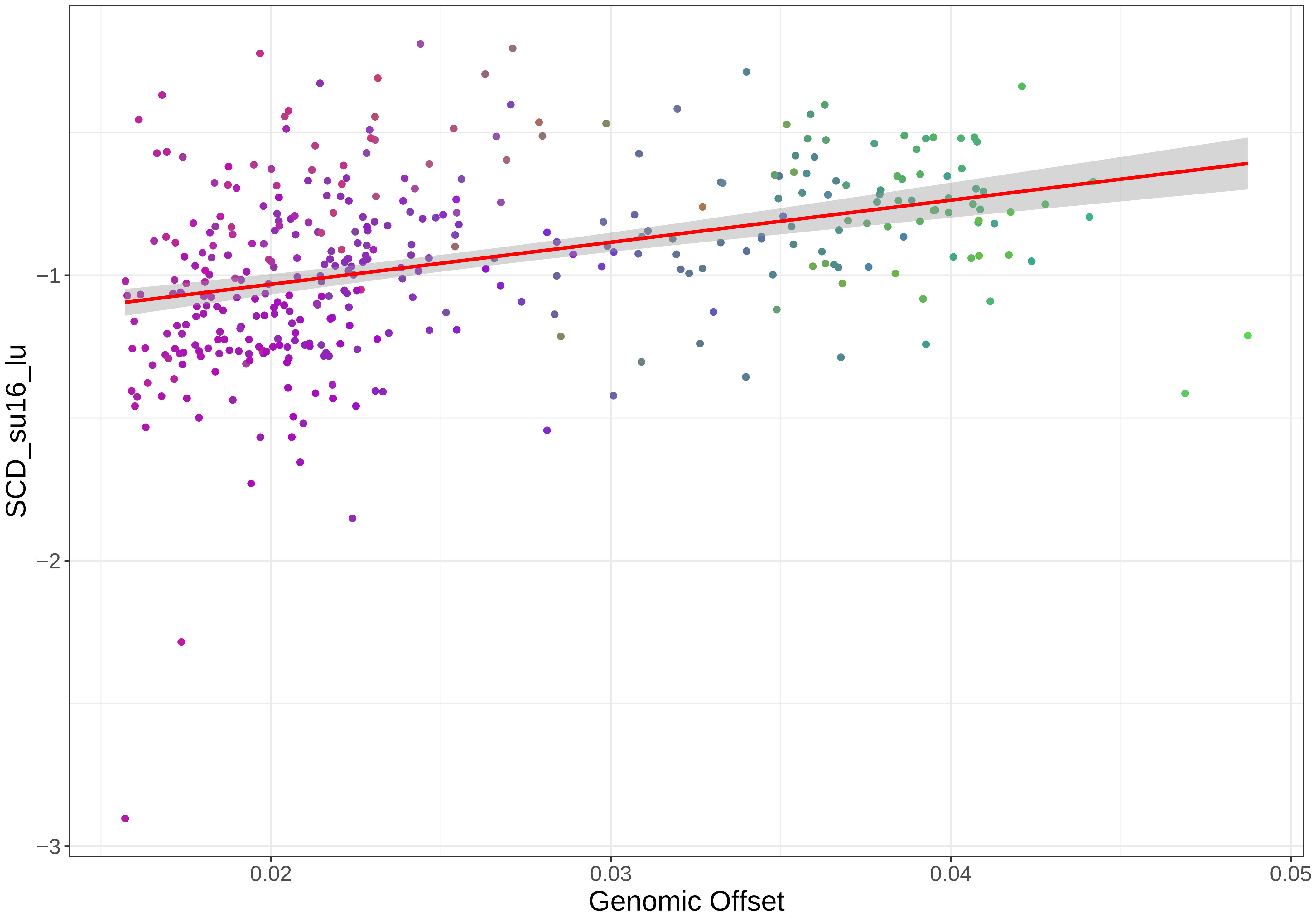
SCD_su16_lu
−1
−2
−3
0.02
0.03
0.04
0.05
Genomic Offset

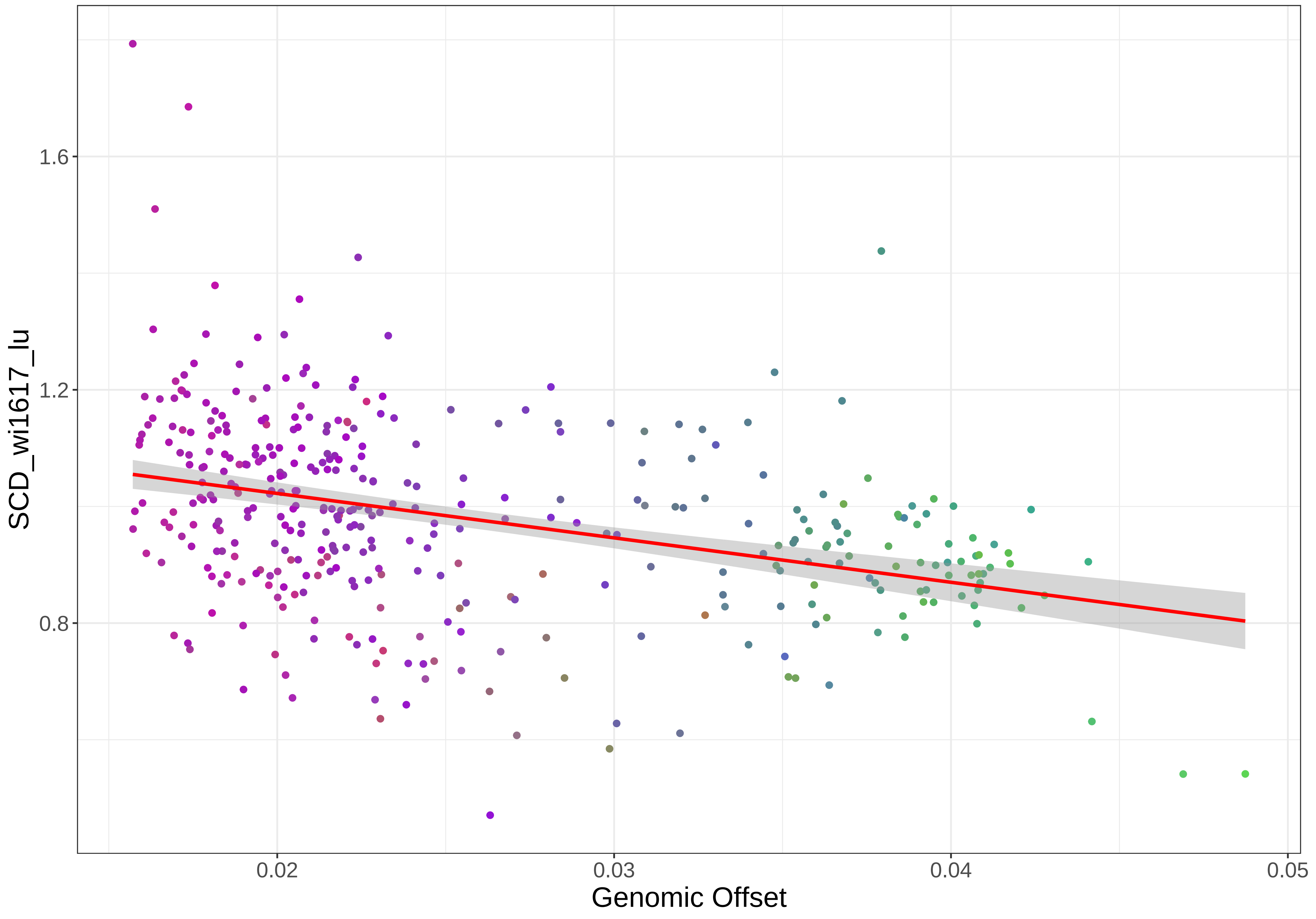

SCD_wi1617_lu
1.6
1.2
0.8
0.02
0.03
0.04
0.05
Genomic Offset

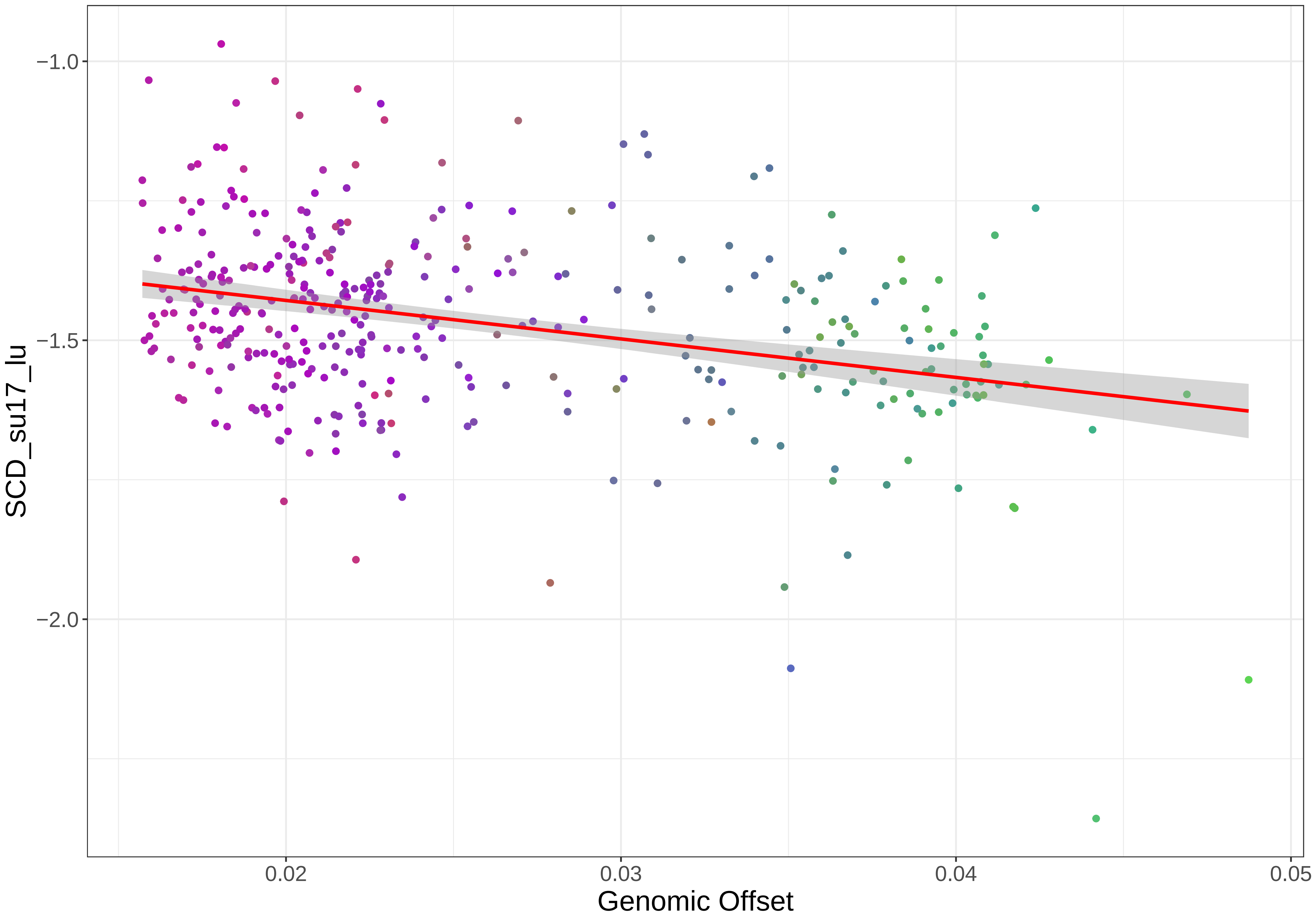

SCD_su17_lu
−1.0
−1.5
−2.0
0.02
0.03
0.04
0.05
Genomic Offset

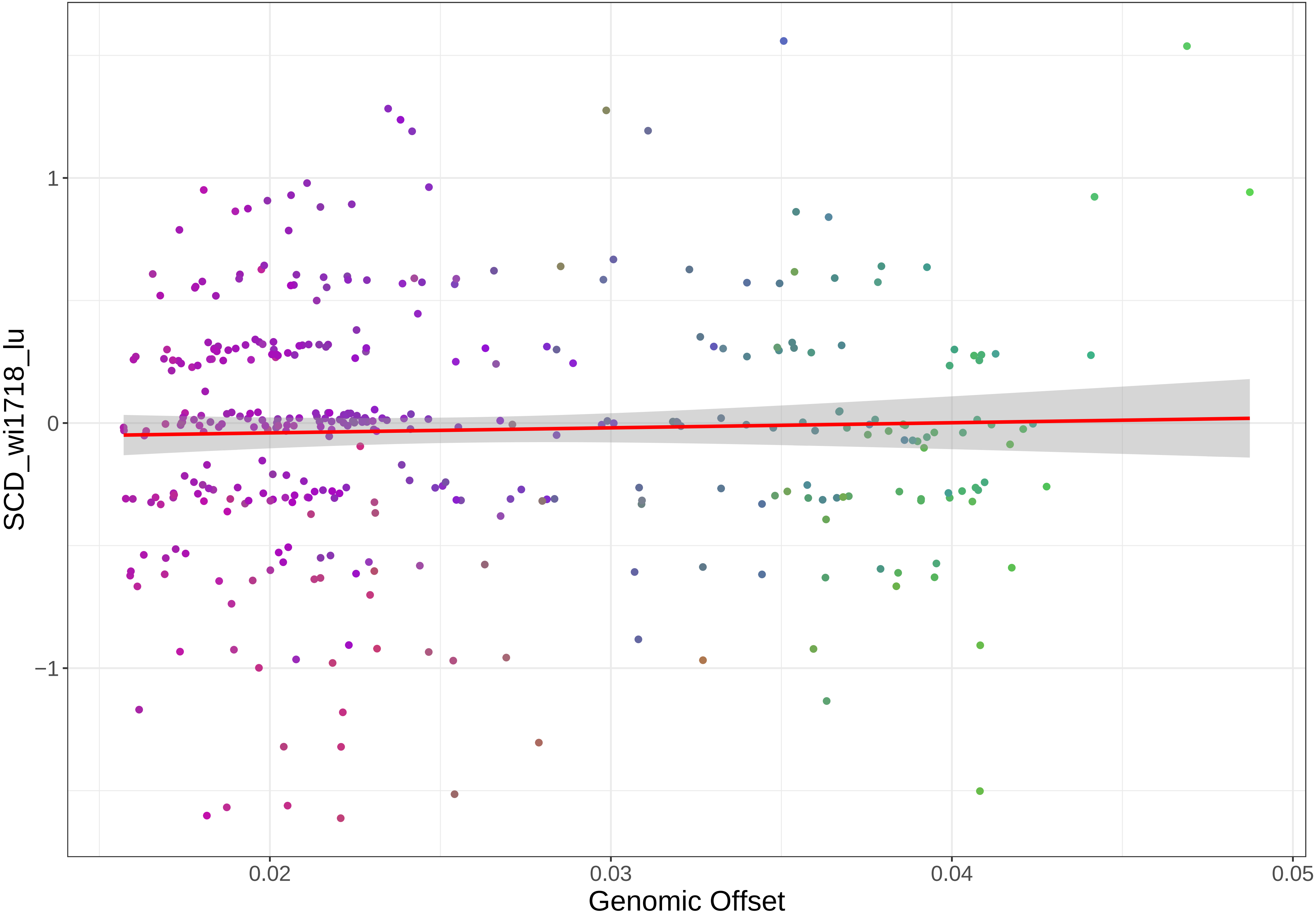

SCD_wi1718_lu
1
0
−1
0.02
0.03
0.04
0.05
Genomic Offset

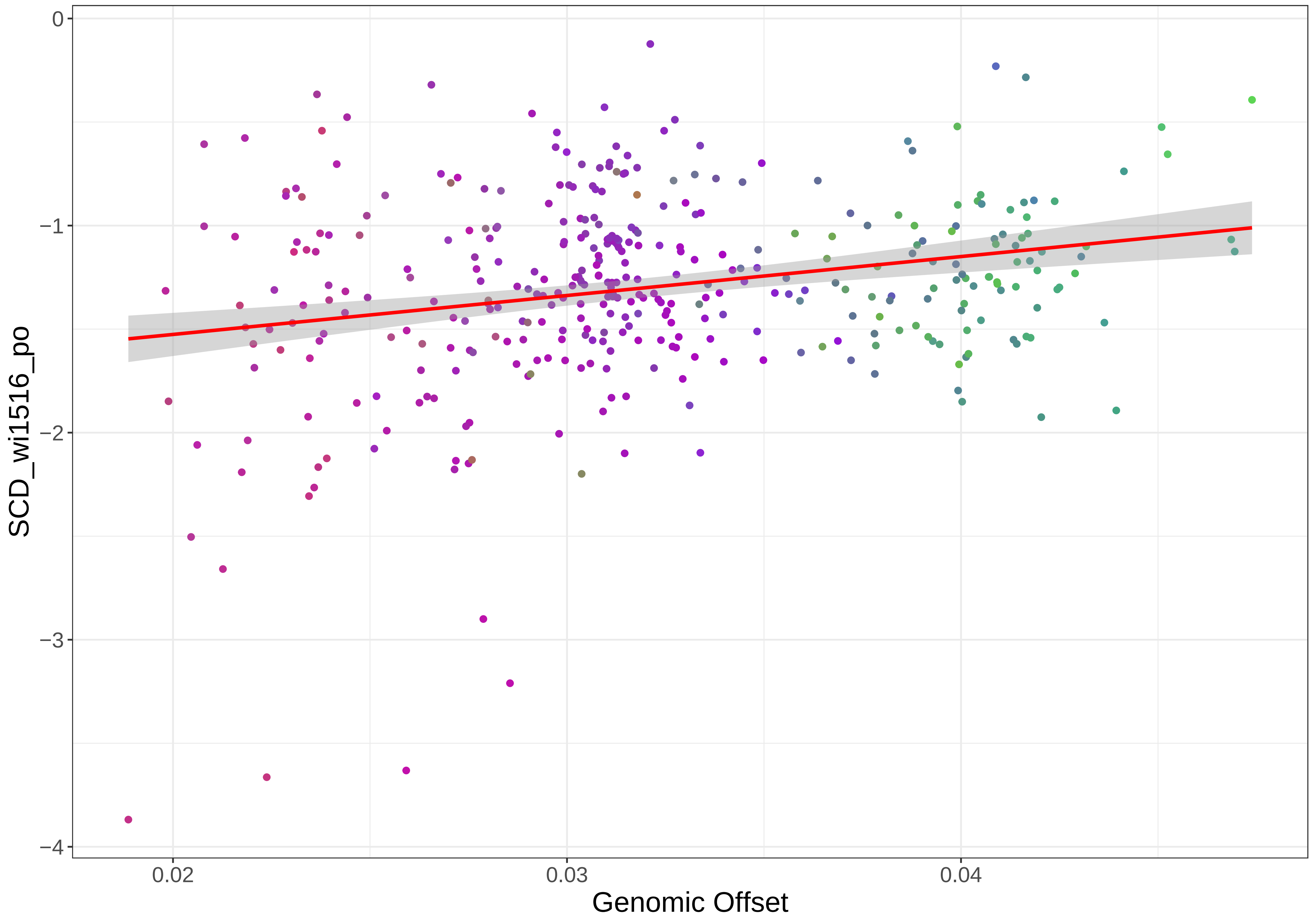

0
−1
−2
−3
−4
SCD_wi1516_po
0.02
0.03
0.04
Genomic Offset

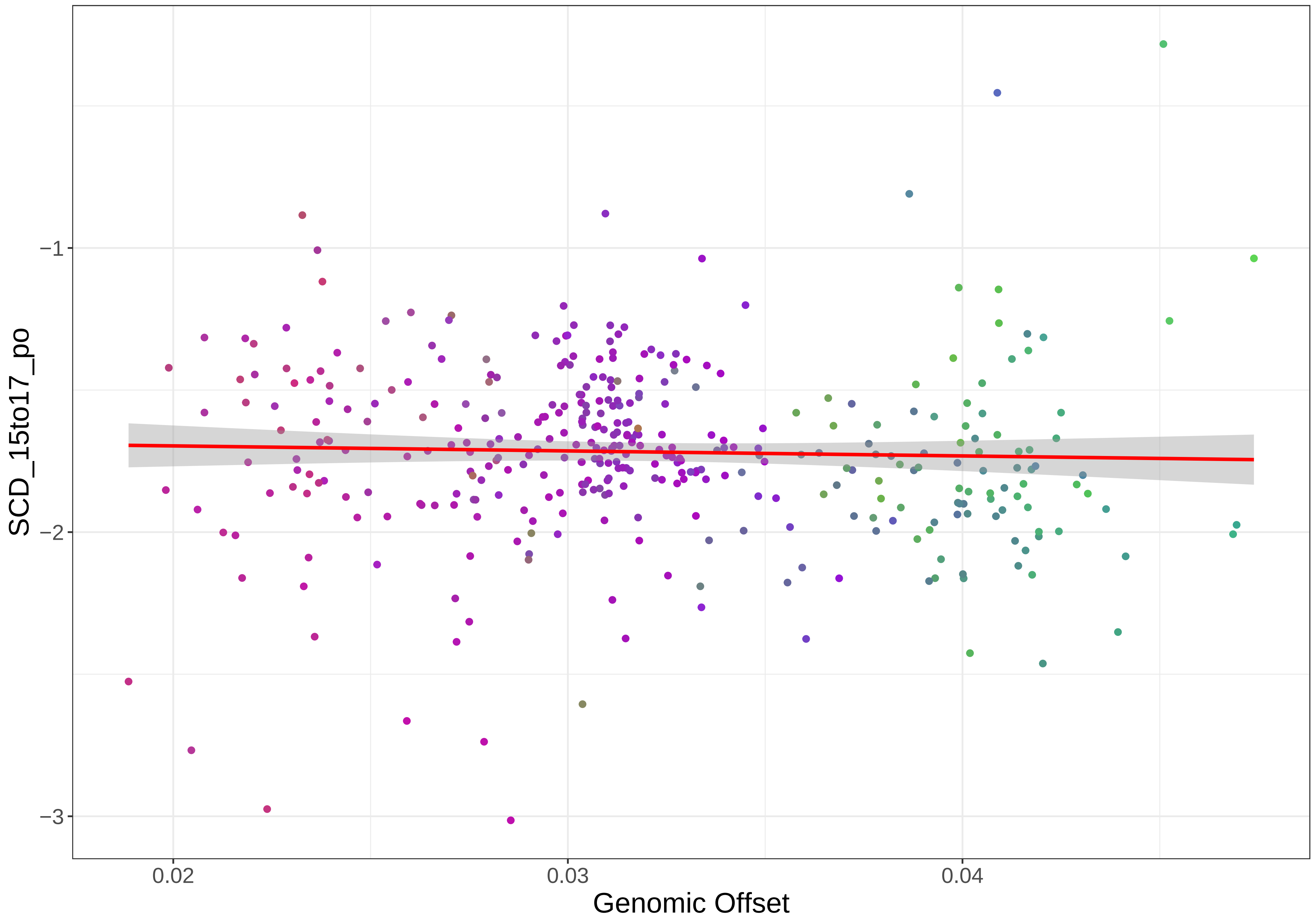

SCD_15to17_po
−1
−2
−3
0.02
0.03
0.04
Genomic Offset

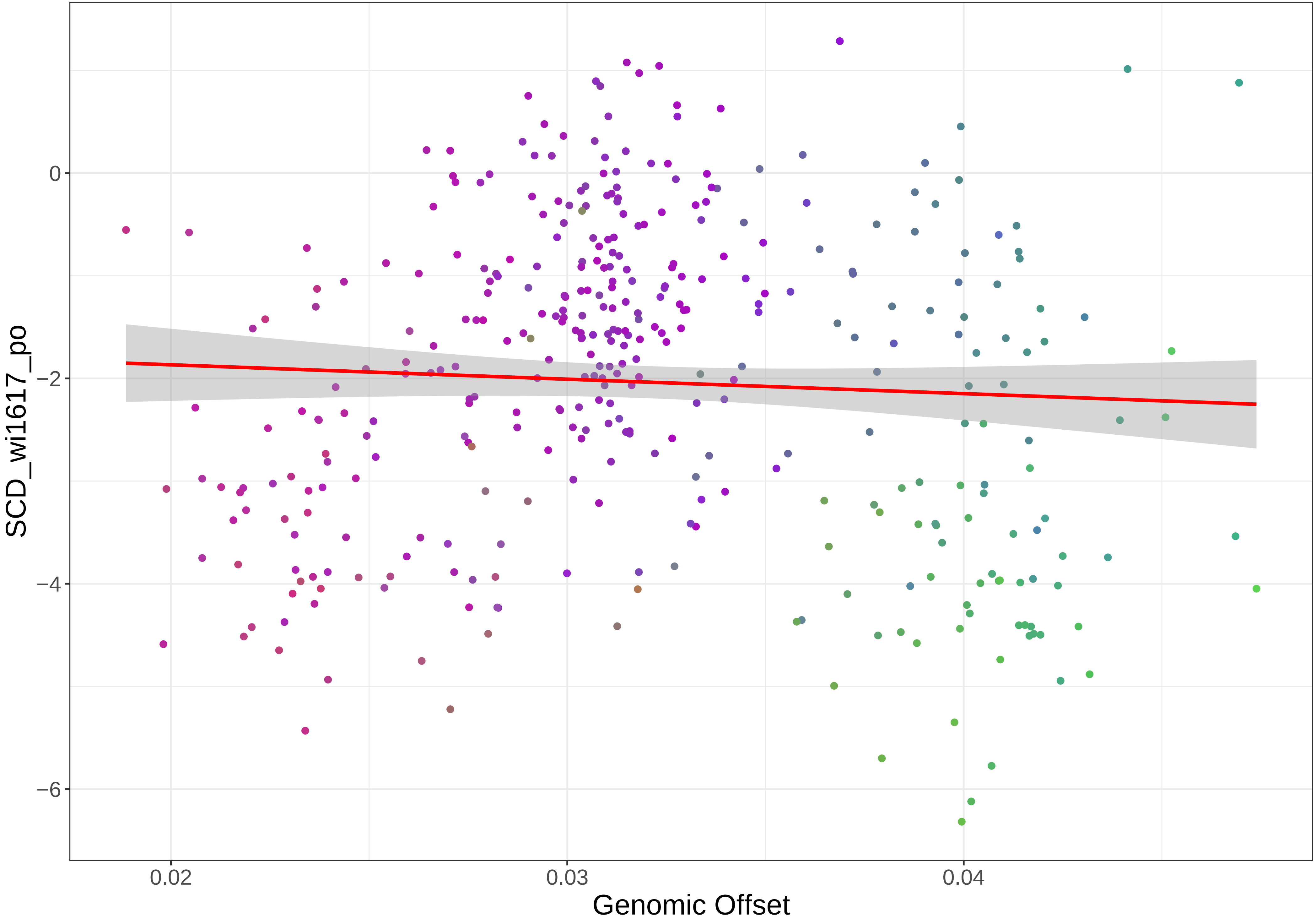

SCD_wi1617_po
0
−2
−4
−6
0.02
0.03
0.04
Genomic Offset

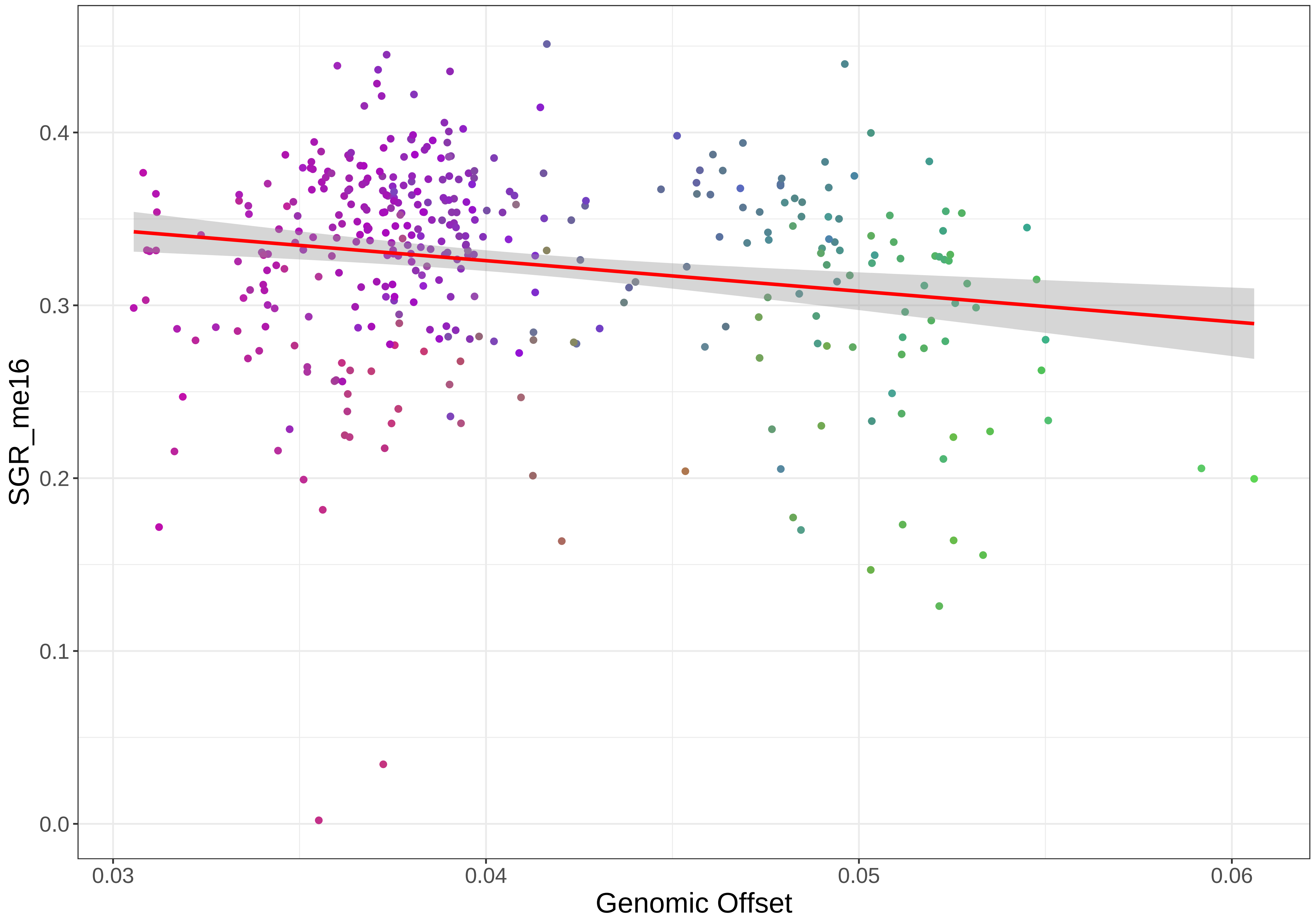

SGR_me16
0.0
0.1
0.2
0.3
0.4
0.03
0.04
0.05
0.06
Genomic Offset

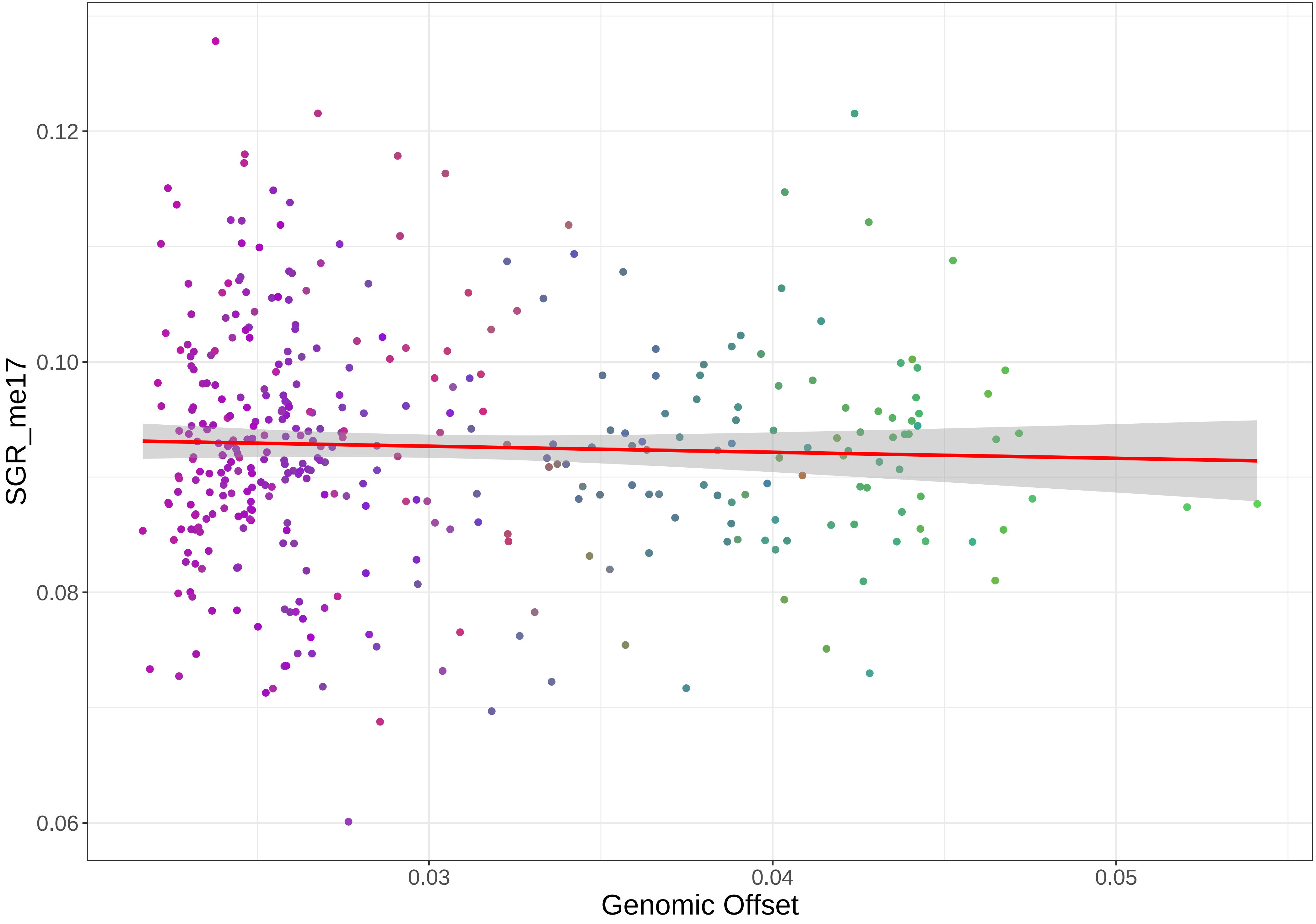

0.12
0.10
0.08
0.06
SGR_me17
0.03
0.04
0.05
Genomic Offset

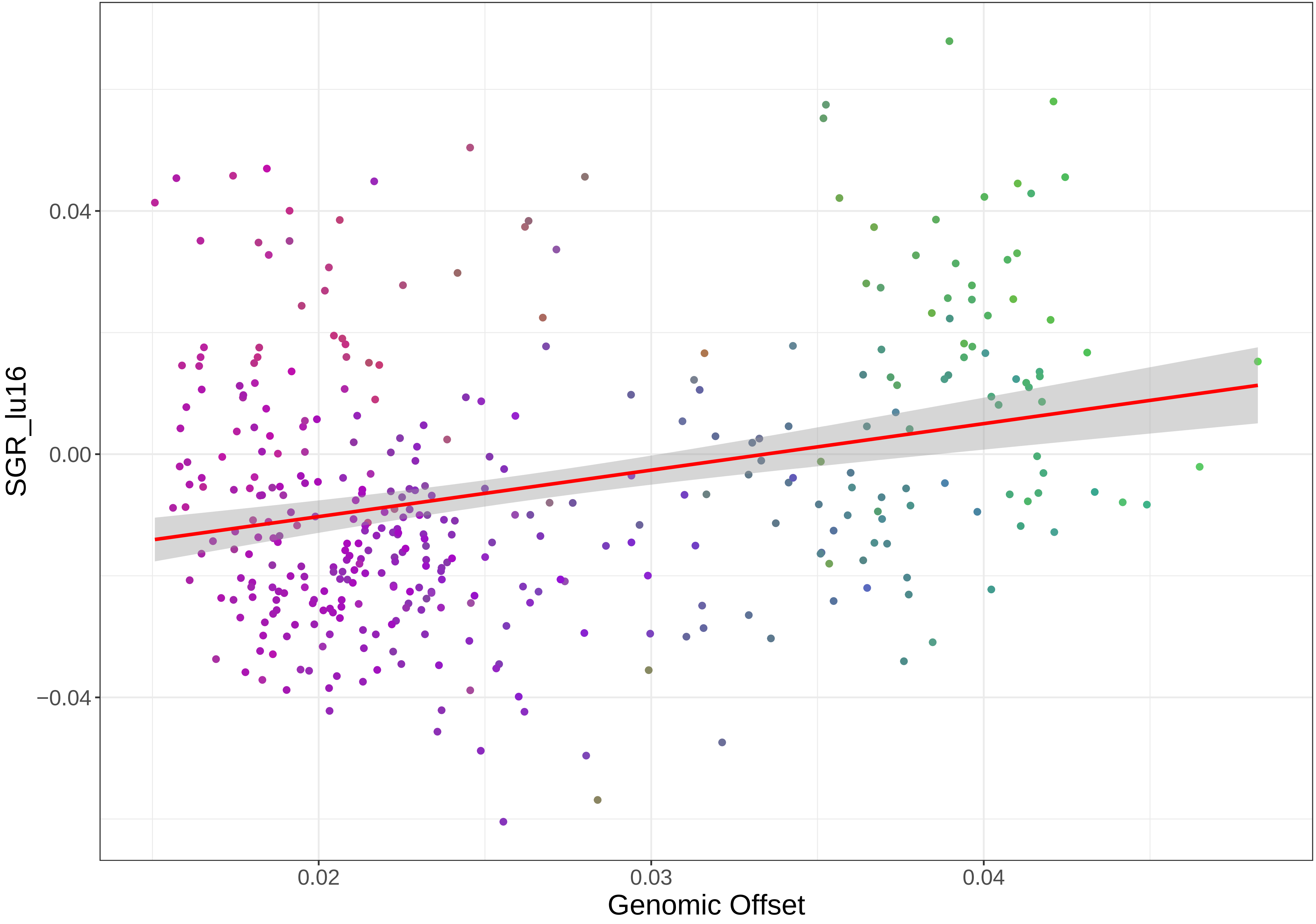

SGR_lu16
0.04
0.00
−0.04
0.02
0.03
0.04
Genomic Offset

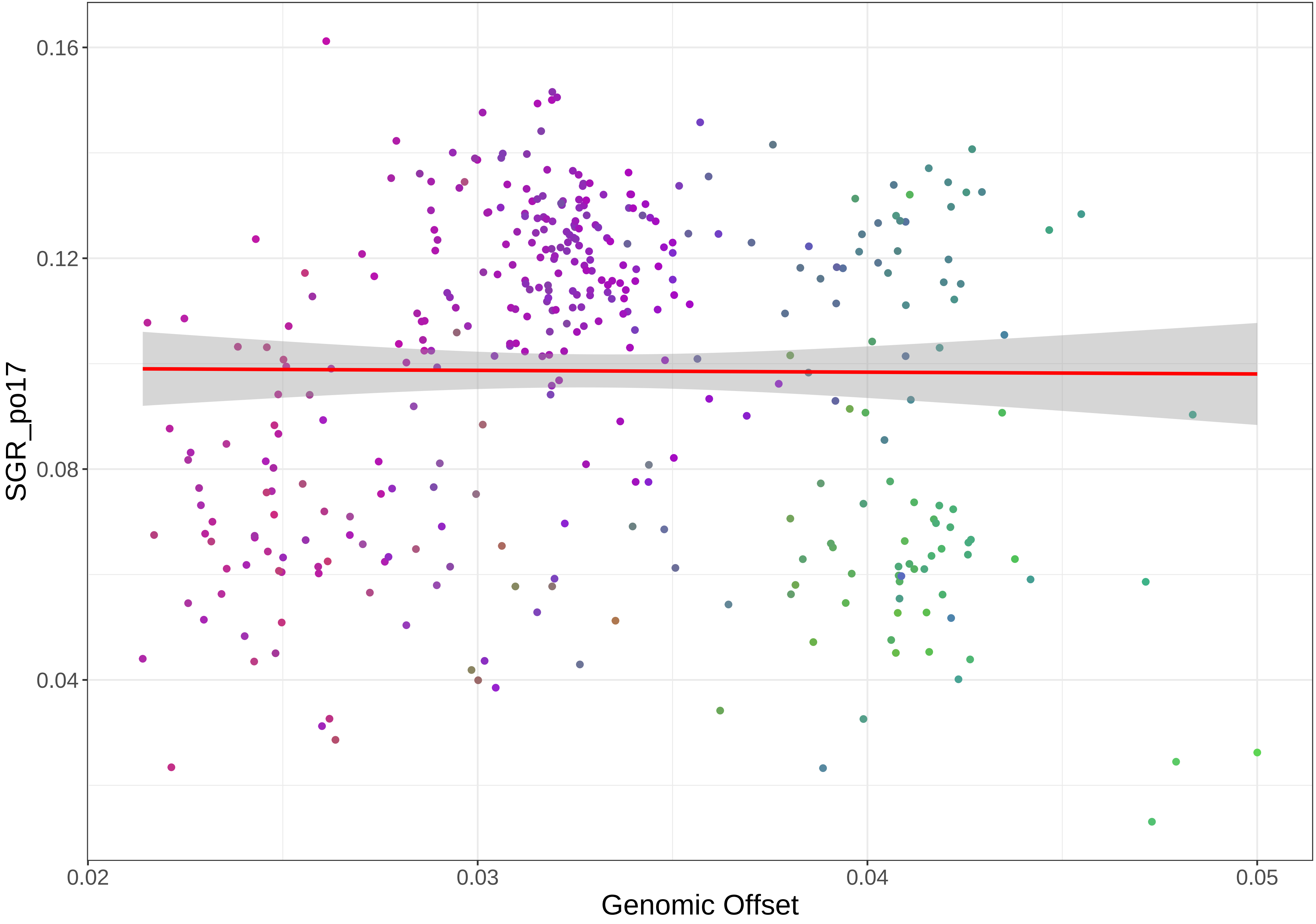
0.16
0.12
0.08
0.04
SGR_po17
0.02
0.03
0.04
0.05
Genomic Offset

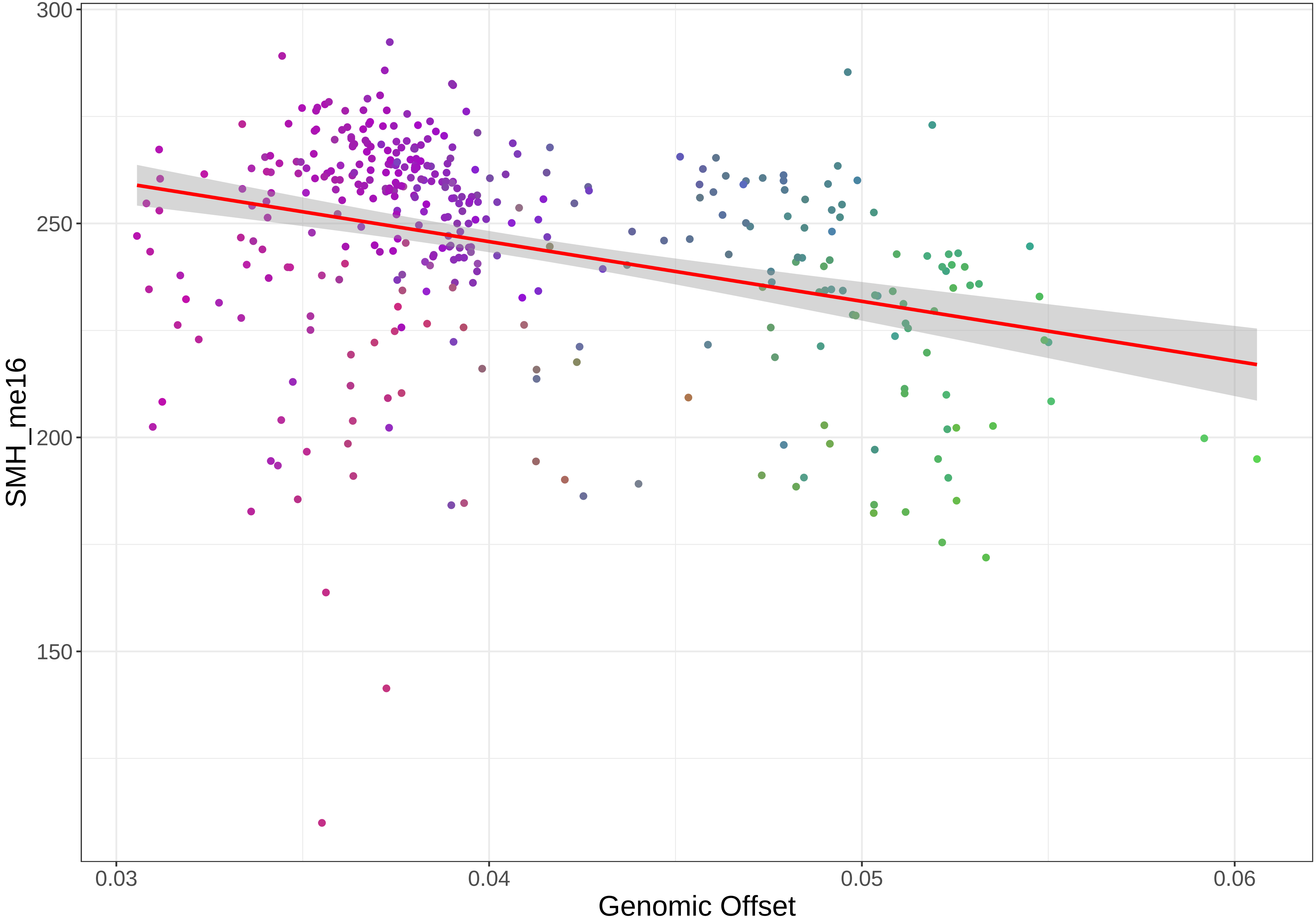

300
250
200
150
SMH_me16
0.03
0.04
0.05
0.06
Genomic Offset

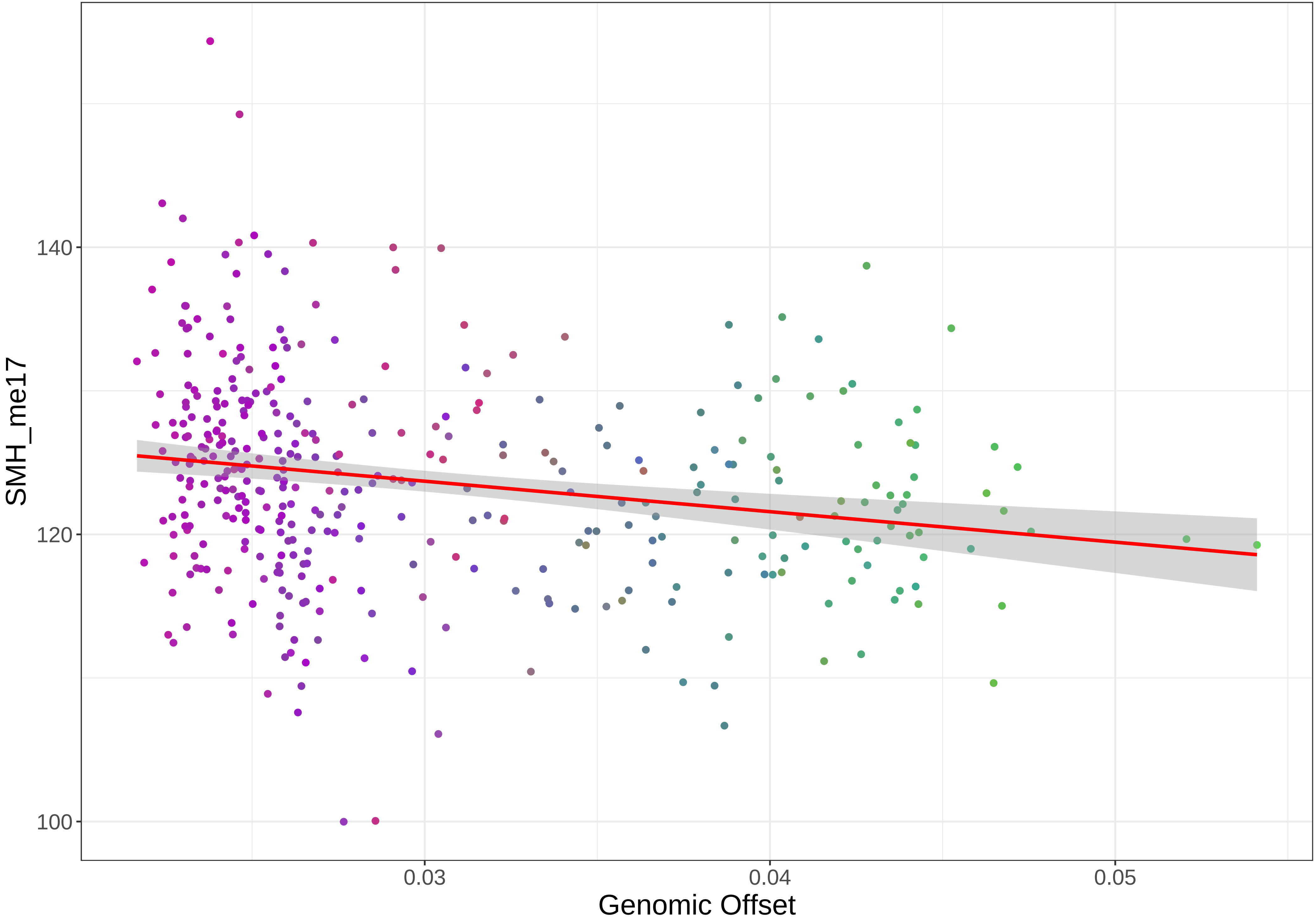
SMH_me17
140
120
100
0.03
0.04
0.05
Genomic Offset

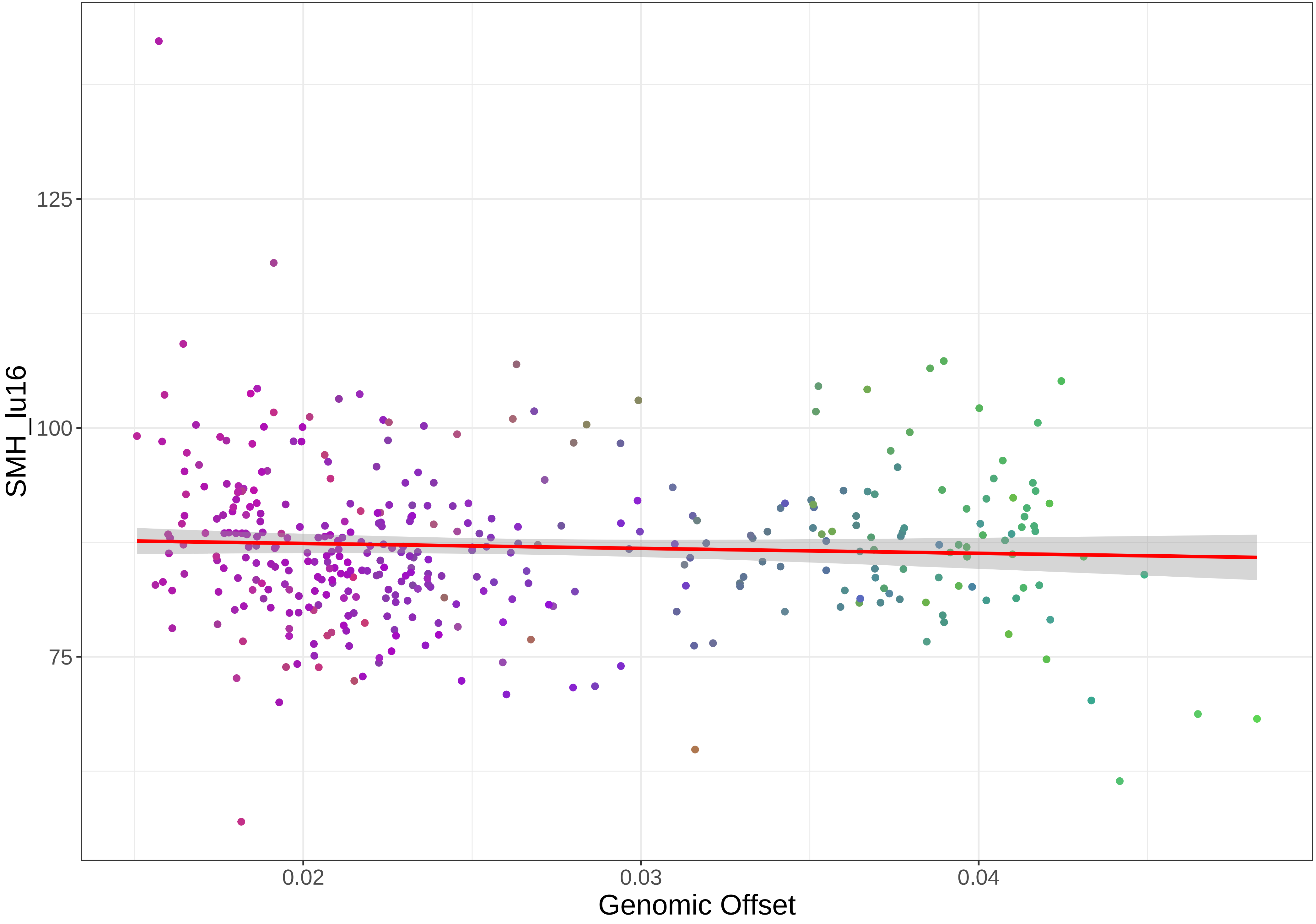

SMH_lu16
125
100
75
0.02
0.03
0.04
Genomic Offset

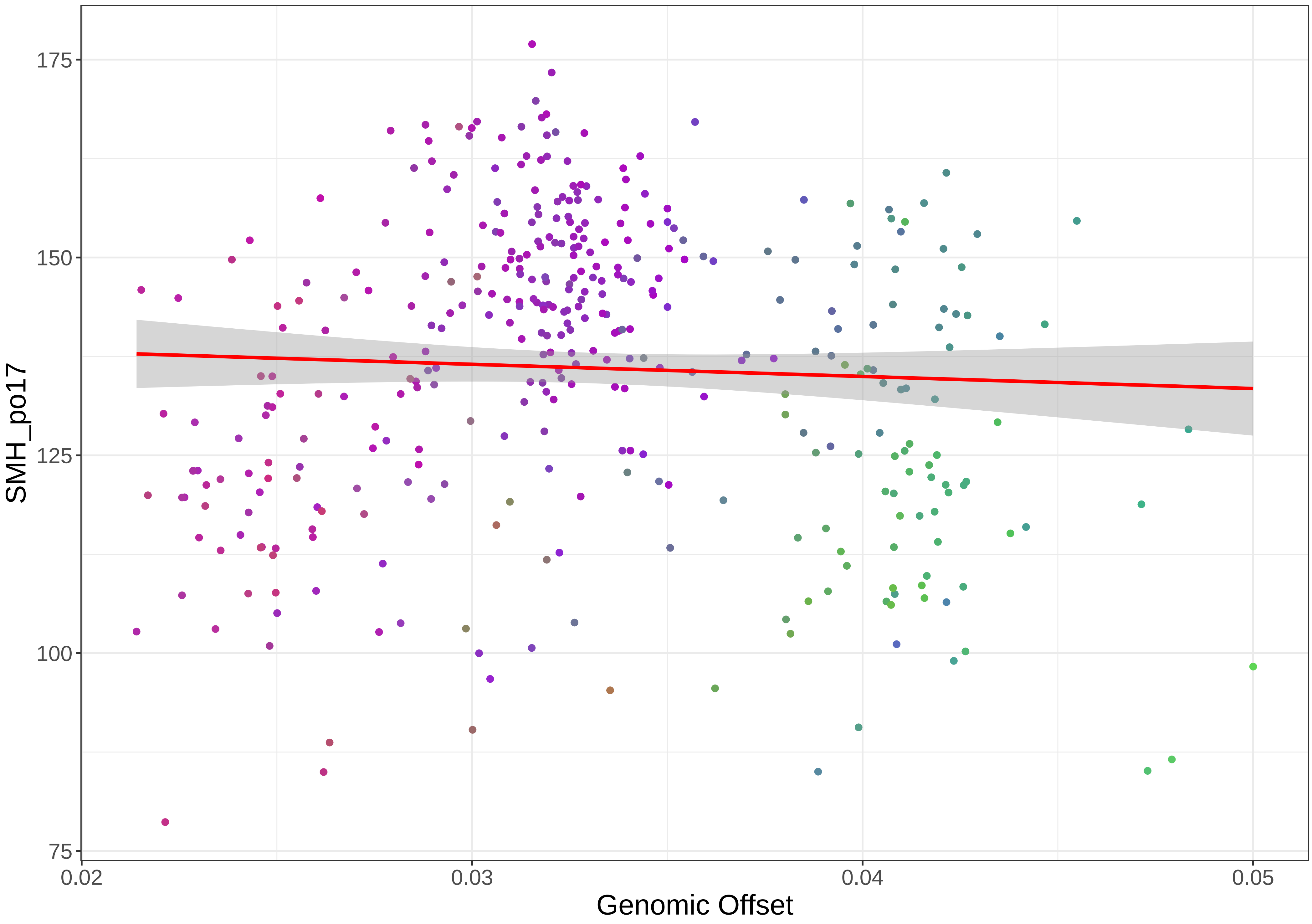
175
150
125
100
75
SMH_po17
0.02
0.03
0.04
0.05
Genomic Offset

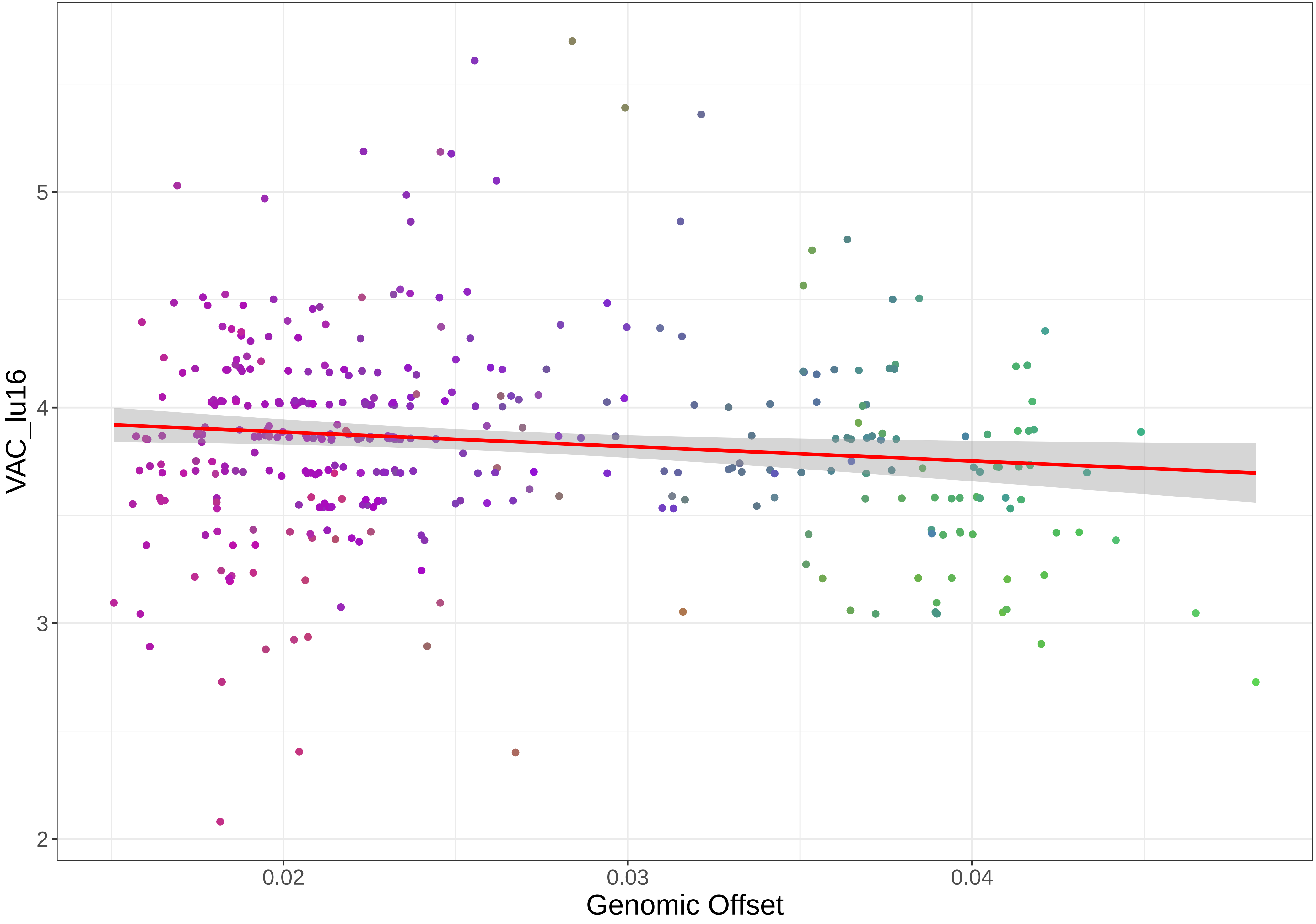

VAC_lu16
5
4
3
2
0.02
0.03
0.04
Genomic Offset

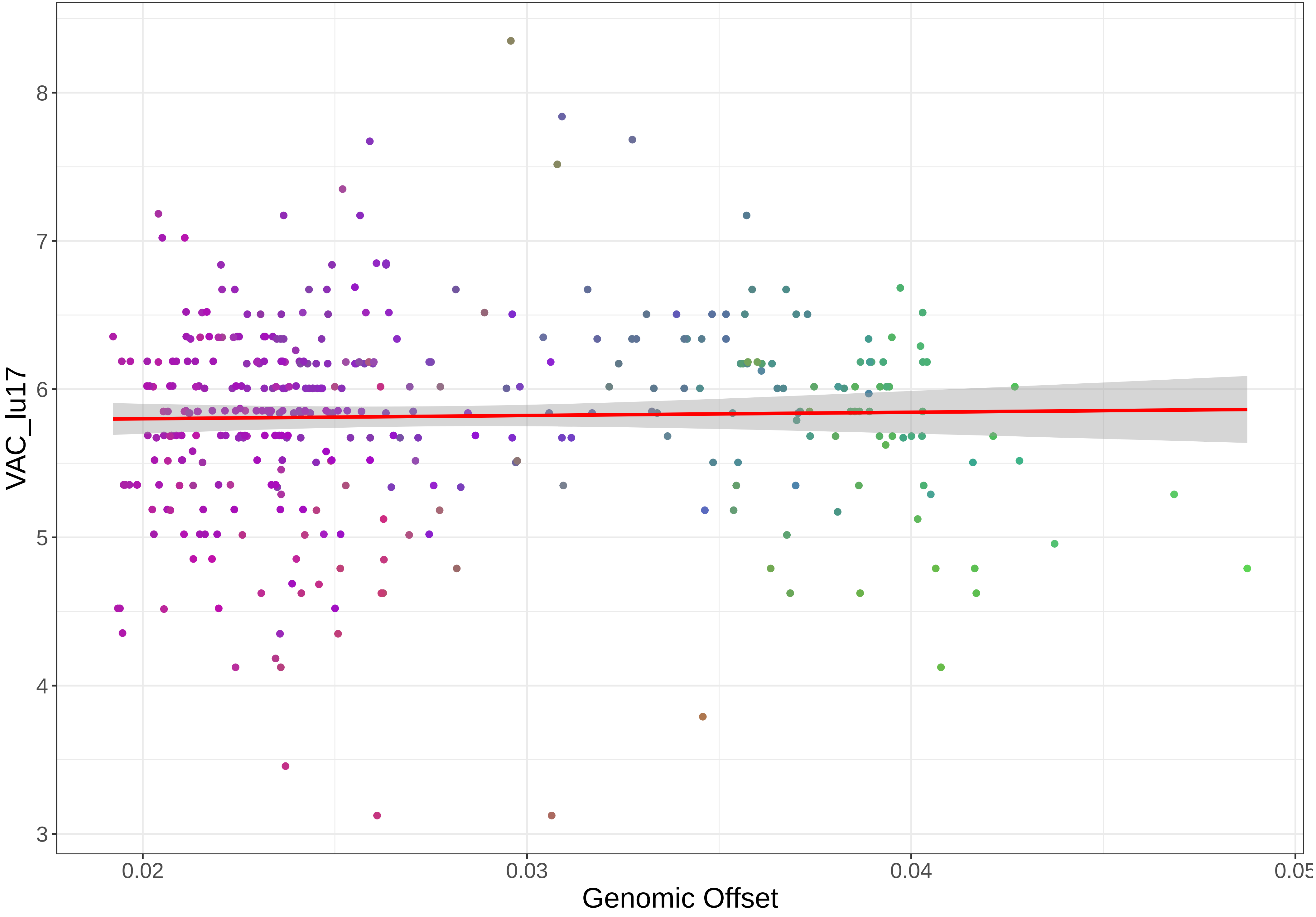

8
7
6
5
4
3
VAC_lu17
0.02
0.03
0.04
0.05
Genomic Offset

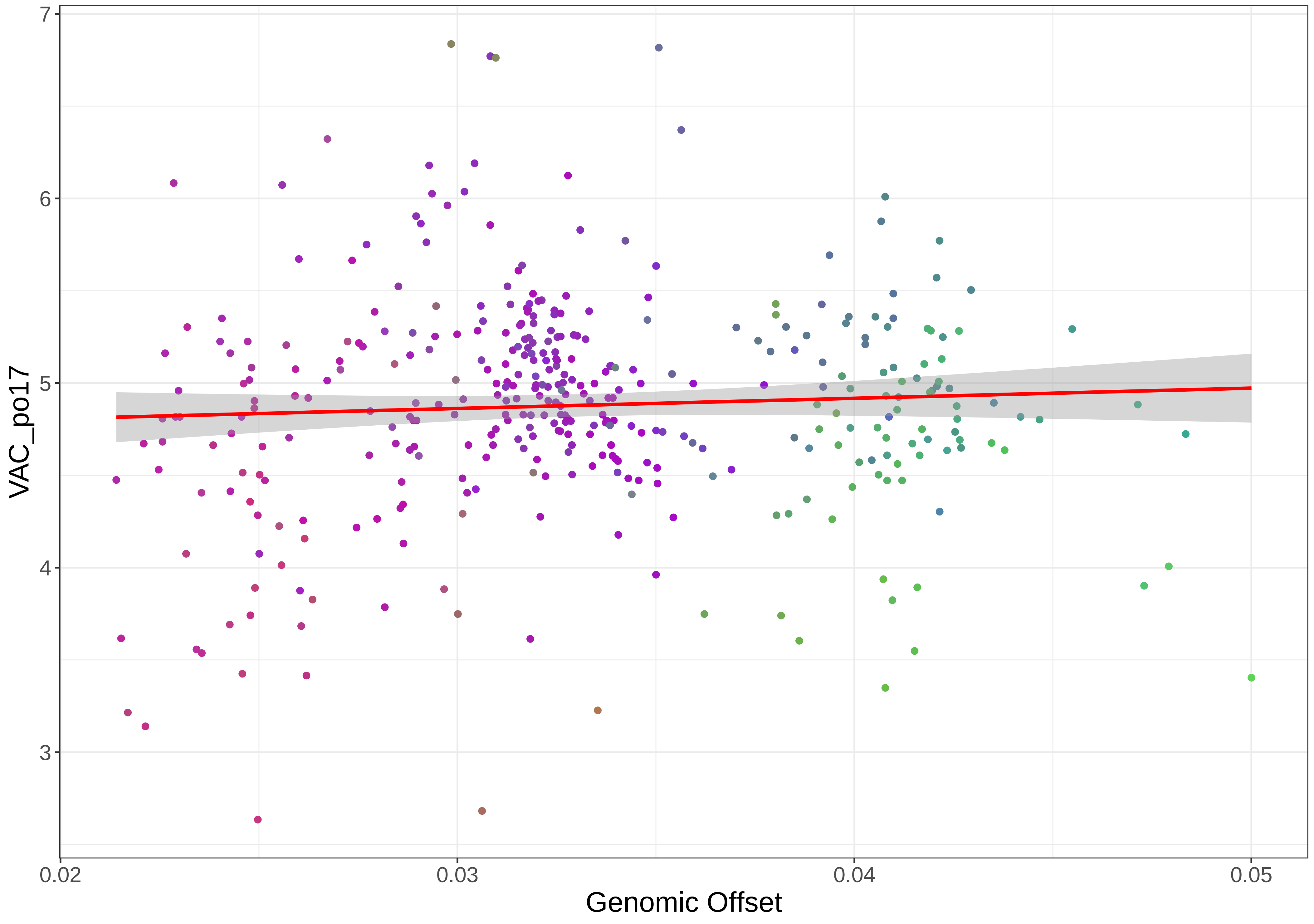
7
6
5
4
3
VAC_po17
0.02
0.03
0.04
0.05
Genomic Offset

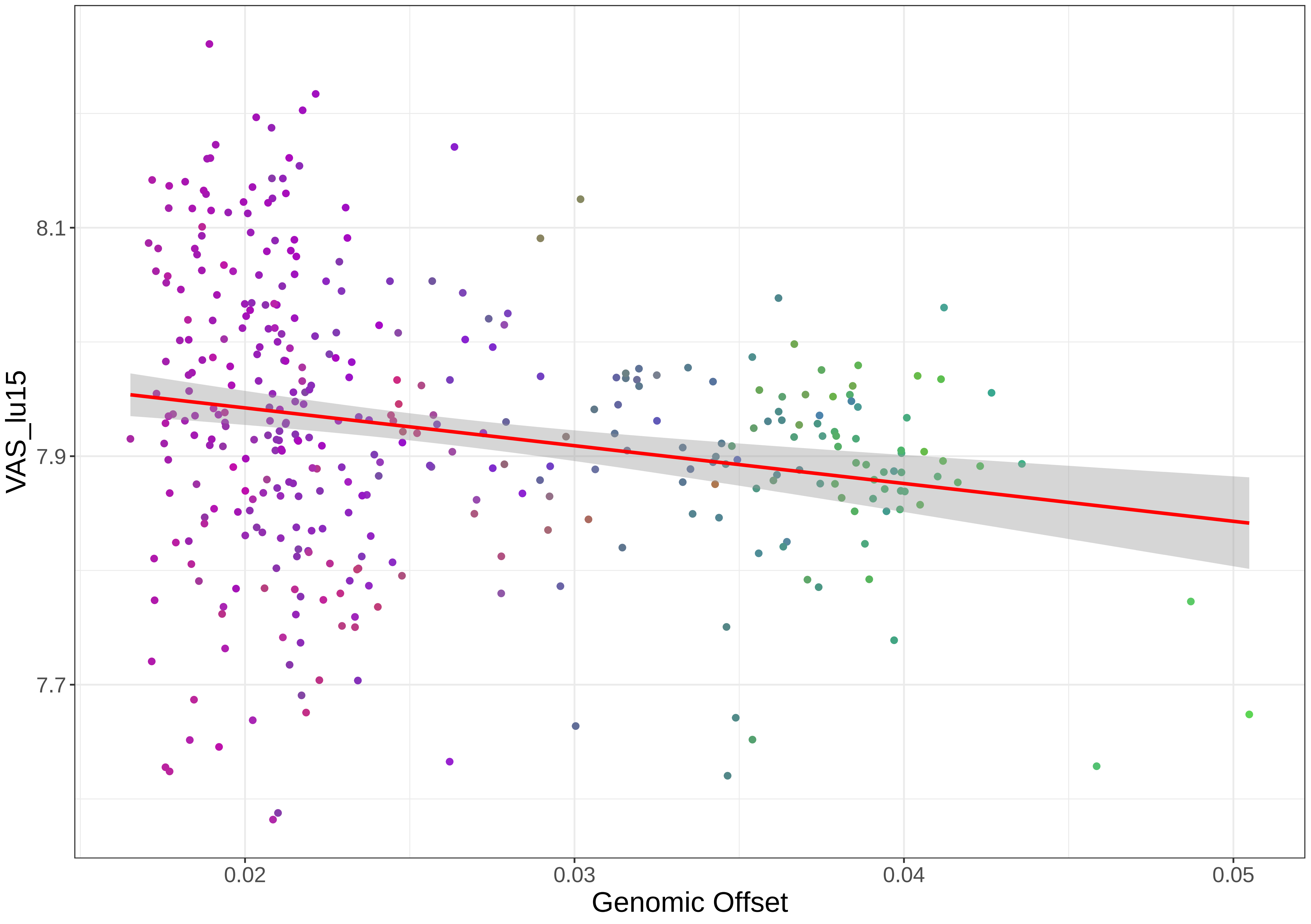

VAS_lu15
8.1
7.9
7.7
0.02
0.03
0.04
0.05
Genomic Offset

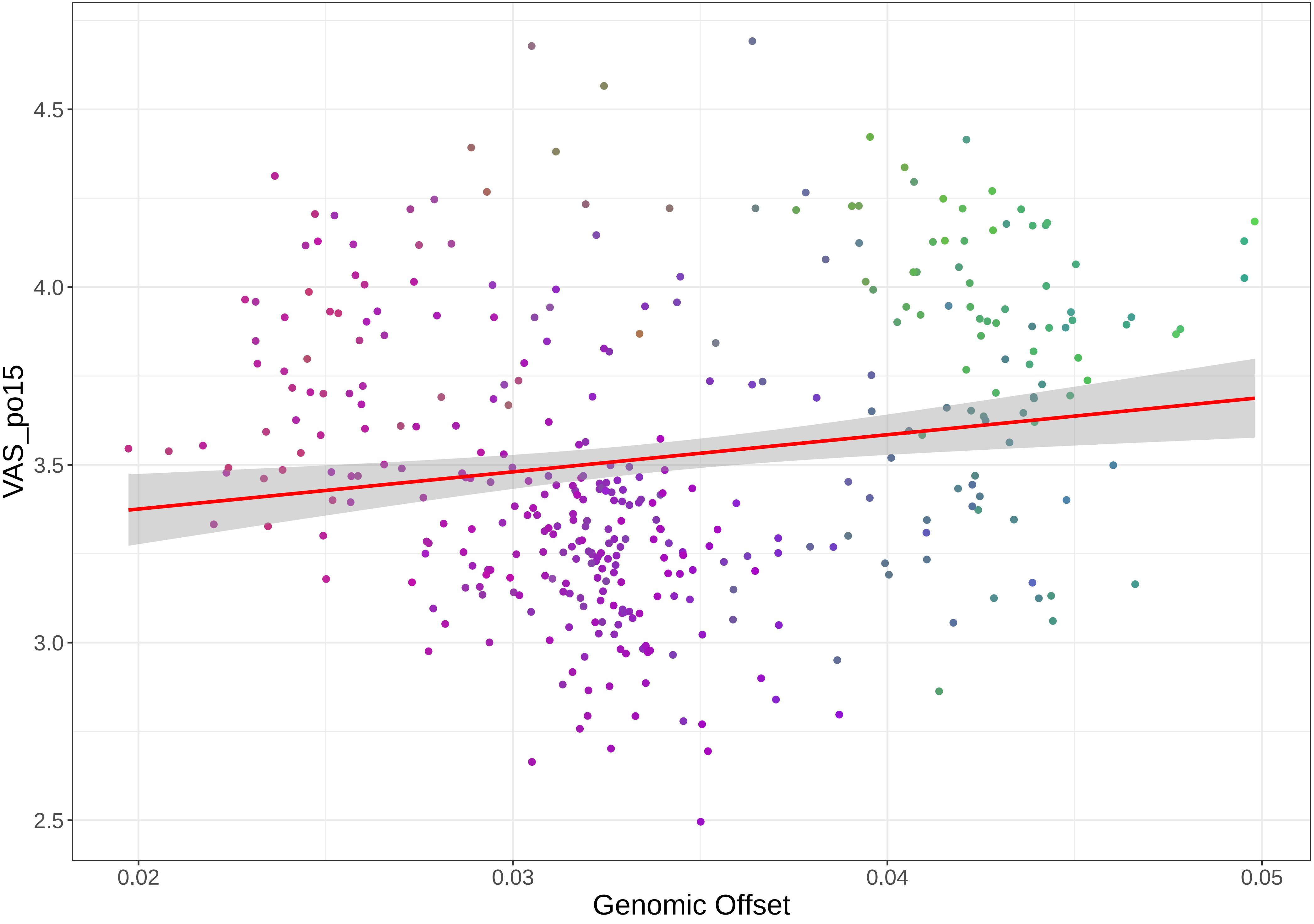
4.5
4.0
3.5
3.0
2.5
VAS_po15
0.02
0.03
0.04
0.05
Genomic Offset

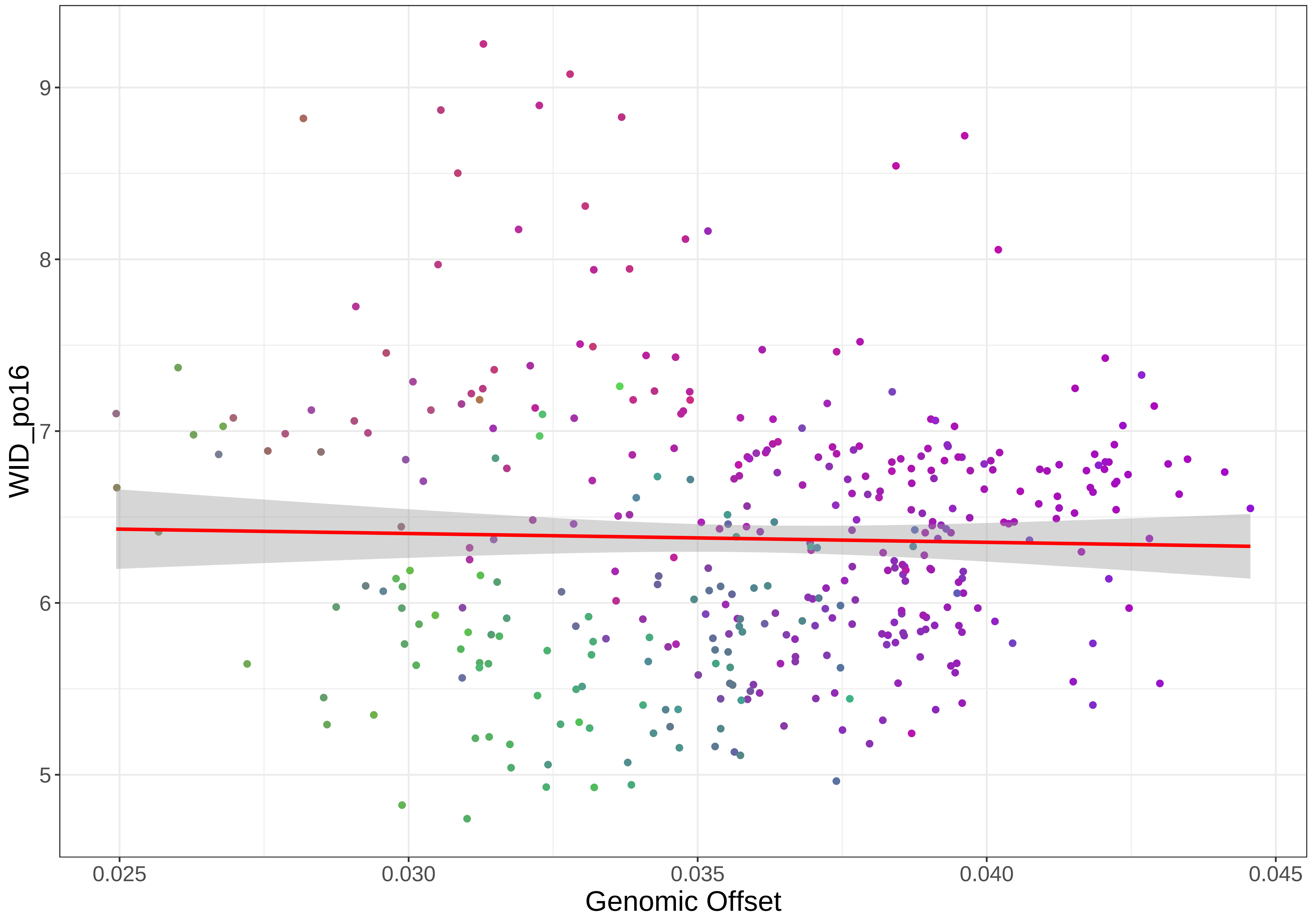
WID_po16
9
8
7
6
5
0.025
0.030
0.035
0.040
0.045
Genomic Offset

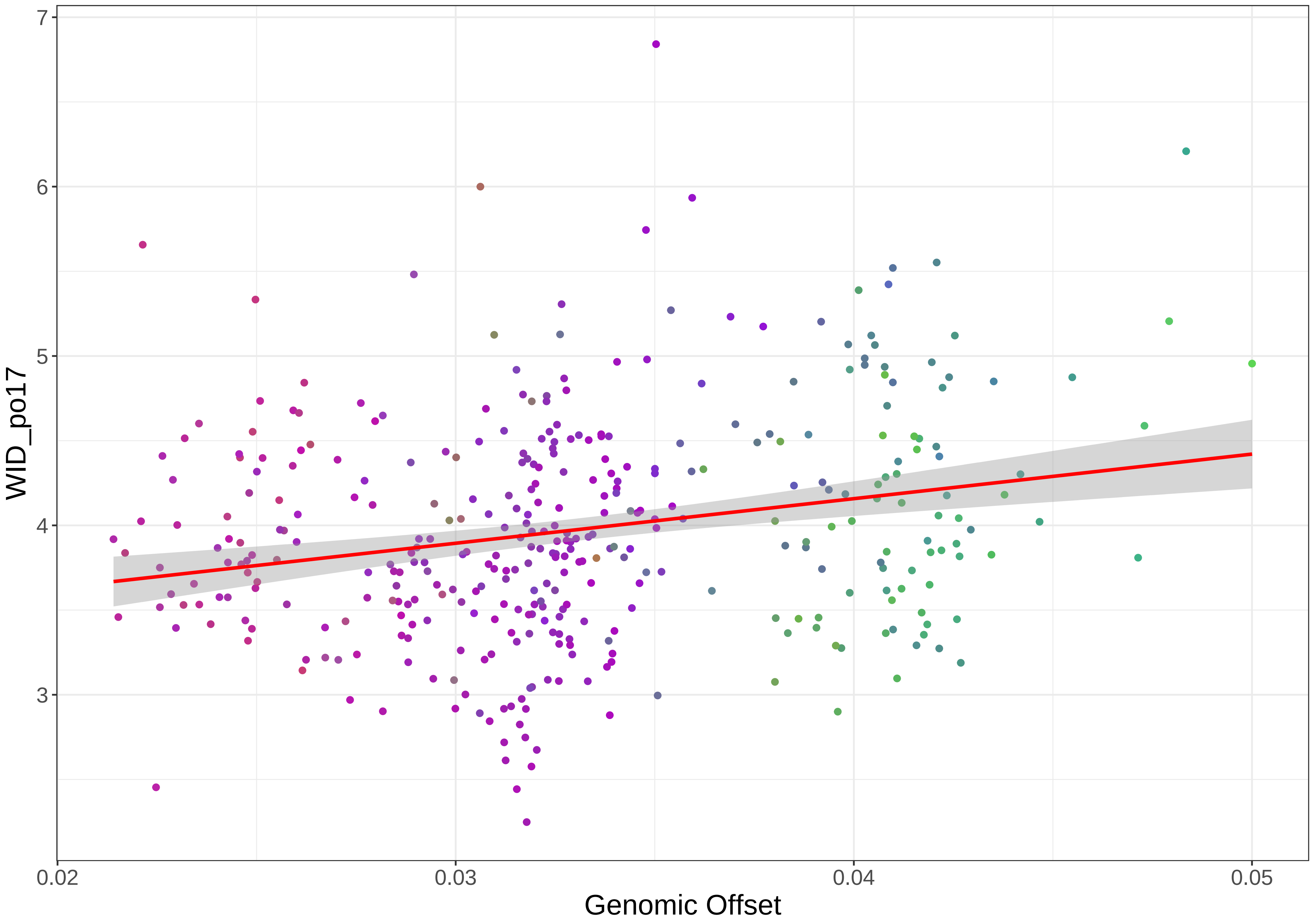
WID_po17
7
6
5
4
3
0.02
0.03
0.04
0.05
Genomic Offset

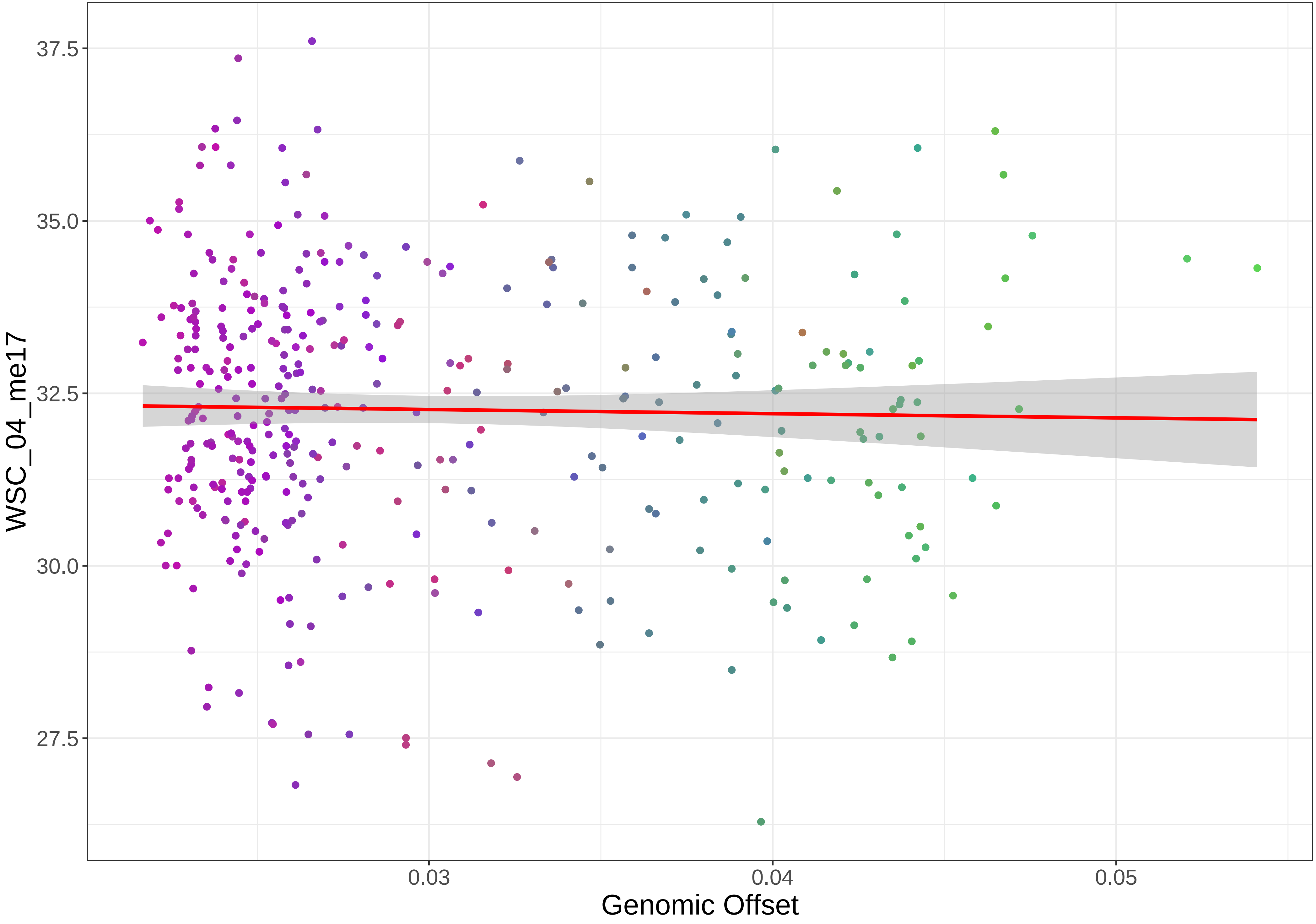

37.5
35.0
32.5
30.0
27.5
WSC_04_me17
0.03
0.04
0.05
Genomic Offset

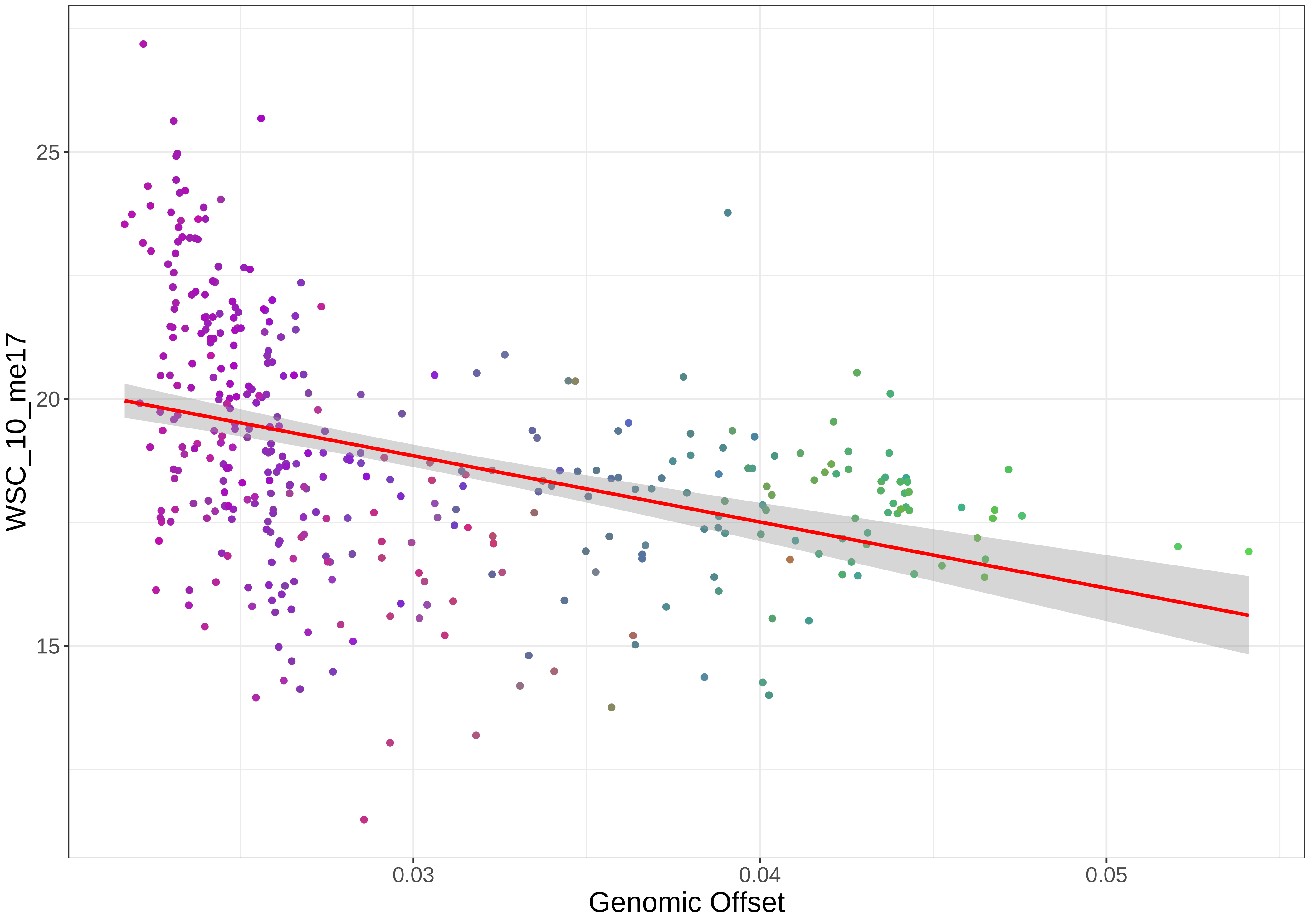

25
20
15
WSC_10_me17
0.03
0.04
0.05
Genomic Offset
