# Supplementary material for: How Robust Are Genomic Offset Predictions to Methodological Choices? Insights From Perennial Ryegrass
Source: Mol Ecol. 2026 Jul 13;35(14):e70463. doi: 10.1111/mec.70463 (PMC13361167; doi:10.1111/mec.70463)
Supplement: Supplementary file 3 — Figure S2: Relationship between phenotypic trait values measured in the common gardens and the genomic offset experienced at each site during the growth period preceding trait measurement, based on the GFCANCOR model. Each panel corresponds to a single phenotypic trait, with the trait label (e.g., ADF_04_me17) indicated on the y‐axis; the trait name encodes the trait, the measurement period, and the common garden and year of measurement. Each point represents one population, coloured according to the adaptive genetic composition of its location of origin (as in Figure 3, derived from the first three principal components of the GF‐transformed environmental variables mapped onto an RGB colour palette). The red line shows the linear regression of the trait on genomic offset, and the grey band its 95% confidence interval. [file MEC-35-e70463-s012.pdf]

Figure S2 : Relationship between phenotypic trait values measured in the common gardens and the genomic offset experienced at each site during the growth period preceding trait measurement, based on the GF_{CANCOR} model. Each panel corresponds to a single phenotypic trait, with the trait label (e.g., ADF_04_me17) indicated on the y-axis; the trait name encodes the trait, the measurement period, and the common garden and year of measurement. Each point represents one population, coloured according to the adaptive genetic composition of its location of origin (as in Figure 3, derived from the first three principal components of the GF-transformed environmental variables mapped onto an RGB colour palette). The red line shows the linear regression of the trait on genomic offset, and the grey band its 95% confidence interval.

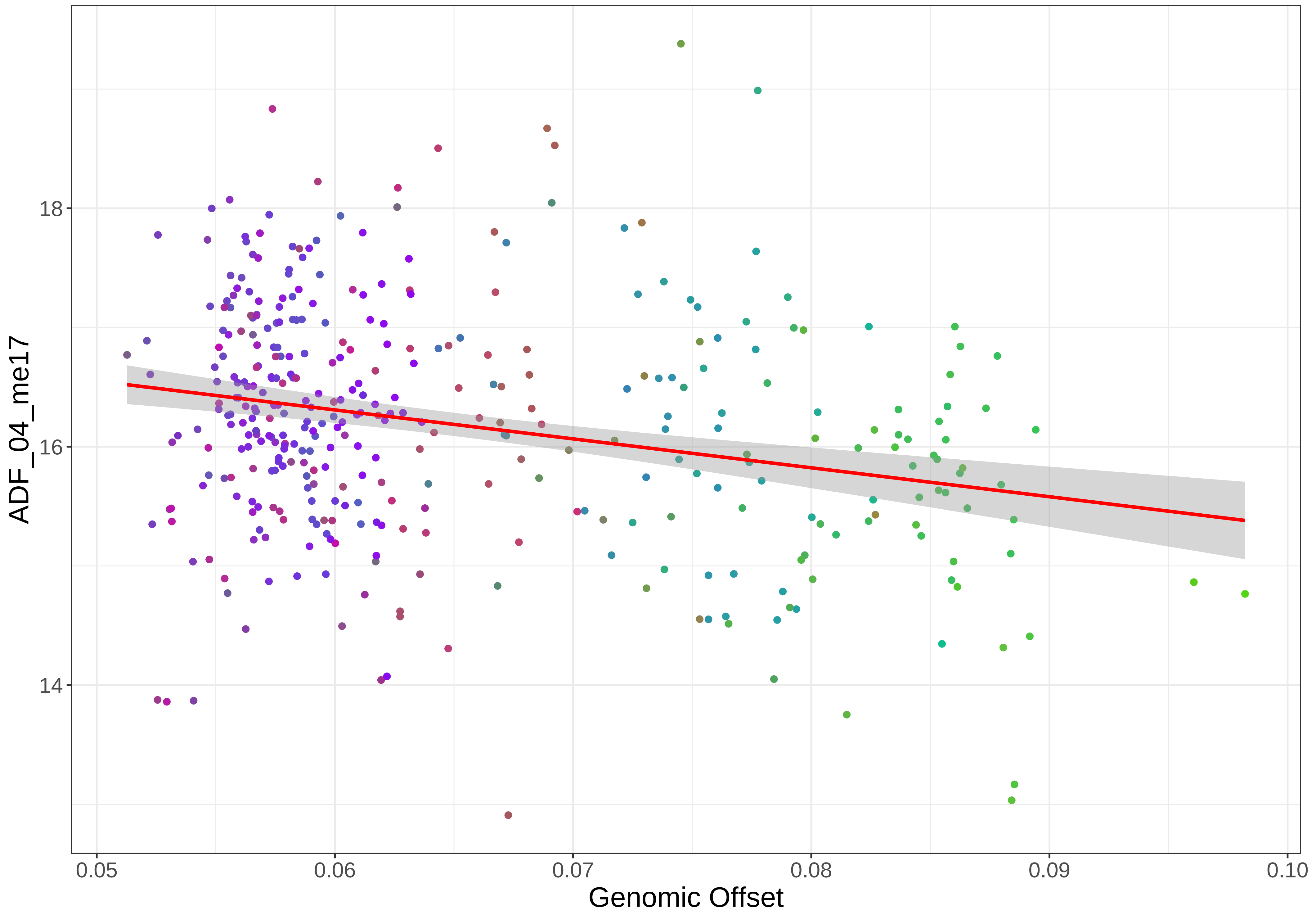

ADF_04_me17
18
16
14
0.05
0.06
0.07
0.08
0.09
0.10
Genomic Offset

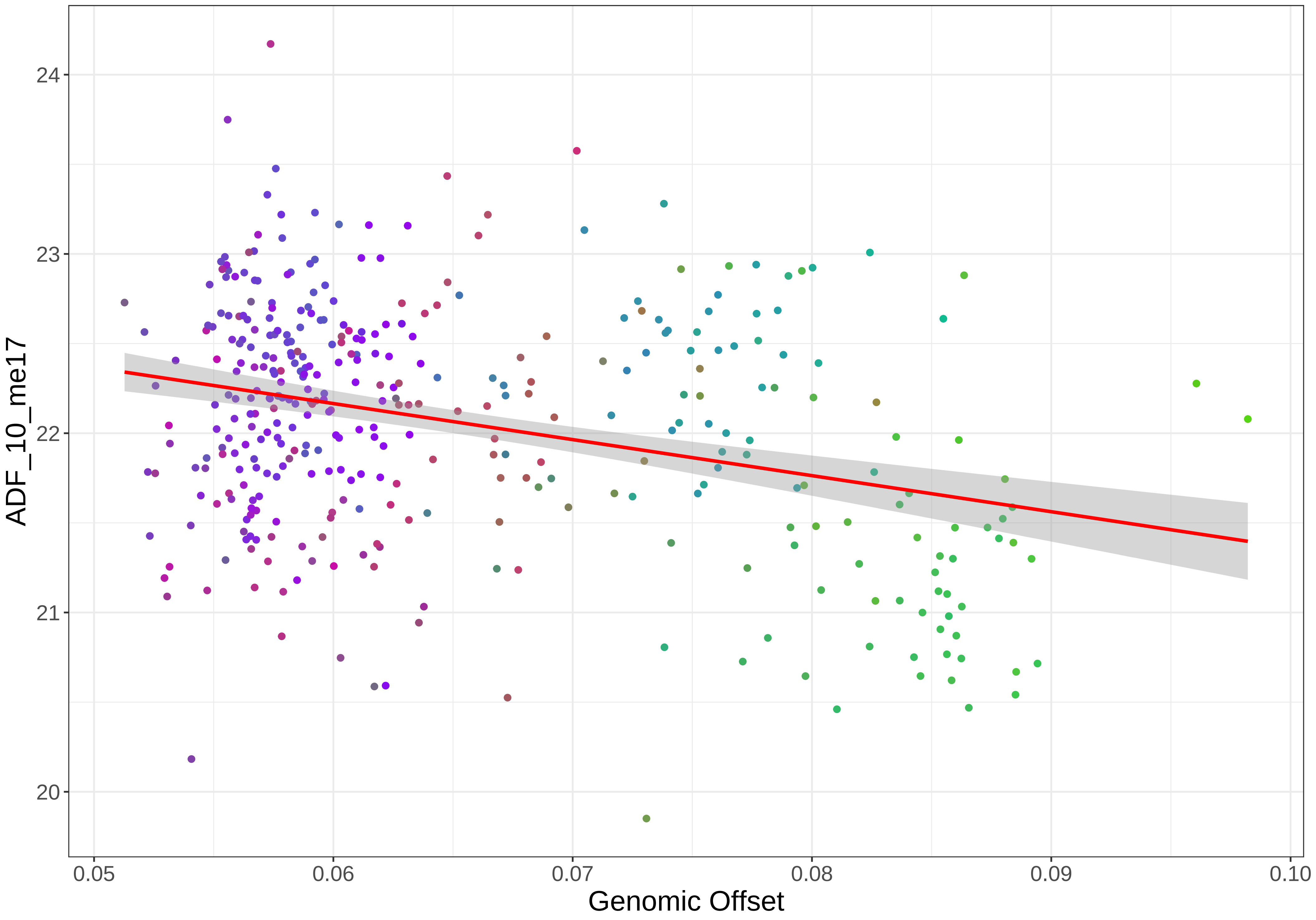

ADF_10_me17
24
23
22
21
20
0.05
0.06
0.07
0.08
0.09
0.10
Genomic Offset

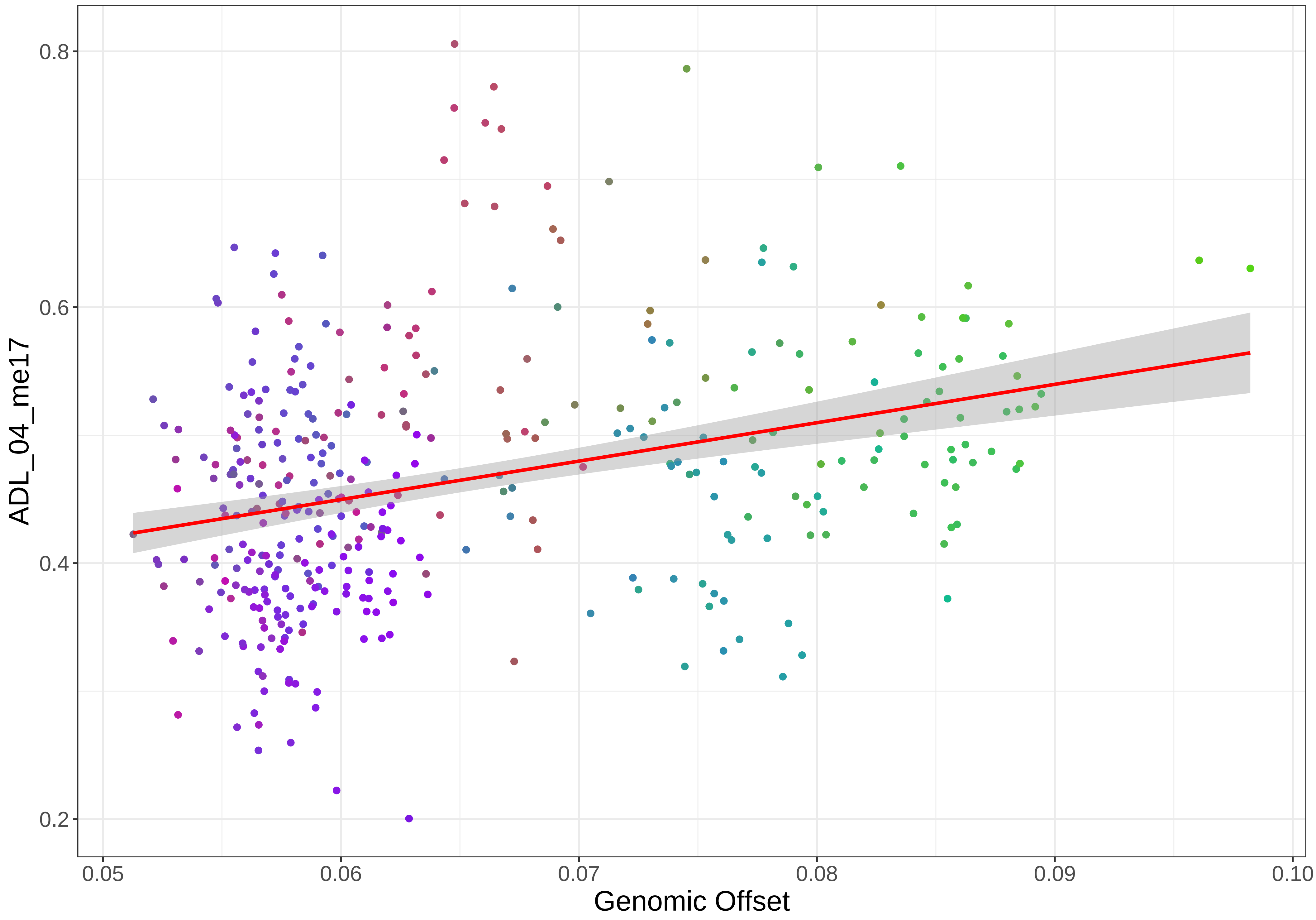

0.8
0.6
0.4
0.2
ADL_04_me17
0.05
0.06
0.07
0.08
0.09
0.10
Genomic Offset

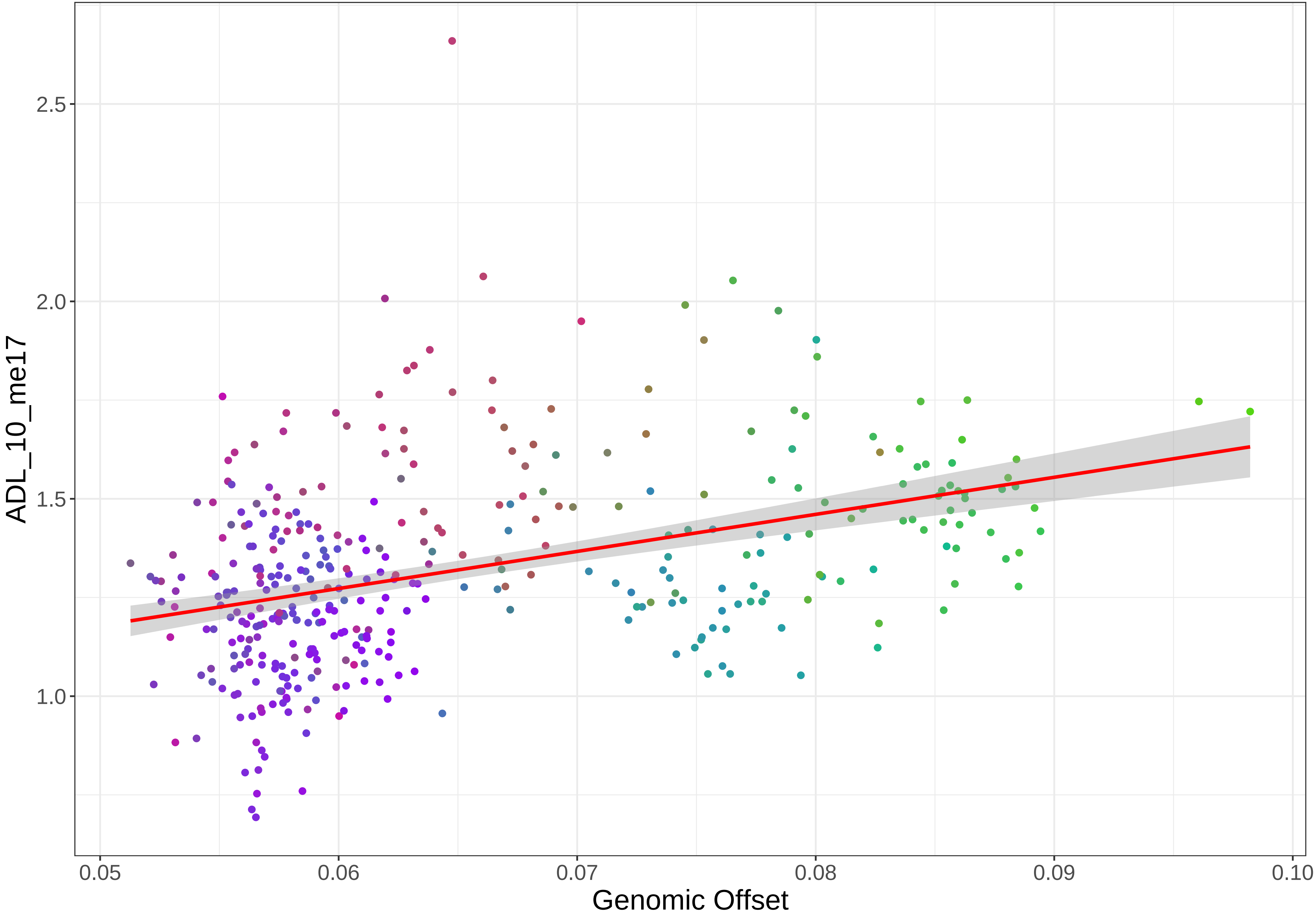

ADL_10_me17
2.5
2.0
1.5
1.0
0.05
0.06
0.07
0.08
0.09
0.10
Genomic Offset

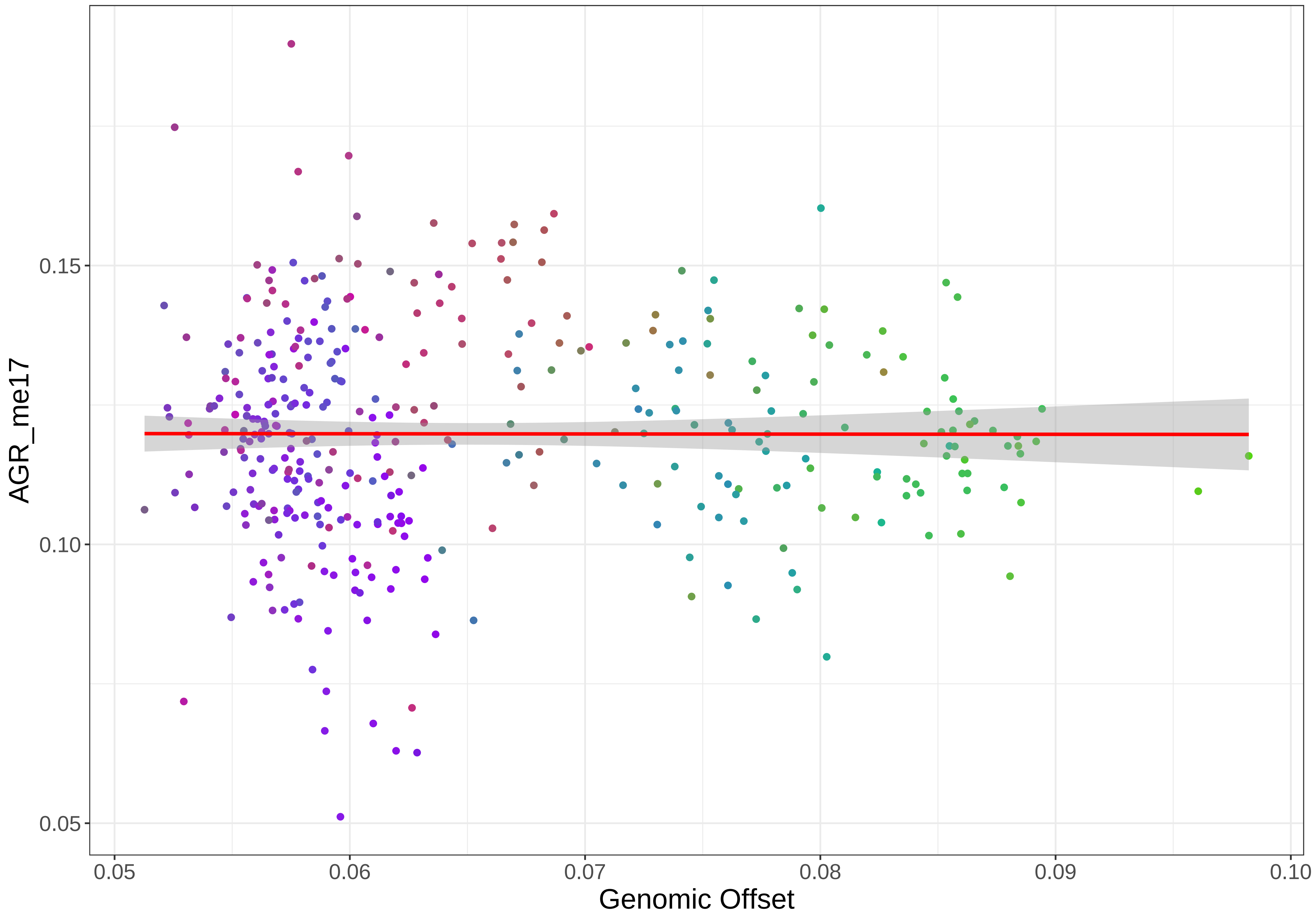

AGR_me17
0.15
0.10
0.05
0.05
0.06
0.07
0.08
0.09
0.10
Genomic Offset

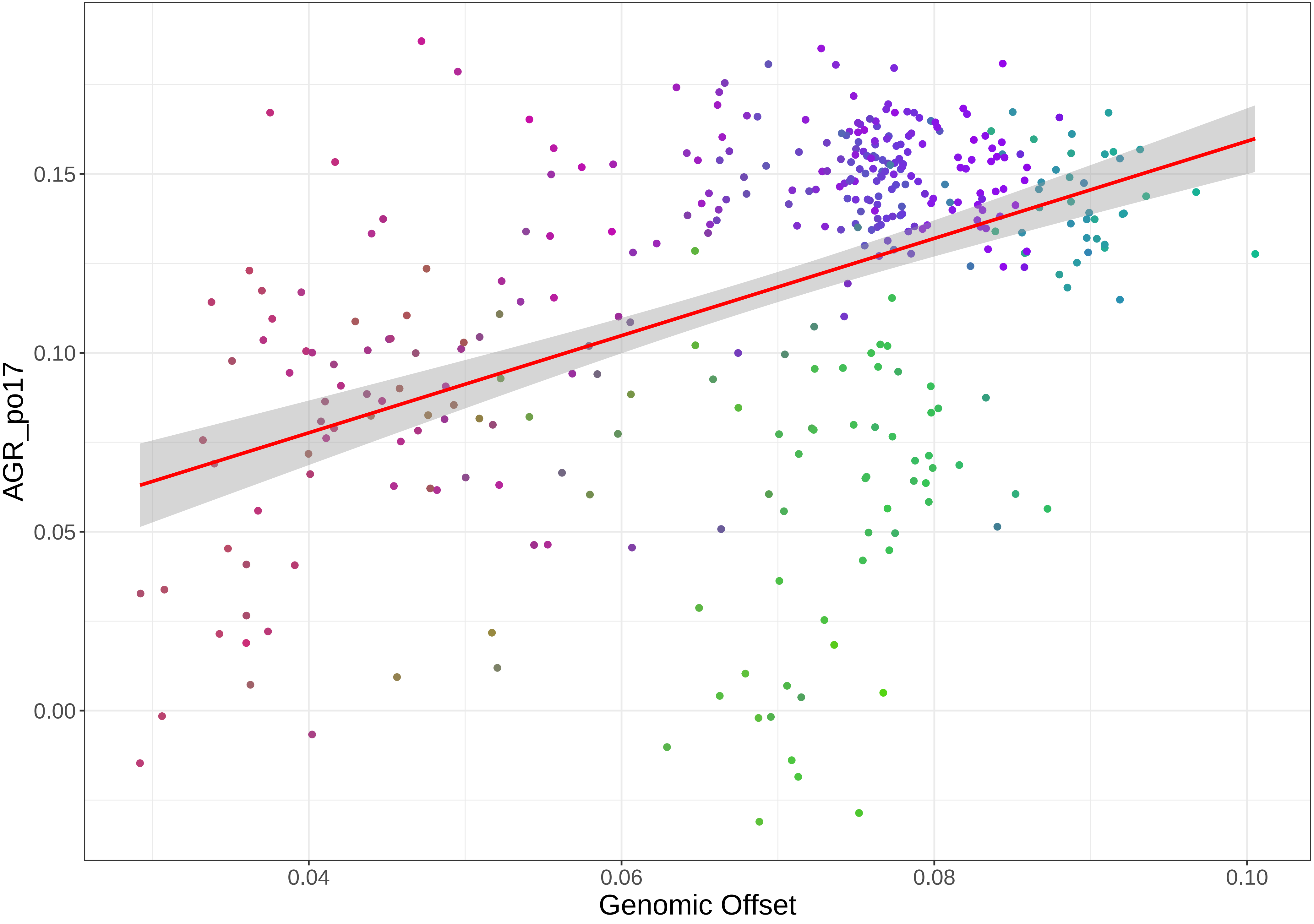

AGR_po17
0.15
0.10
0.05
0.00
0.04
0.06
0.08
0.10
Genomic Offset

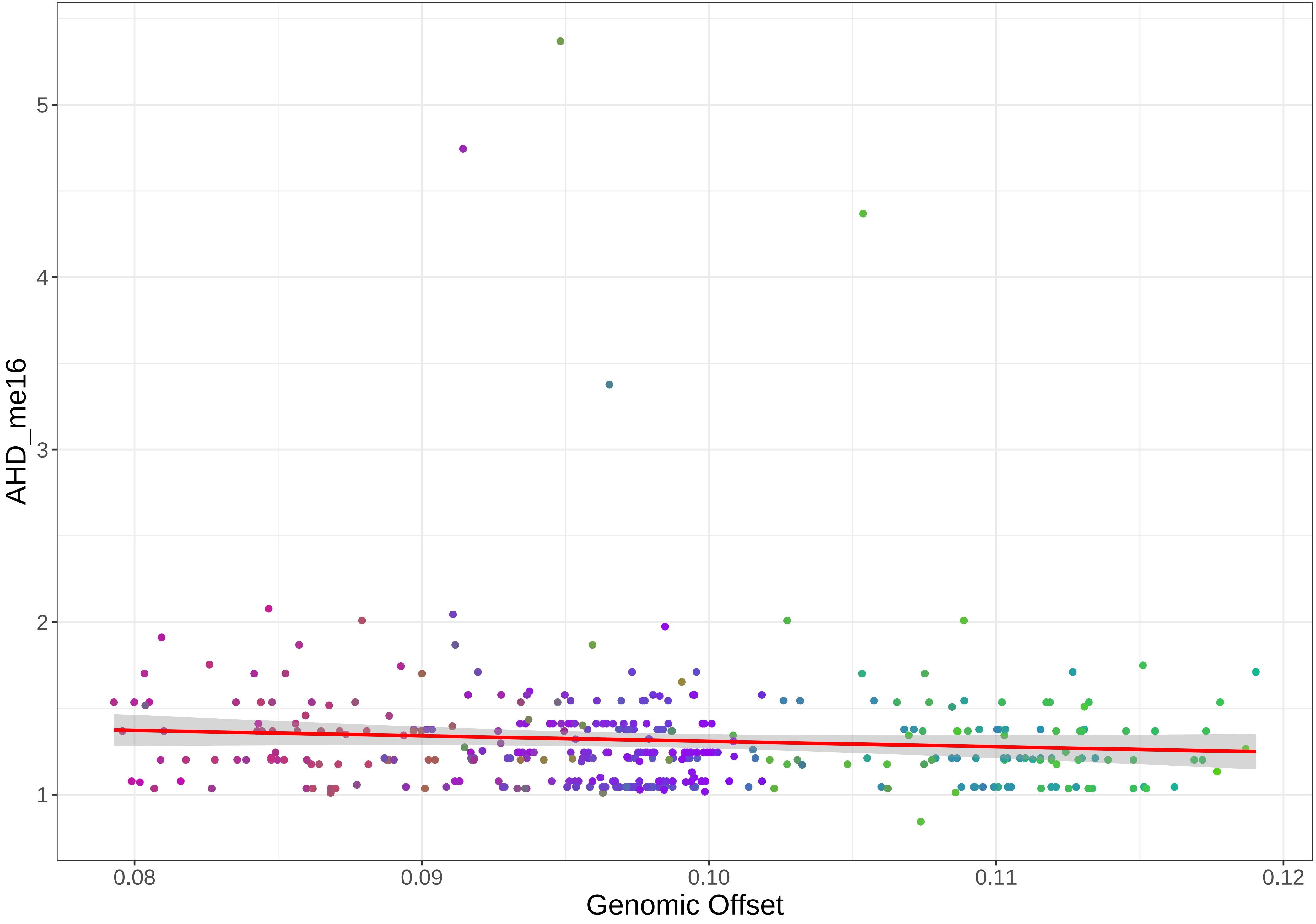

AHD_me16
1
2
3
4
5
0.08
0.09
0.10
0.11
0.12
Genomic Offset

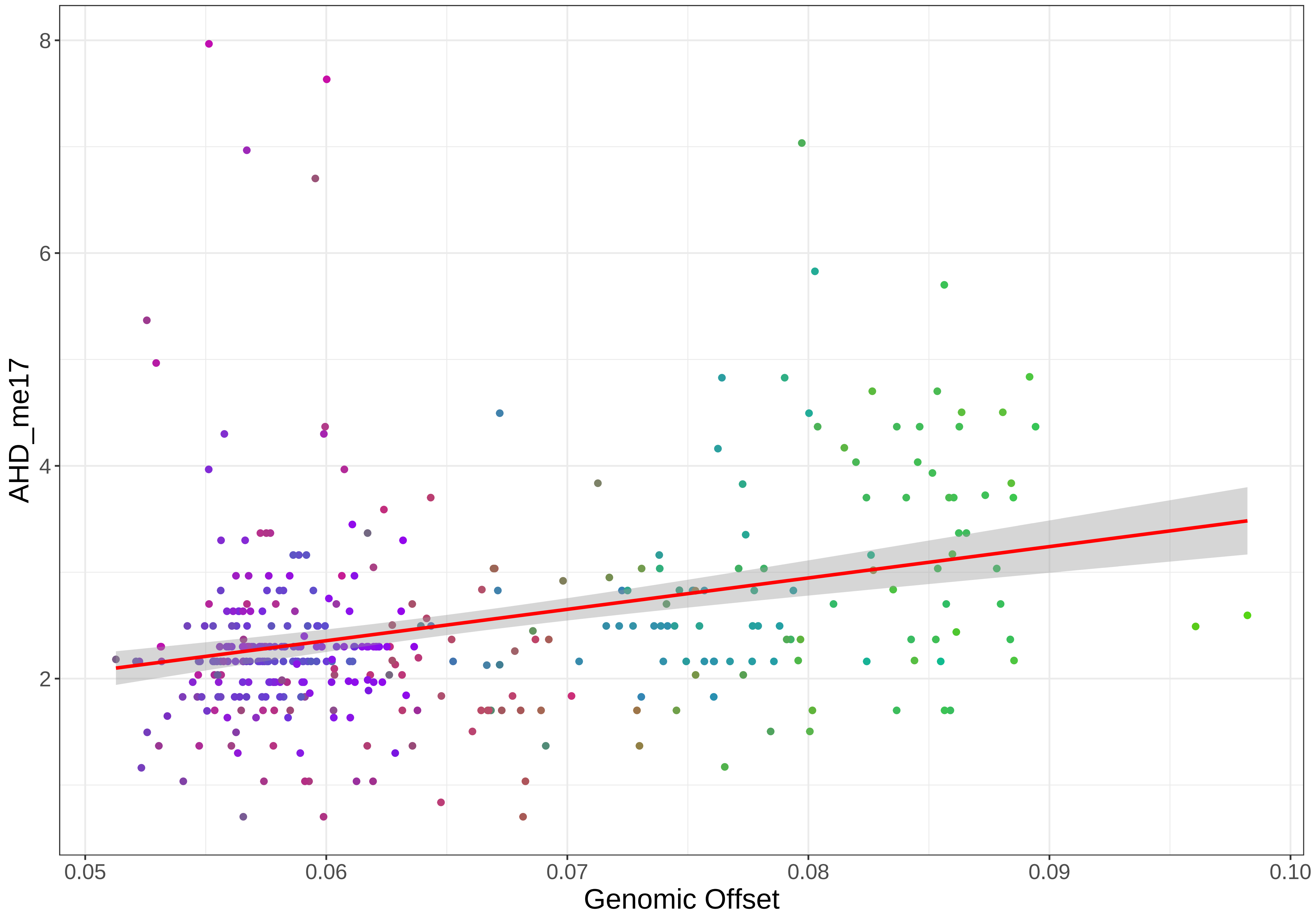

AHD_me17
8
6
4
2
0.05
0.06
0.07
0.08
0.09
0.10
Genomic Offset

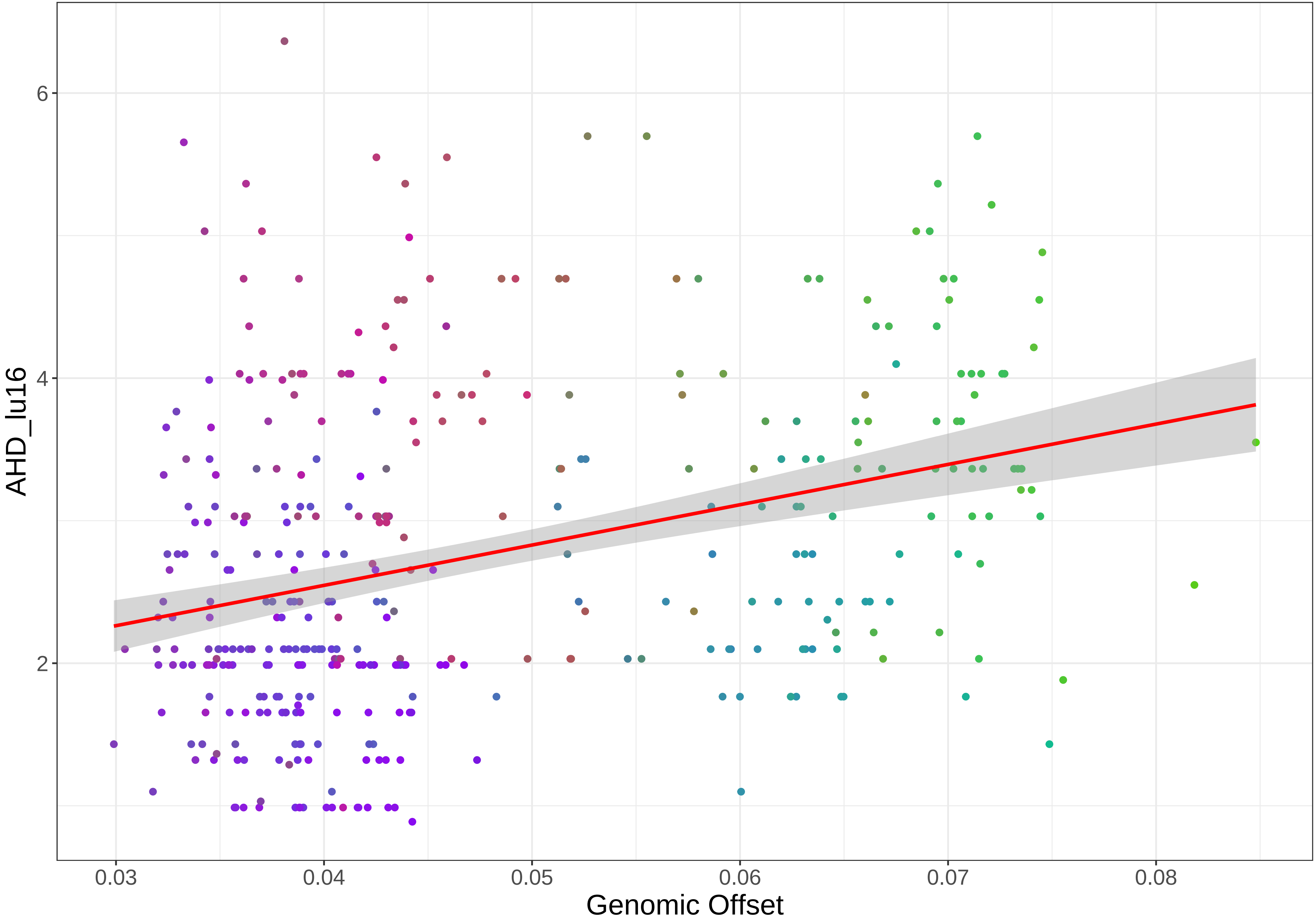

AHD_lu16
6
4
2
0.03
0.04
0.05
0.06
0.07
0.08
Genomic Offset

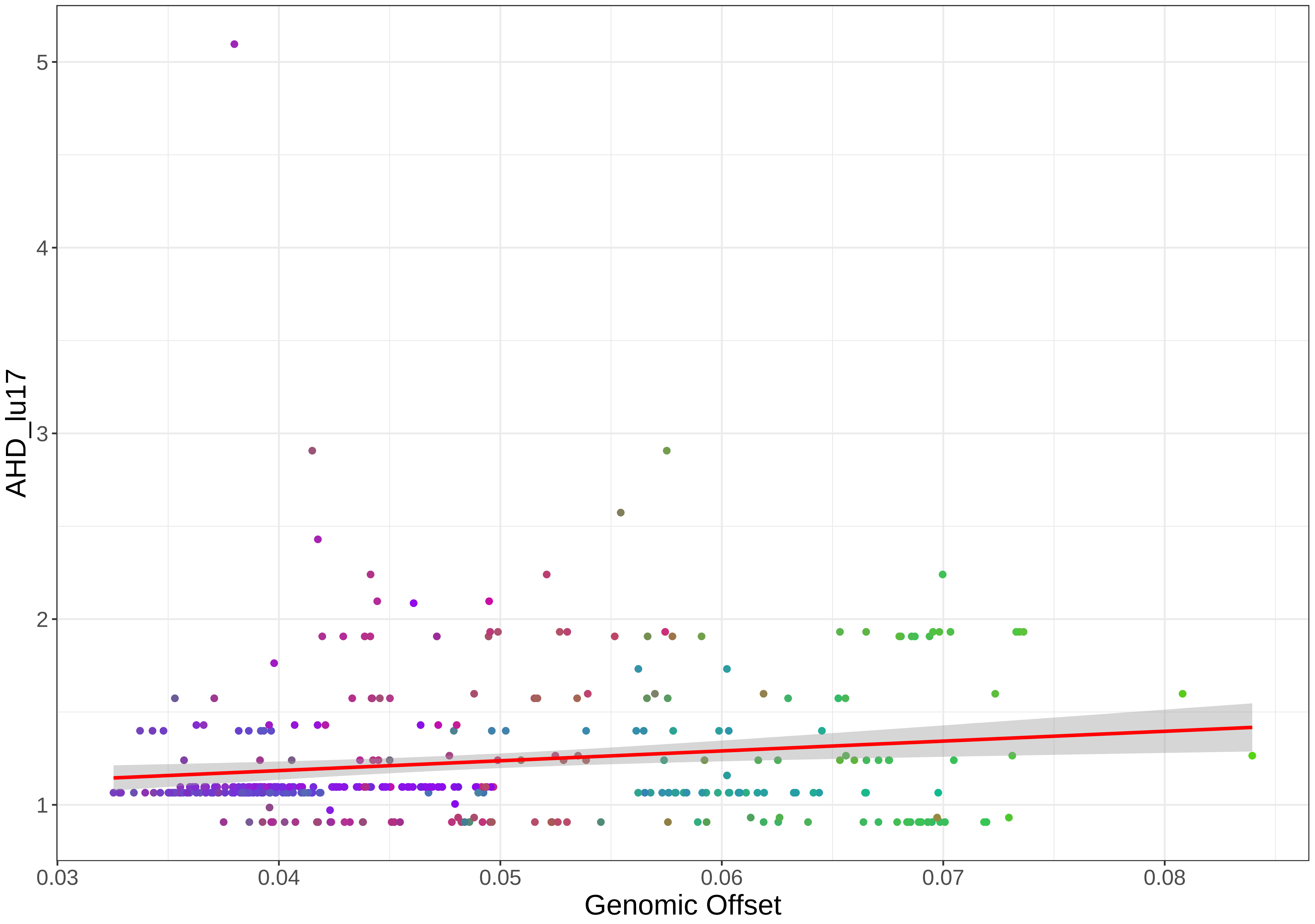
AHD_lu17
5
4
3
2
1
0.03
0.04
0.05
0.06
0.07
0.08
Genomic Offset

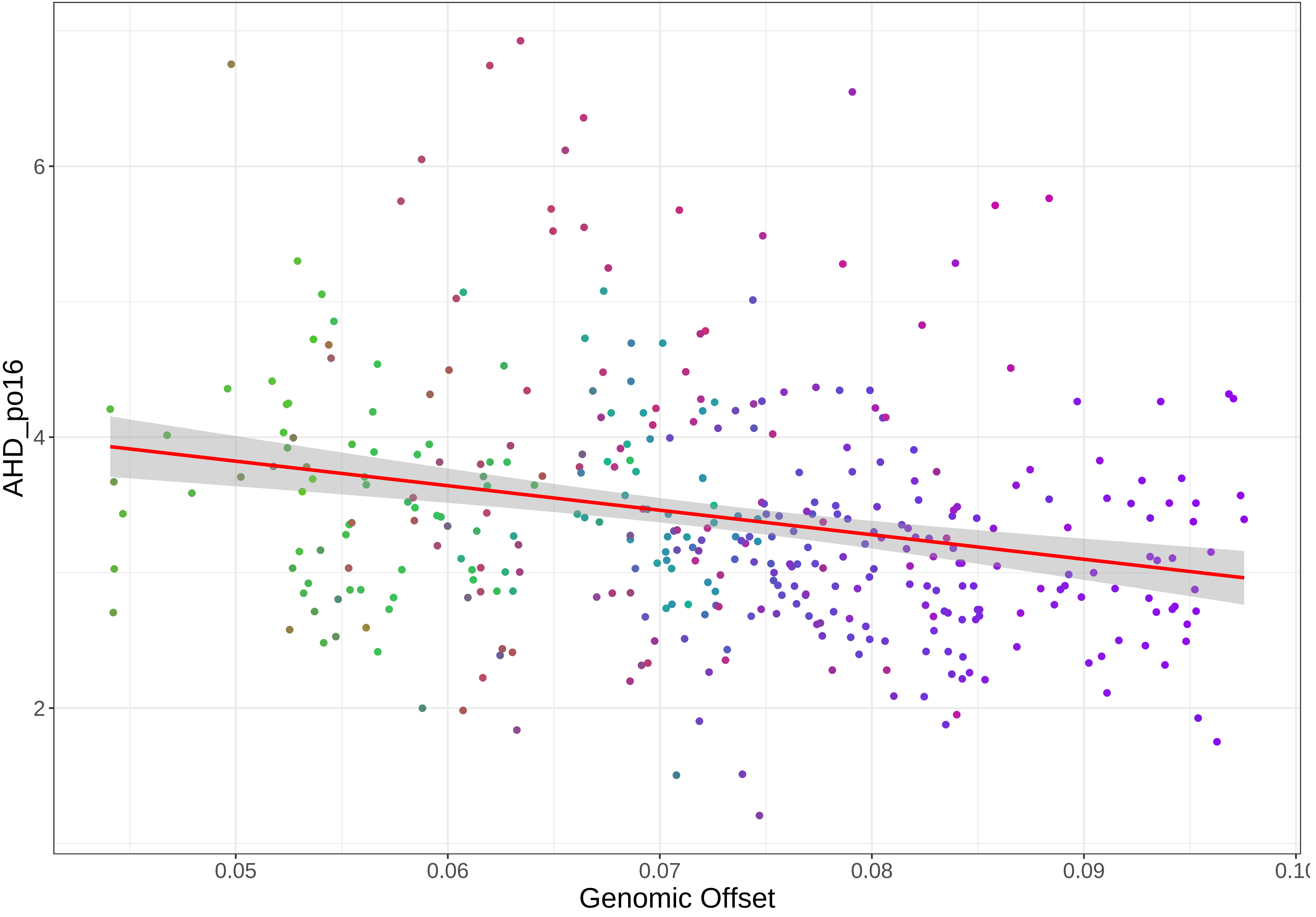

AHD_po16
6
4
2
0.05
0.06
0.07
0.08
0.09
0.10
Genomic Offset

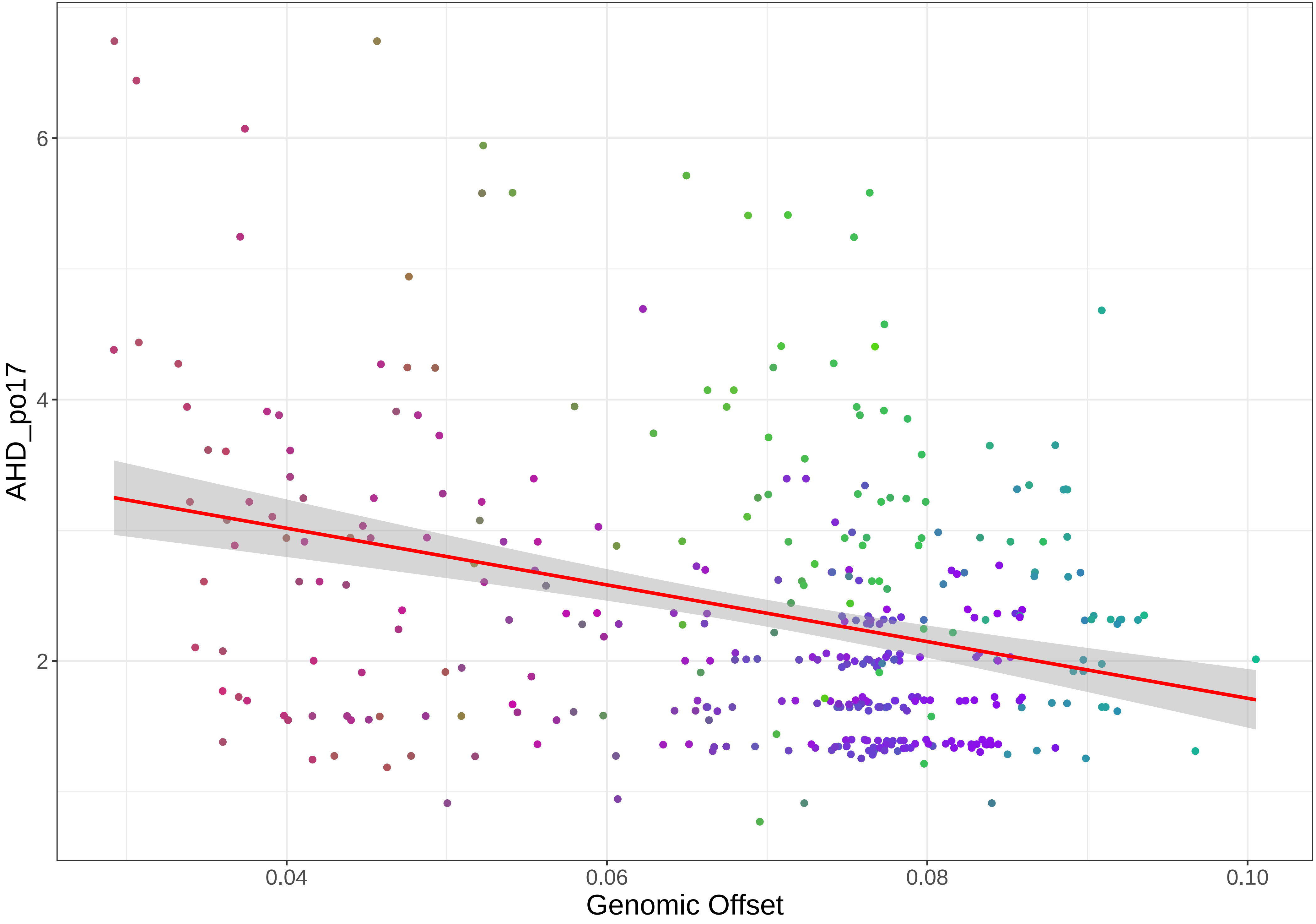

AHD_po17
6
4
2
0.04
0.06
0.08
0.10
Genomic Offset

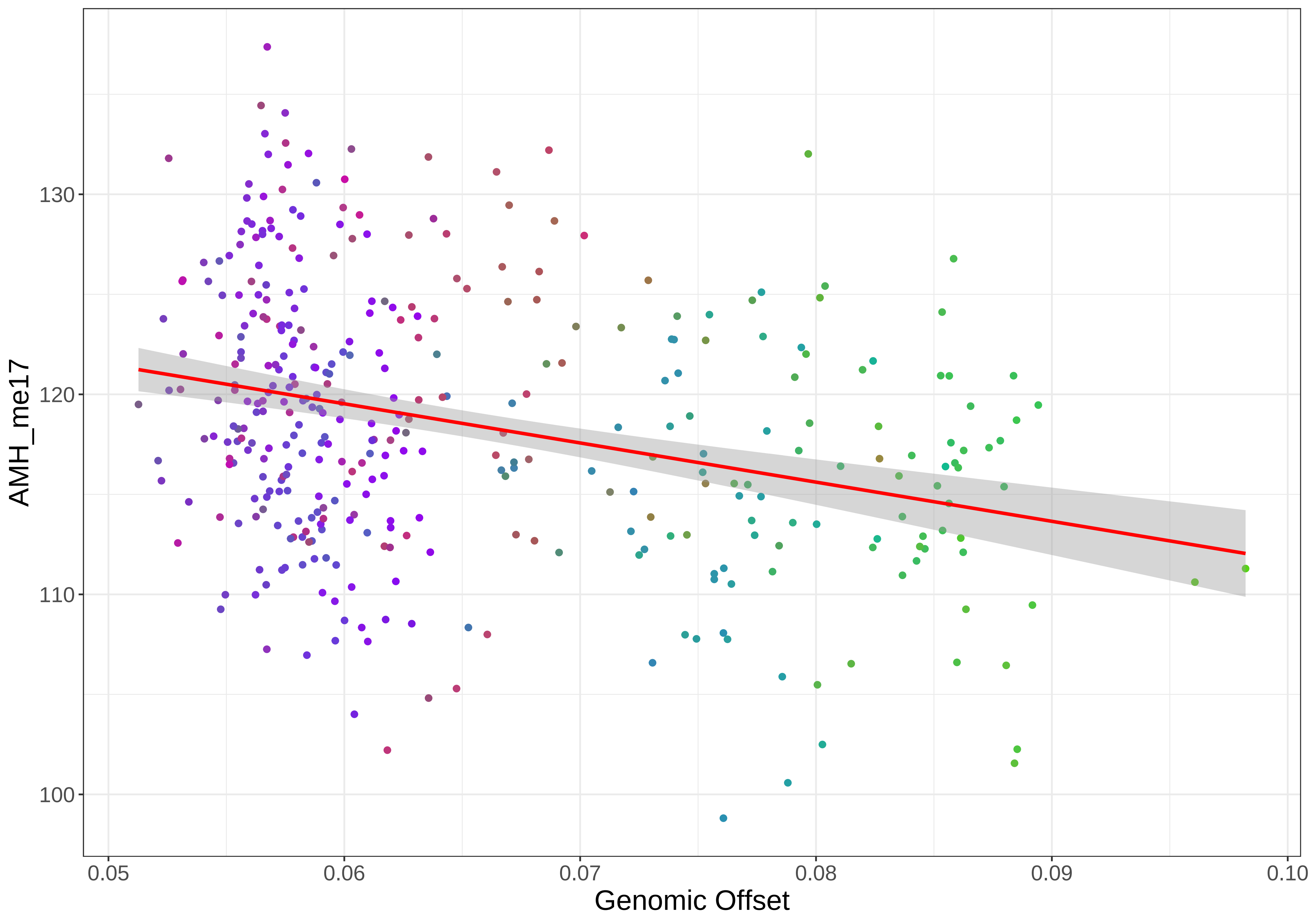
AMH_me17
130
120
110
100
0.05
0.06
0.07
0.08
0.09
0.10
Genomic Offset

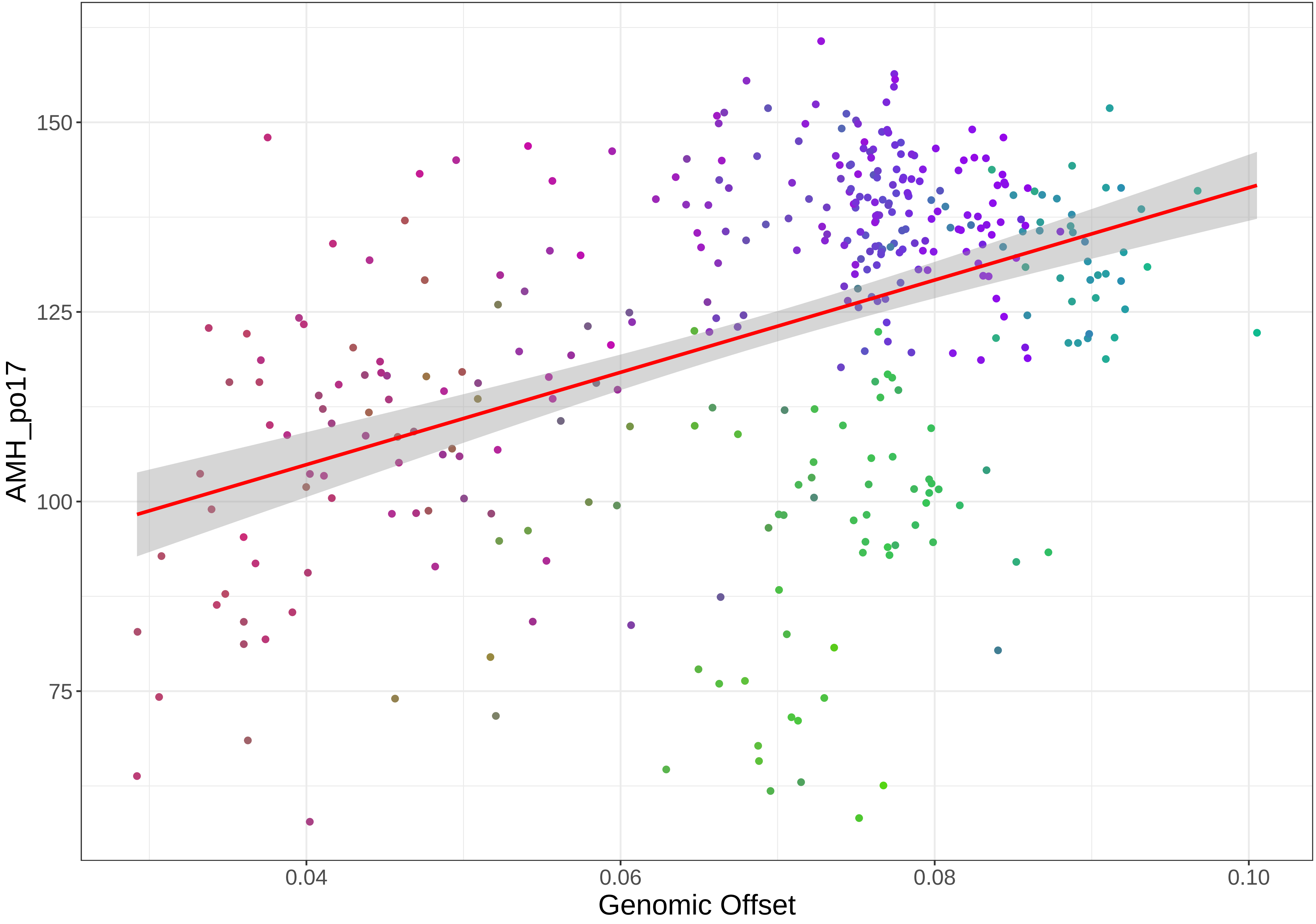
AMH_po17
150
125
100
75
0.04
0.06
0.08
0.10
Genomic Offset

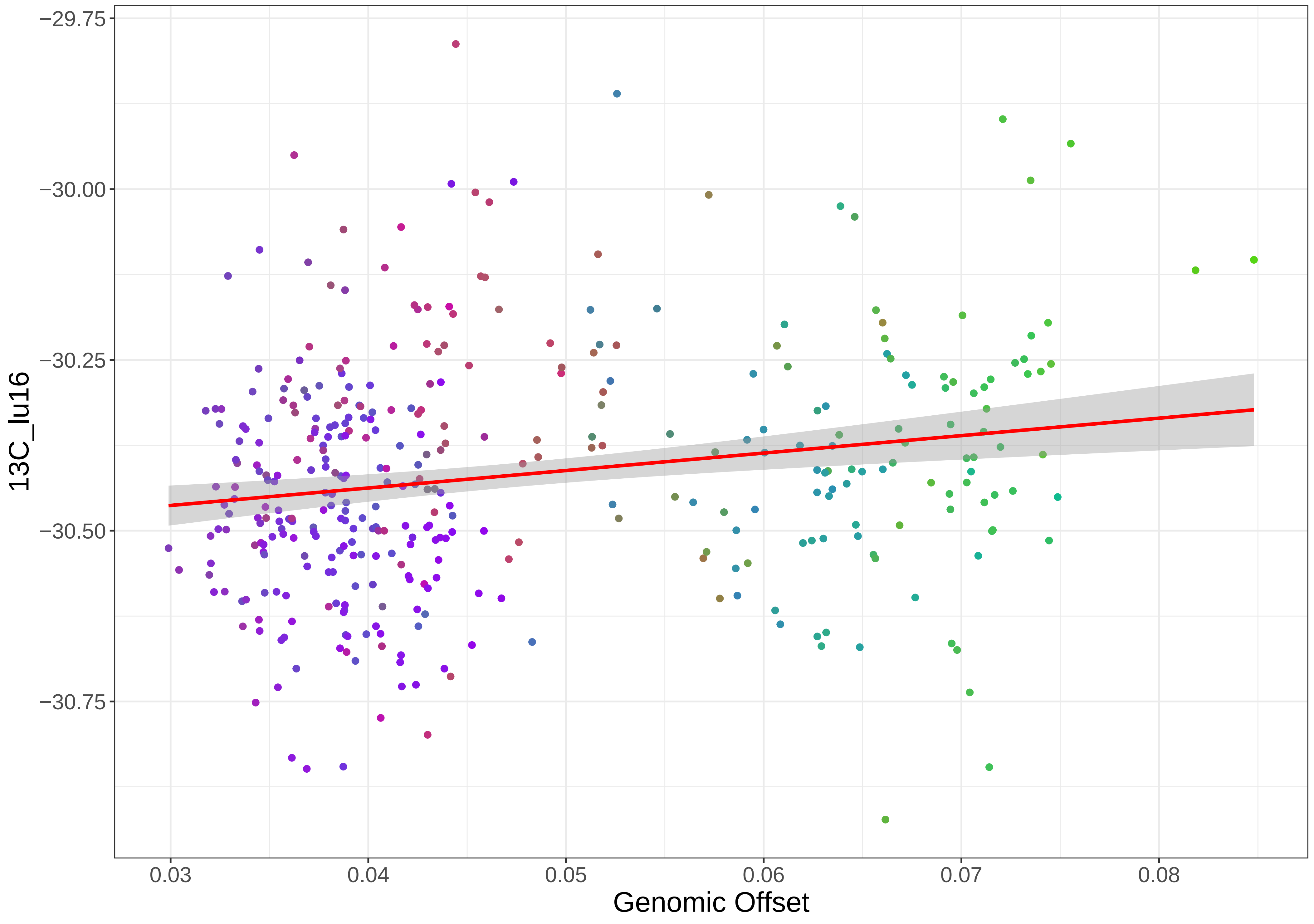
−29.75
−30.00
−30.25
−30.50
−30.75
13C_lu16
0.03
0.04
0.05
0.06
0.07
0.08
Genomic Offset

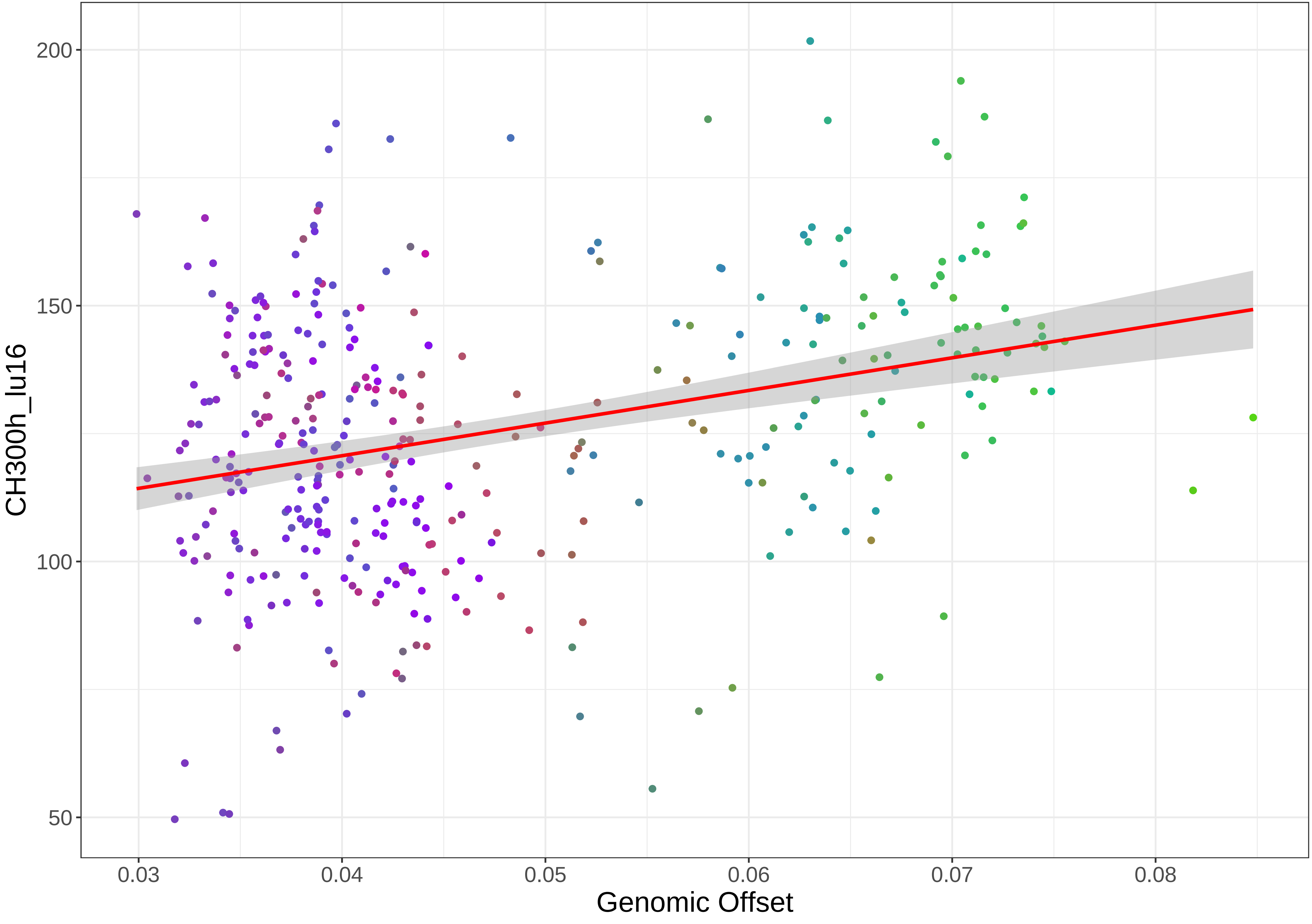

CH300h_lu16
200
150
100
50
0.03
0.04
0.05
0.06
0.07
0.08
Genomic Offset

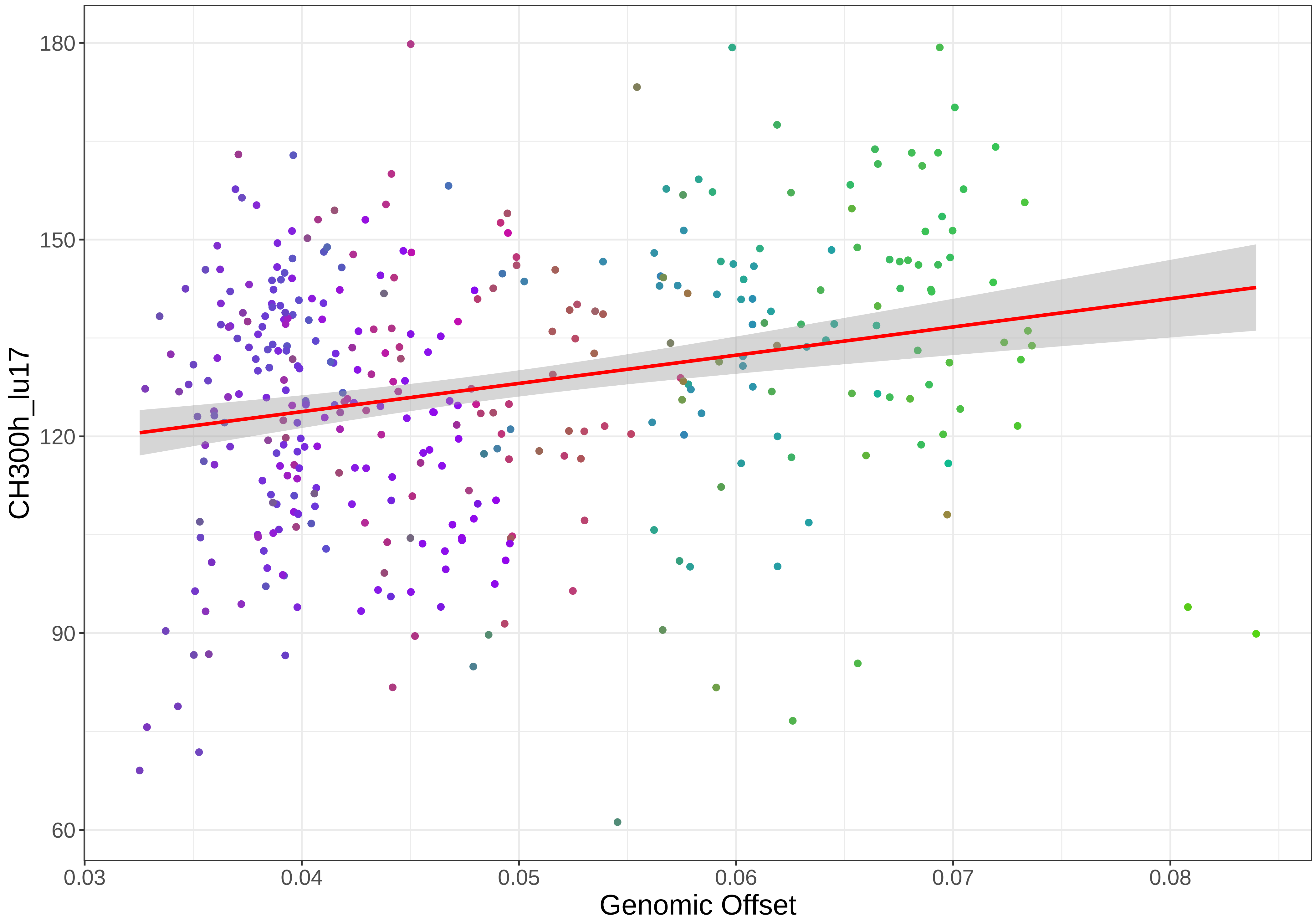
CH300h_lu17
180
150
120
90
60
0.03
0.04
0.05
0.06
0.07
0.08
Genomic Offset

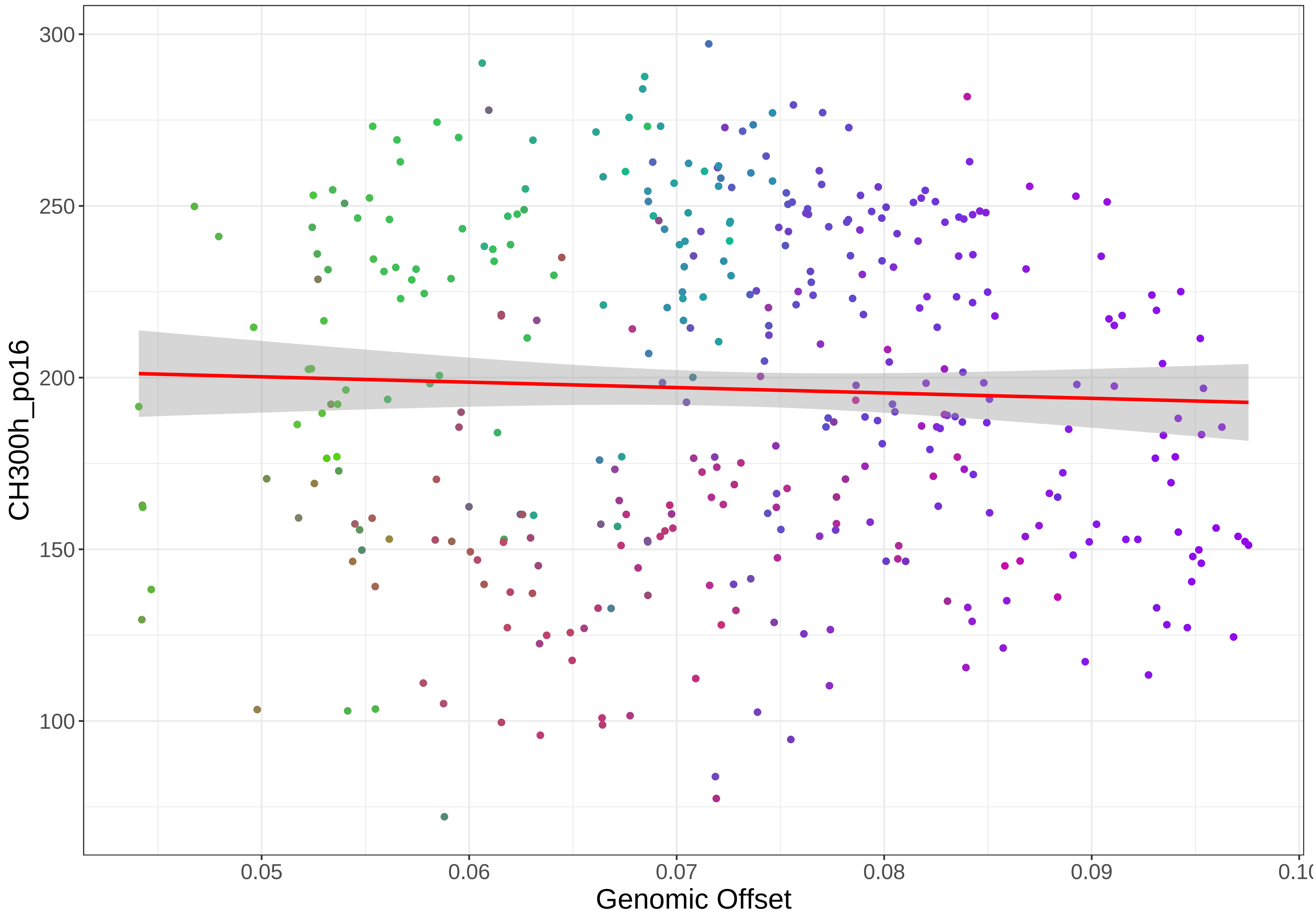

300
250
200
150
100
CH300h_po16
0.05
0.06
0.07
0.08
0.09
Genomic Offset

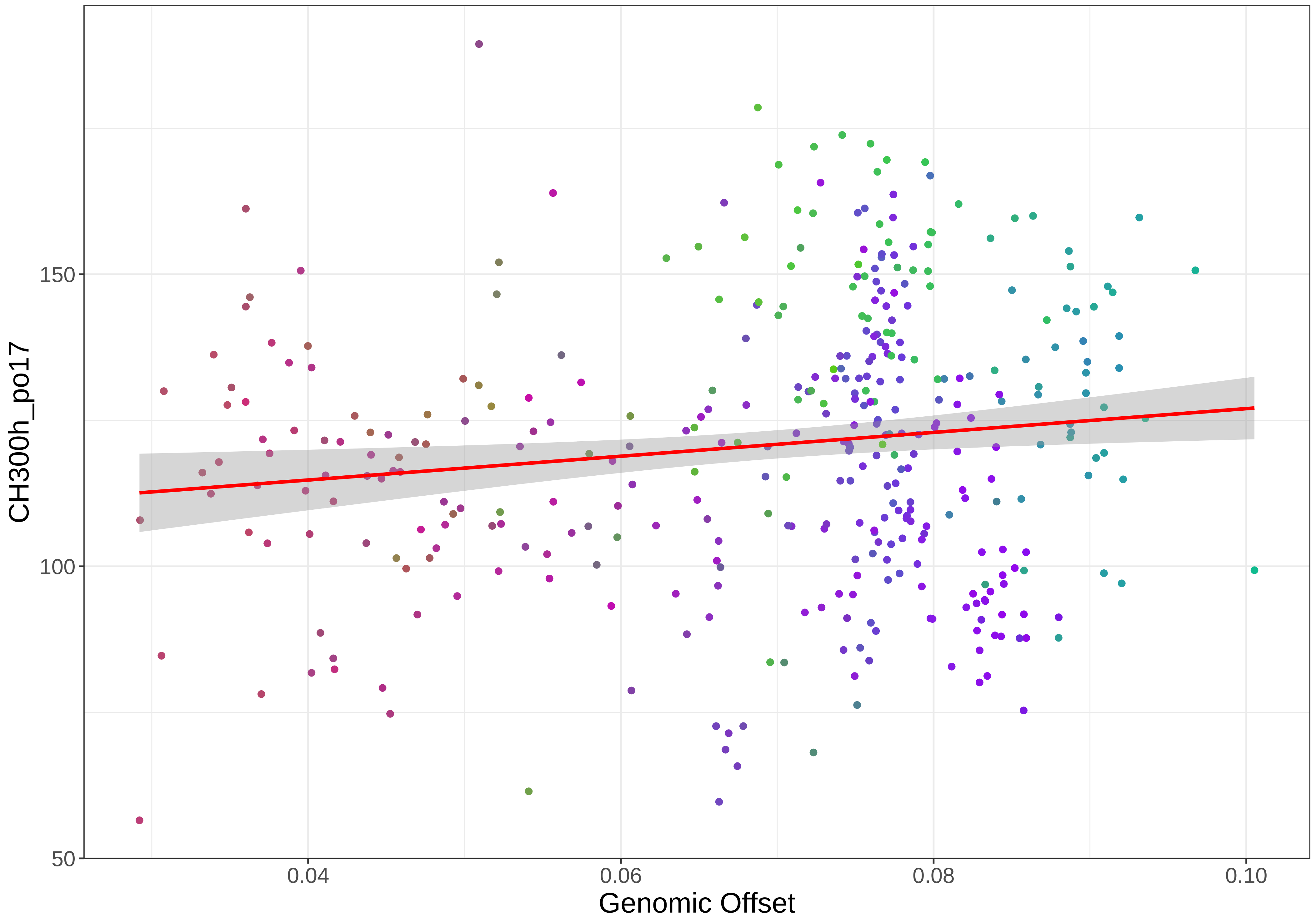
CH300h_po17
150
100
50
0.04
0.06
0.08
0.10
Genomic Offset

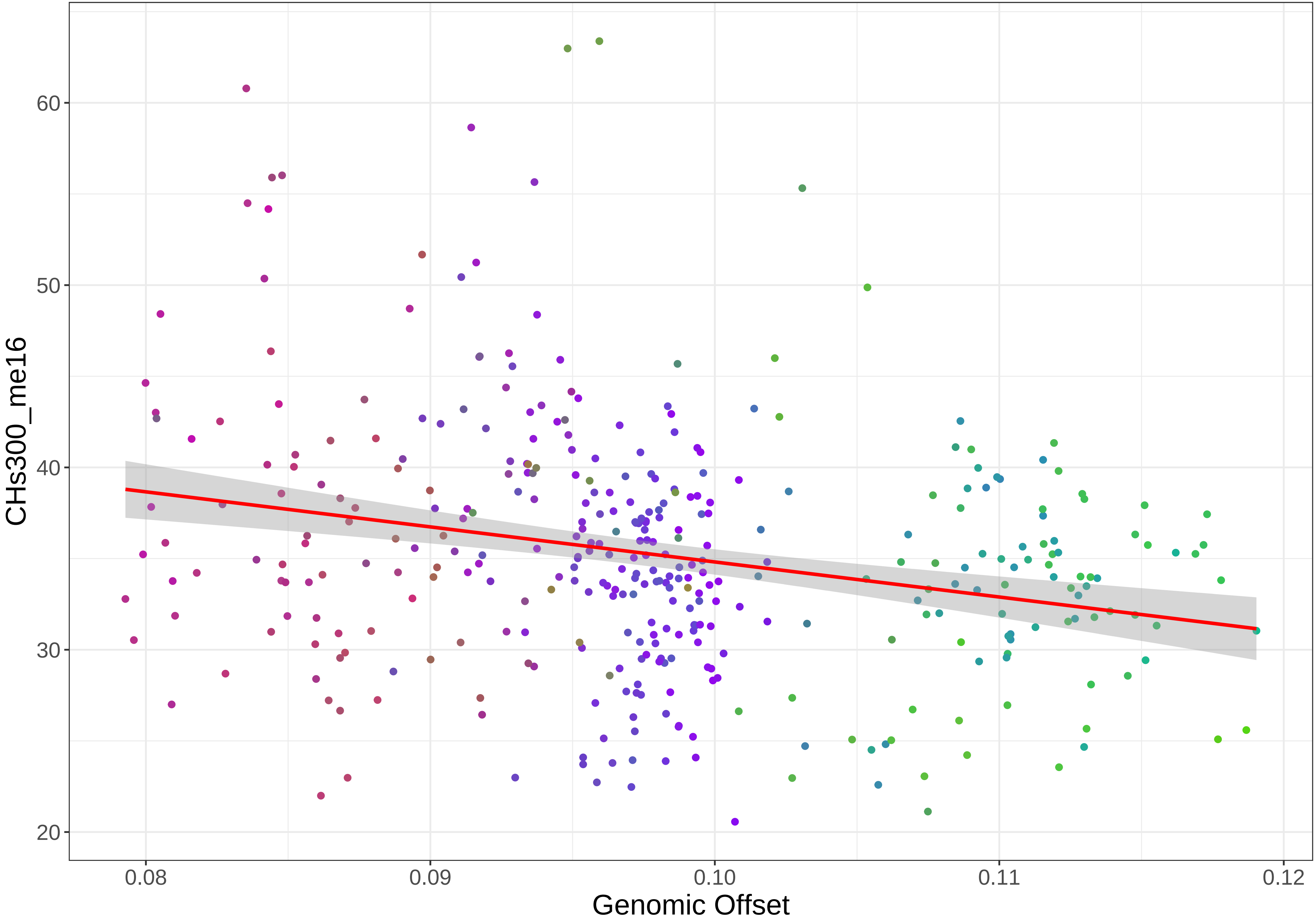

CHs300_me16
60
50
40
30
20
0.08
0.09
0.10
0.11
0.12
Genomic Offset

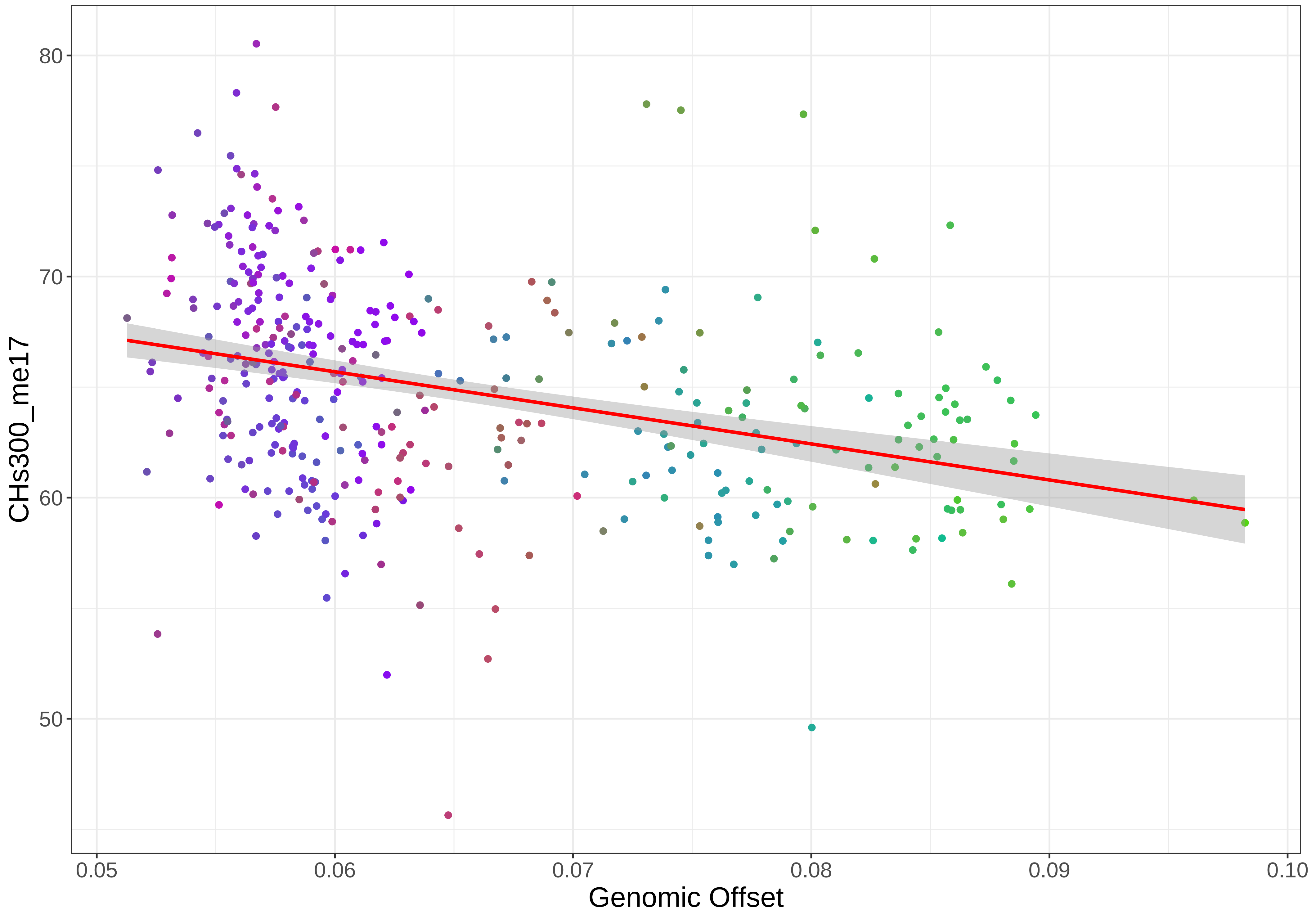

80
70
60
50
CHs300_me17
0.05
0.06
0.07
0.08
0.09
0.10
Genomic Offset

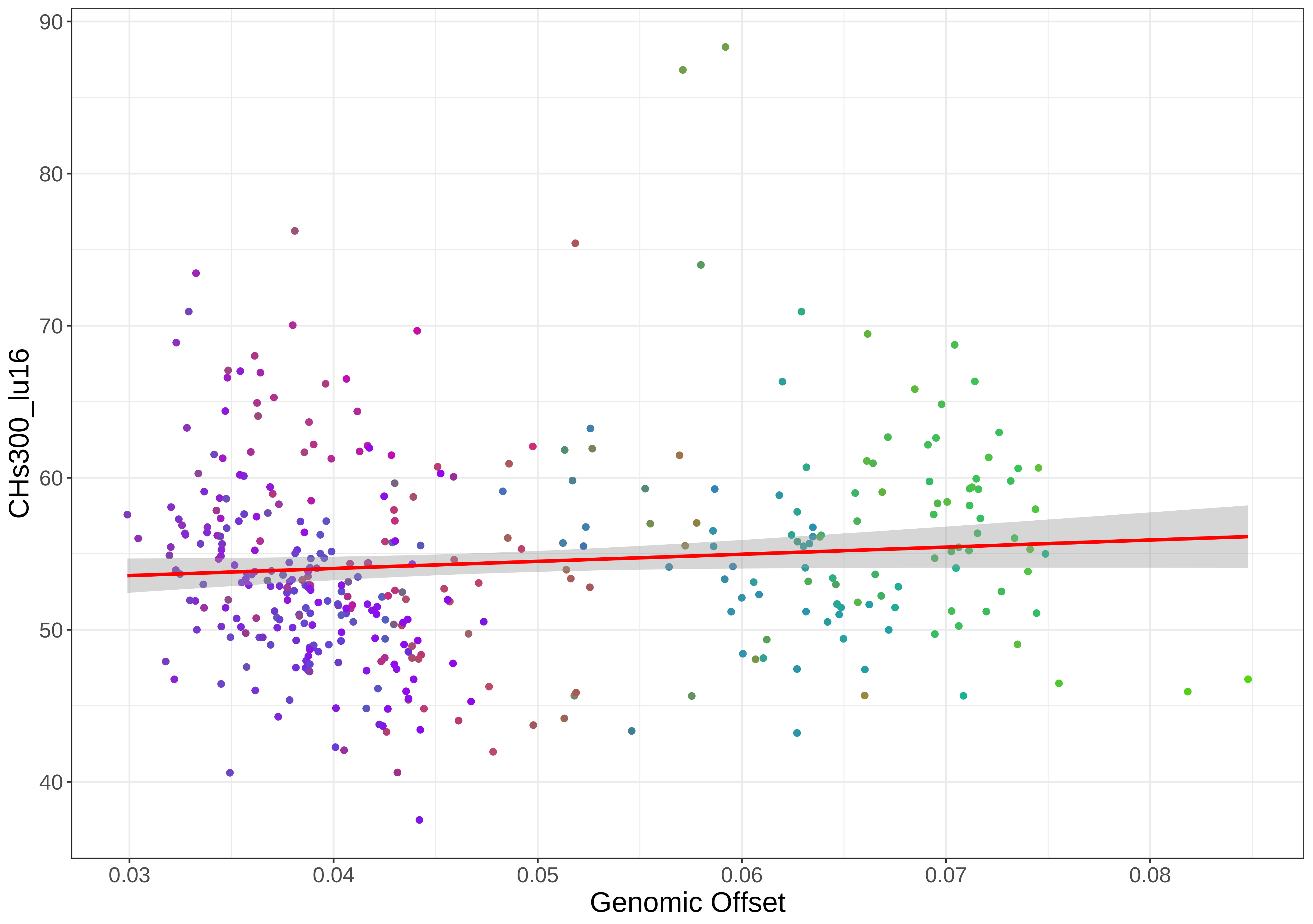

90
80
70
60
50
40
CHs300_lu16
0.03
0.04
0.05
0.06
0.07
0.08
Genomic Offset

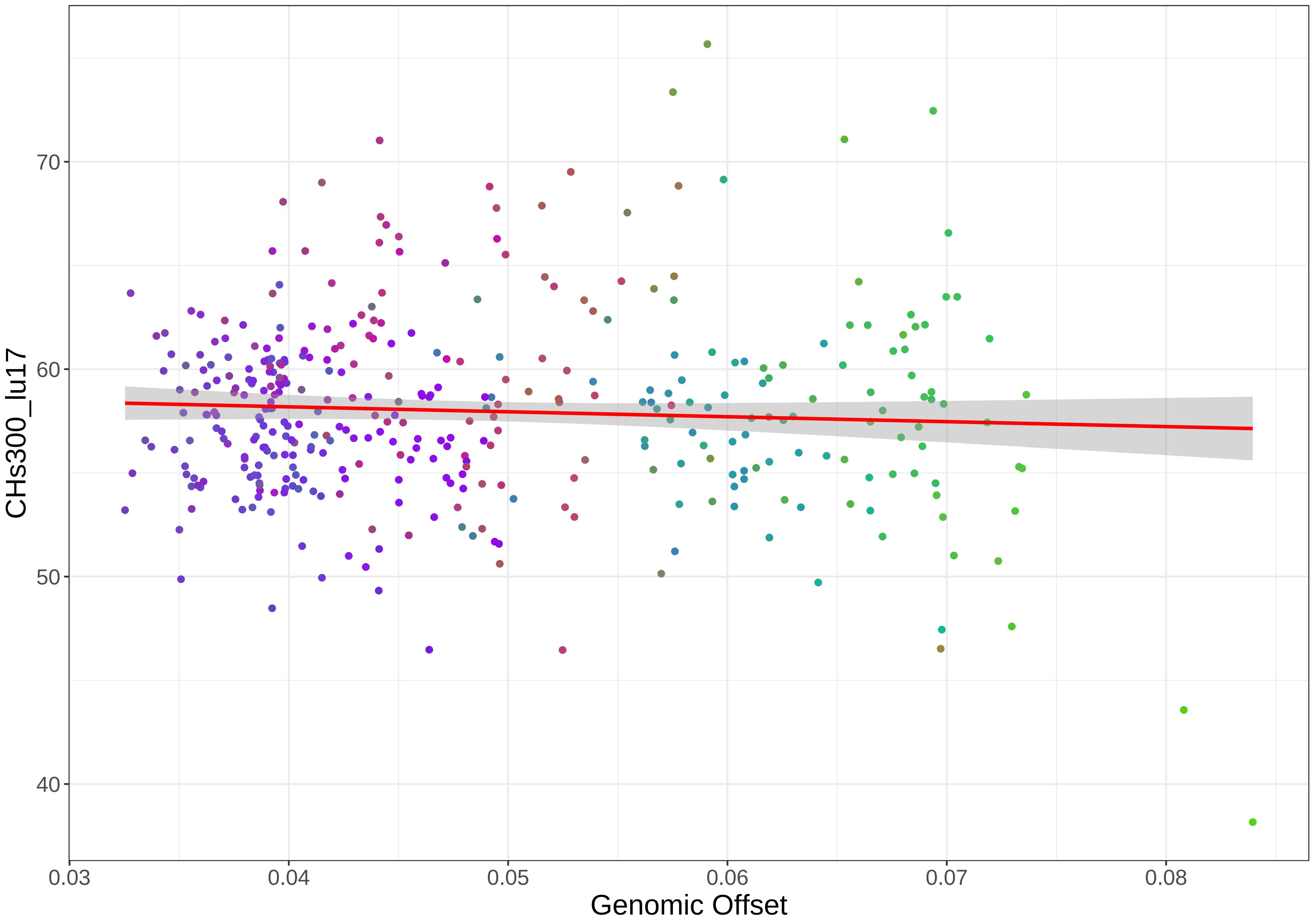

70
60
50
40
CHs300_lu17
0.03
0.04
0.05
0.06
0.07
0.08
Genomic Offset

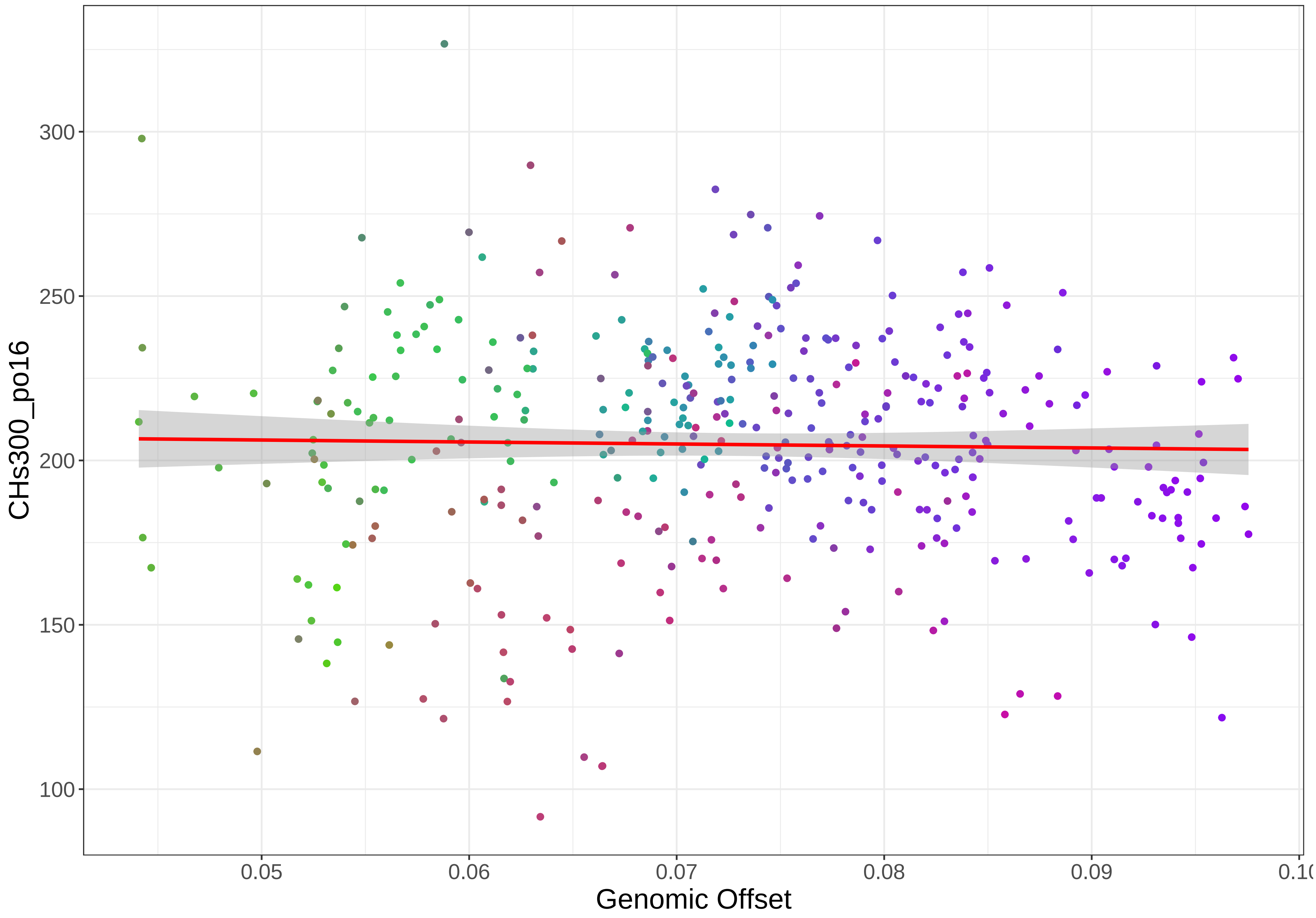
CHs300_po16
300
250
200
150
100
0.05
0.06
0.07
0.08
0.09
Genomic Offset

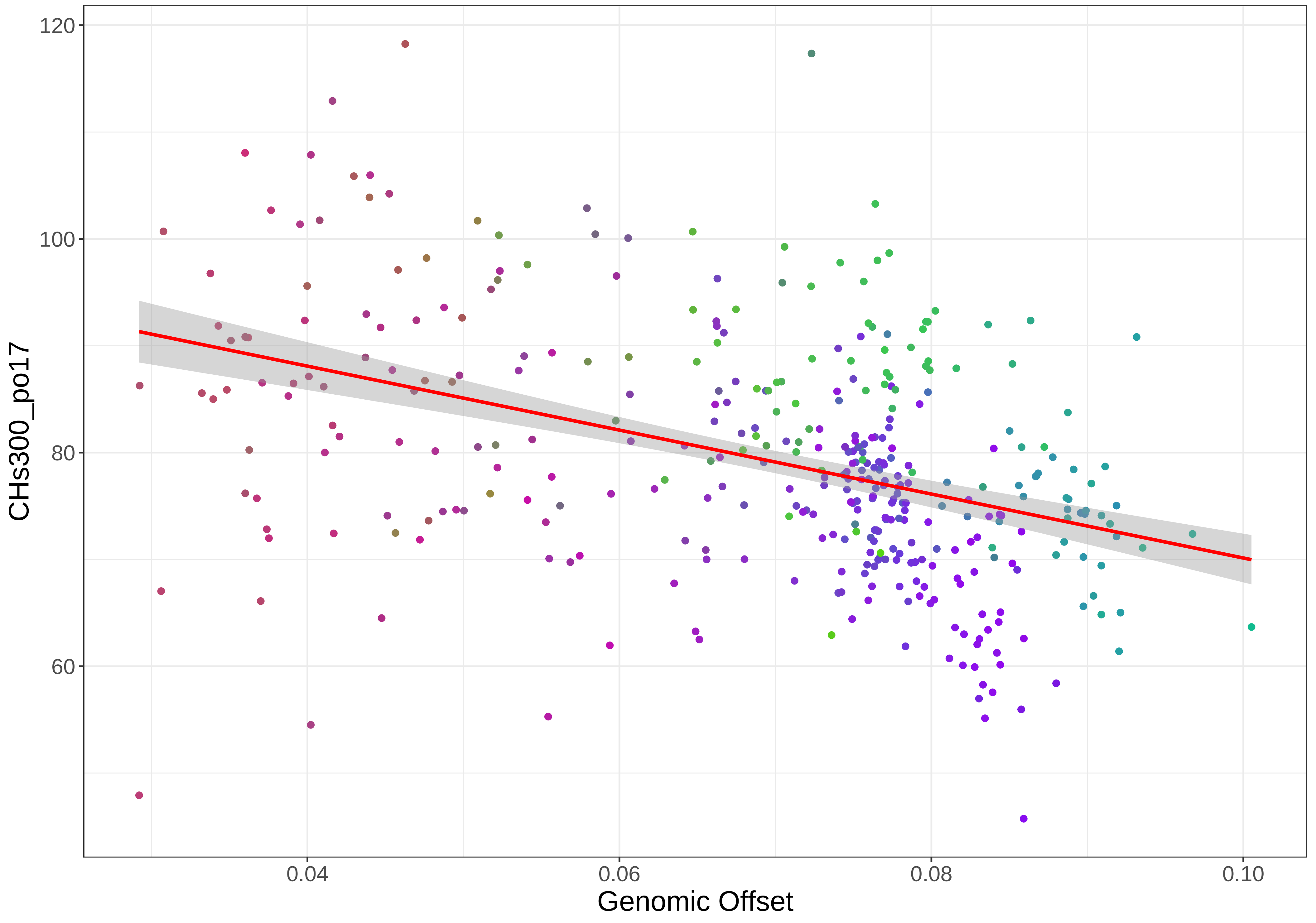

CHs300_po17
120
100
80
60
0.04
0.06
0.08
0.10
Genomic Offset

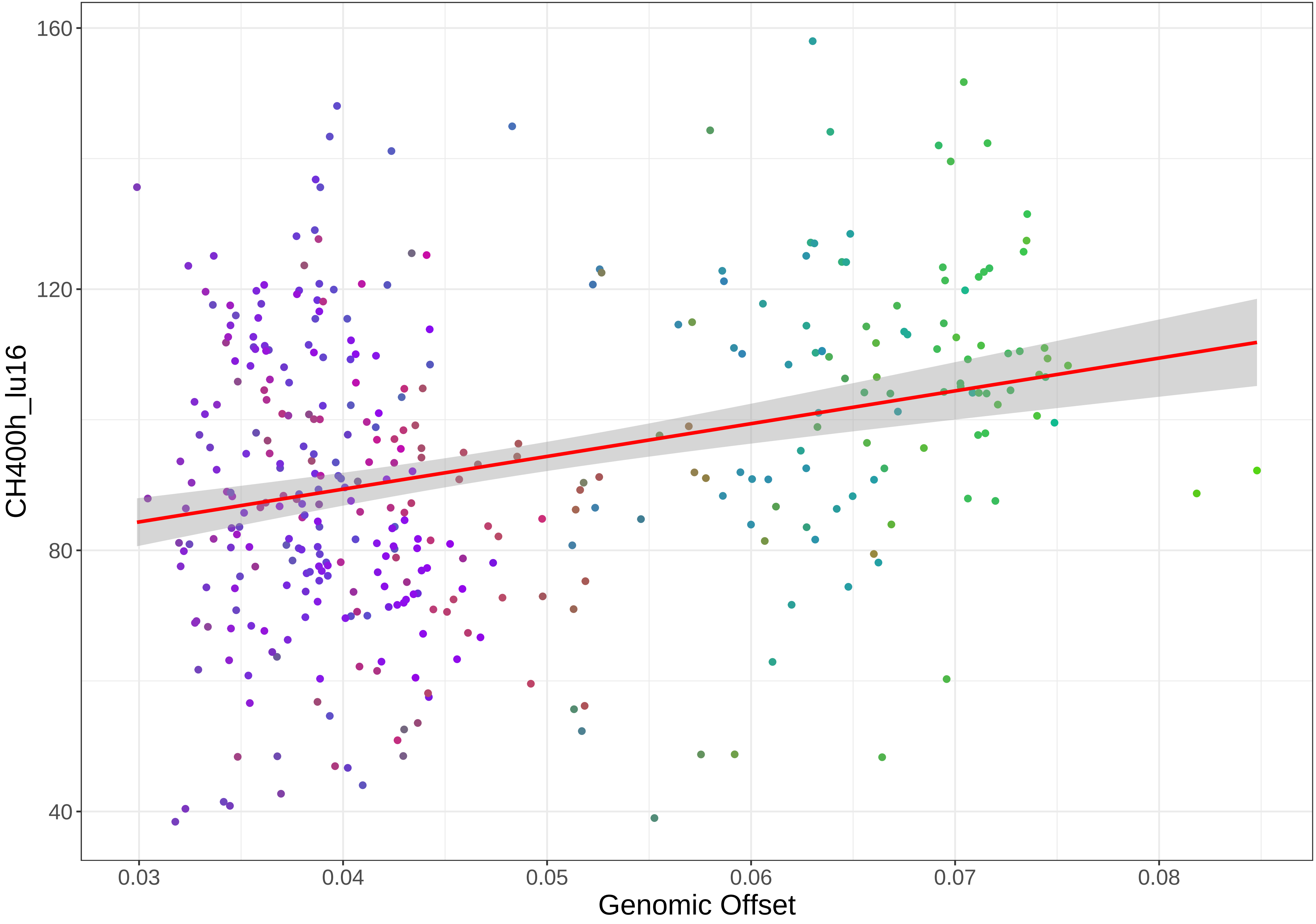

160
120
80
40
CH400h_lu16
0.03
0.04
0.05
0.06
0.07
0.08
Genomic Offset

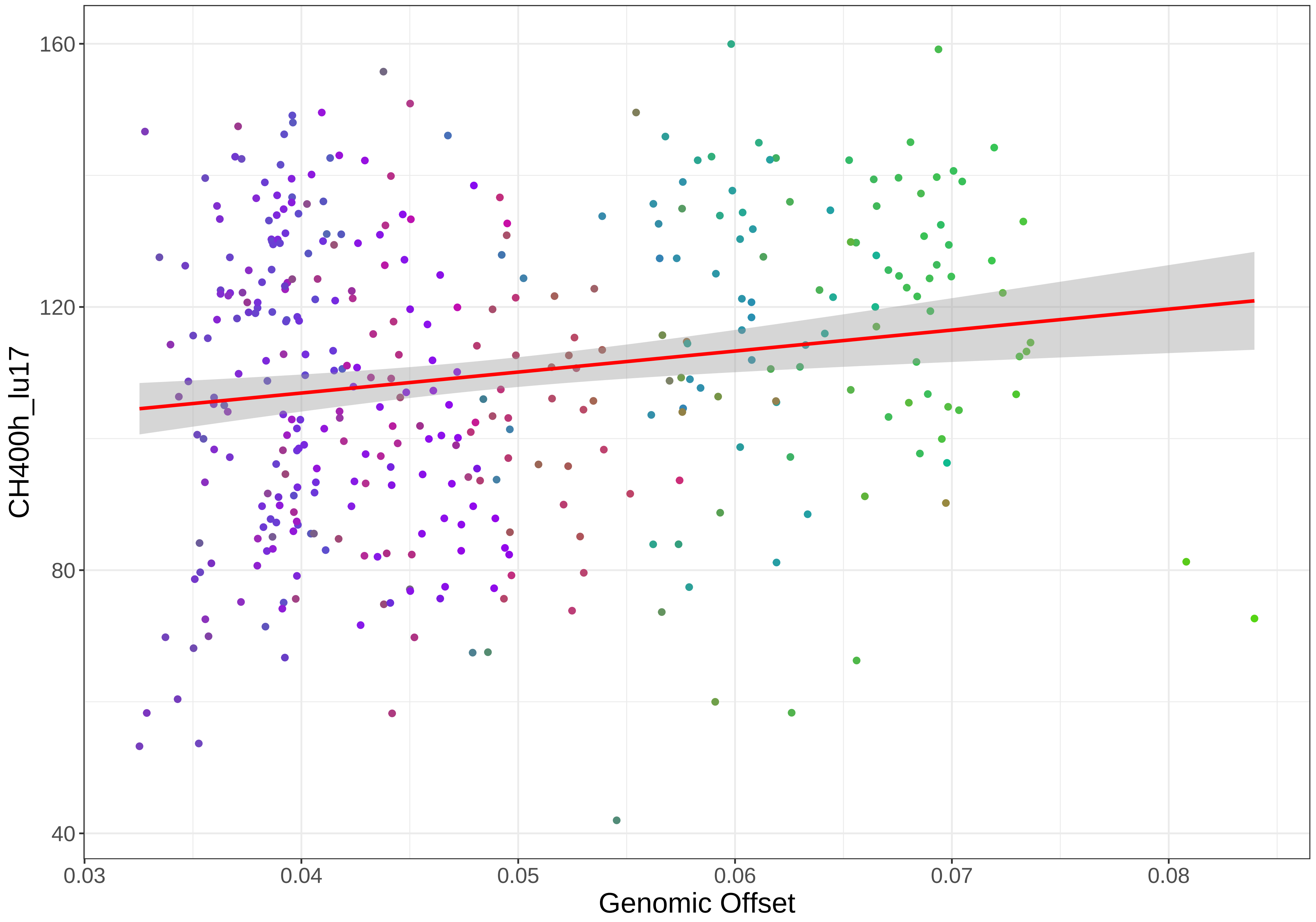

160
120
80
40
0.03
0.04
0.05
0.06
0.07
0.08
Genomic Offset
CH400h_lu17

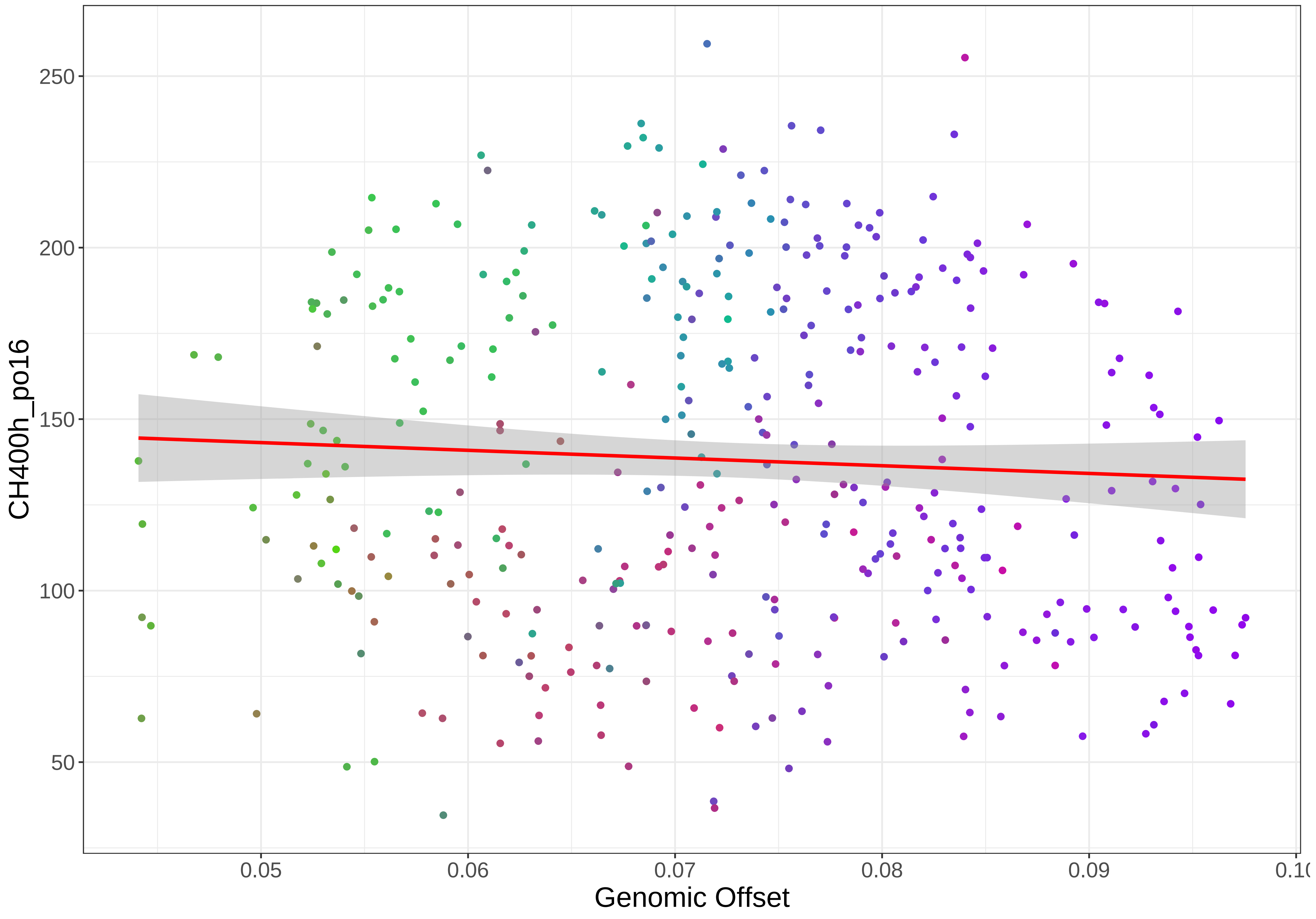

250
200
150
100
50
CH400h_po16
0.05
0.06
0.07
0.08
0.09
0.10
Genomic Offset

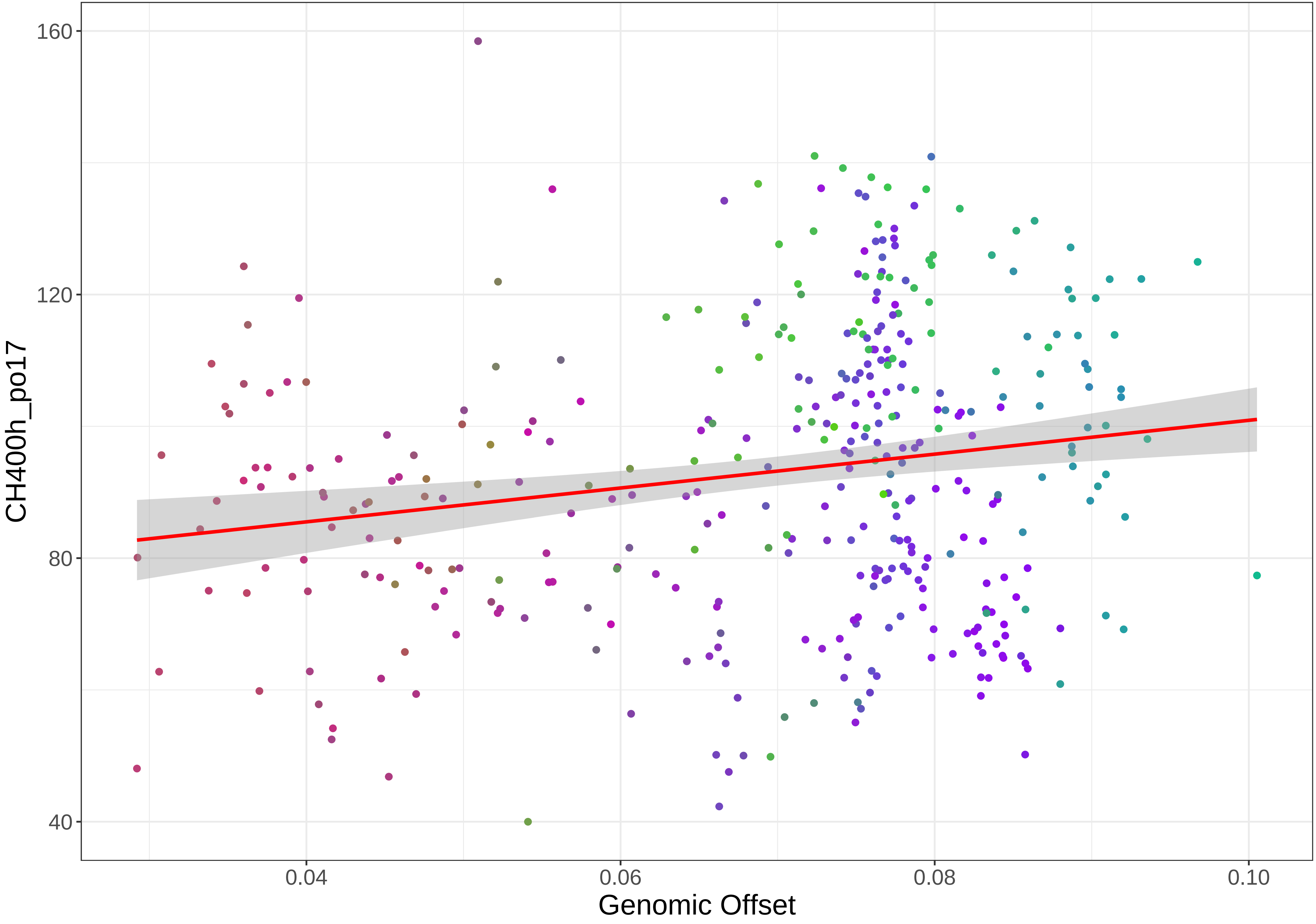

160
120
80
40
CH400h_po17
0.04
0.06
0.08
0.10
Genomic Offset

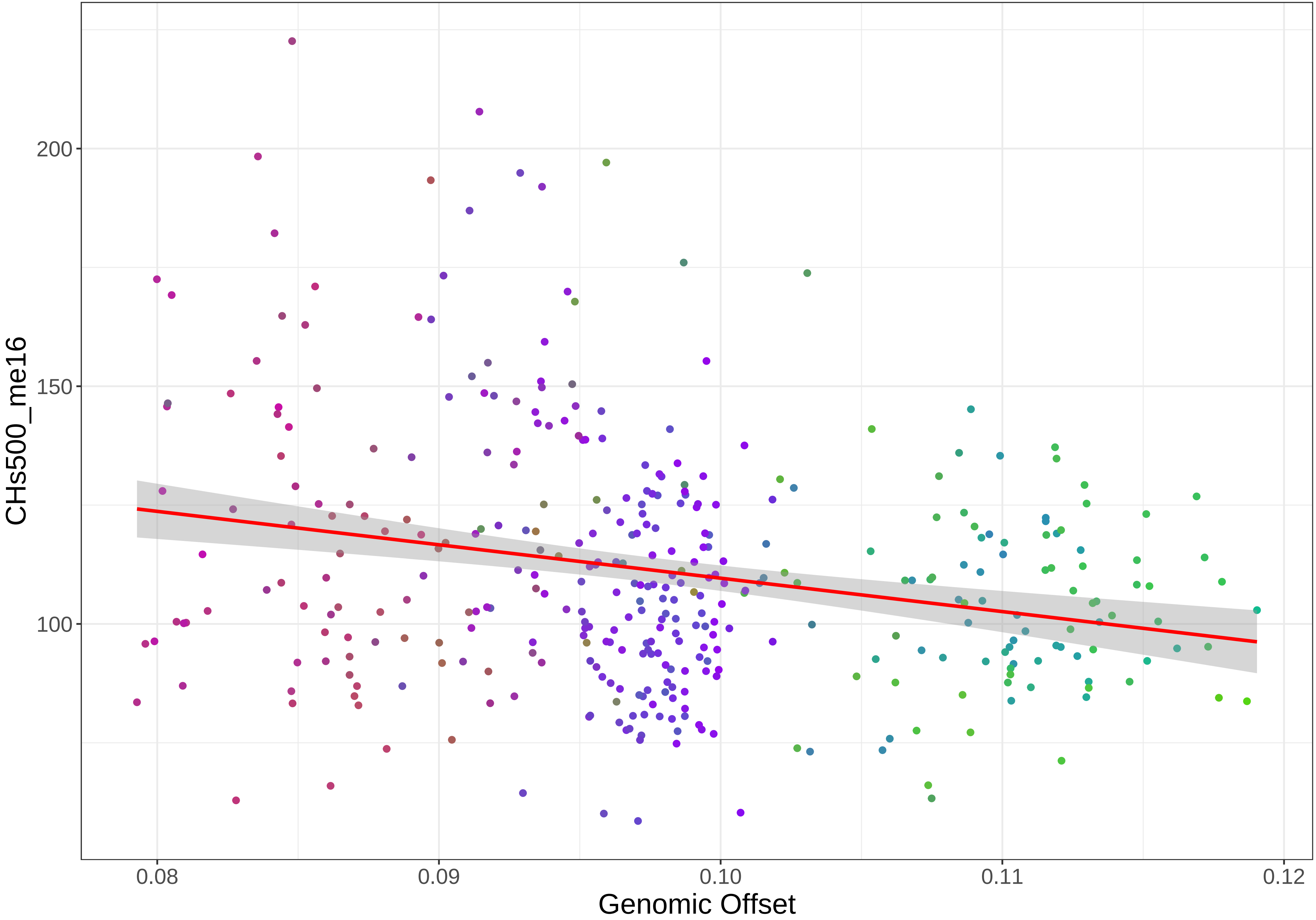
CHs500_me16
200
150
100
0.08
0.09
0.10
0.11
0.12
Genomic Offset

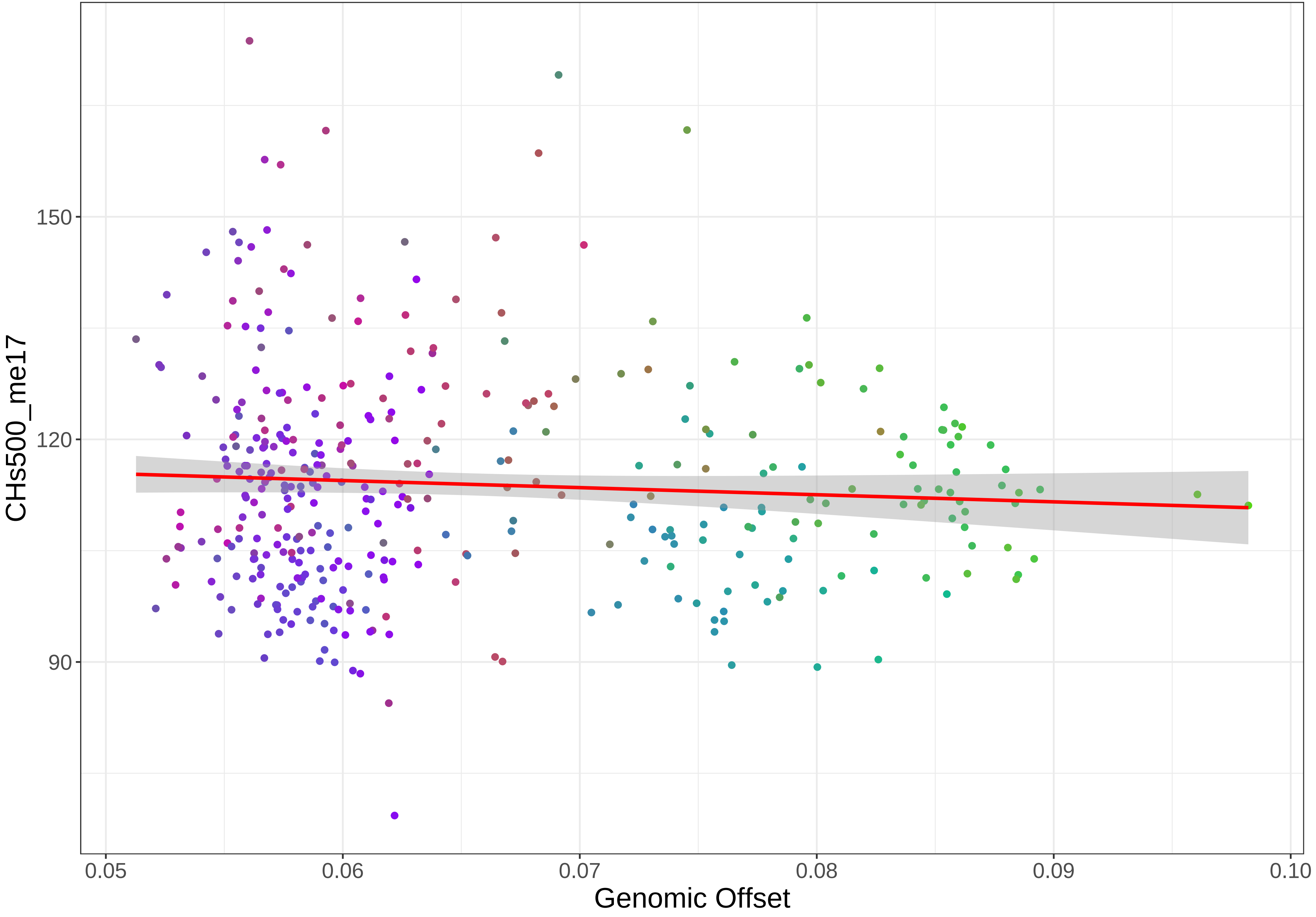

CHs500_me17
150
120
90
0.05
0.06
0.07
0.08
0.09
0.10
Genomic Offset

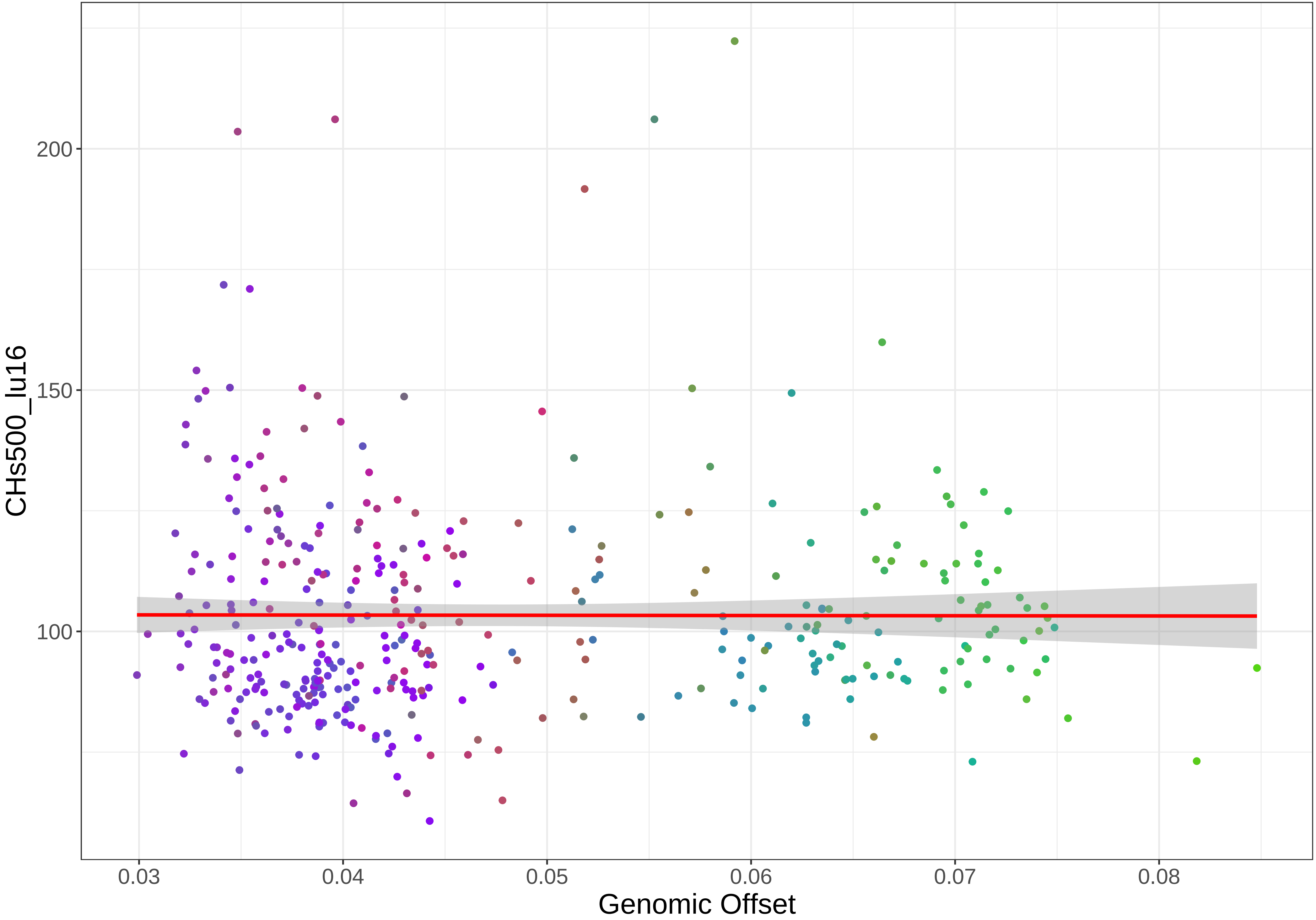

CHs500_lu16
200
150
100
0.03
0.04
0.05
0.06
0.07
0.08
Genomic Offset

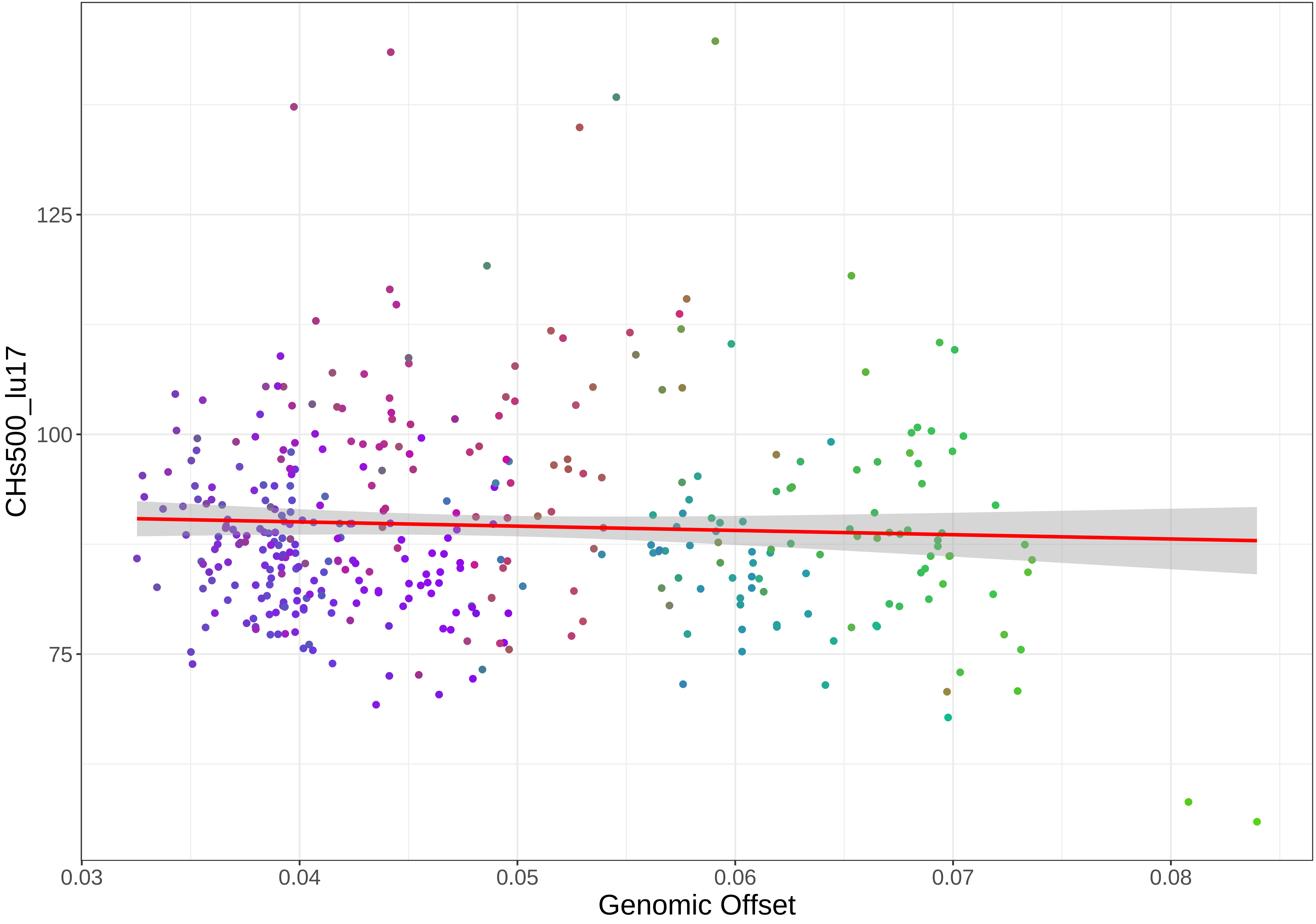

CHs500_lu17
125
100
75
0.03
0.04
0.05
0.06
0.07
0.08
Genomic Offset

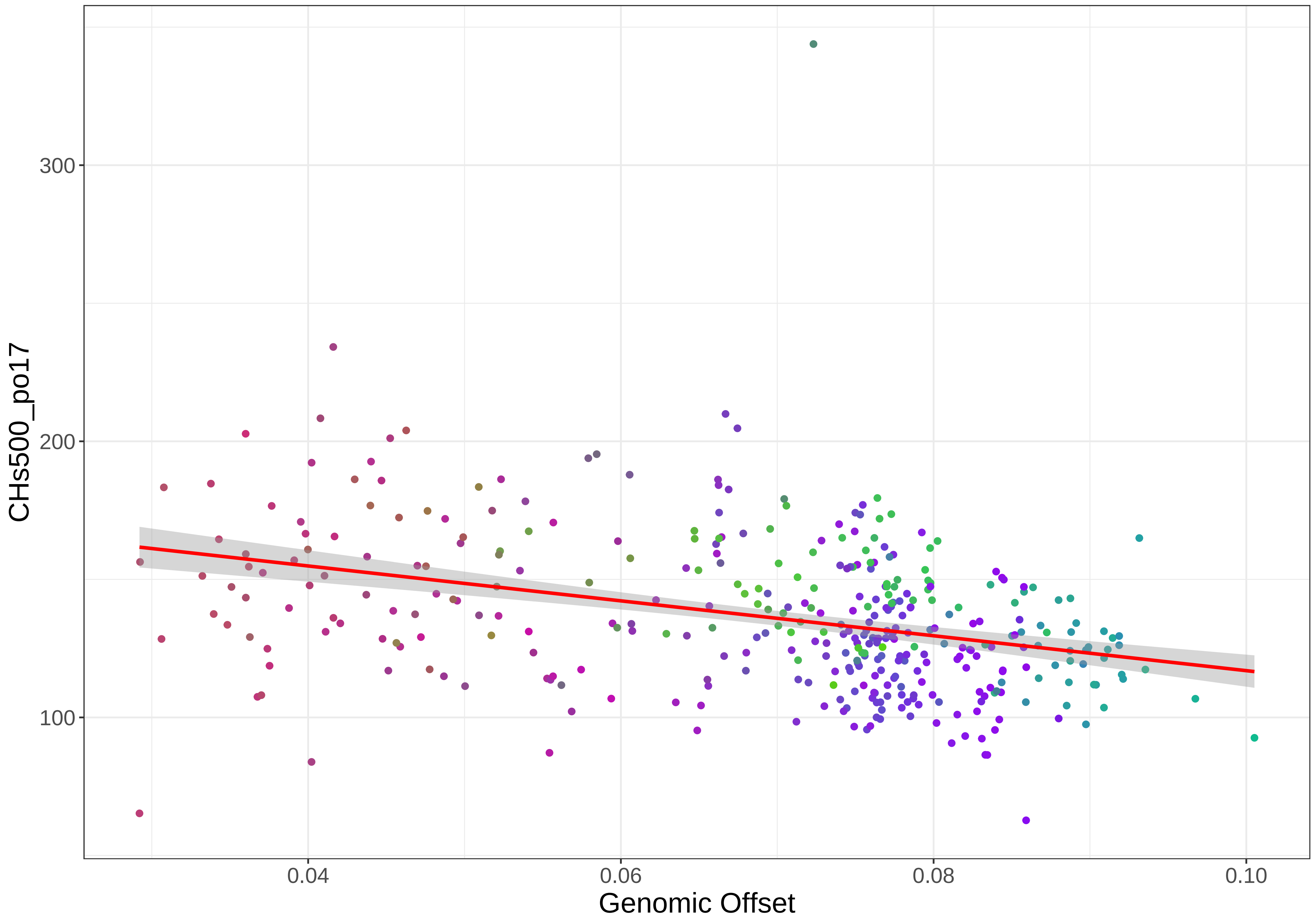
300
200
100
CHs500_po17
0.04
0.06
0.08
0.10
Genomic Offset

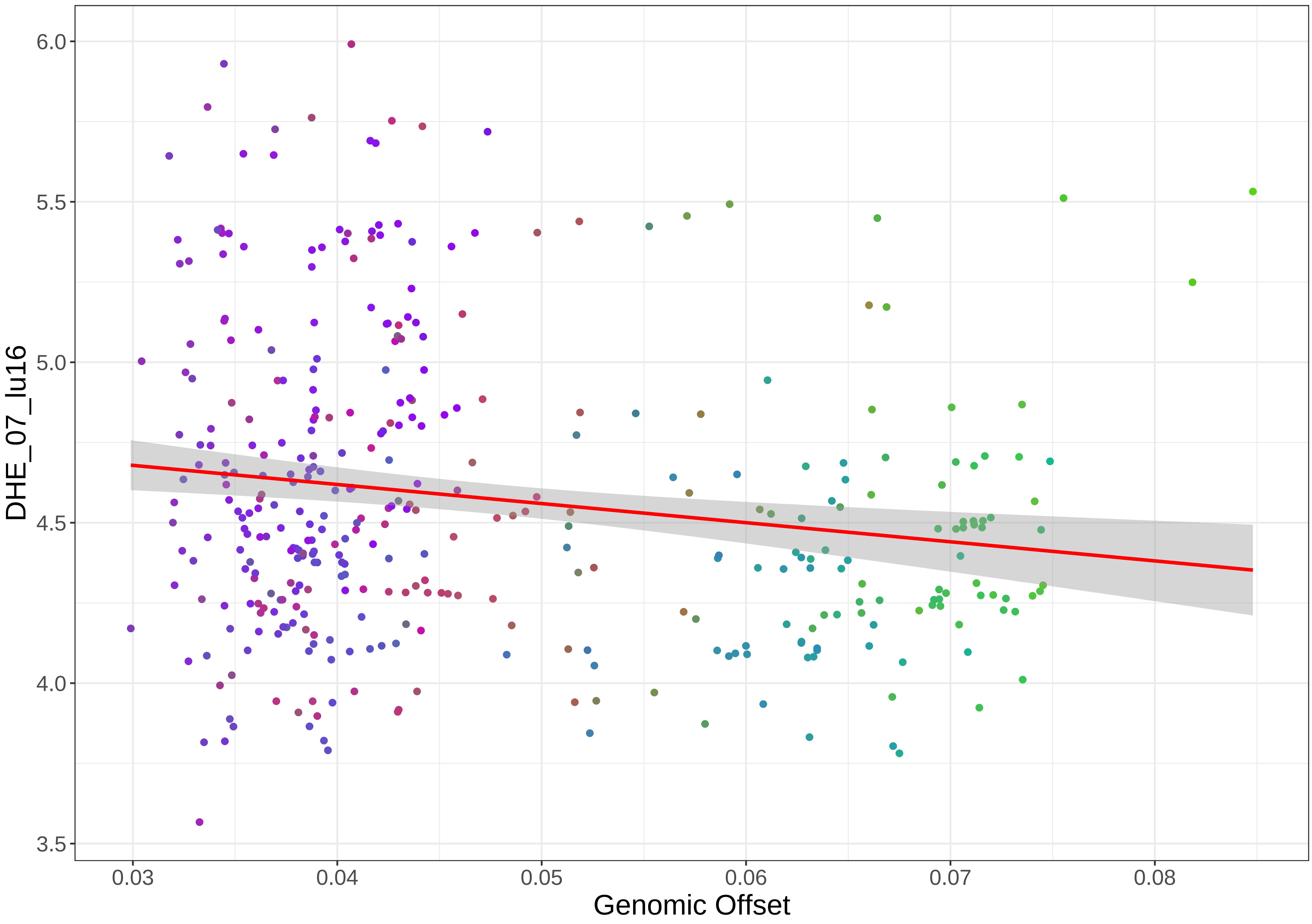

DHE_07_lu16
6.0
5.5
5.0
4.5
4.0
3.5
0.03
0.04
0.05
0.06
0.07
0.08
Genomic Offset

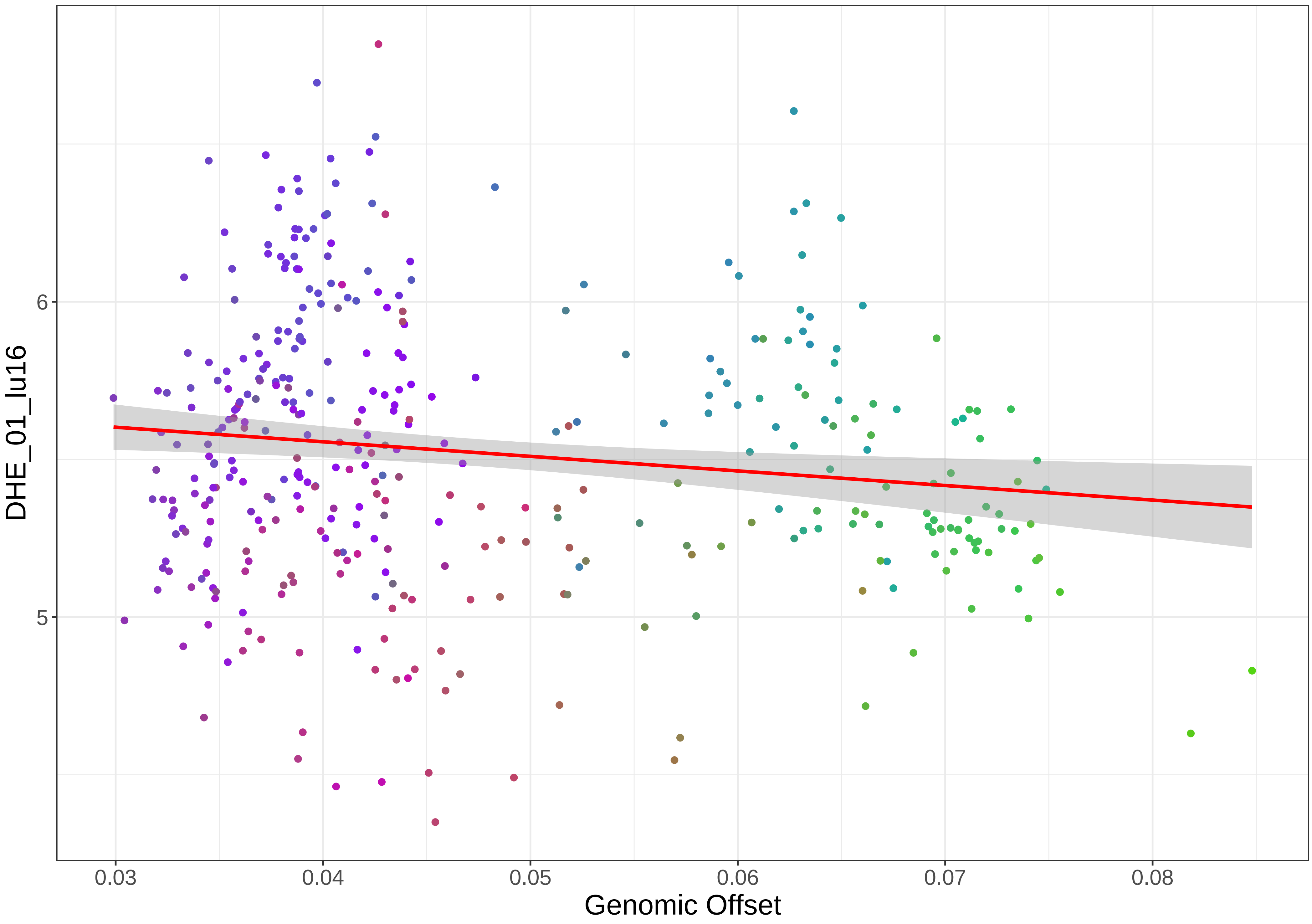

DHE_01_lu16
6
5
0.03
0.04
0.05
0.06
0.07
0.08
Genomic Offset

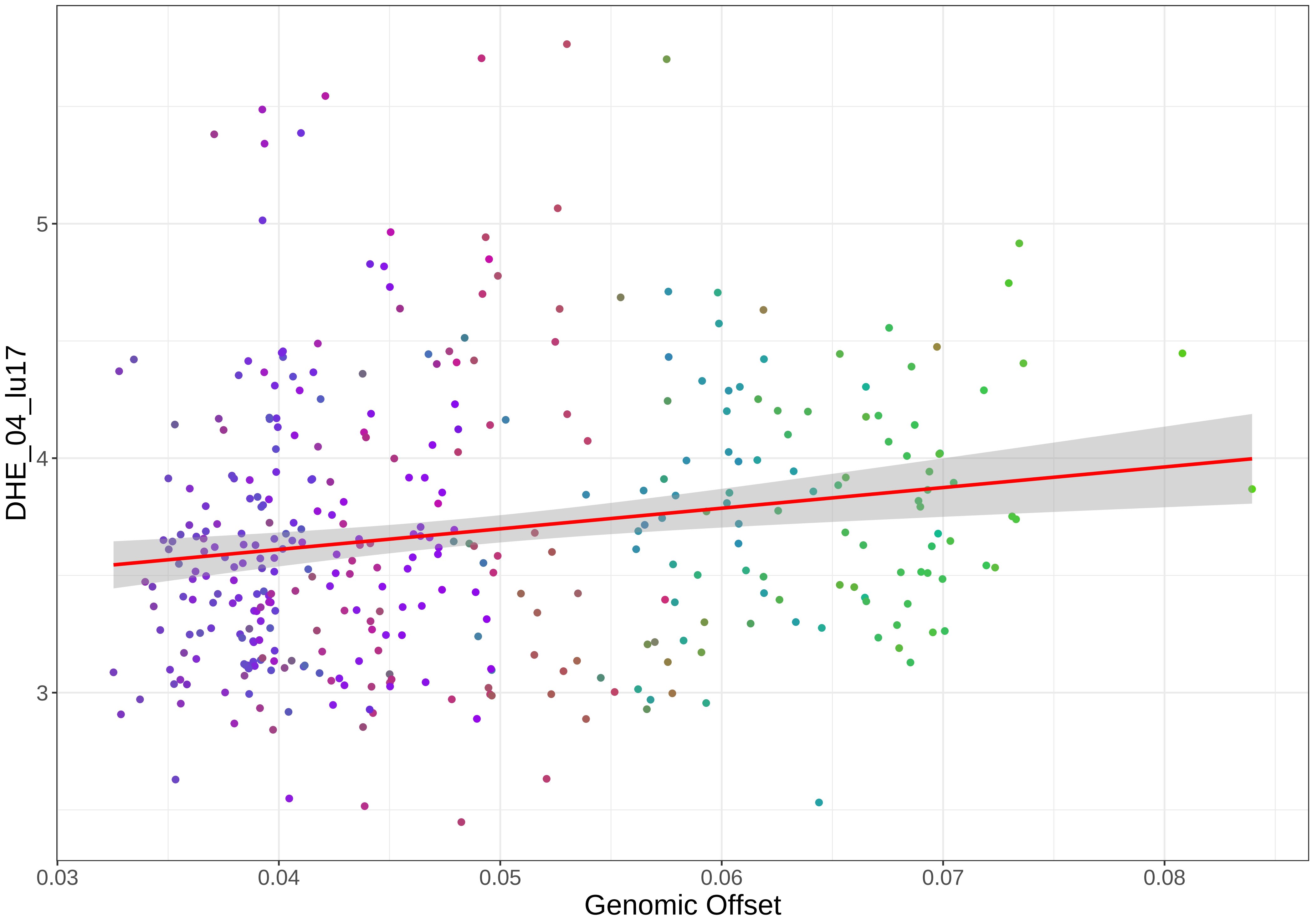

DHE_04_lu17
5
4
3
0.03
0.04
0.05
0.06
0.07
0.08
Genomic Offset

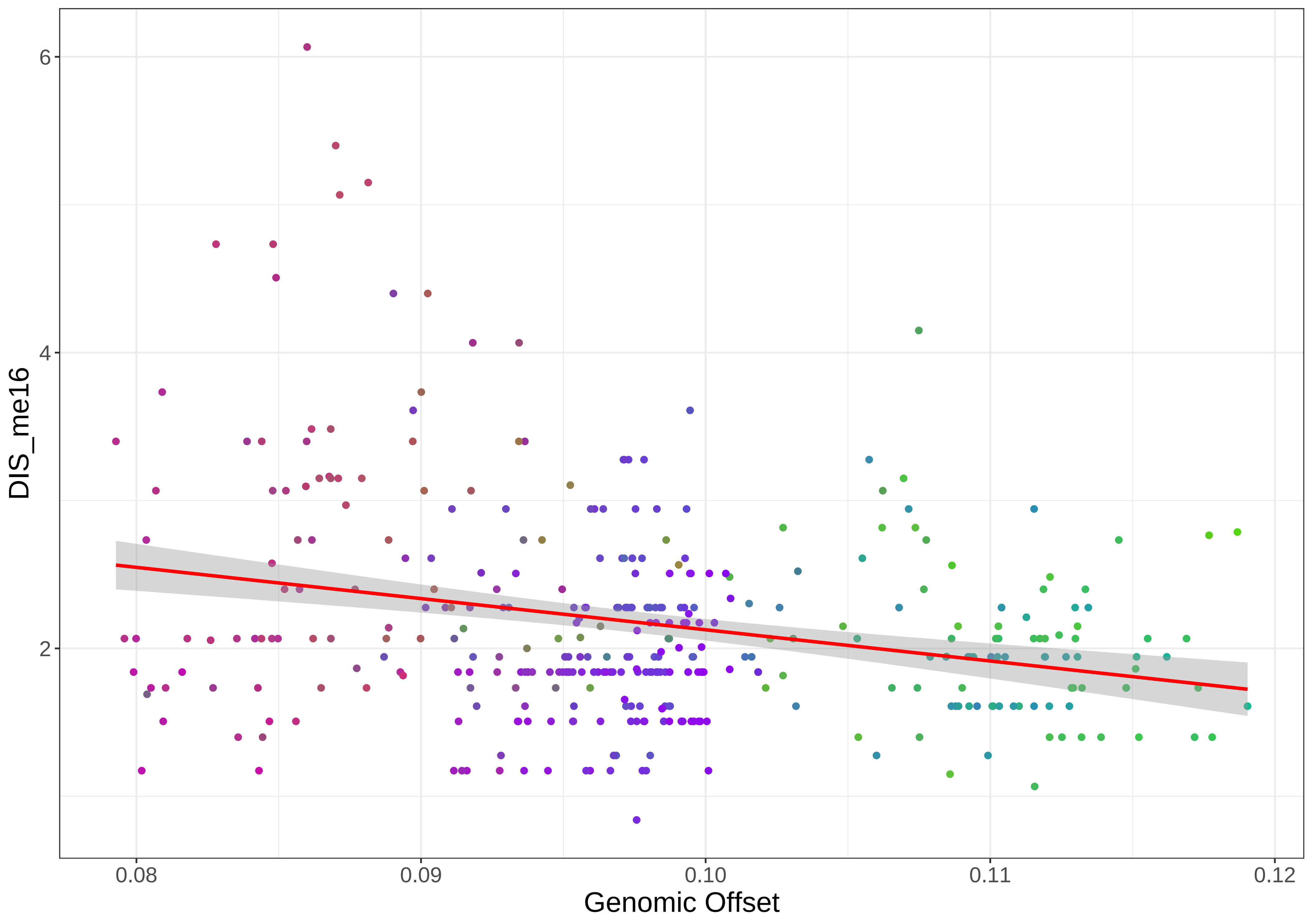

DIS_me16
6
4
2
0.08
0.09
0.10
0.11
0.12
Genomic Offset

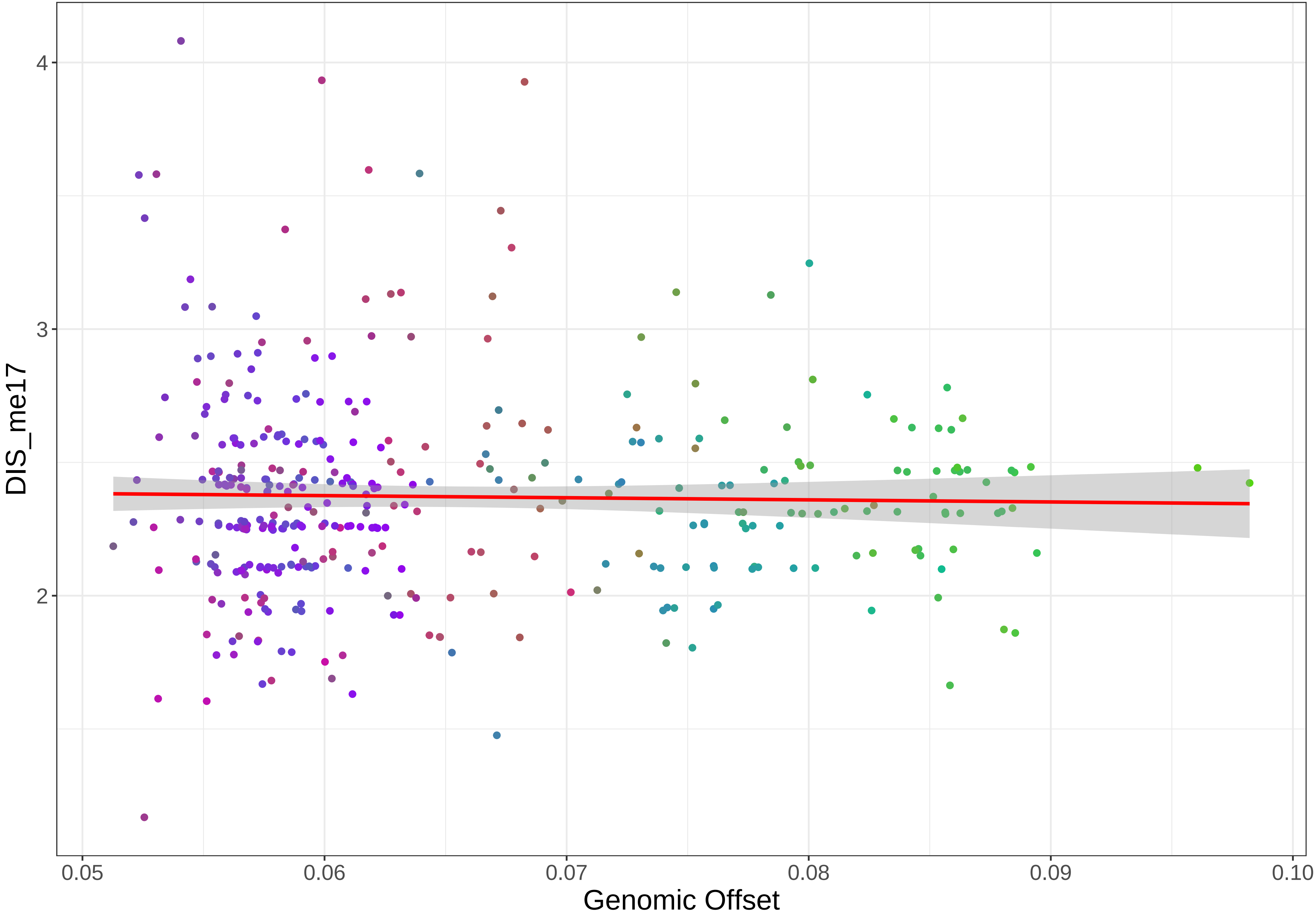

4
3
2
DIS_me17
0.05
0.06
0.07
0.08
0.09
0.10
Genomic Offset

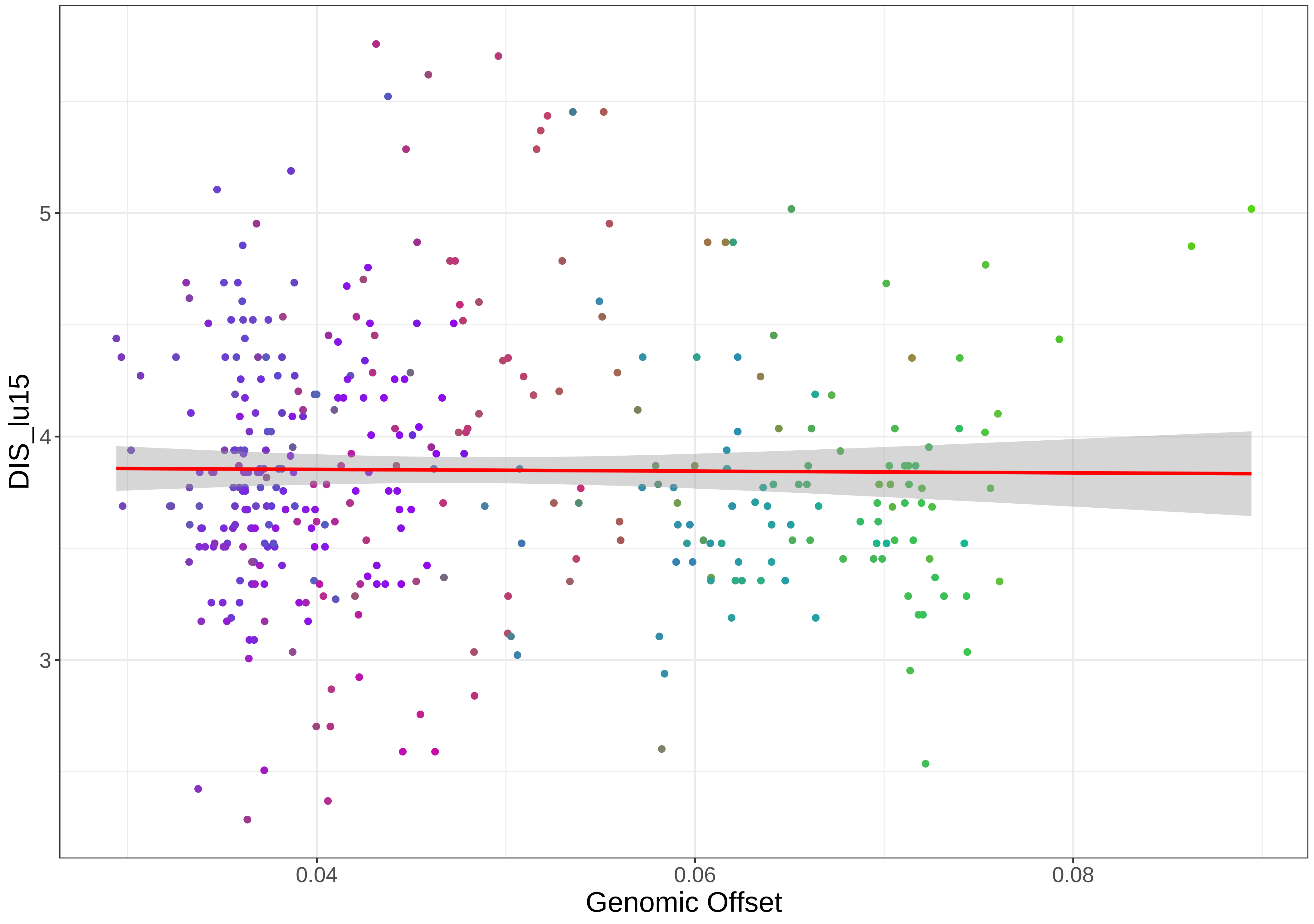

DIS_lu15
5
4
3
0.04
0.06
0.08
Genomic Offset

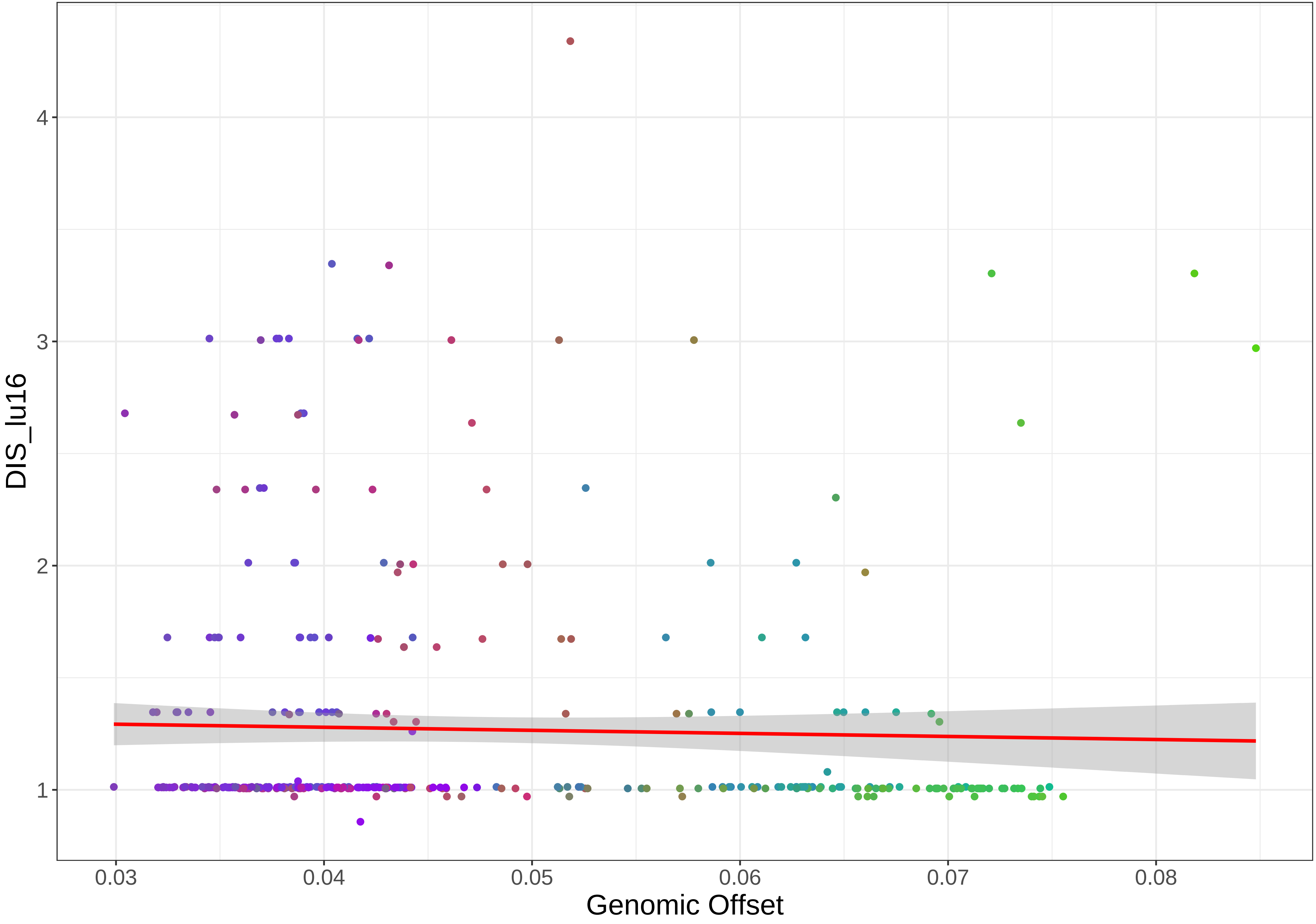

DIS_lu16
4
3
2
1
0.03
0.04
0.05
0.06
0.07
0.08
Genomic Offset

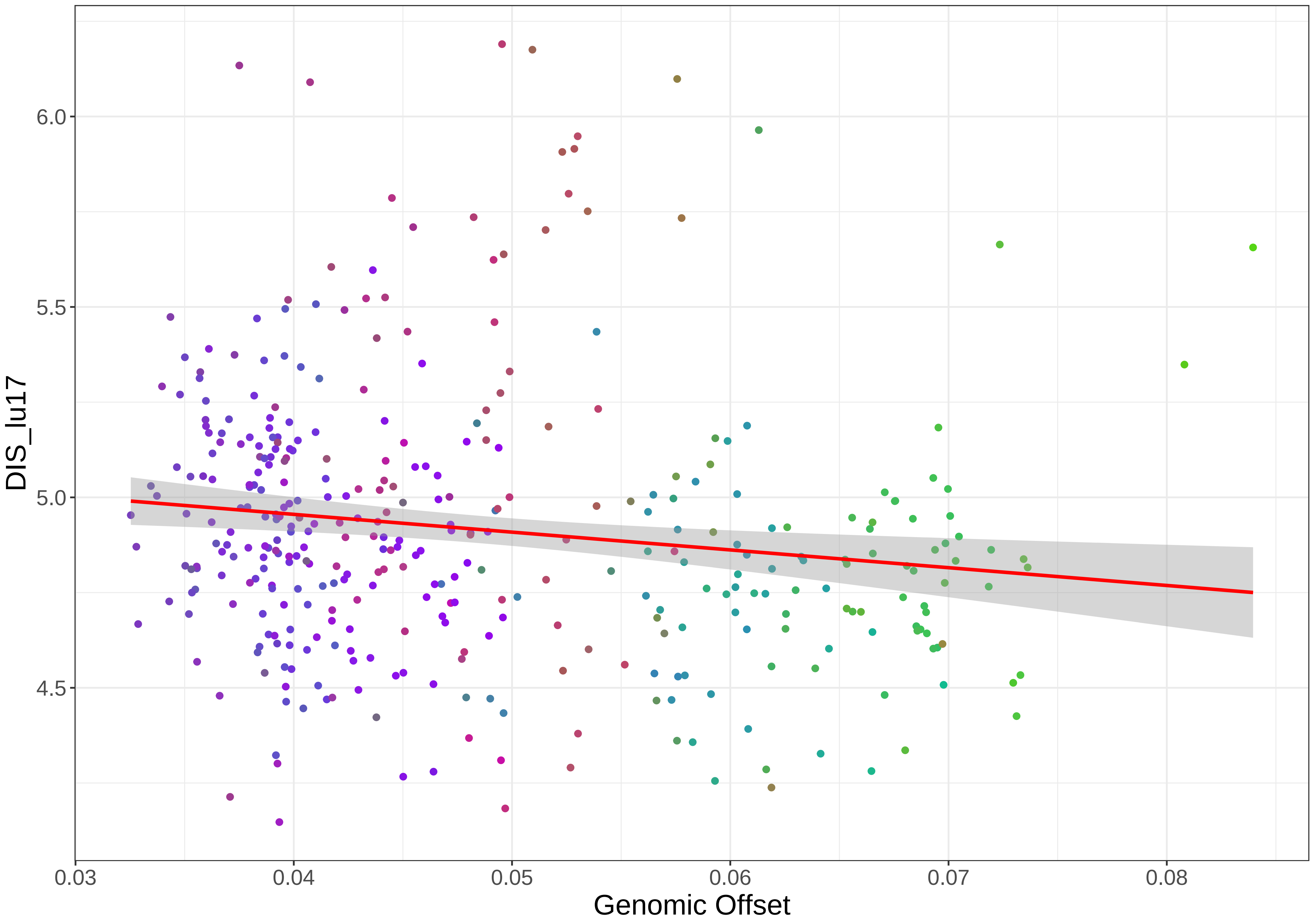

DIS_lu17
6.0
5.5
5.0
4.5
0.03
0.04
0.05
0.06
0.07
0.08
Genomic Offset

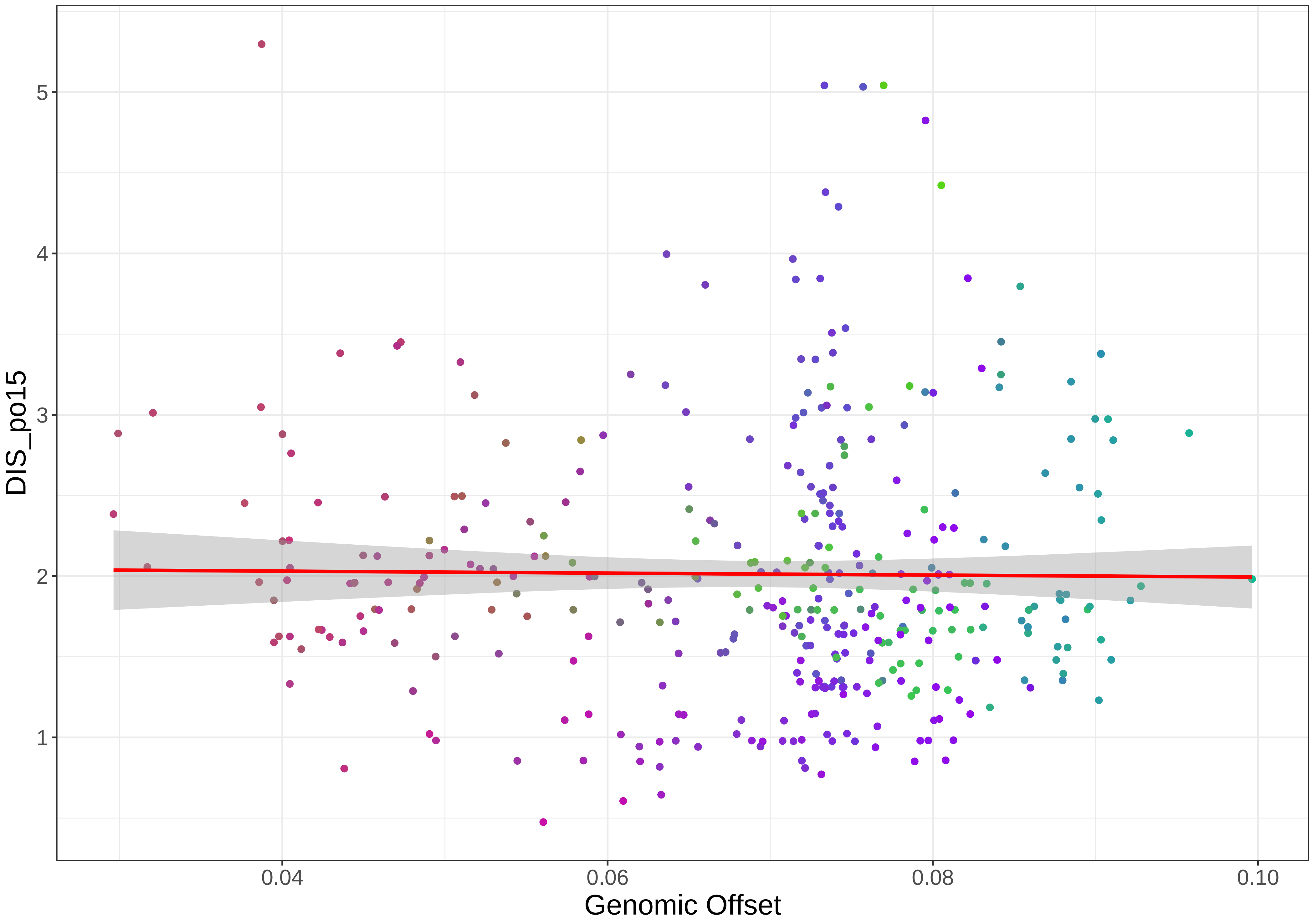

DIS_po15
1
2
3
4
5
0.04
0.06
0.08
0.10
Genomic Offset

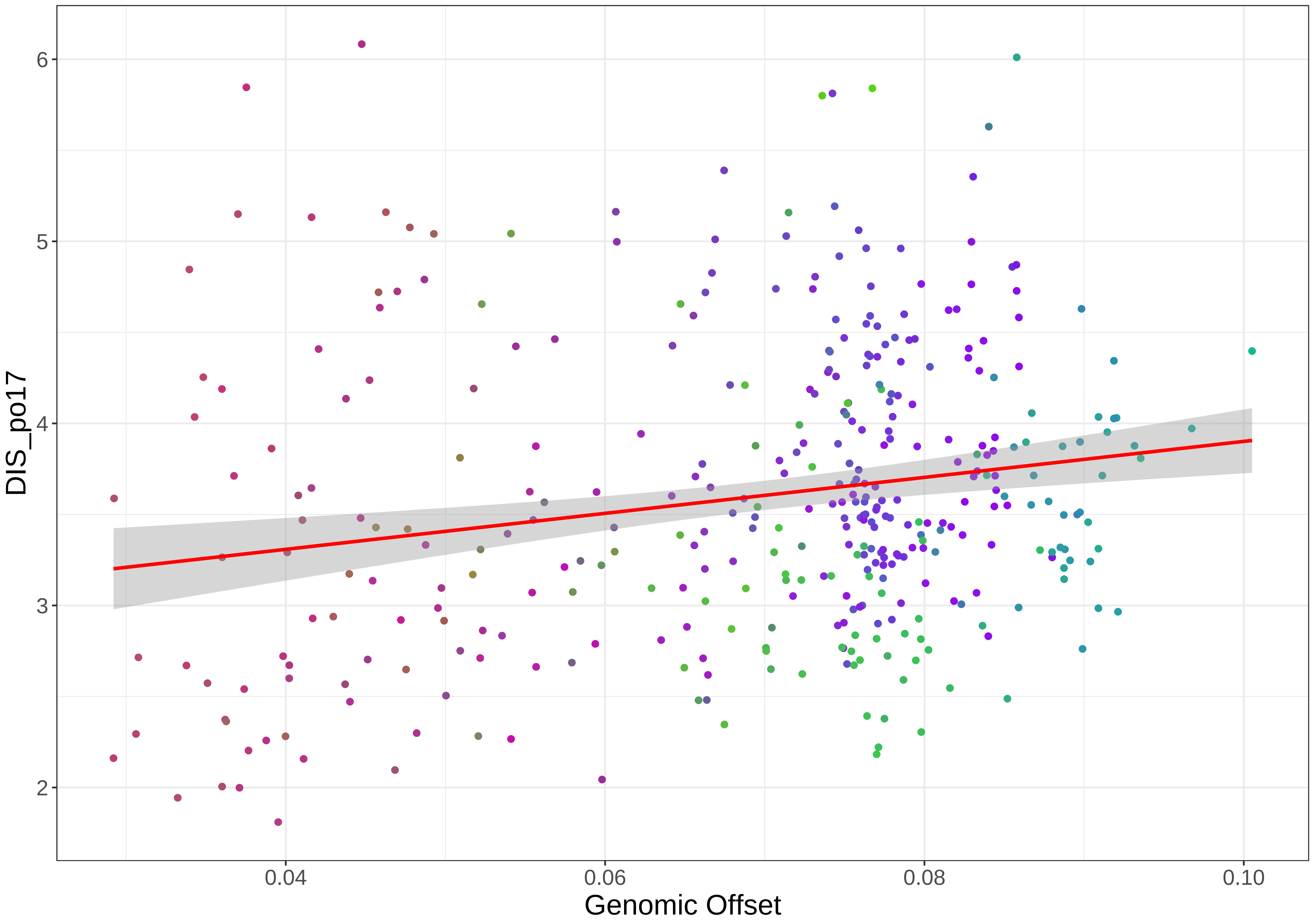
6
5
4
3
2
DIS_po17
0.04
0.06
0.08
0.10
Genomic Offset

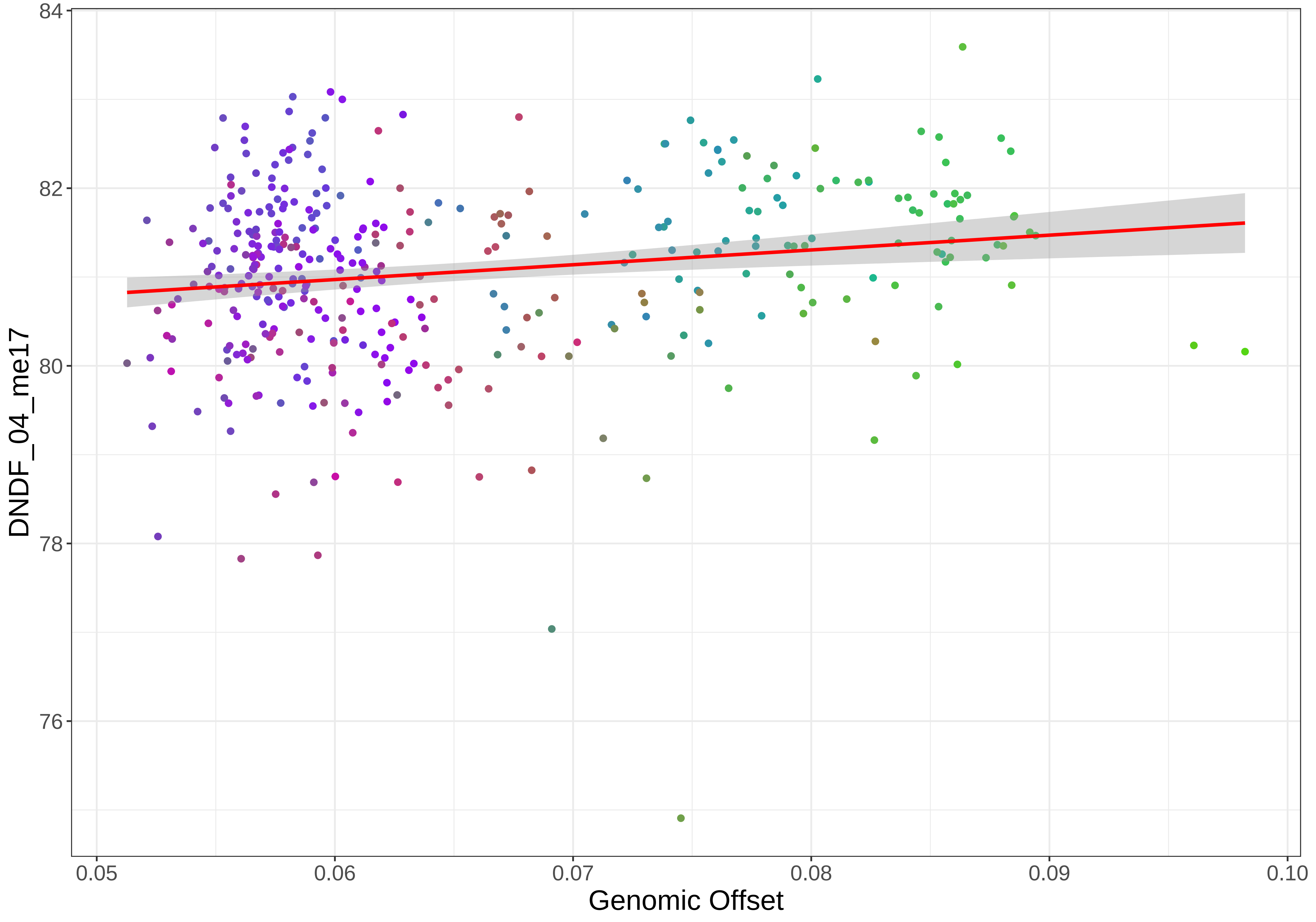

84
82
80
78
76
DNDF_04_me17
0.05
0.06
0.07
0.08
0.09
0.10
Genomic Offset

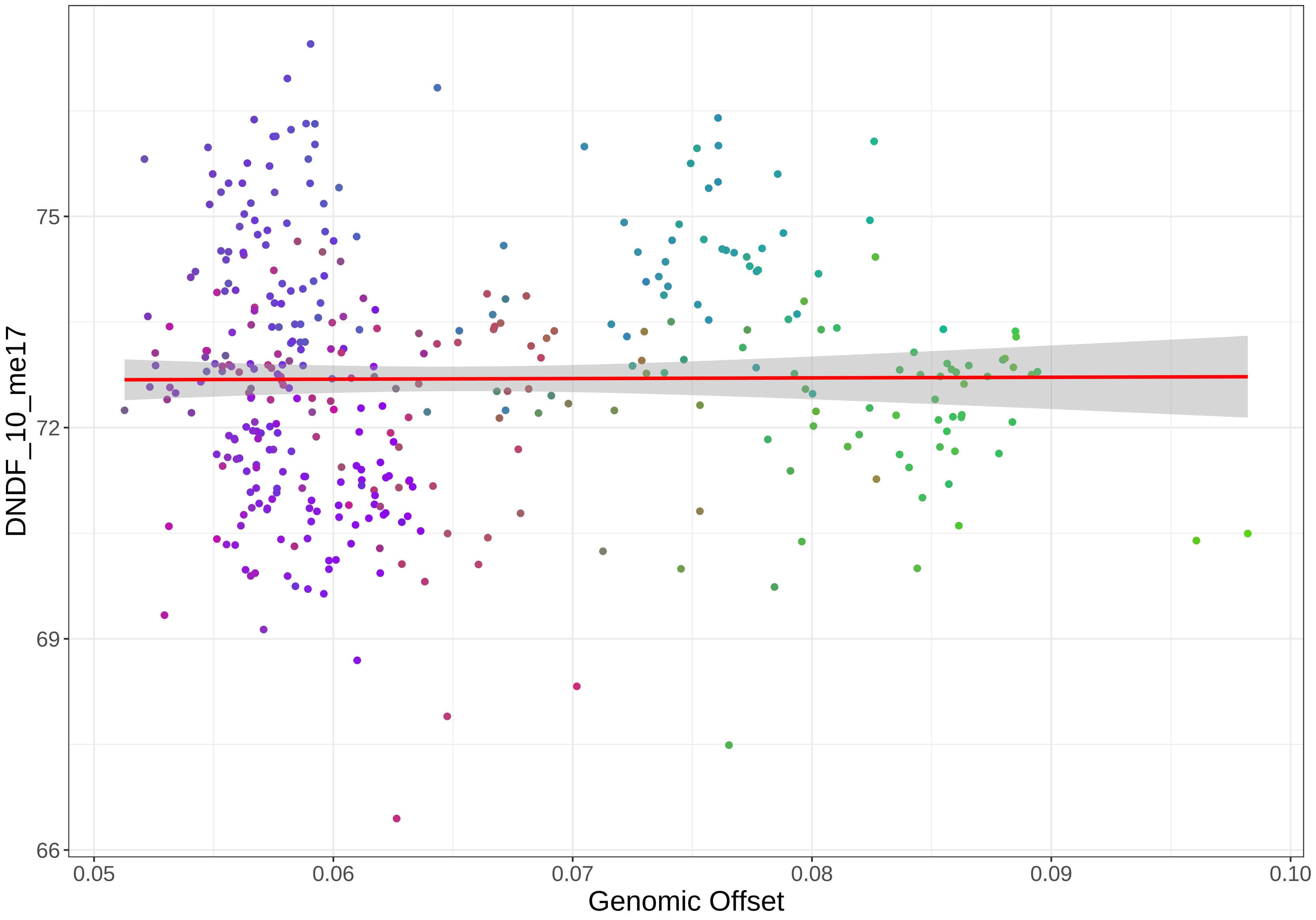

DNDF_10_me17
75
72
69
66
0.05
0.06
0.07
0.08
0.09
0.10
Genomic Offset

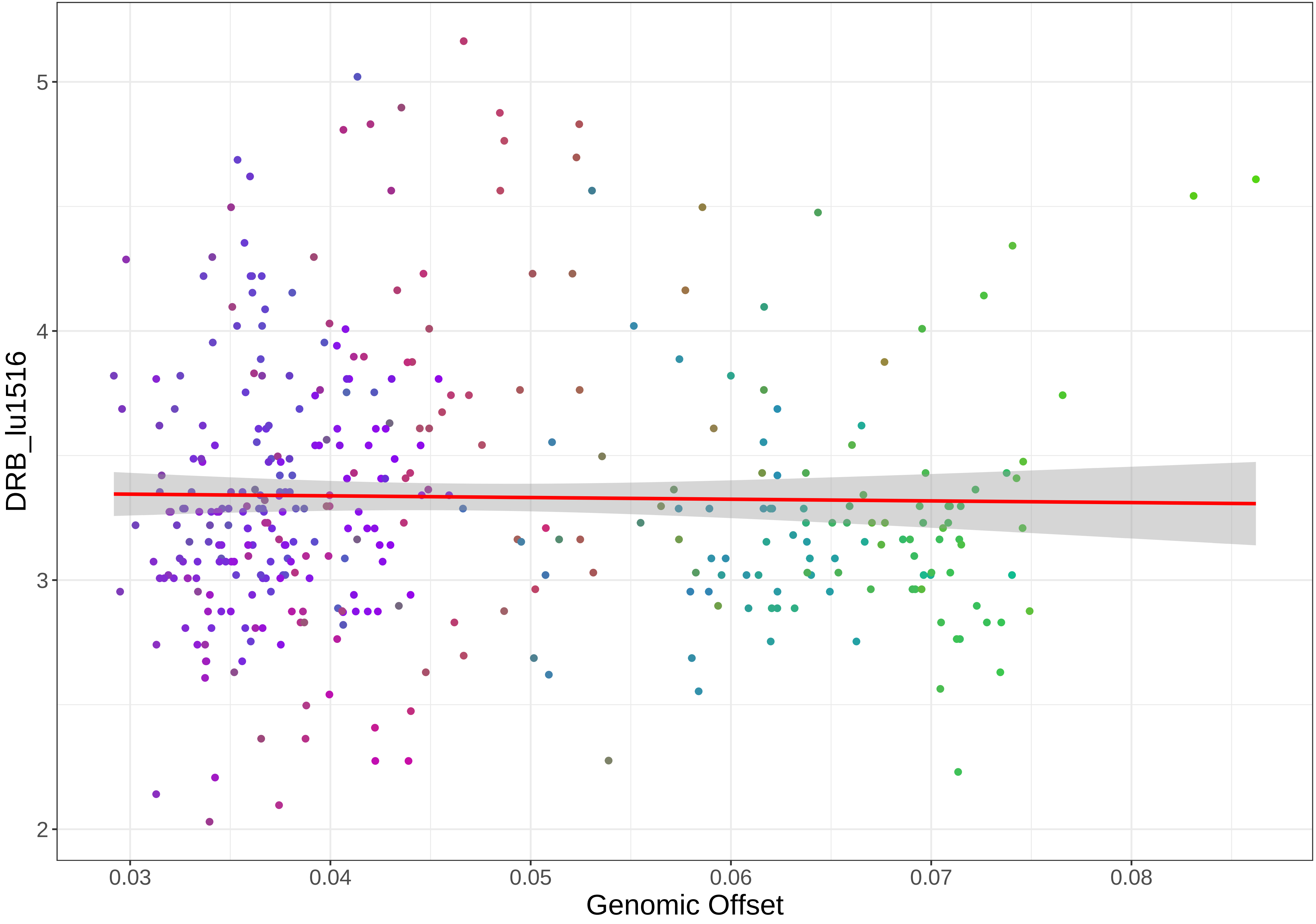
DRB_lu1516
5
4
3
2
0.03
0.04
0.05
0.06
0.07
0.08
Genomic Offset

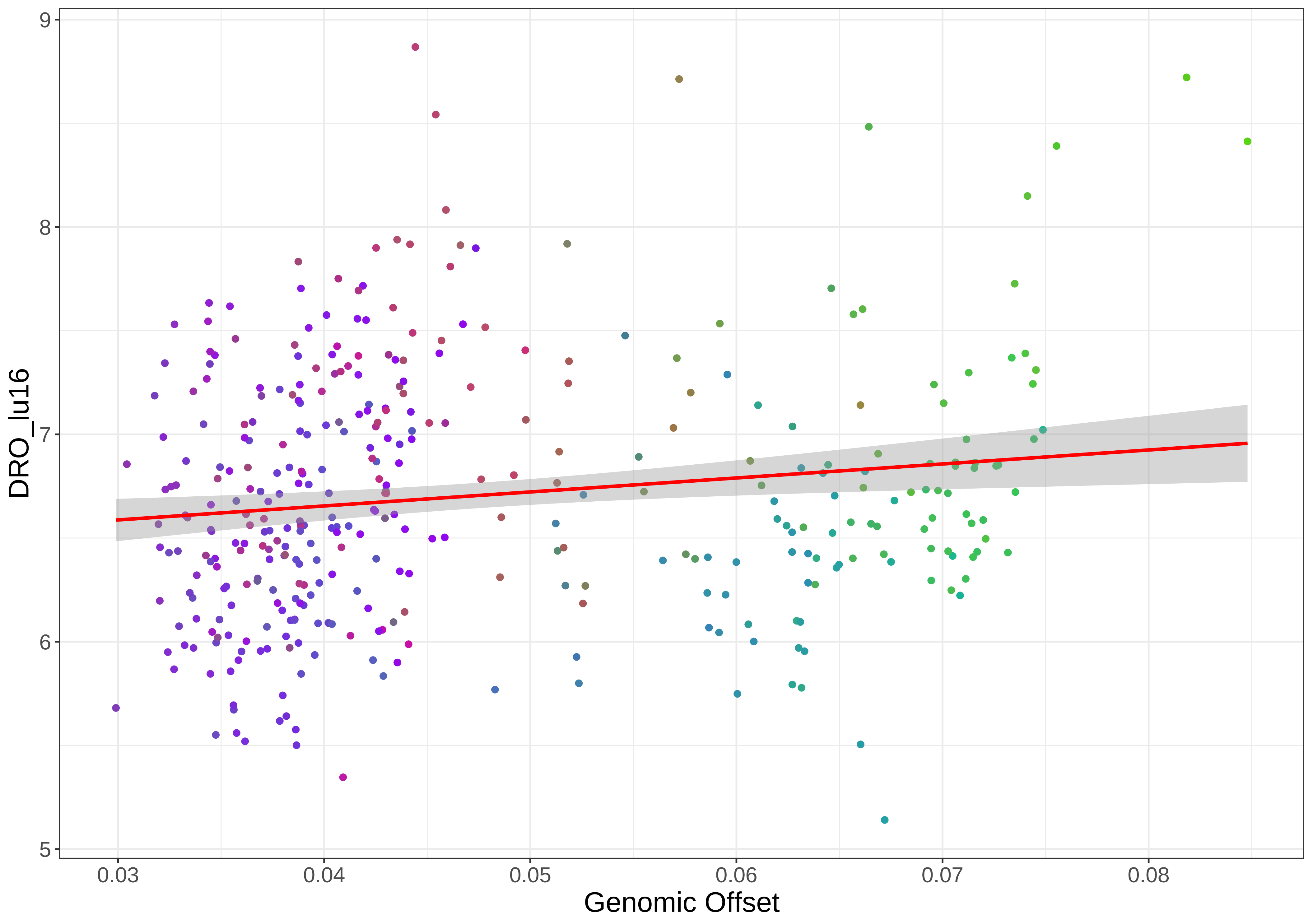
9
8
7
6
5
DRO_lu16
0.03
0.04
0.05
0.06
0.07
0.08
Genomic Offset

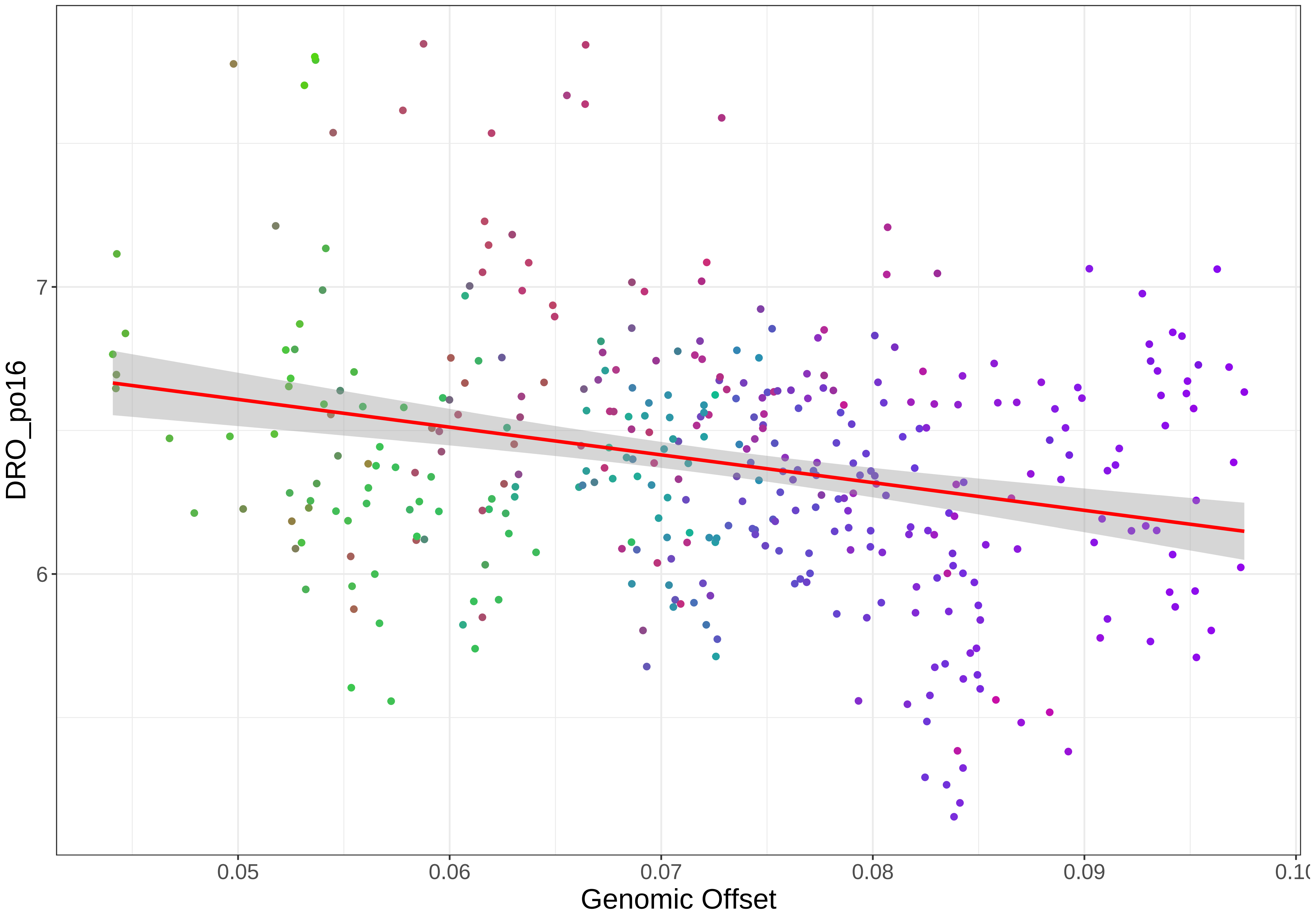

7
6
DRO_po16
0.05
0.06
0.07
0.08
0.09
Genomic Offset

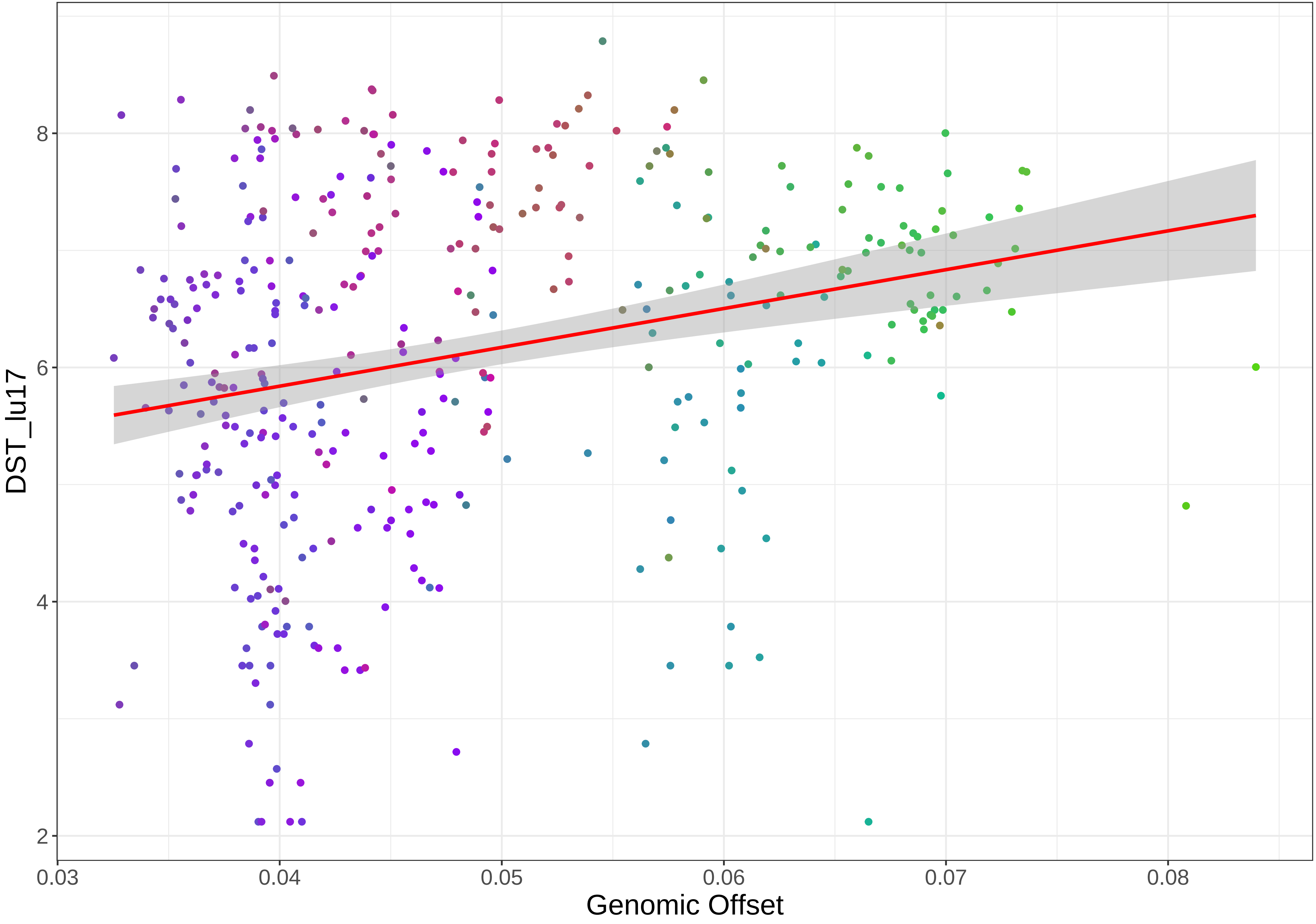
DST_lu17
Genomic Offset
8
6
4
2
0.03
0.04
0.05
0.06
0.07
0.08

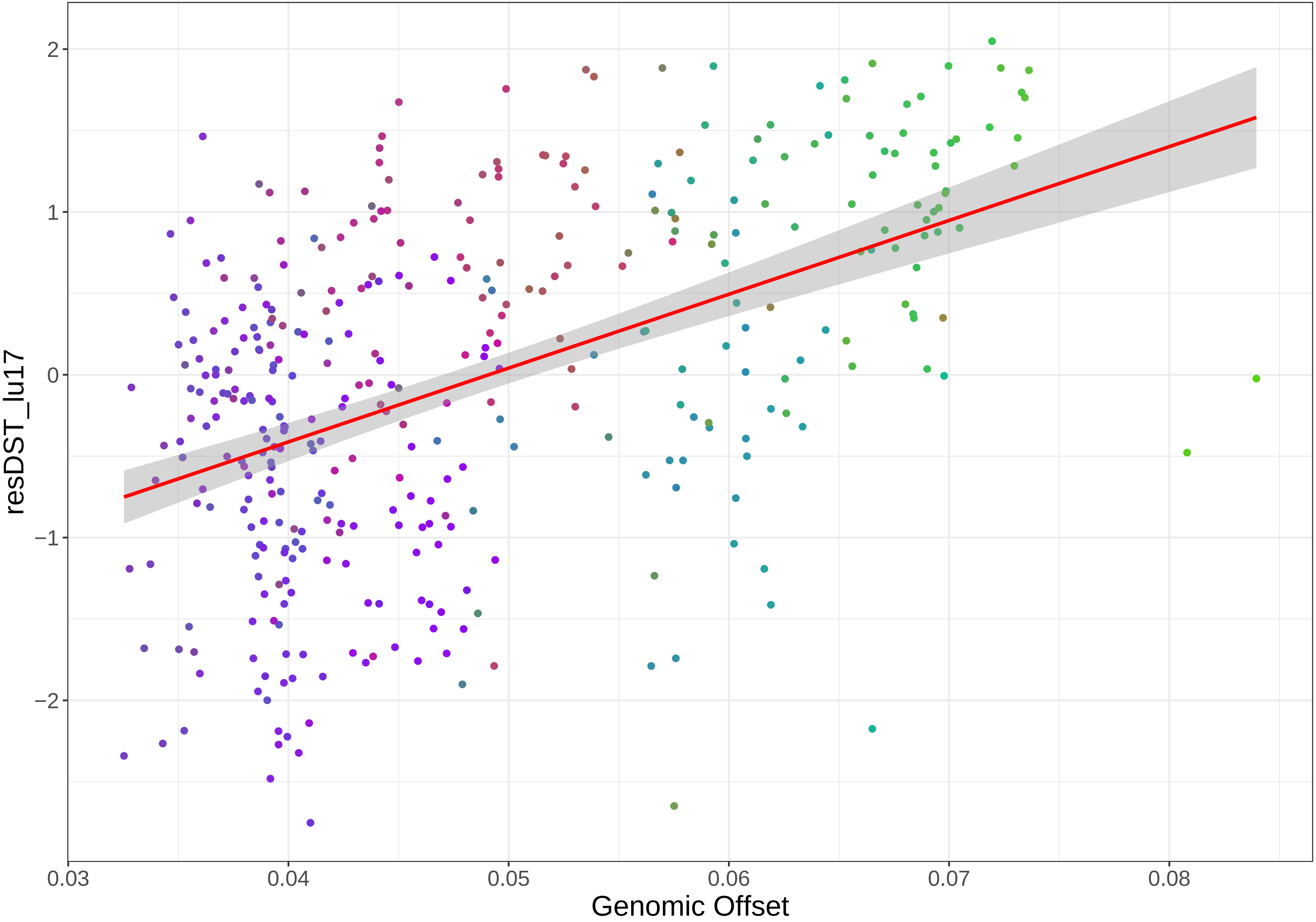

resDST_lu17
Genomic Offset
2
1
0
−1
−2
0.03
0.04
0.05
0.06
0.07
0.08

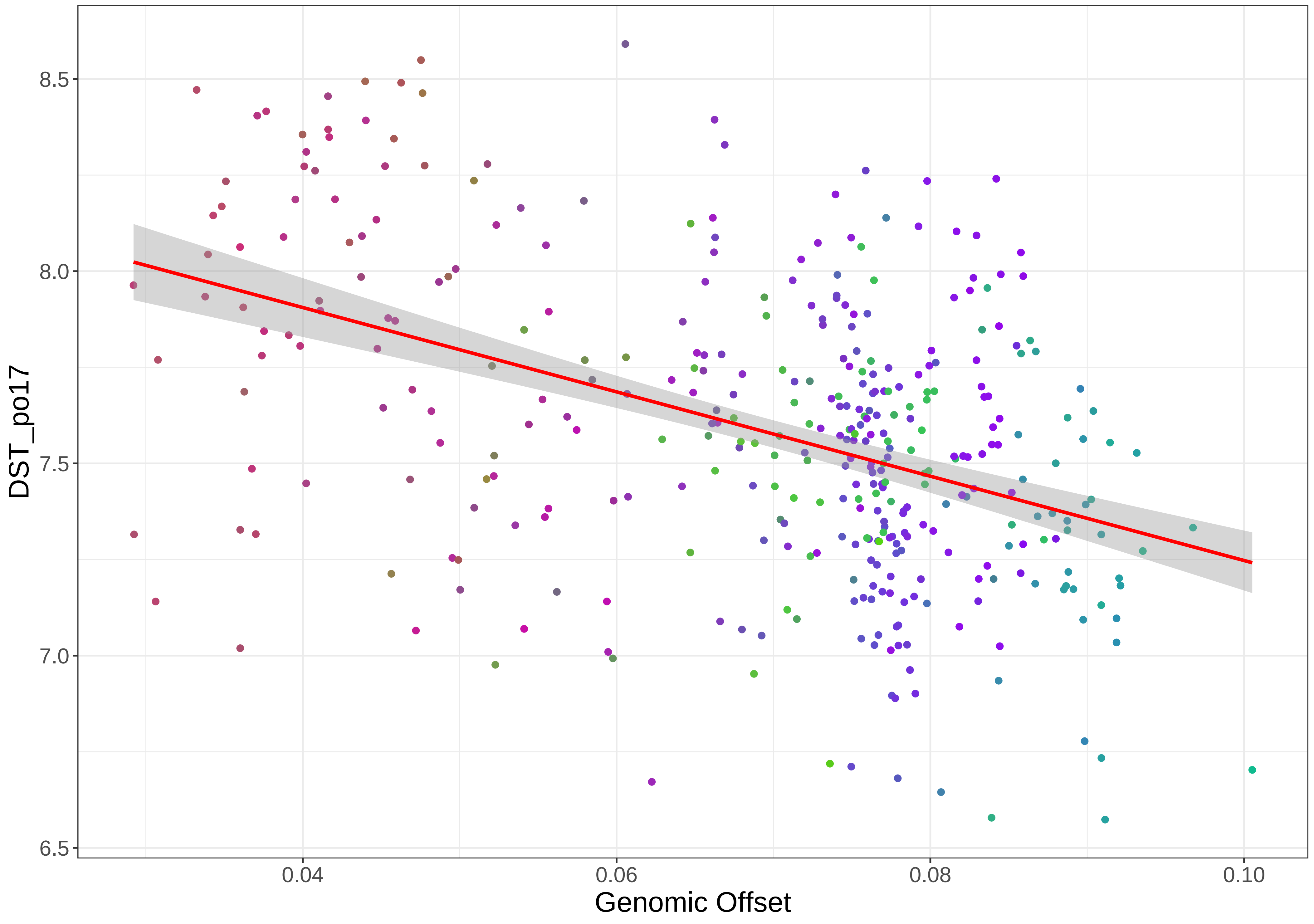
8.5
8.0
7.5
7.0
6.5
DST_po17
0.04
0.06
0.08
0.10
Genomic Offset

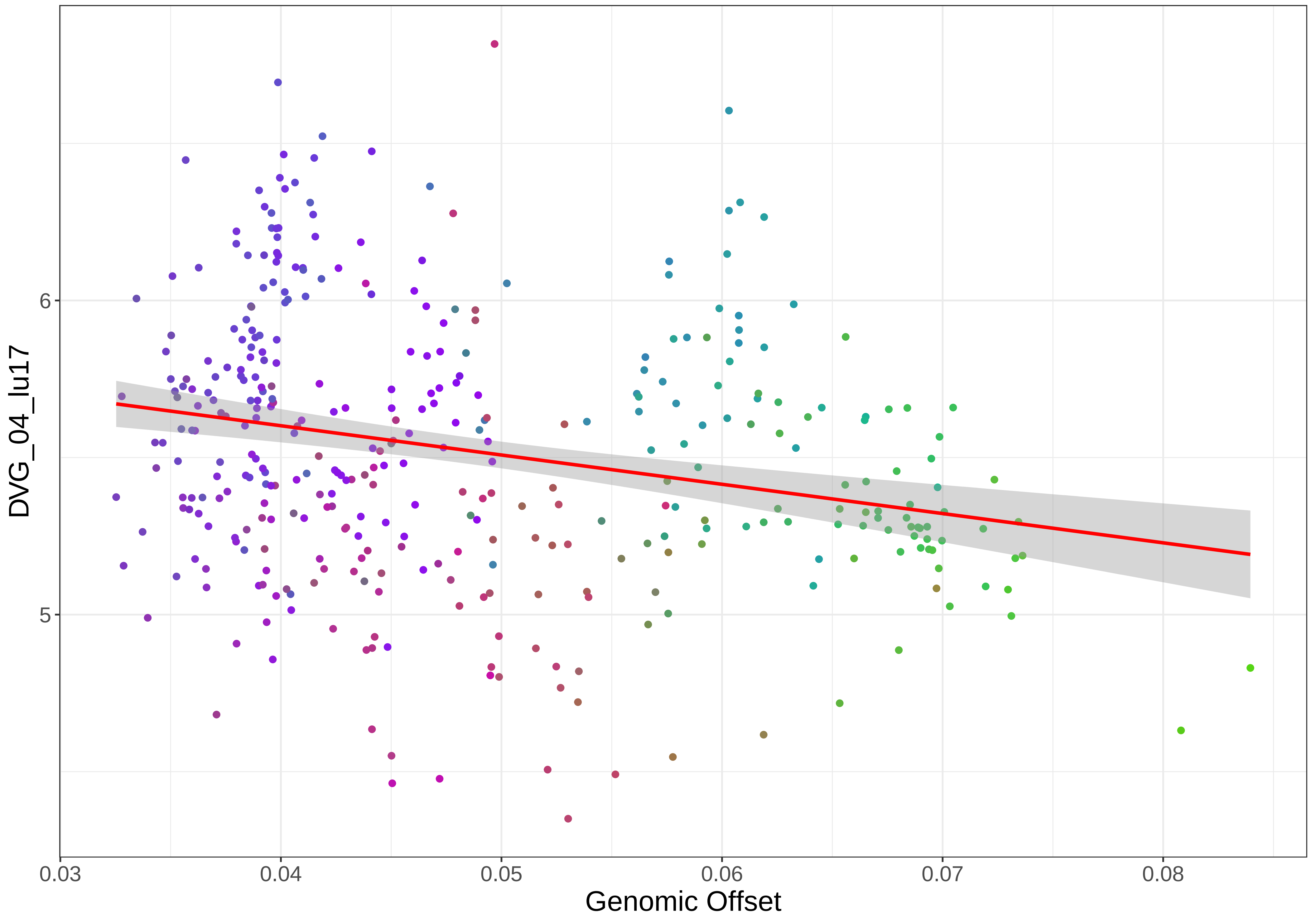

DVG_04_lu17
6
5
0.03
0.04
0.05
0.06
0.07
0.08
Genomic Offset

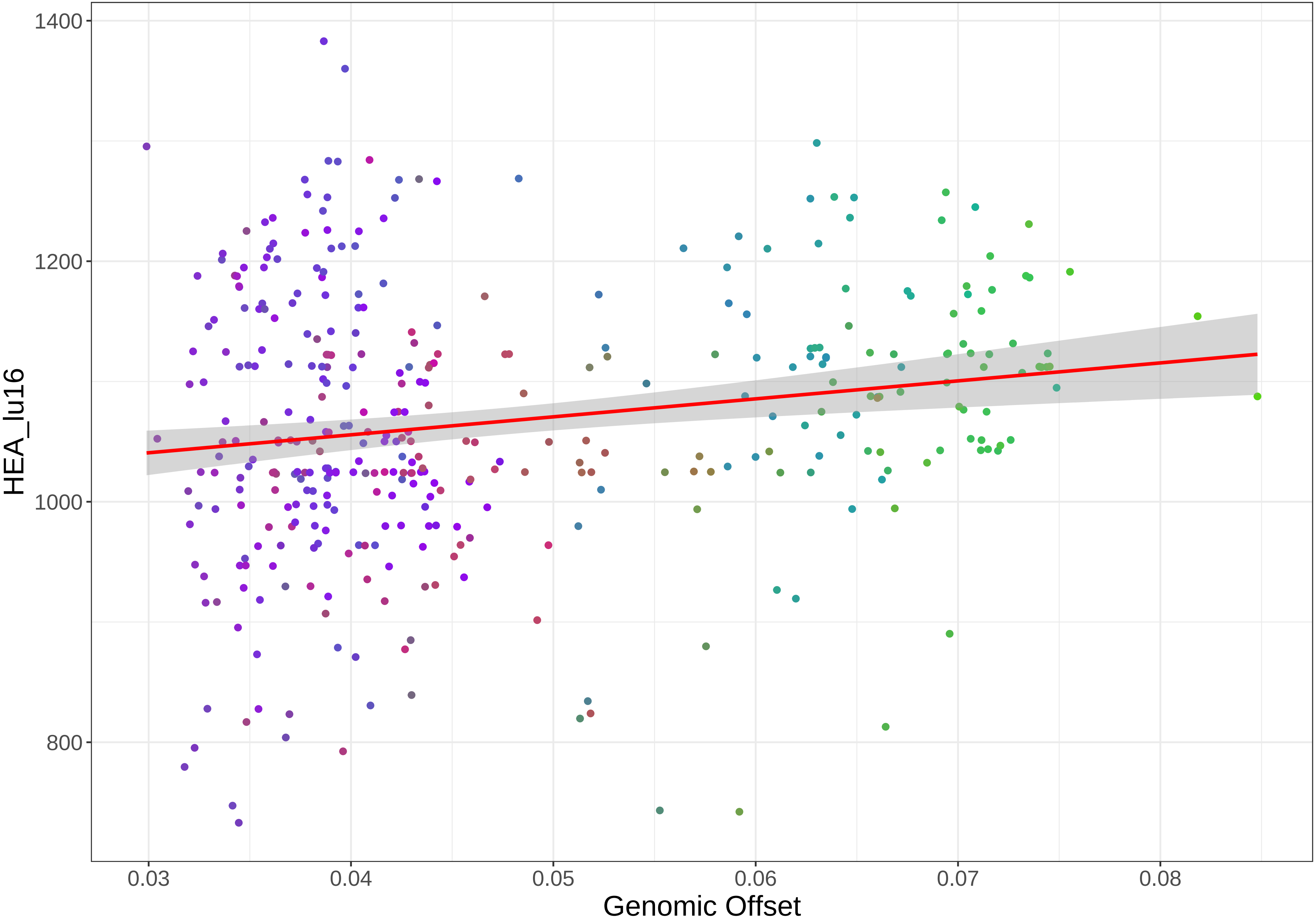

HEA_lu16
1400
1200
1000
800
0.03
0.04
0.05
0.06
0.07
0.08
Genomic Offset

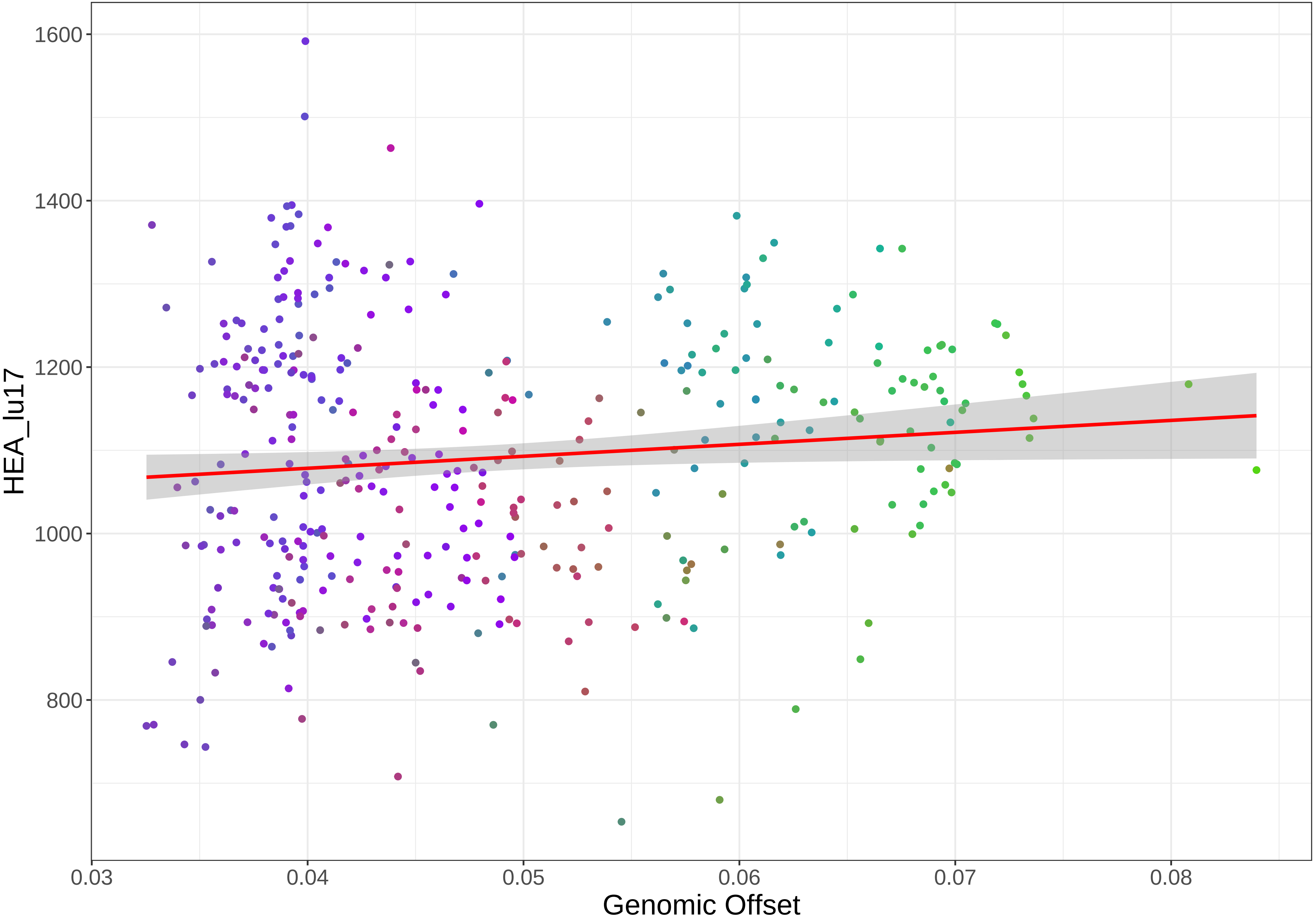

HEA_lu17
1600
1400
1200
1000
800
0.03
0.04
0.05
0.06
0.07
0.08
Genomic Offset

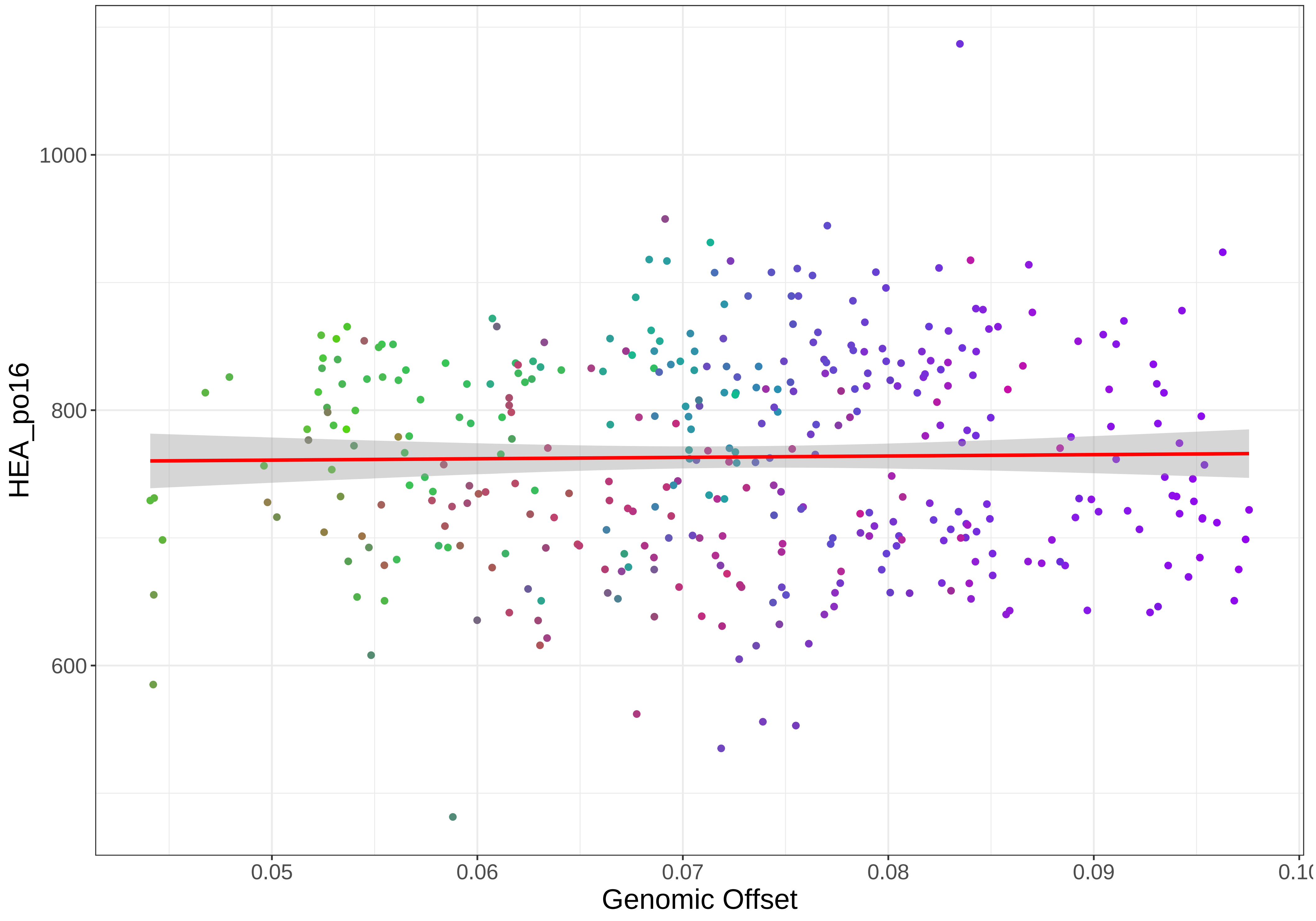

1000
800
600
HEA_po16
0.05
0.06
0.07
0.08
0.09
Genomic Offset

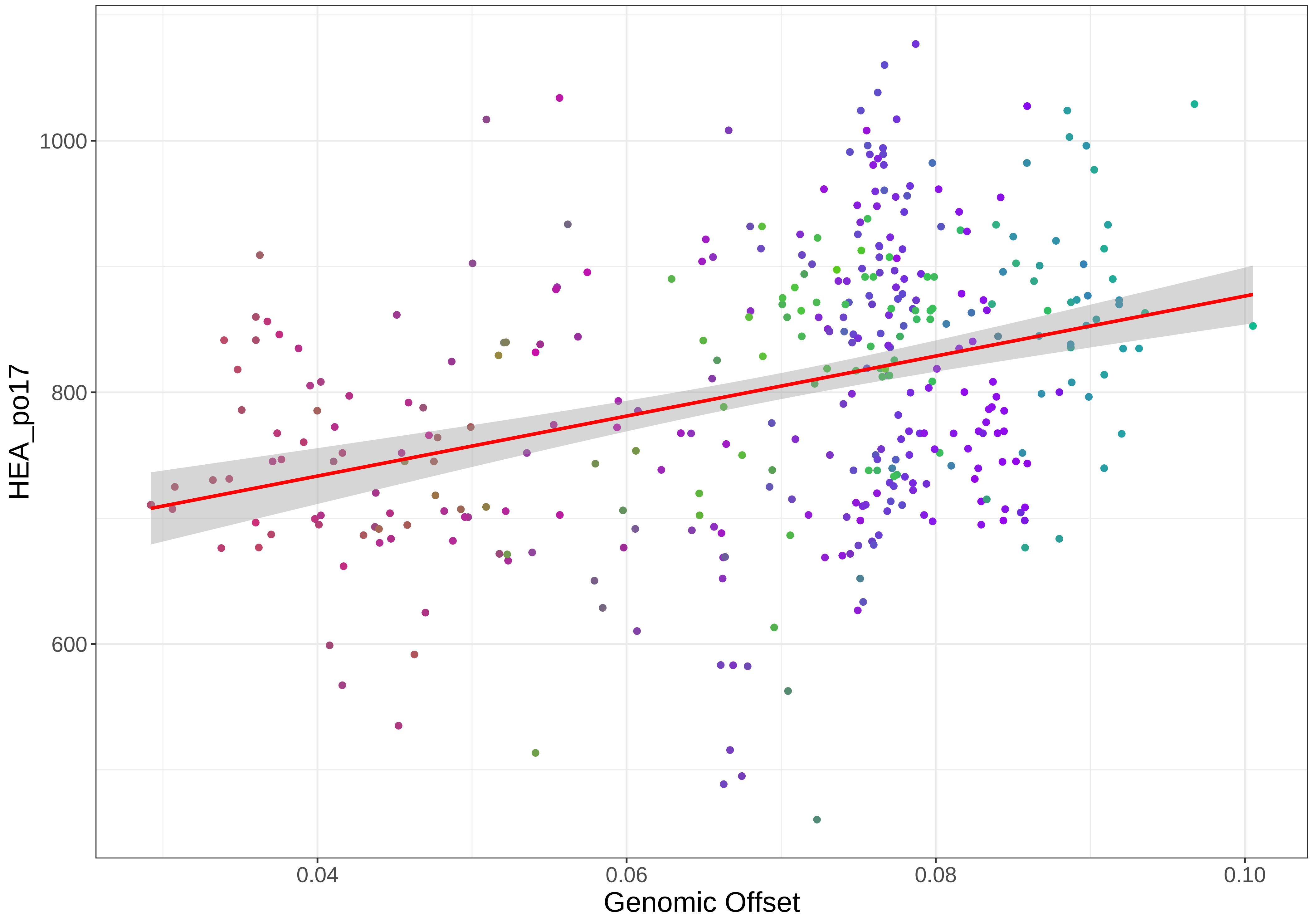
HEA_po17
1000
800
600
0.04
0.06
0.08
0.10
Genomic Offset

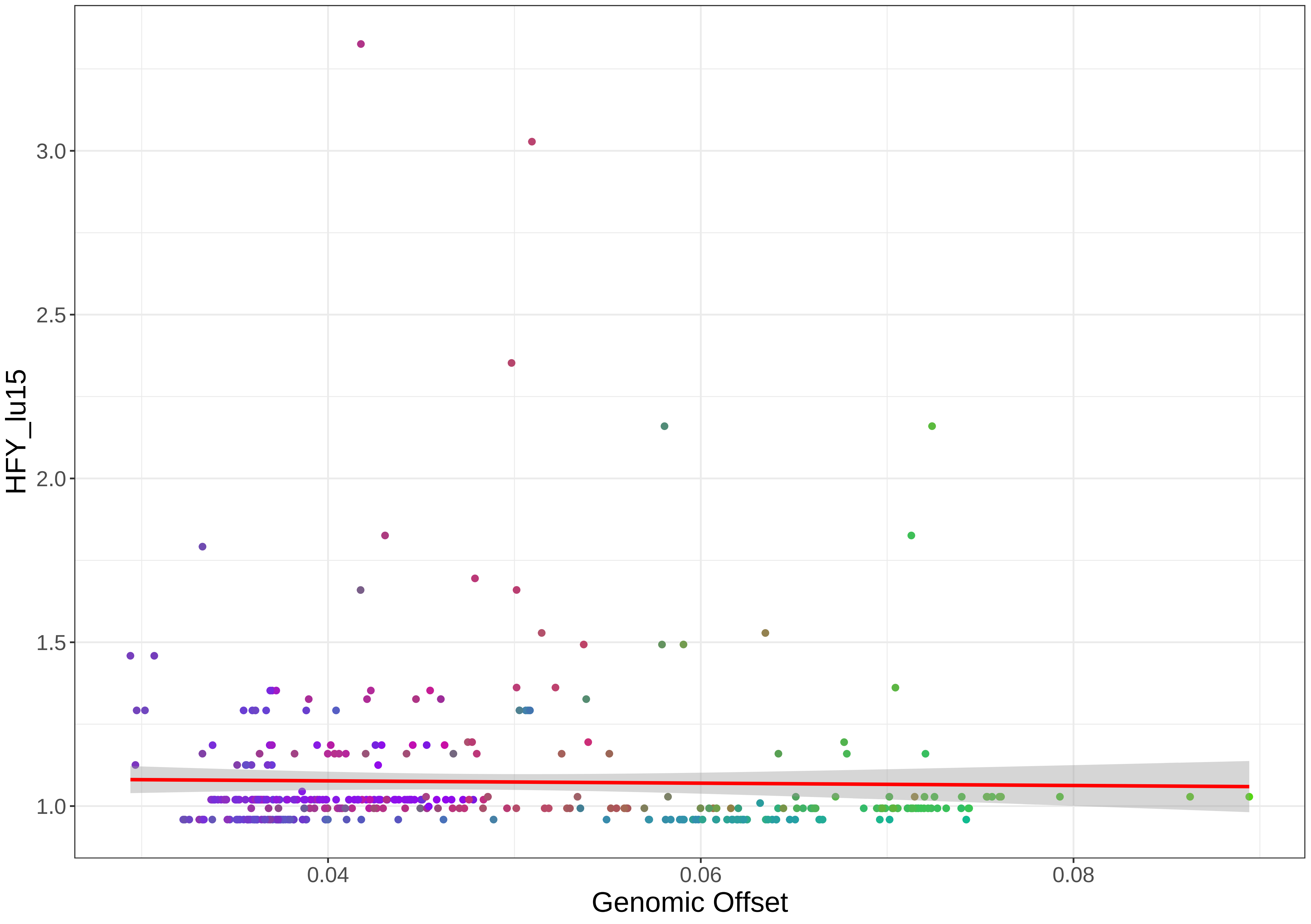

HFY_lu15
3.0
2.5
2.0
1.5
1.0
0.04
0.06
0.08
Genomic Offset

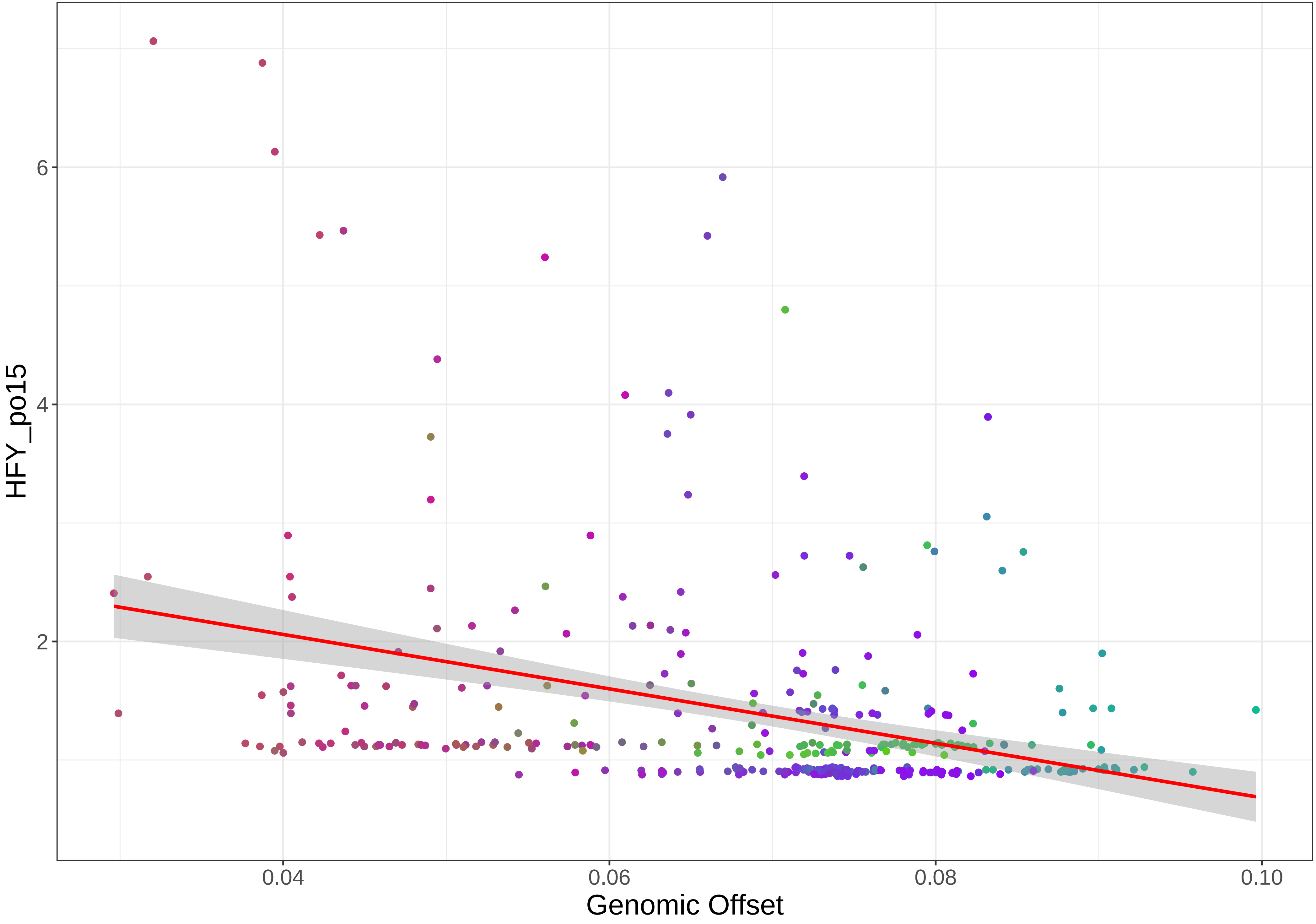

HFY_po15
6
4
2
0.04
0.06
0.08
0.10
Genomic Offset

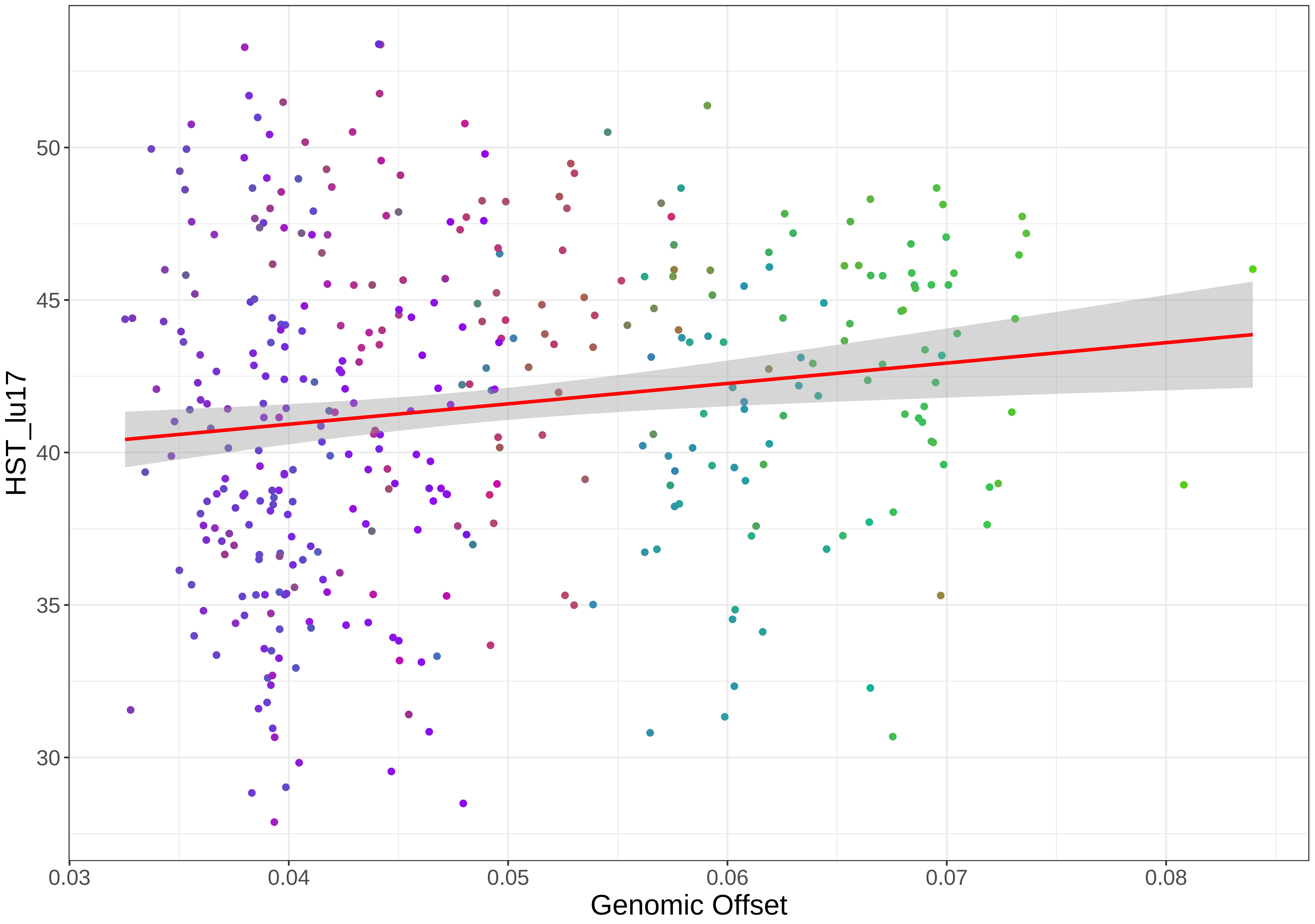

HST_lu17
50
45
40
35
30
0.03
0.04
0.05
0.06
0.07
0.08
Genomic Offset

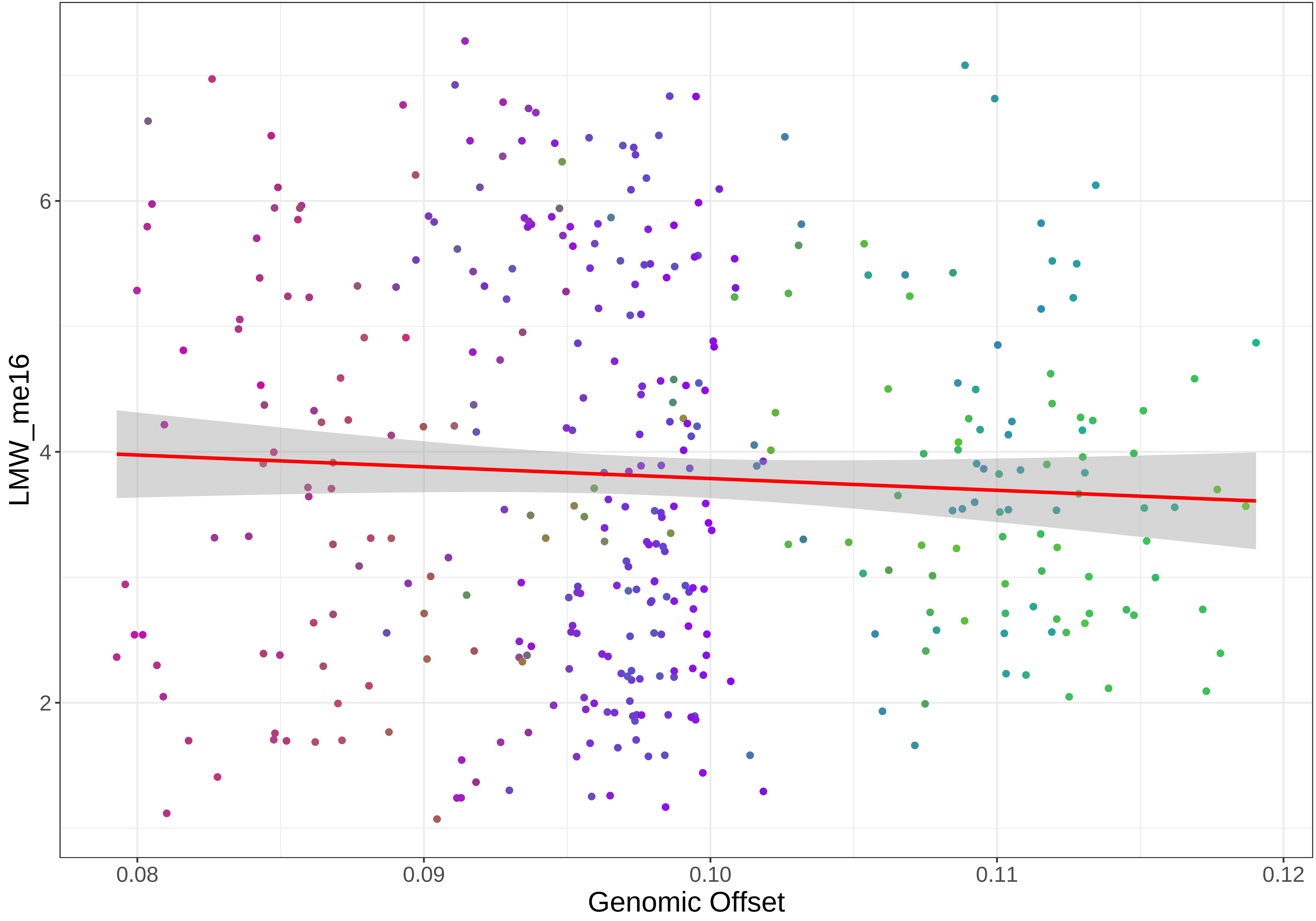

LMW_me16
6
4
2
0.08
0.09
0.10
0.11
0.12
Genomic Offset

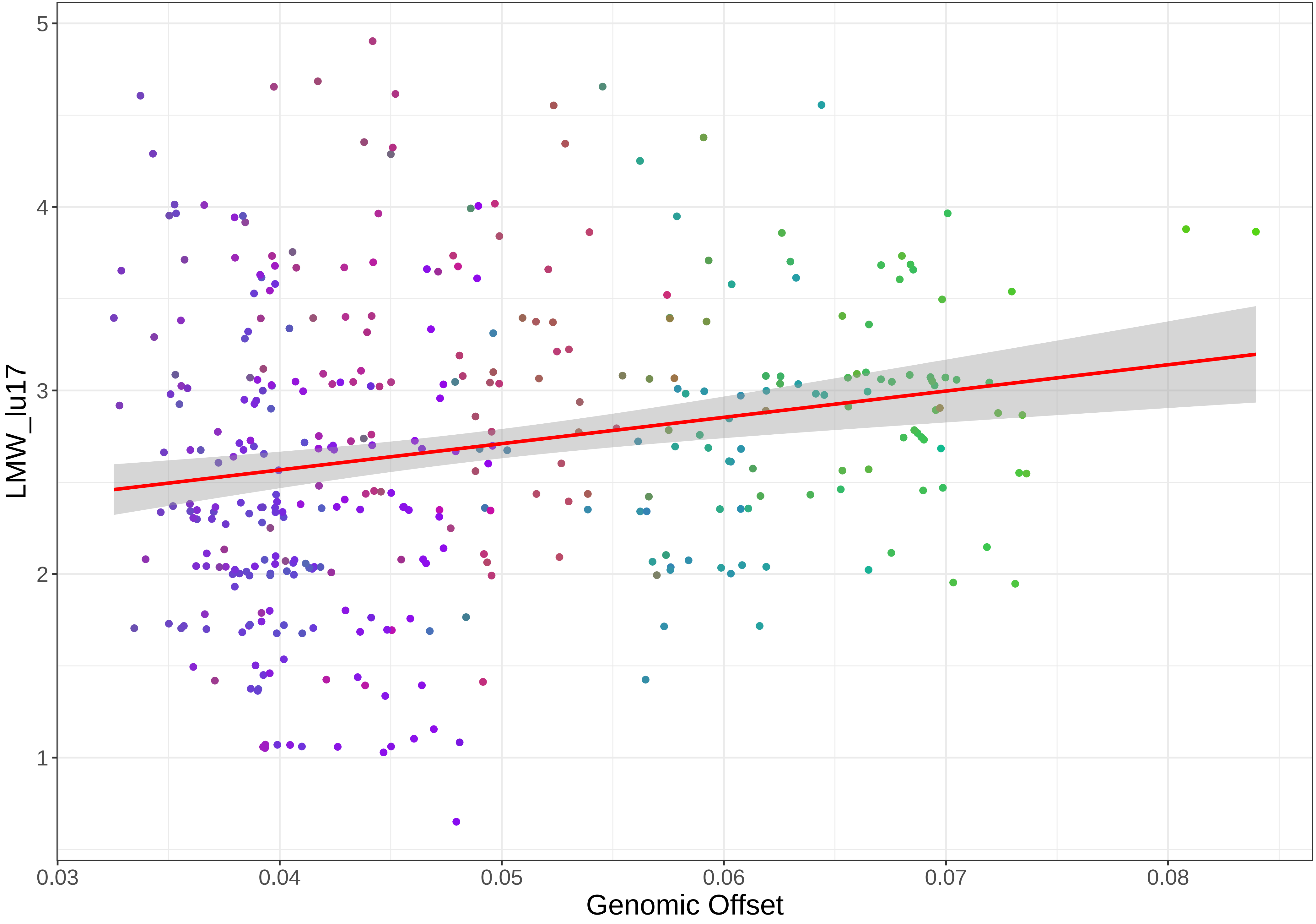

LMW_lu17
5
4
3
2
1
0.03
0.04
0.05
0.06
0.07
0.08
Genomic Offset

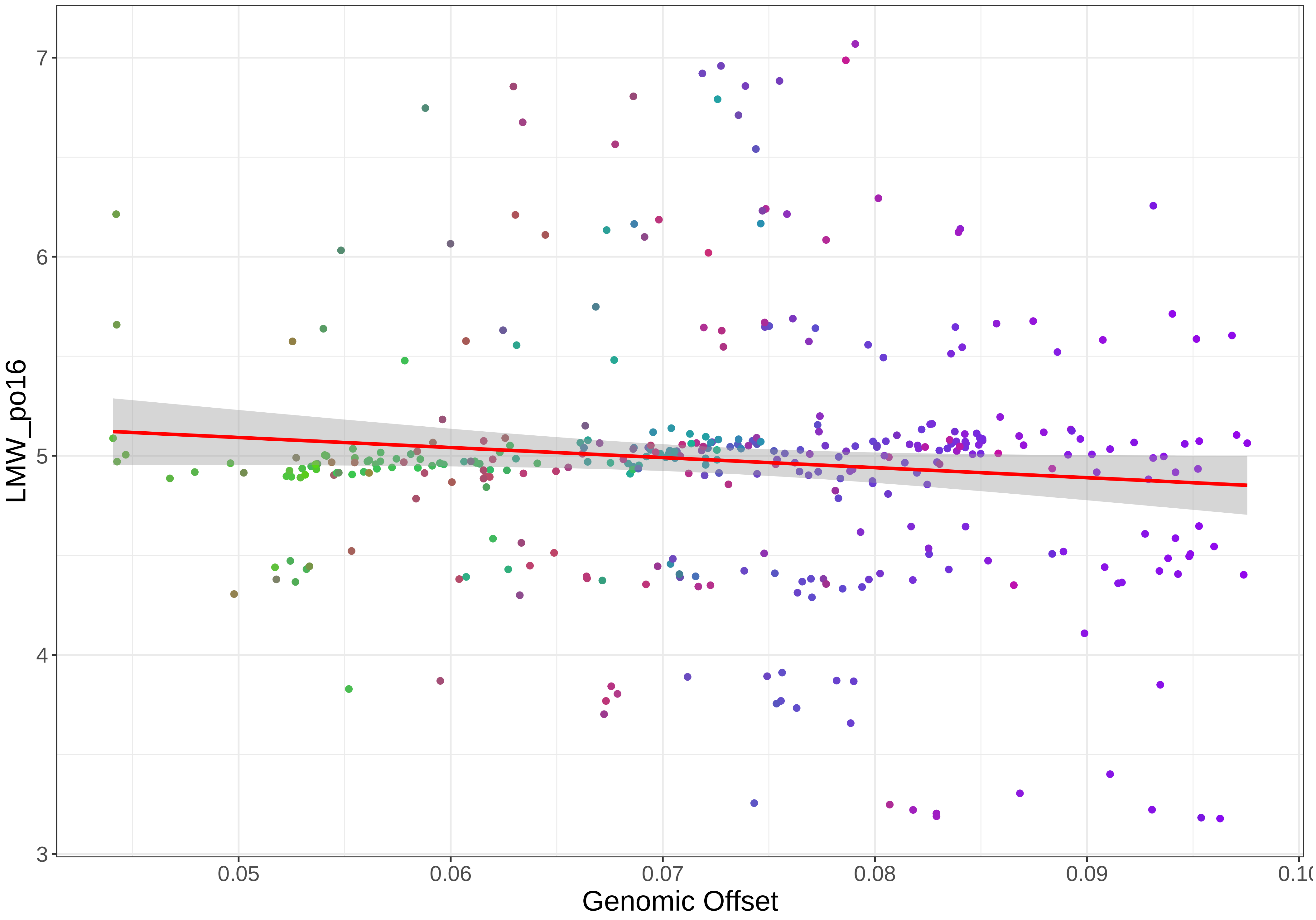

LMW_po16
7
6
5
4
3
0.05
0.06
0.07
0.08
0.09
Genomic Offset

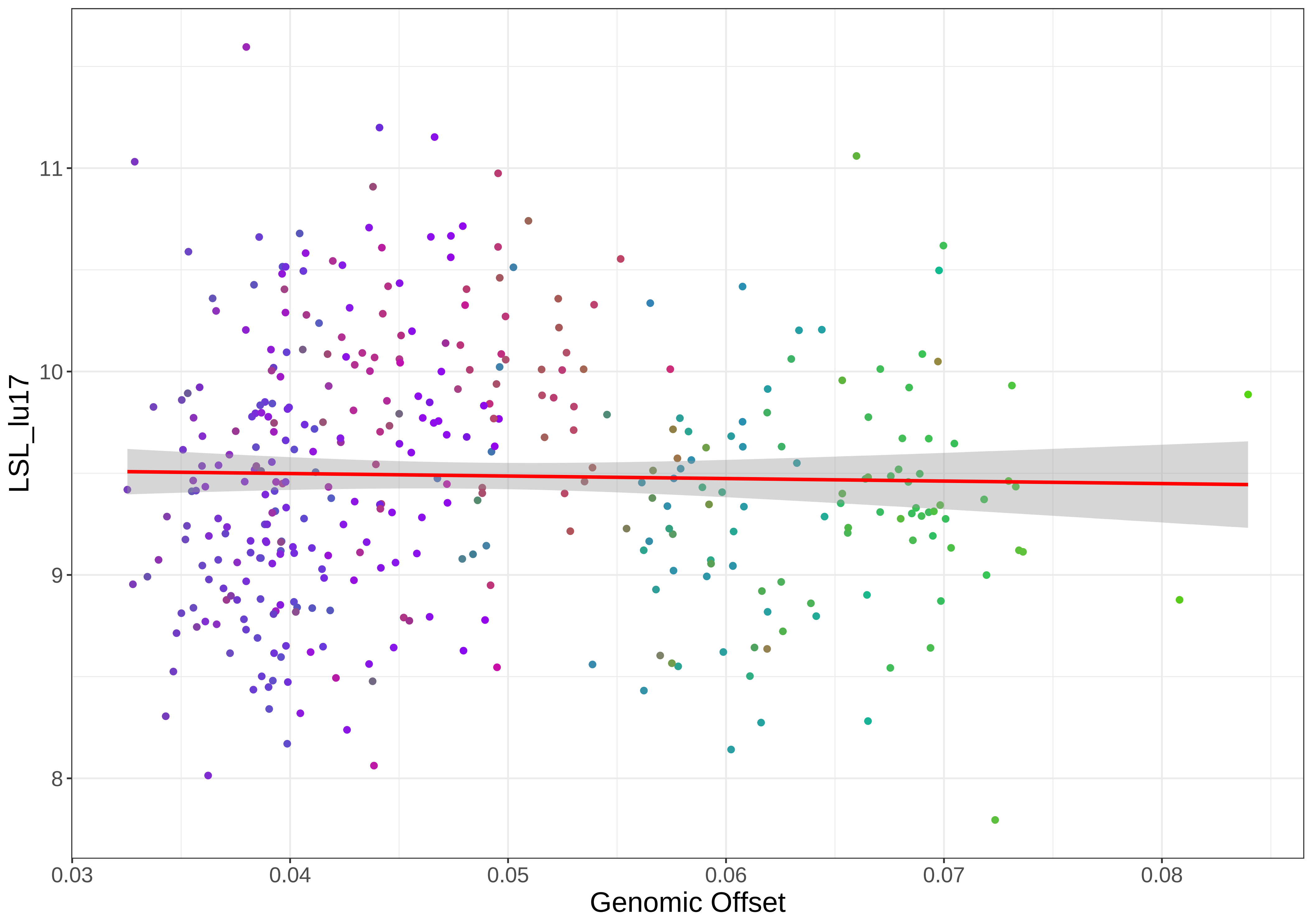

LSL_lu17
11
10
9
8
0.03
0.04
0.05
0.06
0.07
0.08
Genomic Offset

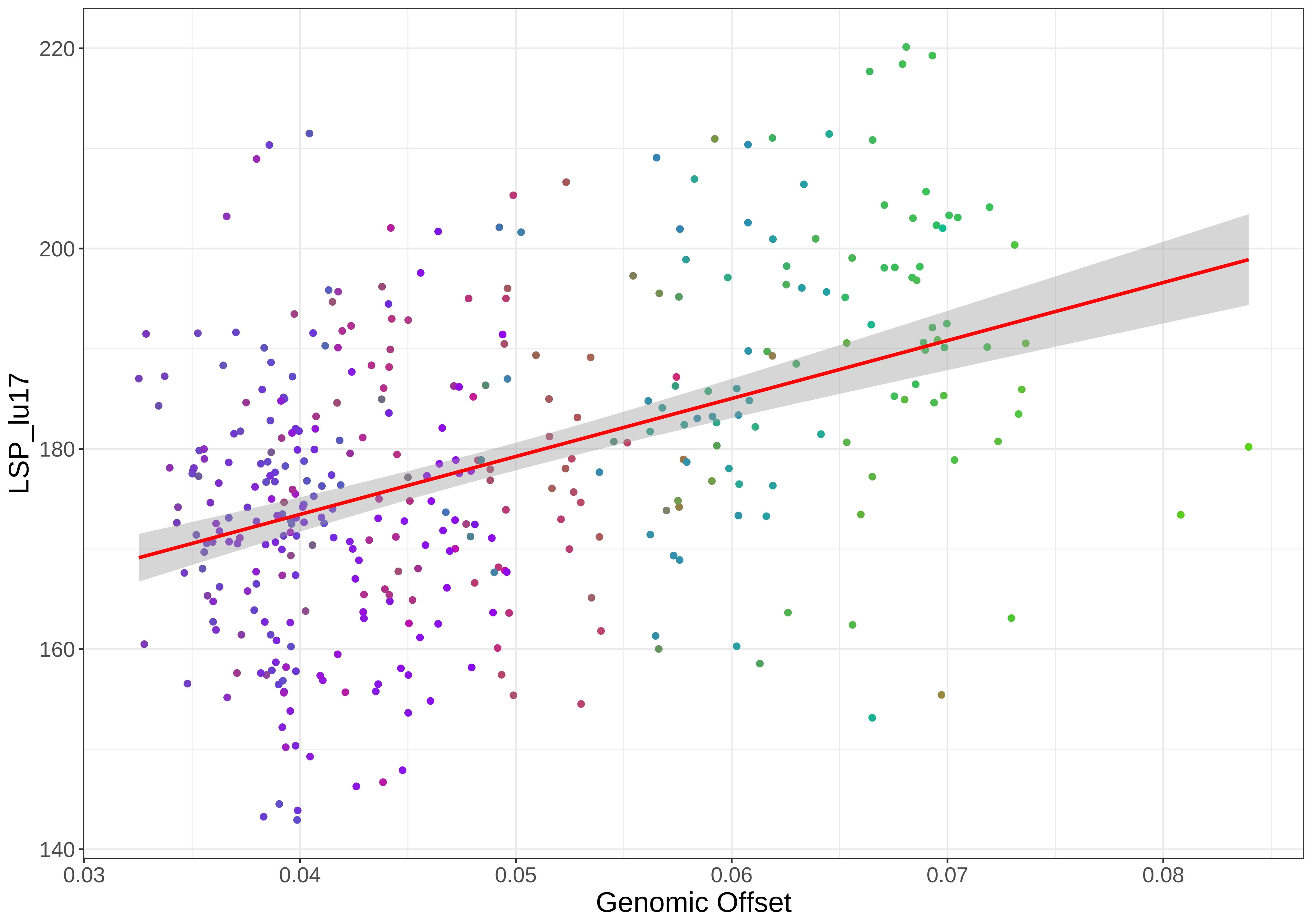
220
200
180
160
140
LSP_lu17
0.03
0.04
0.05
0.06
0.07
0.08
Genomic Offset

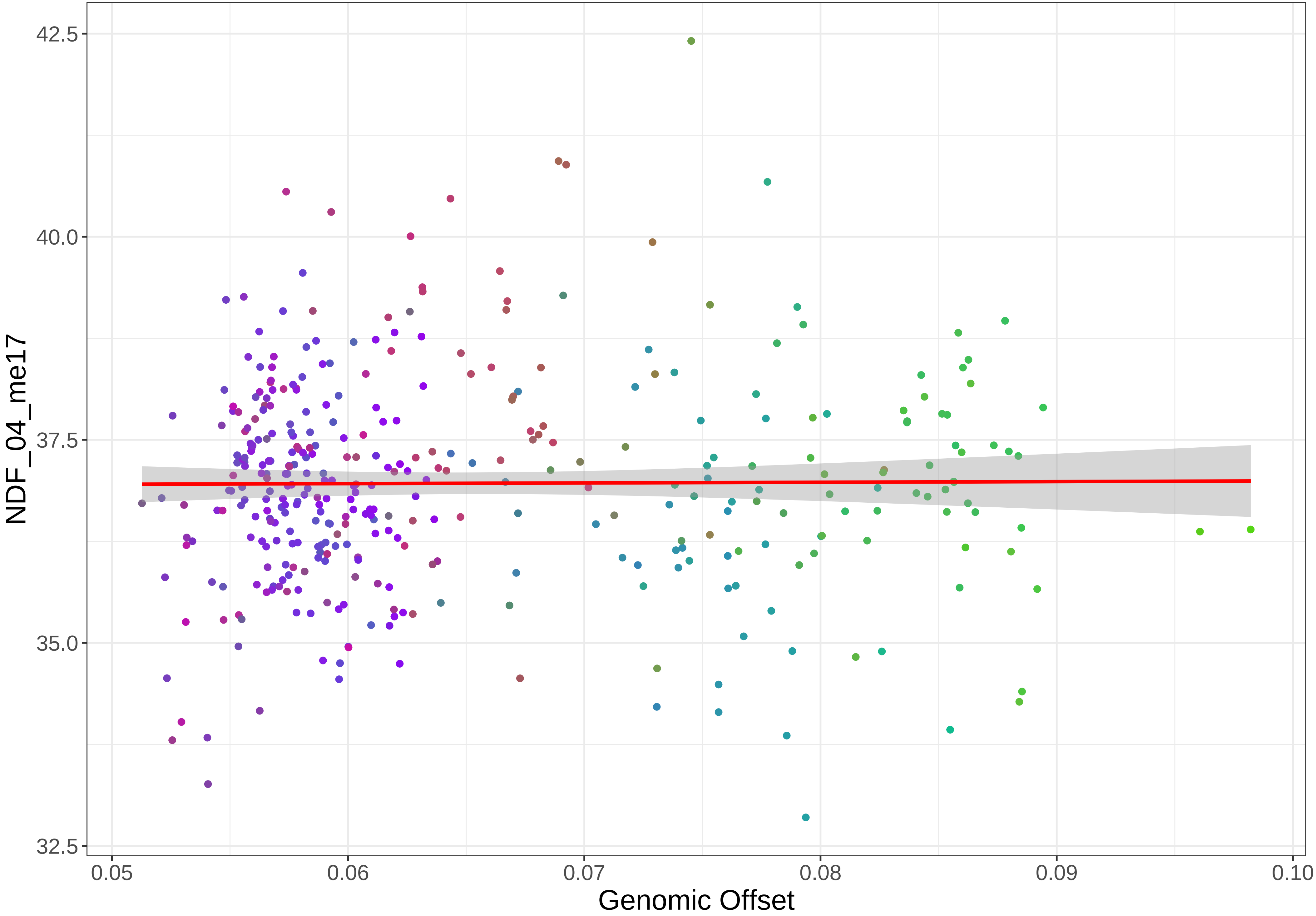

NDF_04_me17
42.5
40.0
37.5
35.0
32.5
0.05
0.06
0.07
0.08
0.09
0.10
Genomic Offset

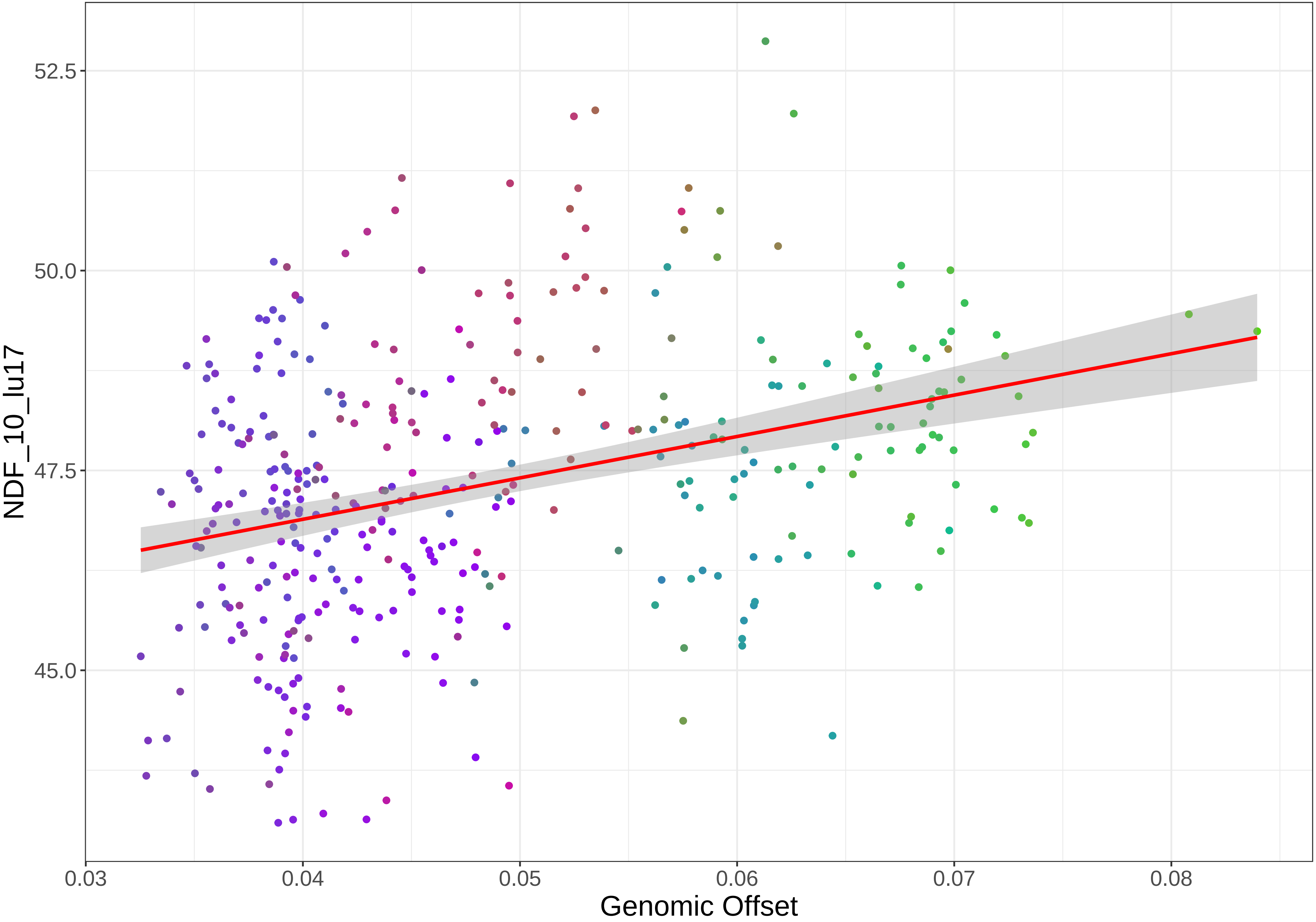

52.5
50.0
47.5
45.0
NDF_10_lu17
0.03
0.04
0.05
0.06
0.07
0.08
Genomic Offset

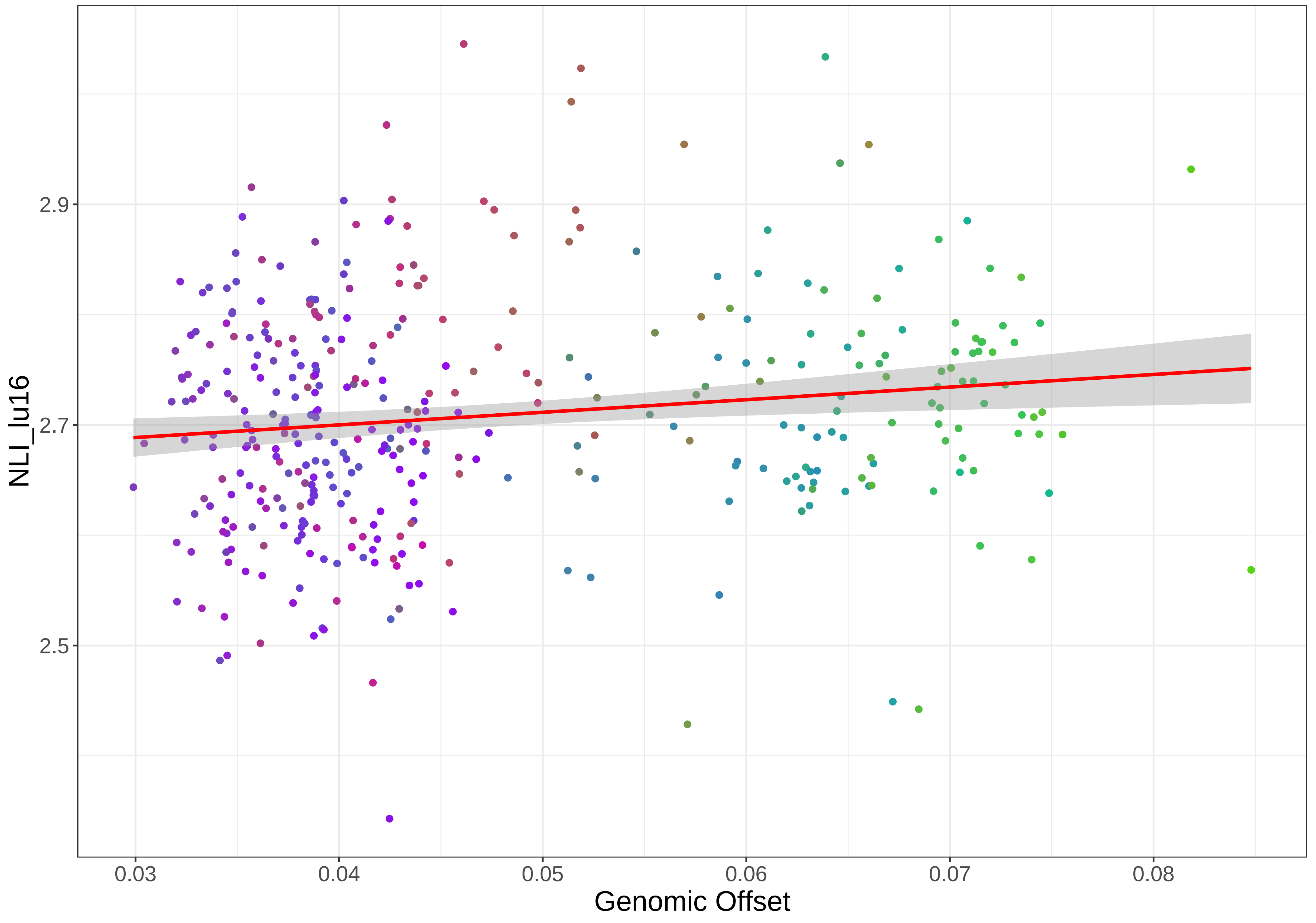

2.9
2.7
2.5
NLI_lu16
0.03
0.04
0.05
0.06
0.07
0.08
Genomic Offset

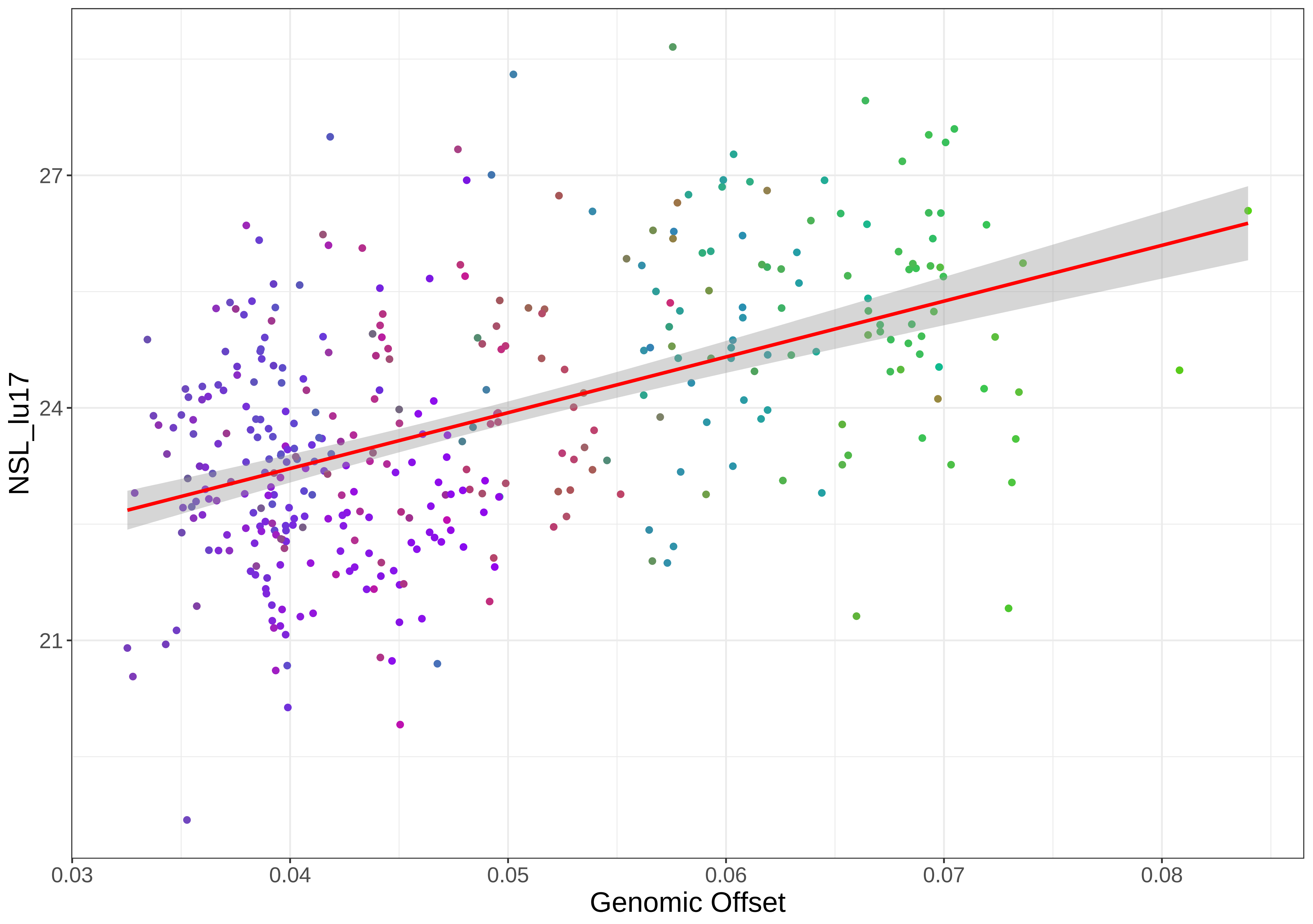

27
24
21
NSL_lu17
0.03
0.04
0.05
0.06
0.07
0.08
Genomic Offset

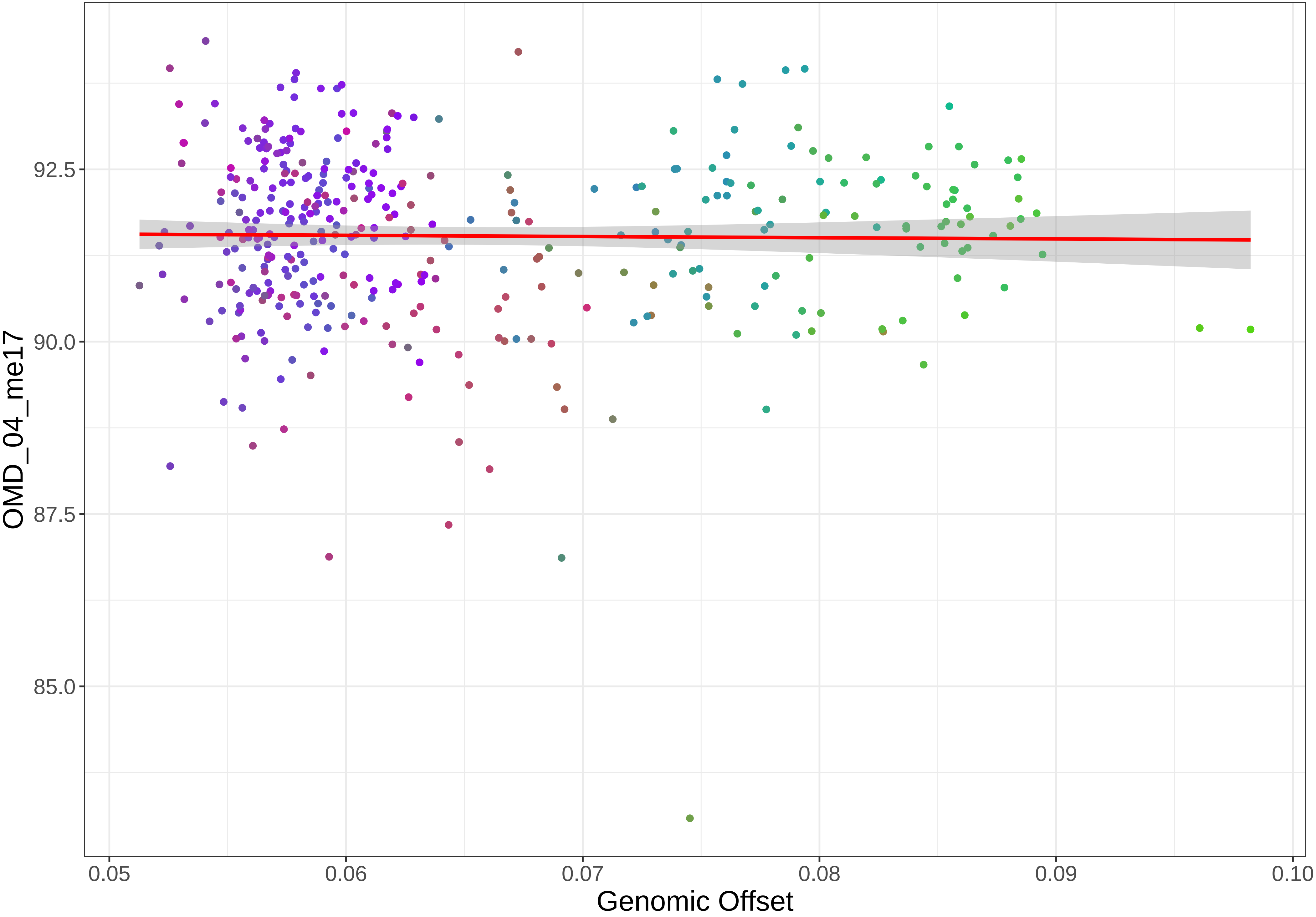
OMD_04_me17
92.5
90.0
87.5
85.0
0.05
0.06
0.07
0.08
0.09
0.10
Genomic Offset

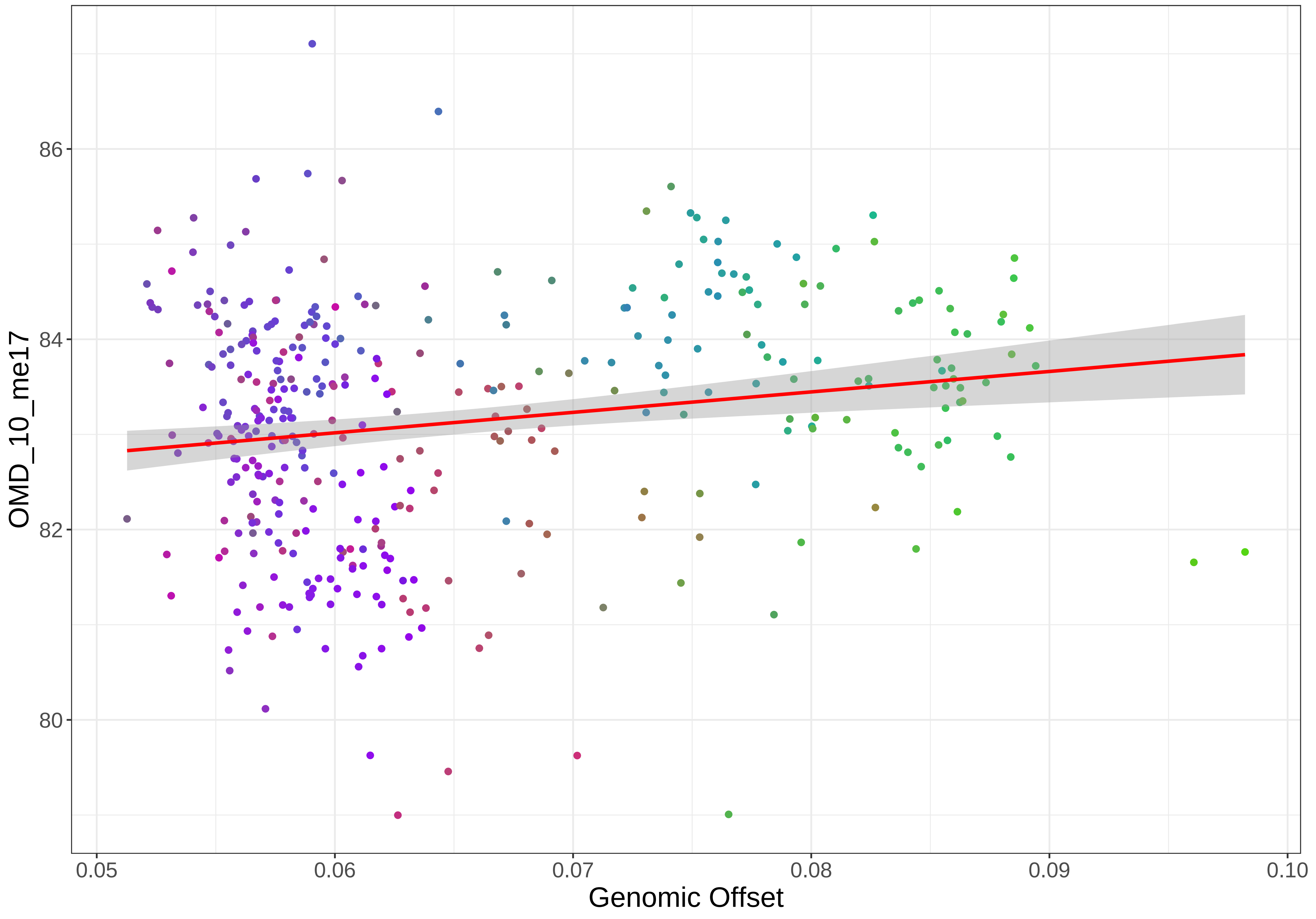
86
84
82
80
OMD_10_me17
0.05
0.06
0.07
0.08
0.09
0.10
Genomic Offset

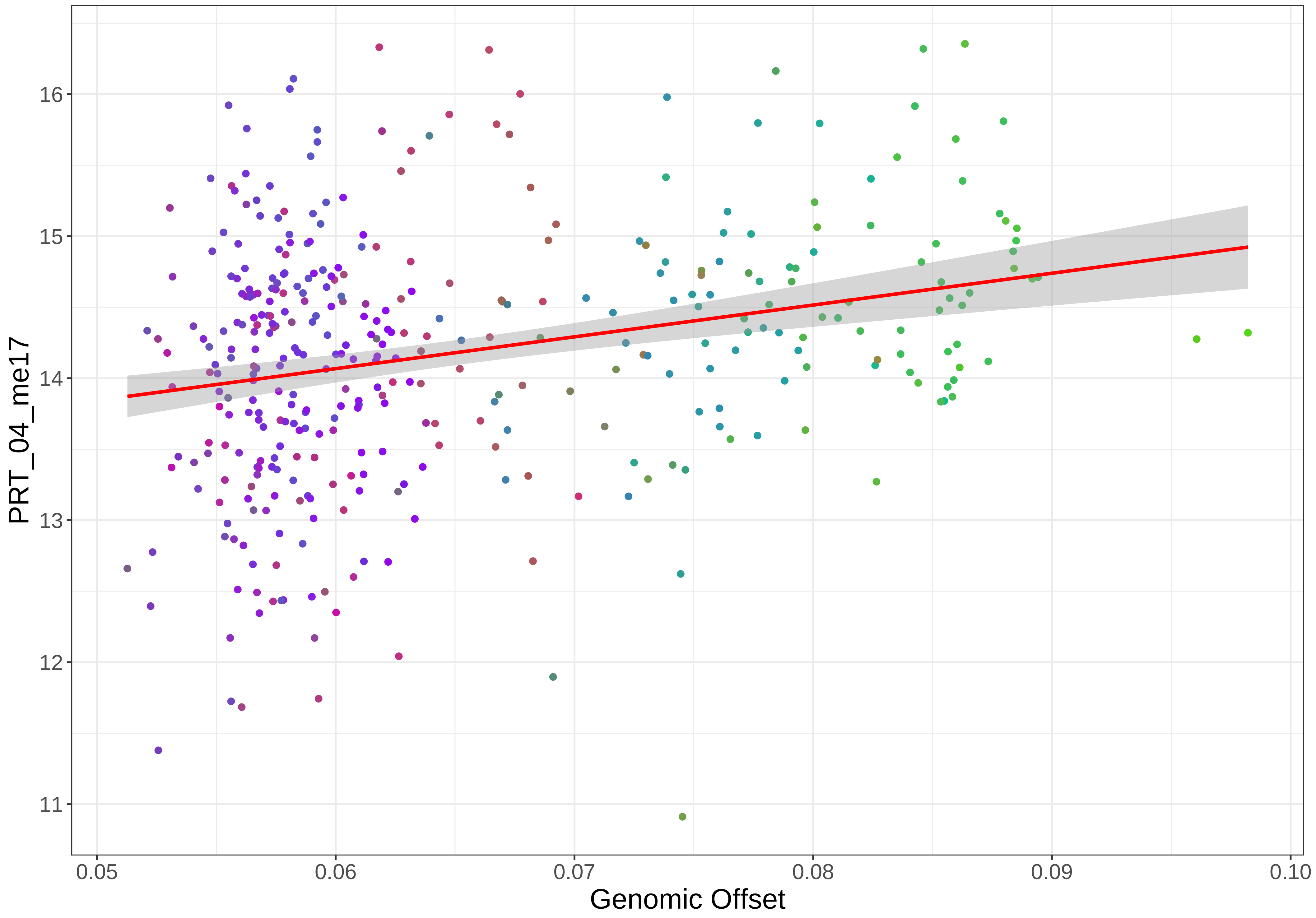

PRT_04_me17
Genomic Offset
11
12
13
14
15
16
0.05
0.06
0.07
0.08
0.09
0.10

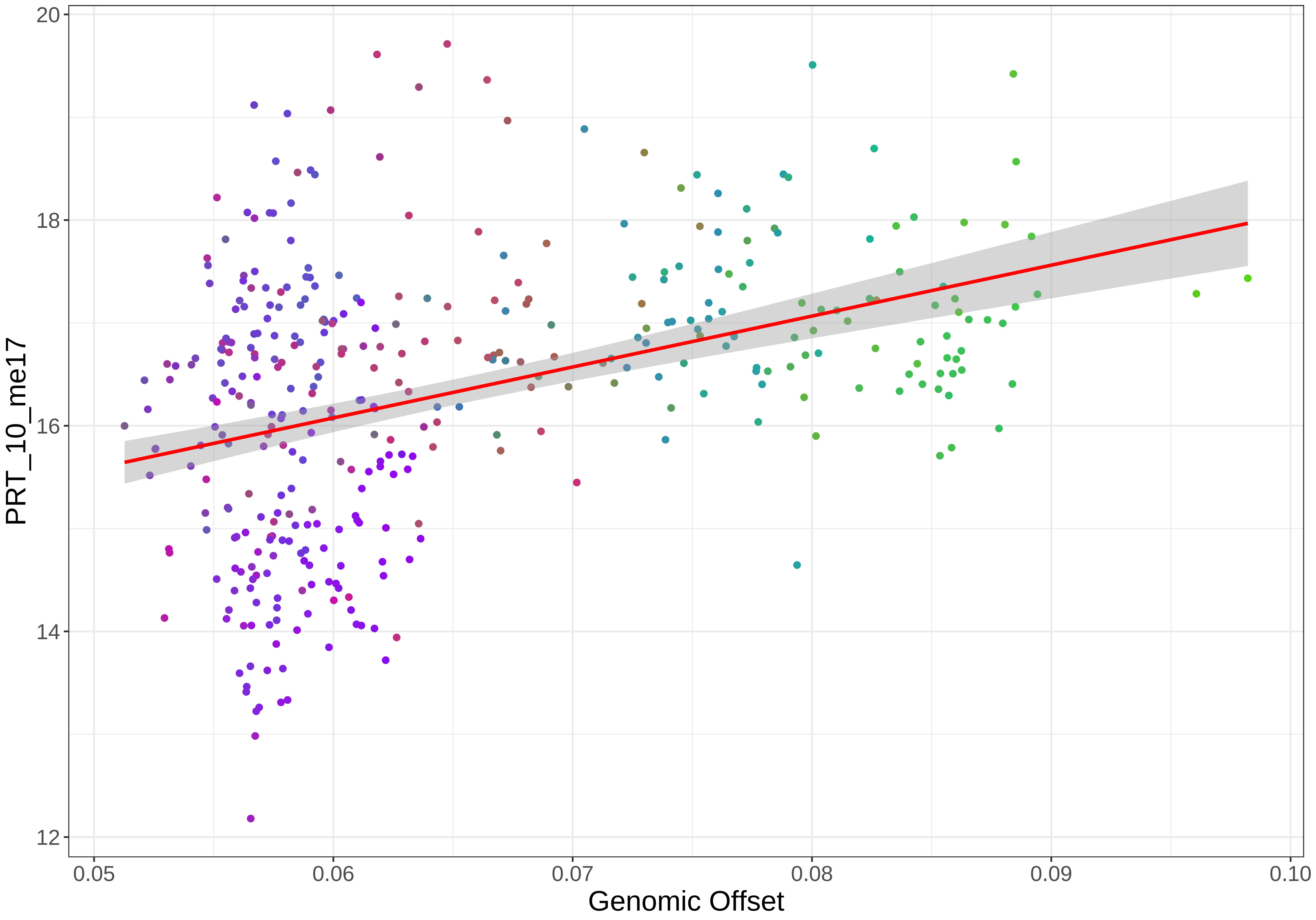
PRT_10_me17
20
18
16
14
12
0.05
0.06
0.07
0.08
0.09
0.10
Genomic Offset

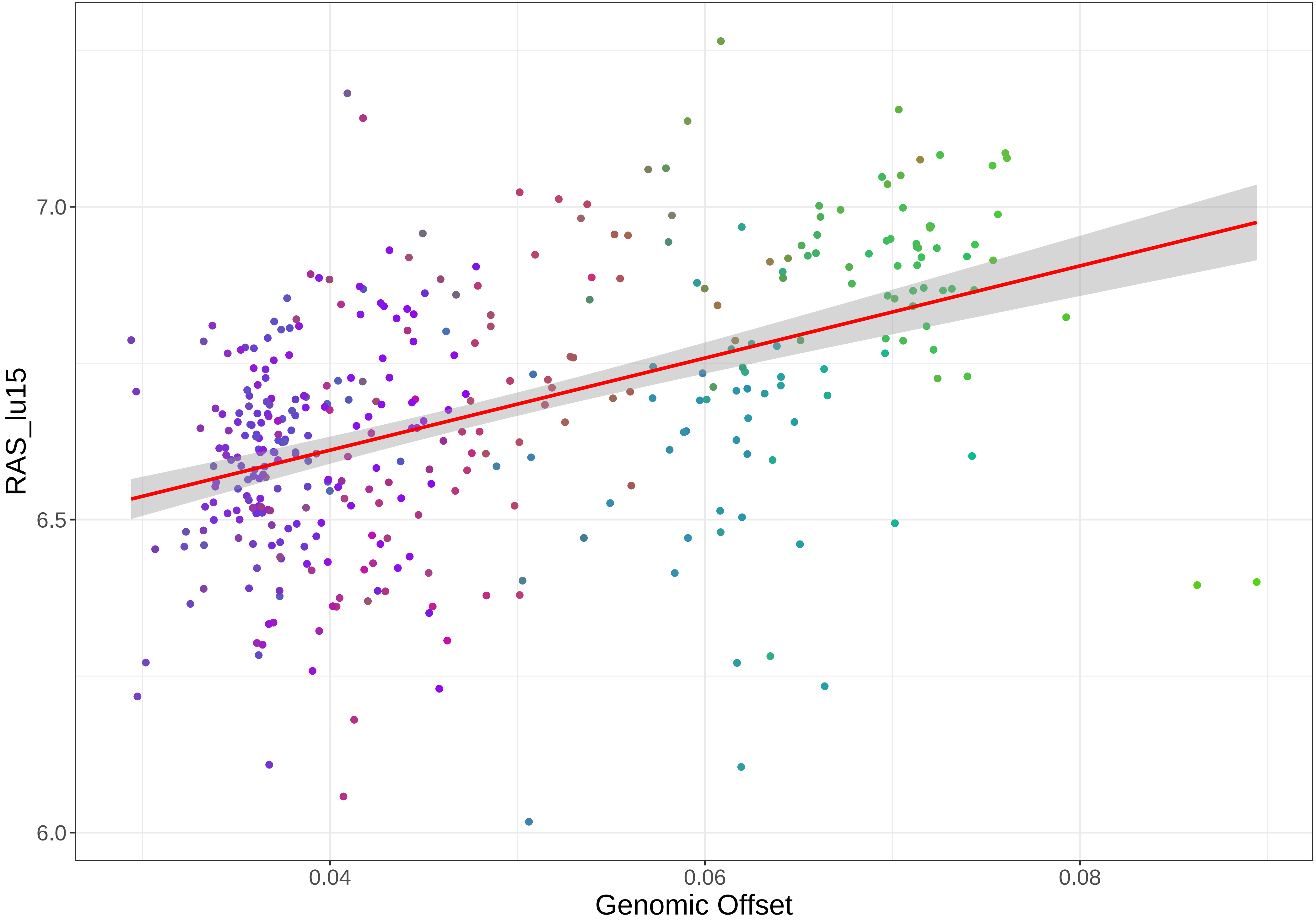

RAS_lu15
7.0
6.5
6.0
0.04
0.06
0.08
Genomic Offset

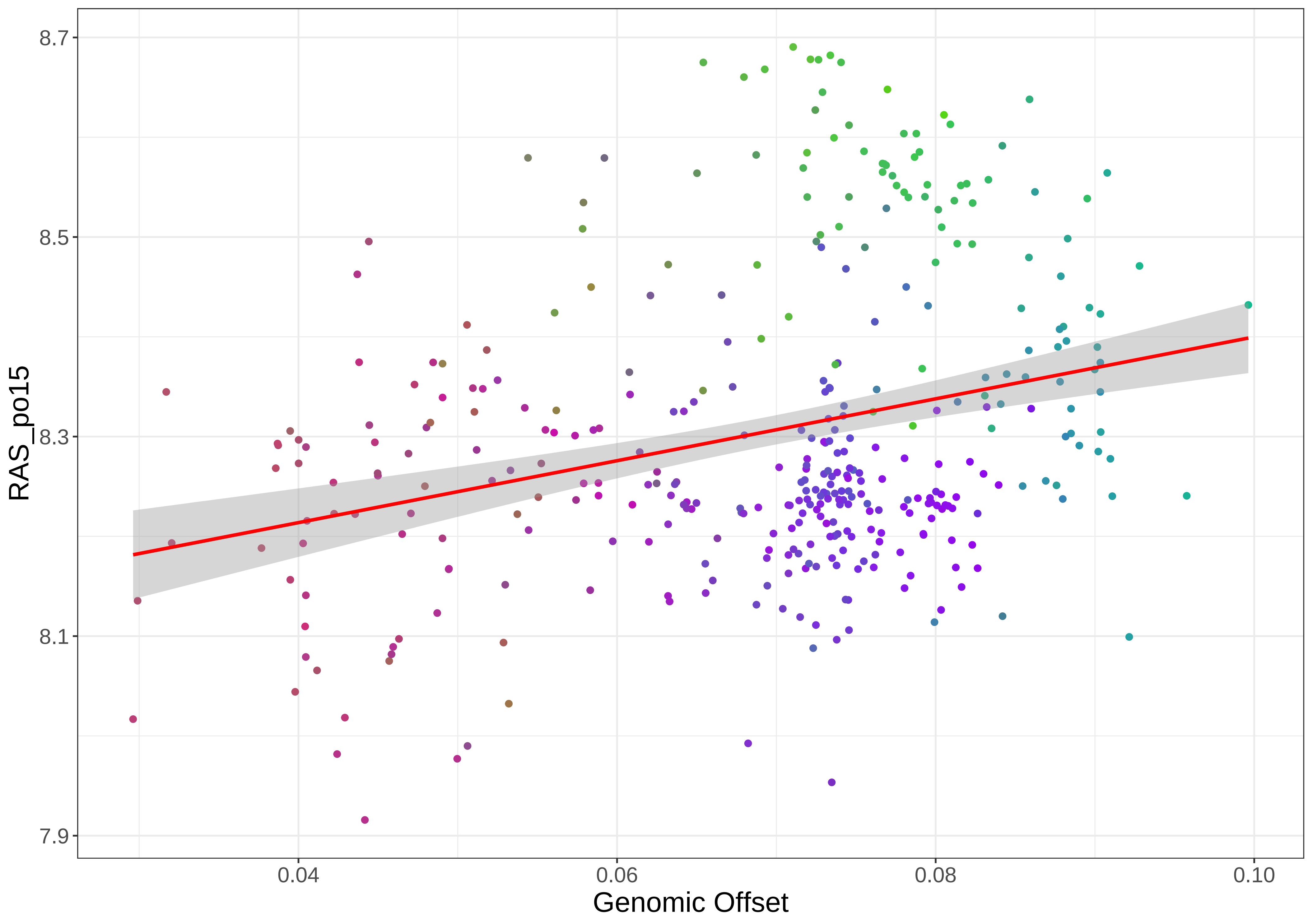

8.7
8.5
8.3
8.1
7.9
RAS_po15
0.04
0.06
0.08
0.10
Genomic Offset

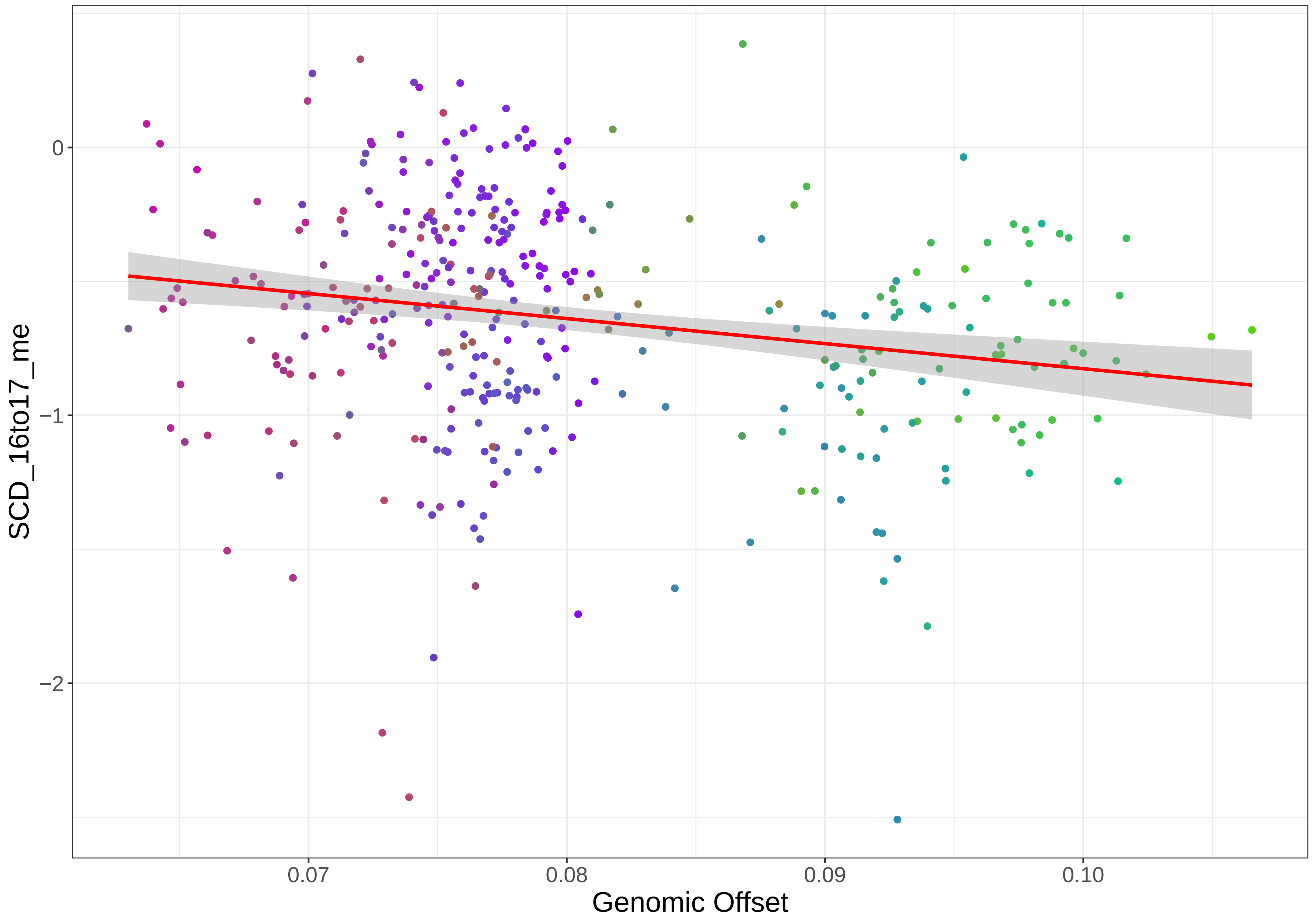
0
−1
−2
0.07
0.08
0.09
0.10
Genomic Offset
SCD_16to17_me

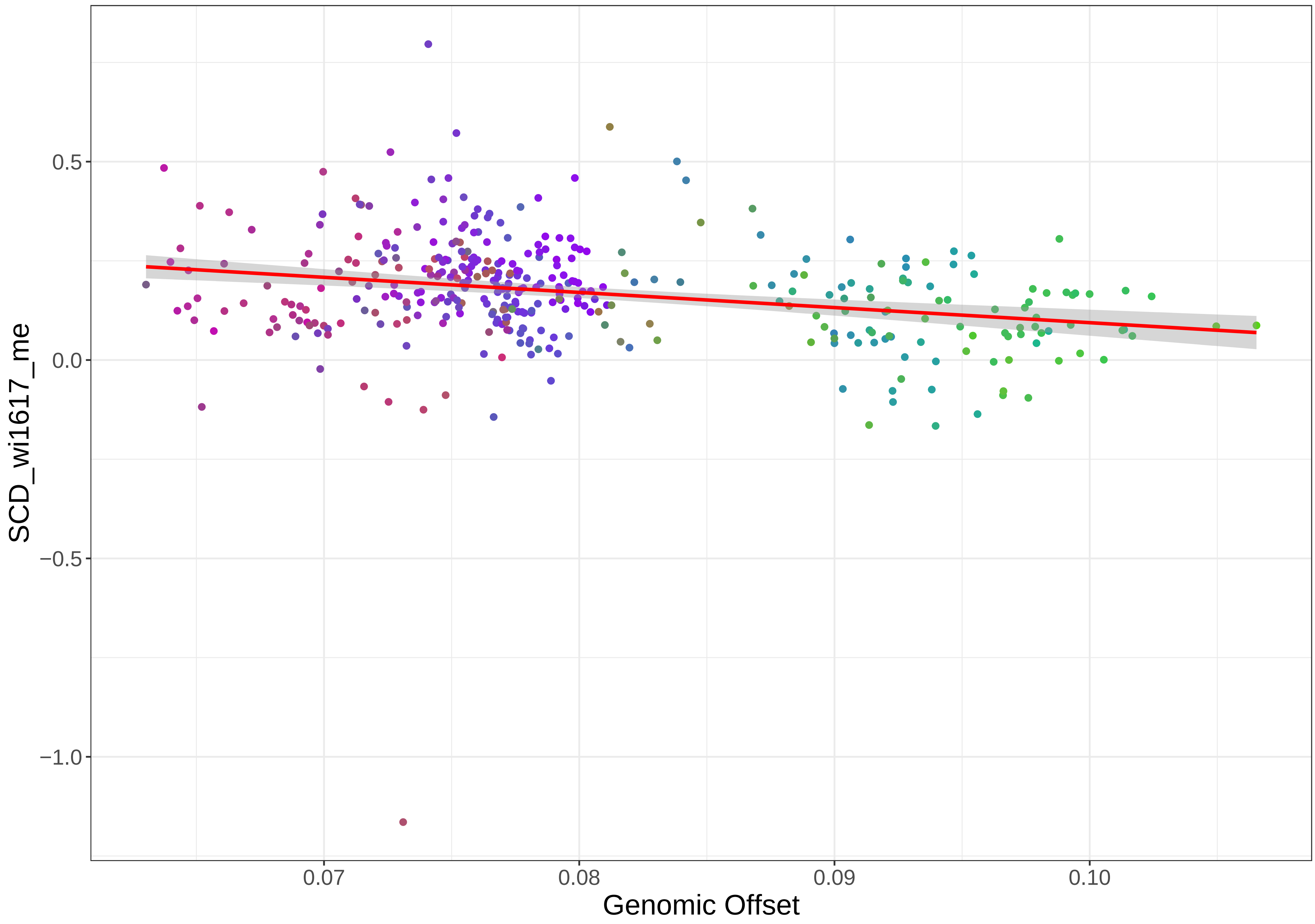

SCD_wi1617_me
0.5
0.0
−0.5
−1.0
0.07
0.08
0.09
0.10
Genomic Offset

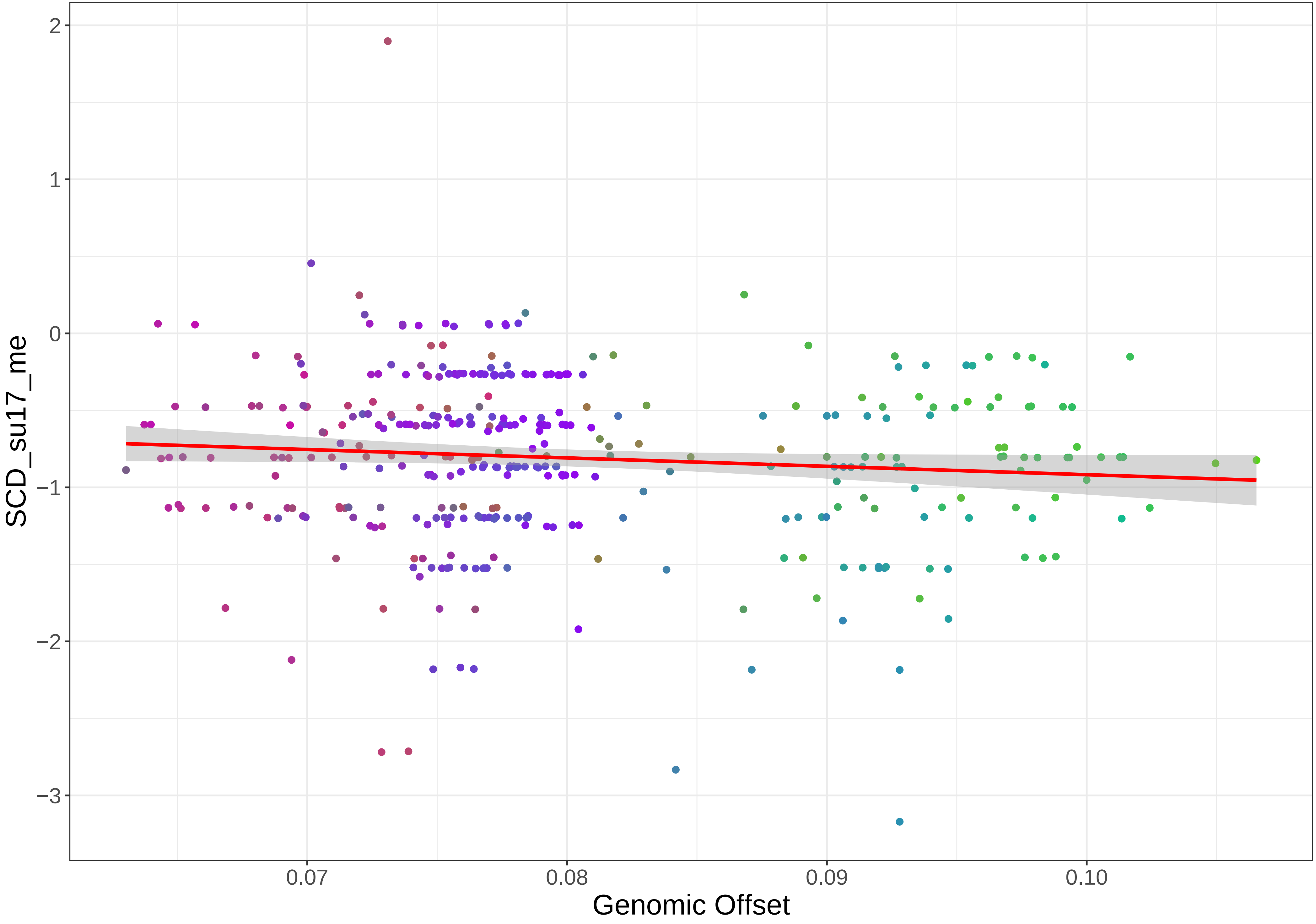

2
1
0
−1
−2
−3
SCD_su17_me
0.07
0.08
0.09
0.10
Genomic Offset

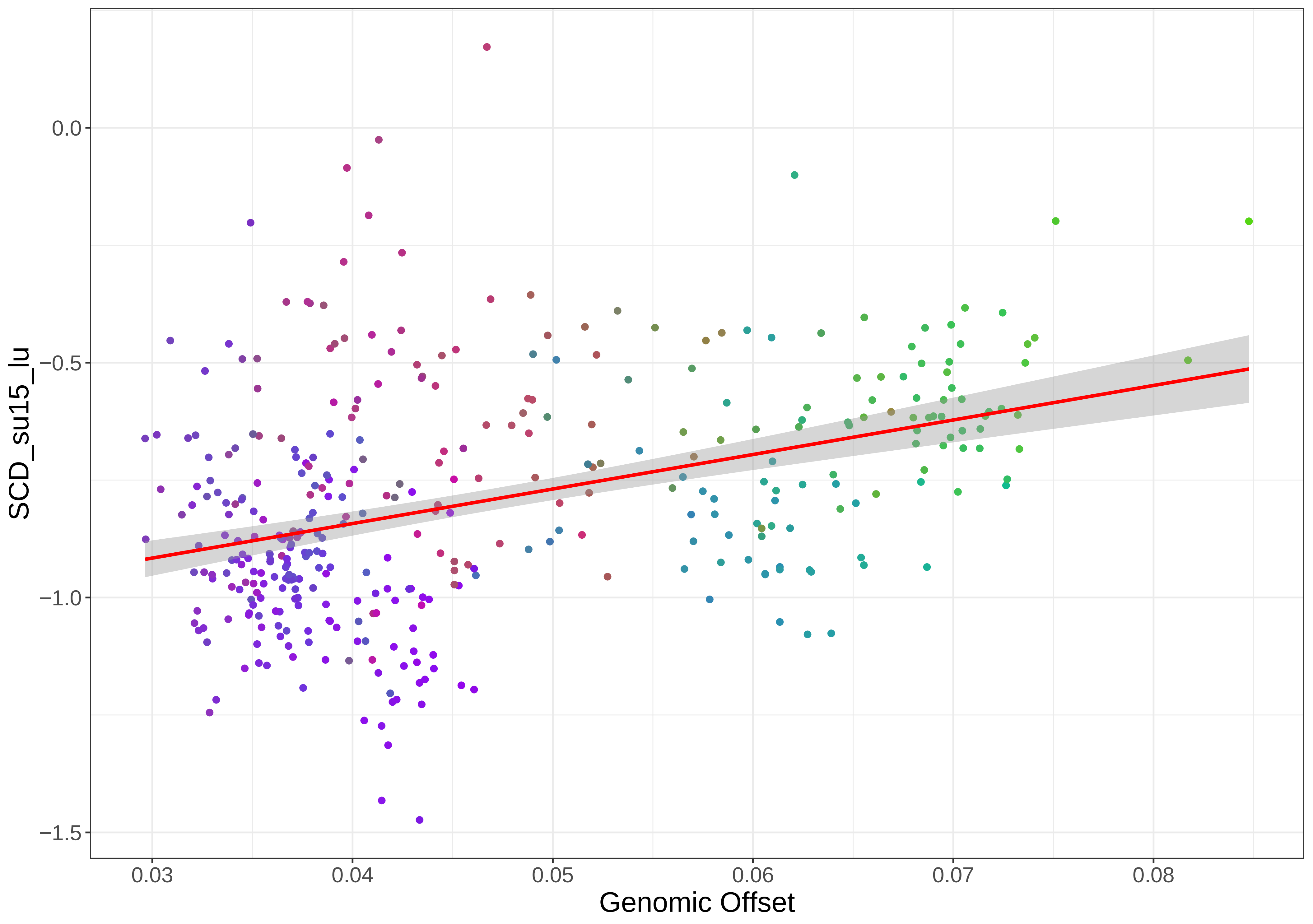

0.0
−0.5
−1.0
−1.5
SCD_su15_lu
0.03
0.04
0.05
0.06
0.07
0.08
Genomic Offset

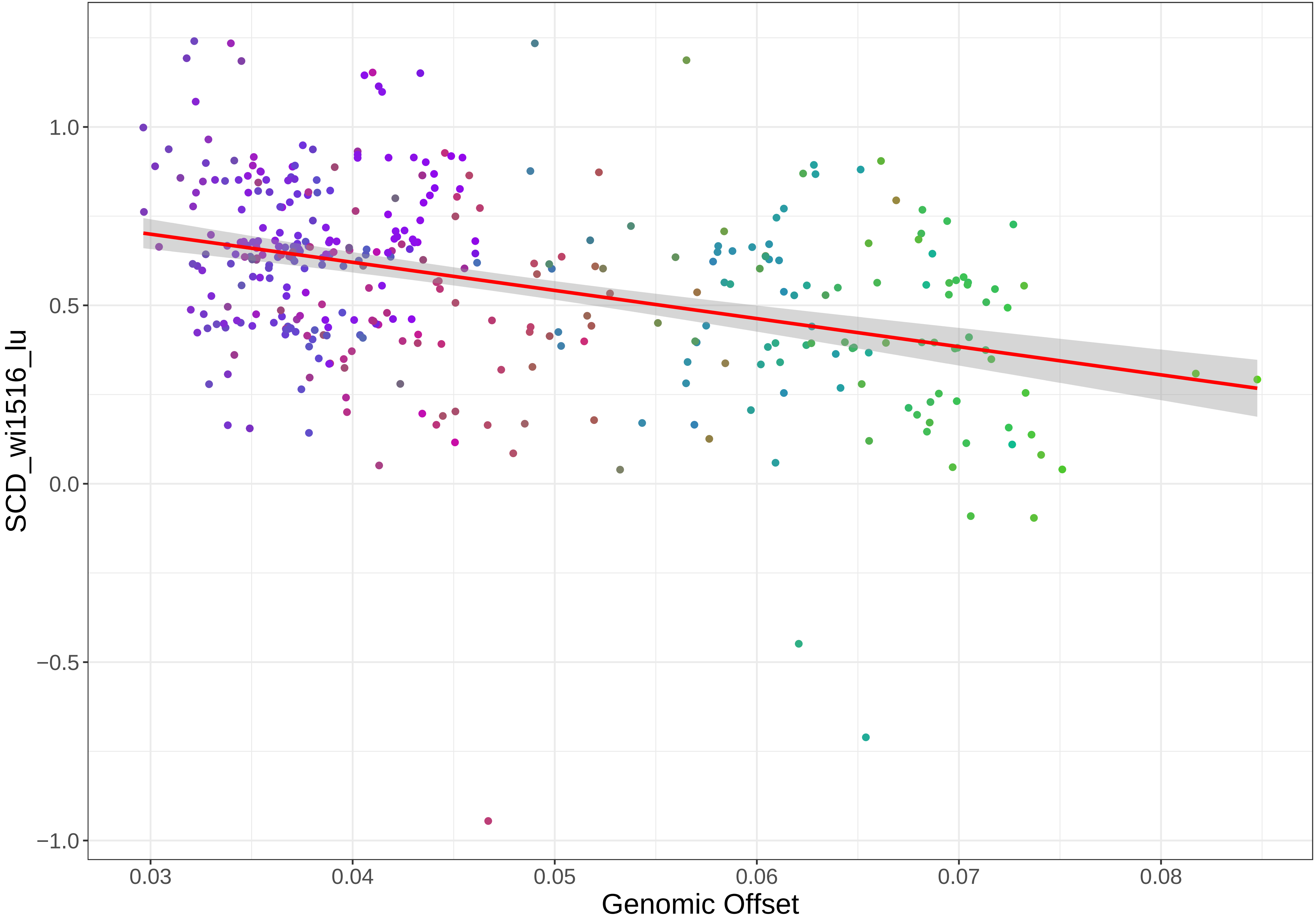
SCD_wi1516_lu
1.0
0.5
0.0
−0.5
−1.0
0.03
0.04
0.05
0.06
0.07
0.08
Genomic Offset

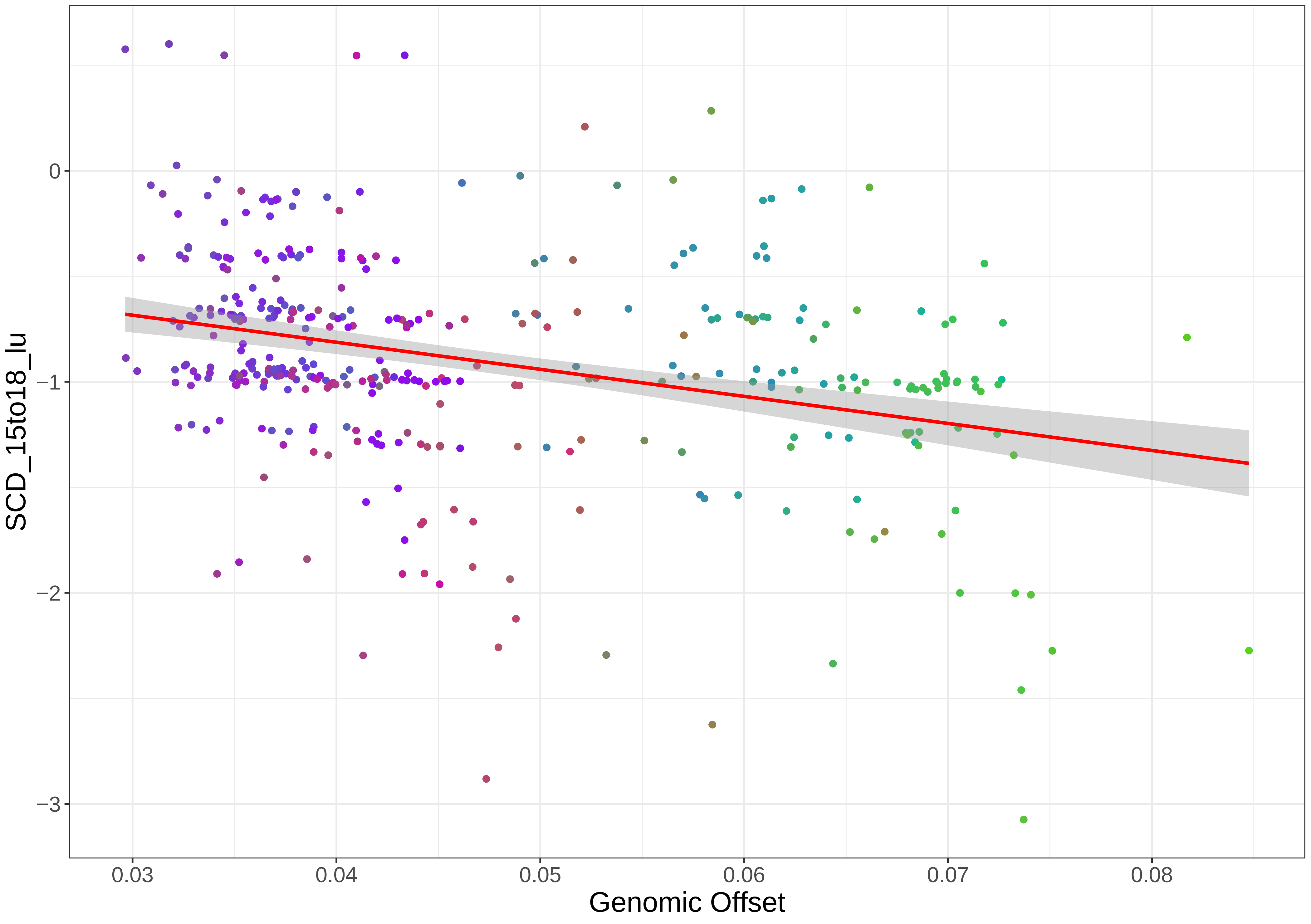

SCD_15to18_lu
0
−1
−2
−3
0.03
0.04
0.05
0.06
0.07
0.08
Genomic Offset

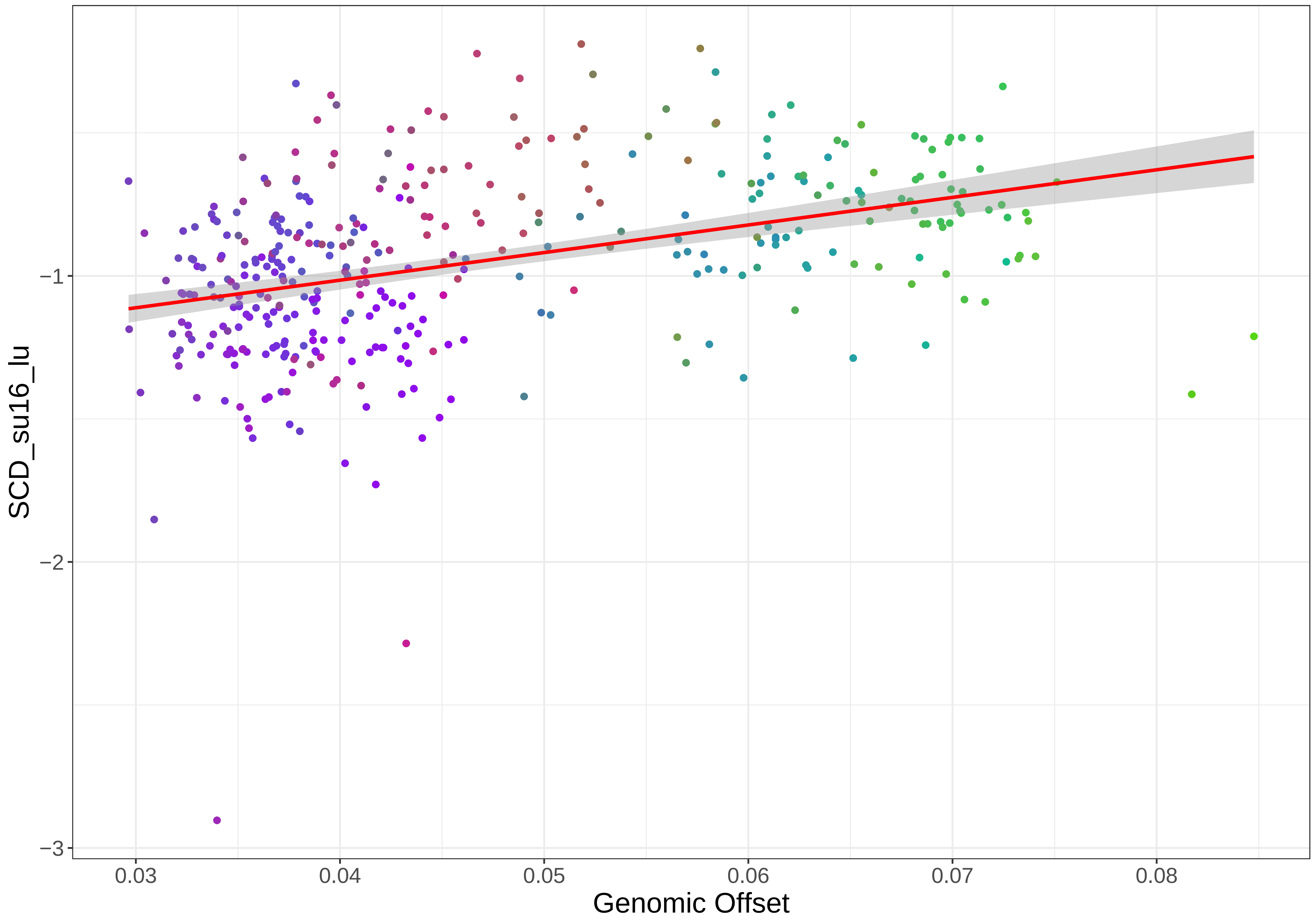

SCD_su16_lu
−1
−2
−3
0.03
0.04
0.05
0.06
0.07
0.08
Genomic Offset

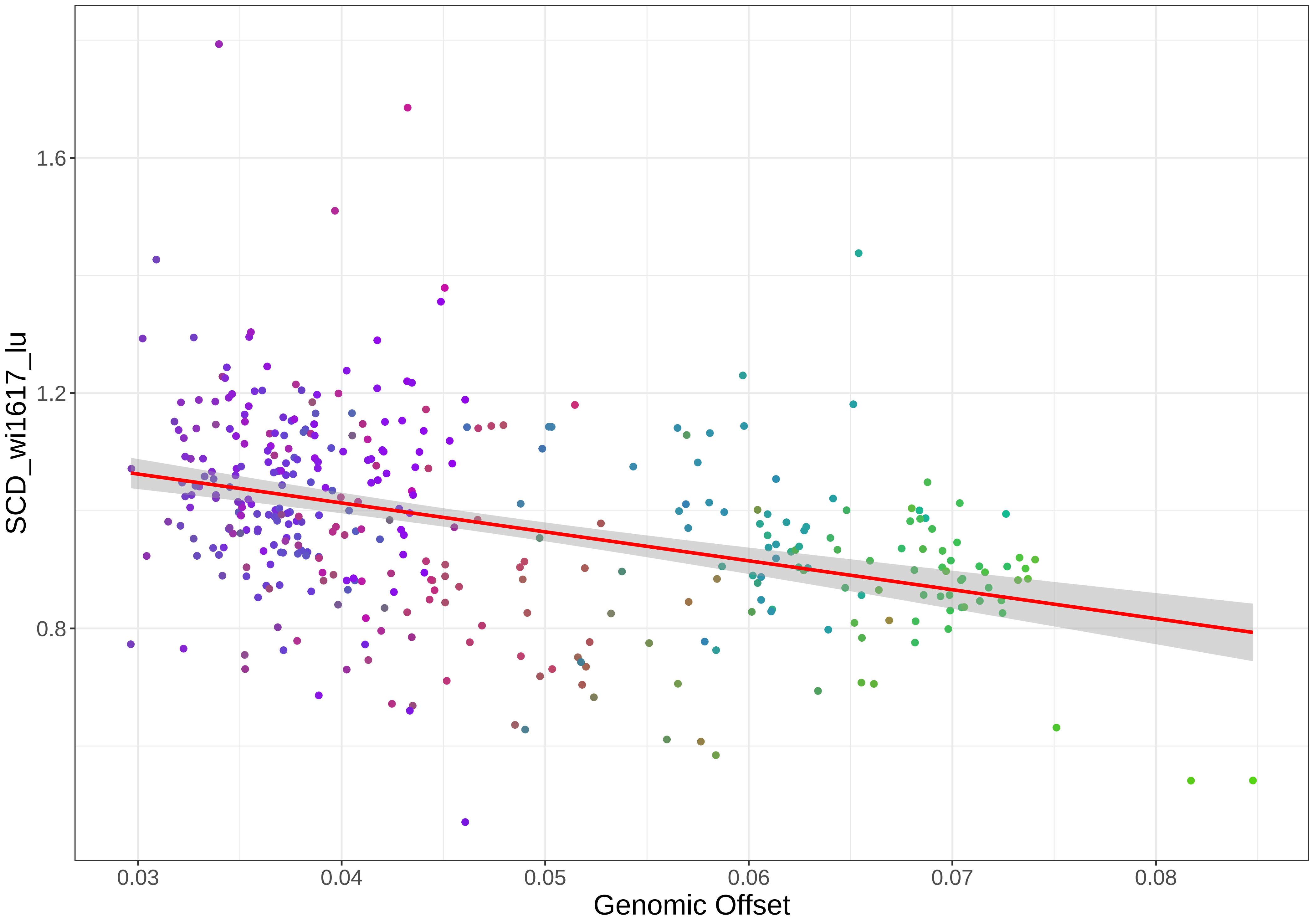

1.6
1.2
0.8
SCD_wi1617_lu
0.03
0.04
0.05
0.06
0.07
0.08
Genomic Offset

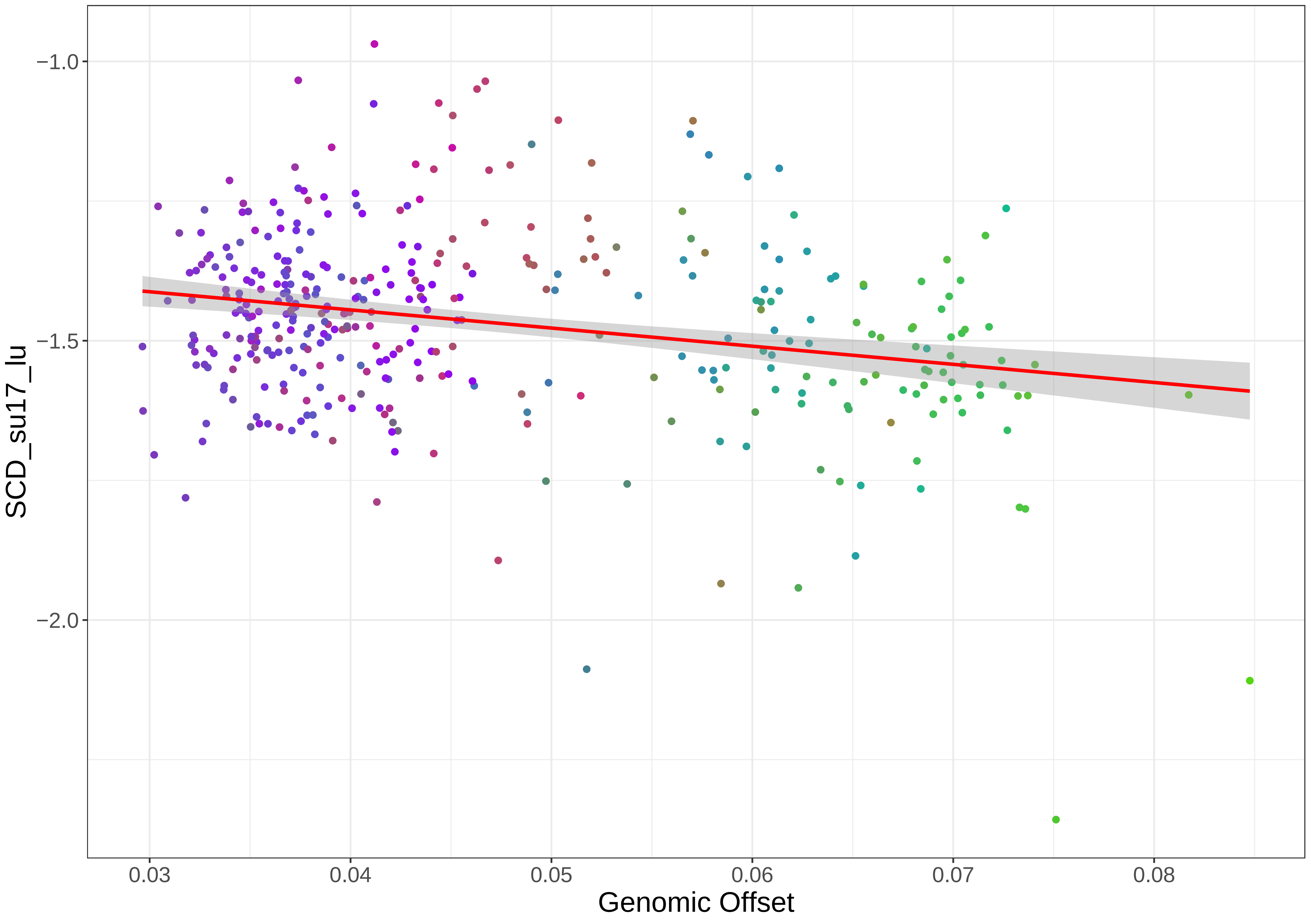

SCD_su17_lu
−1.0
−1.5
−2.0
0.03
0.04
0.05
0.06
0.07
0.08
Genomic Offset

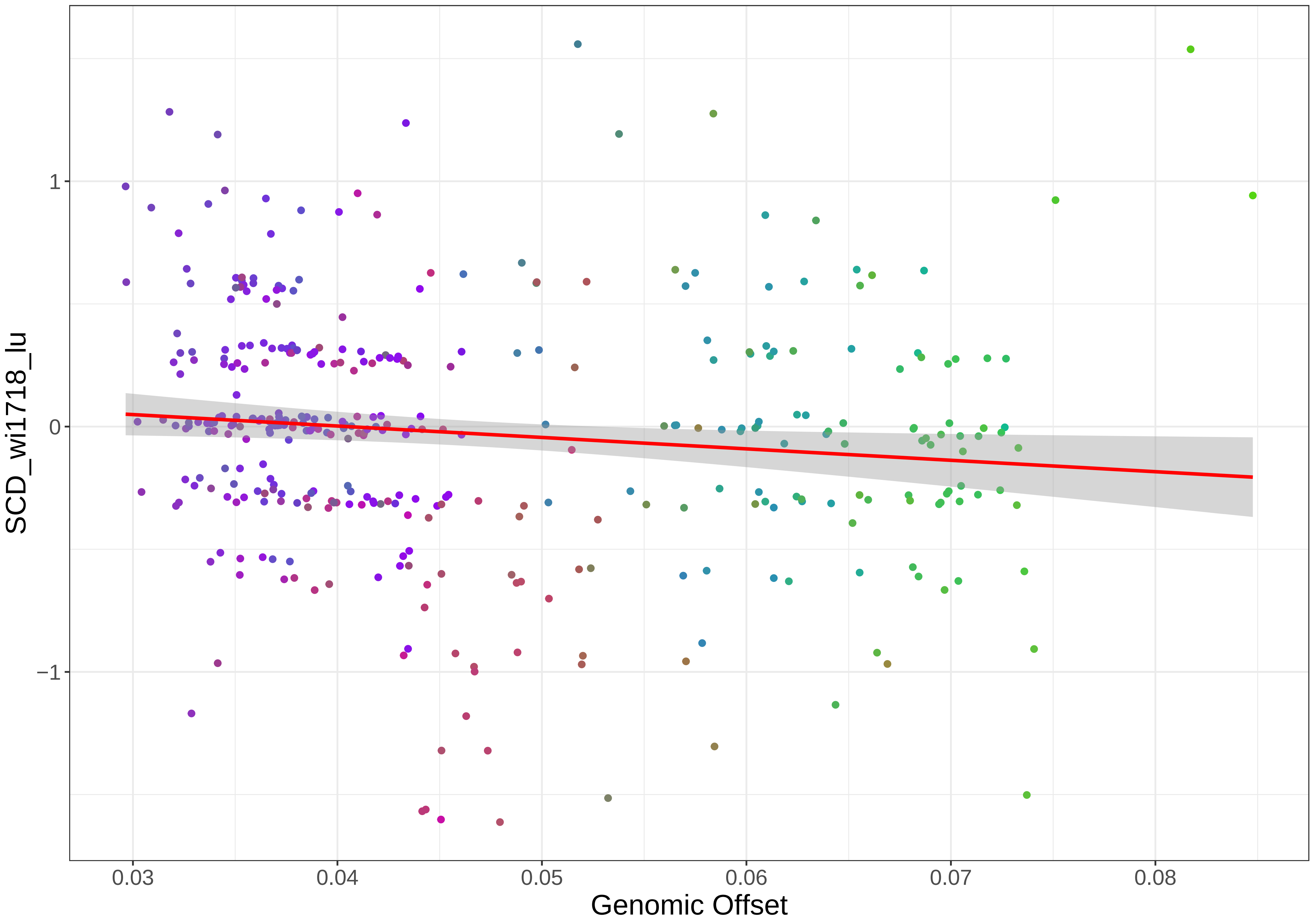

SCD_wi1718_lu
1
0
−1
0.03
0.04
0.05
0.06
0.07
0.08
Genomic Offset

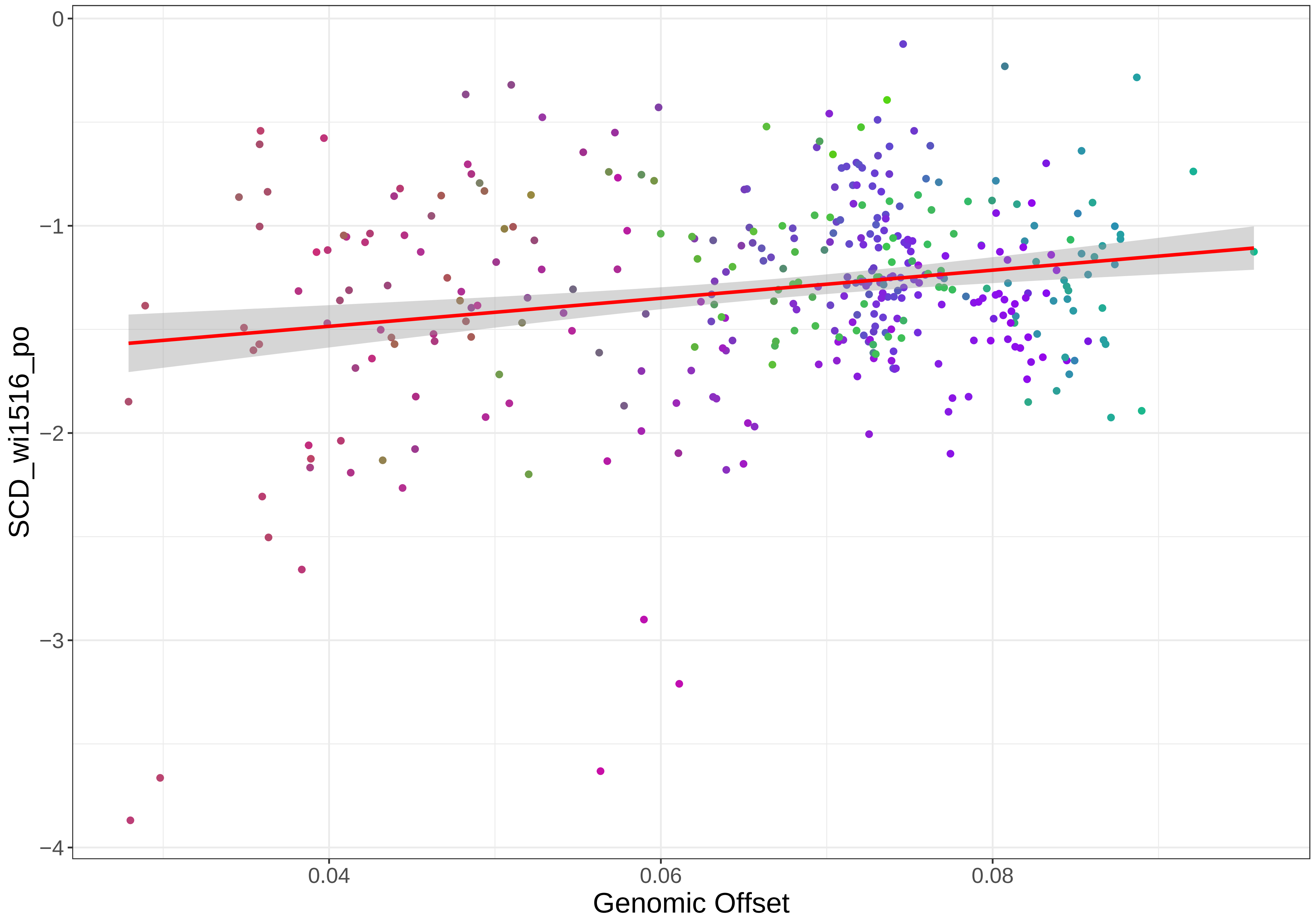

0
−1
−2
−3
−4
SCD_wi1516_po
0.04
0.06
0.08
Genomic Offset

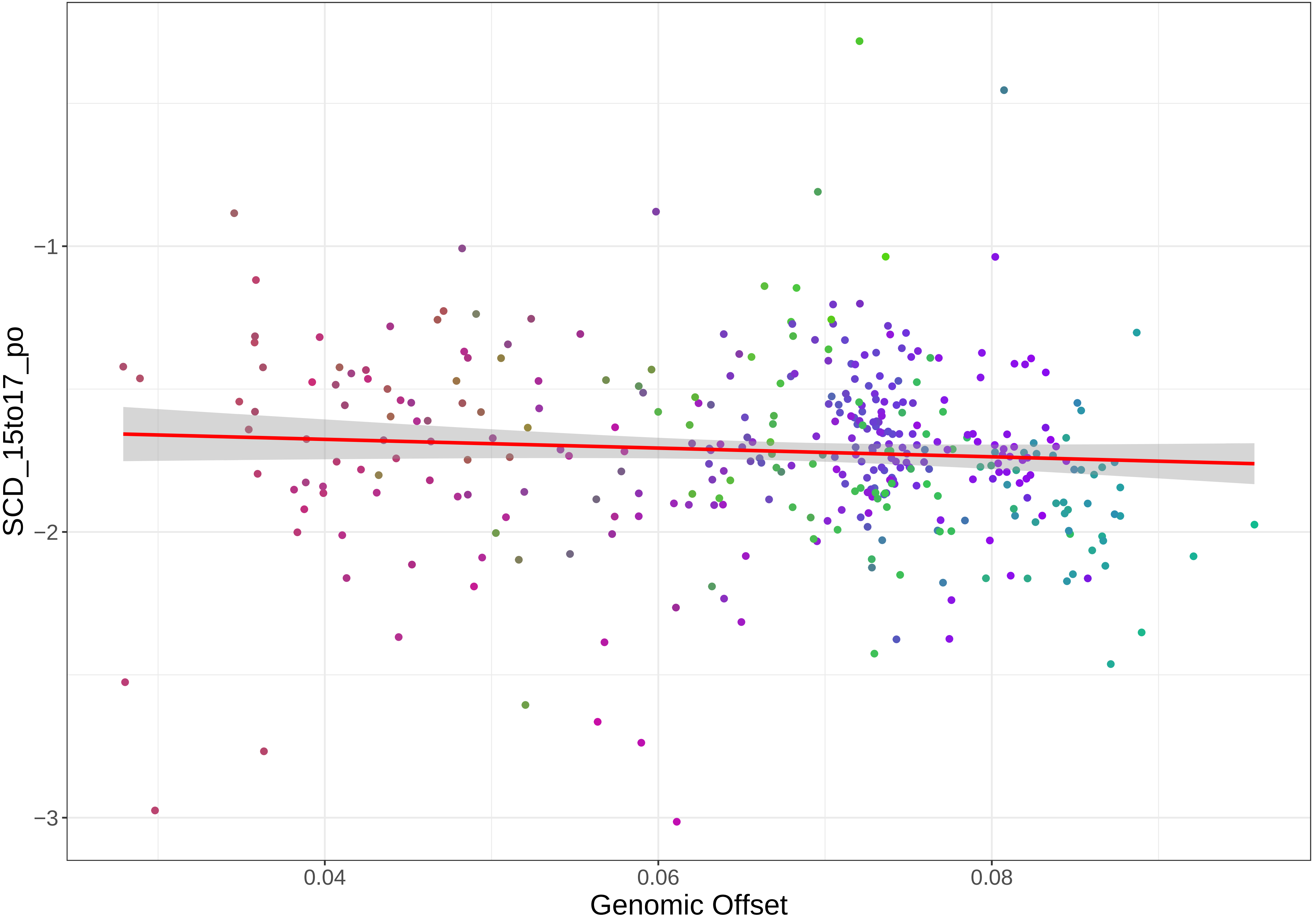

SCD_15to17_po
−1
−2
−3
0.04
0.06
0.08
Genomic Offset

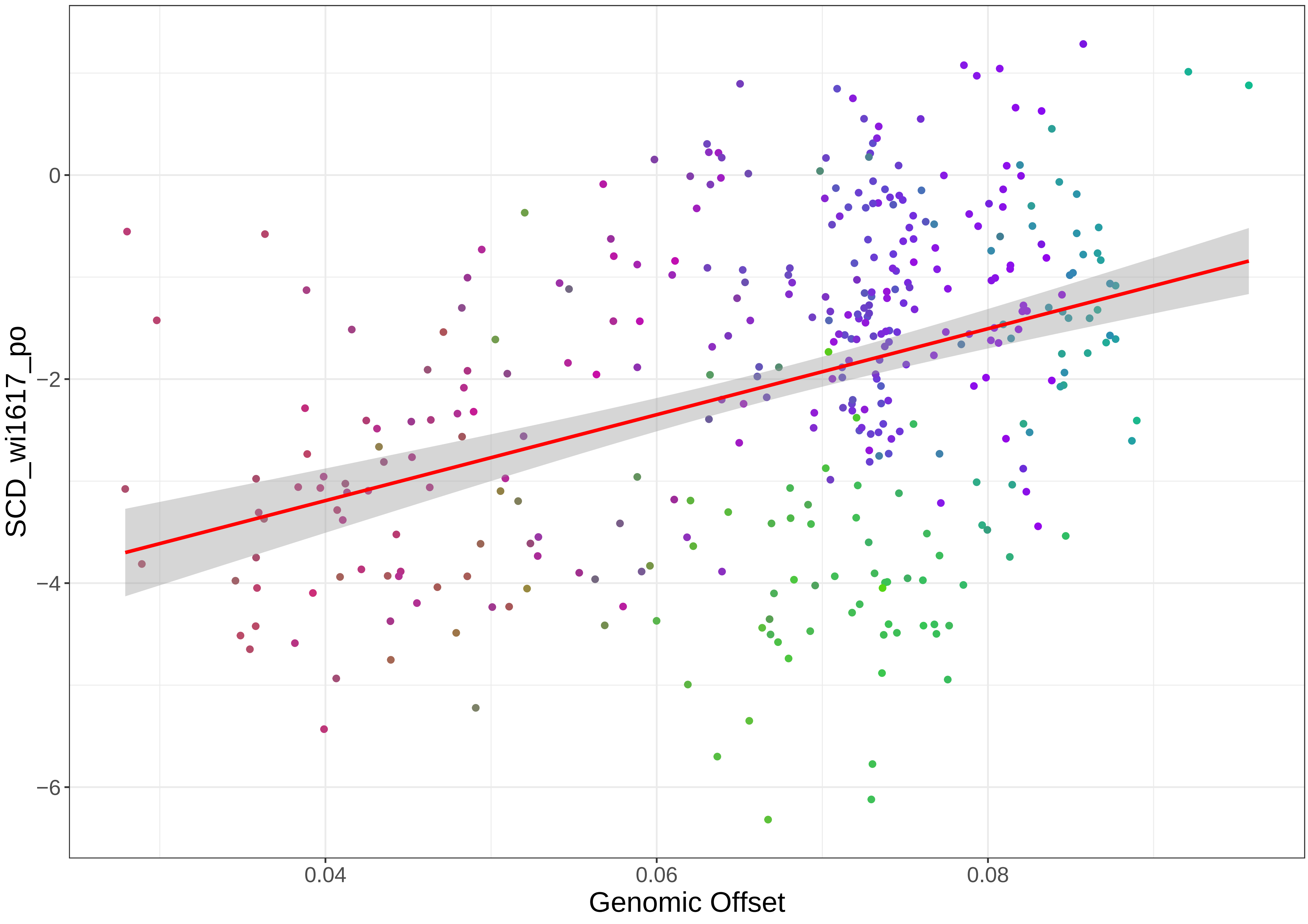
SCD_wi1617_po
0
−2
−4
−6
0.04
0.06
0.08
Genomic Offset

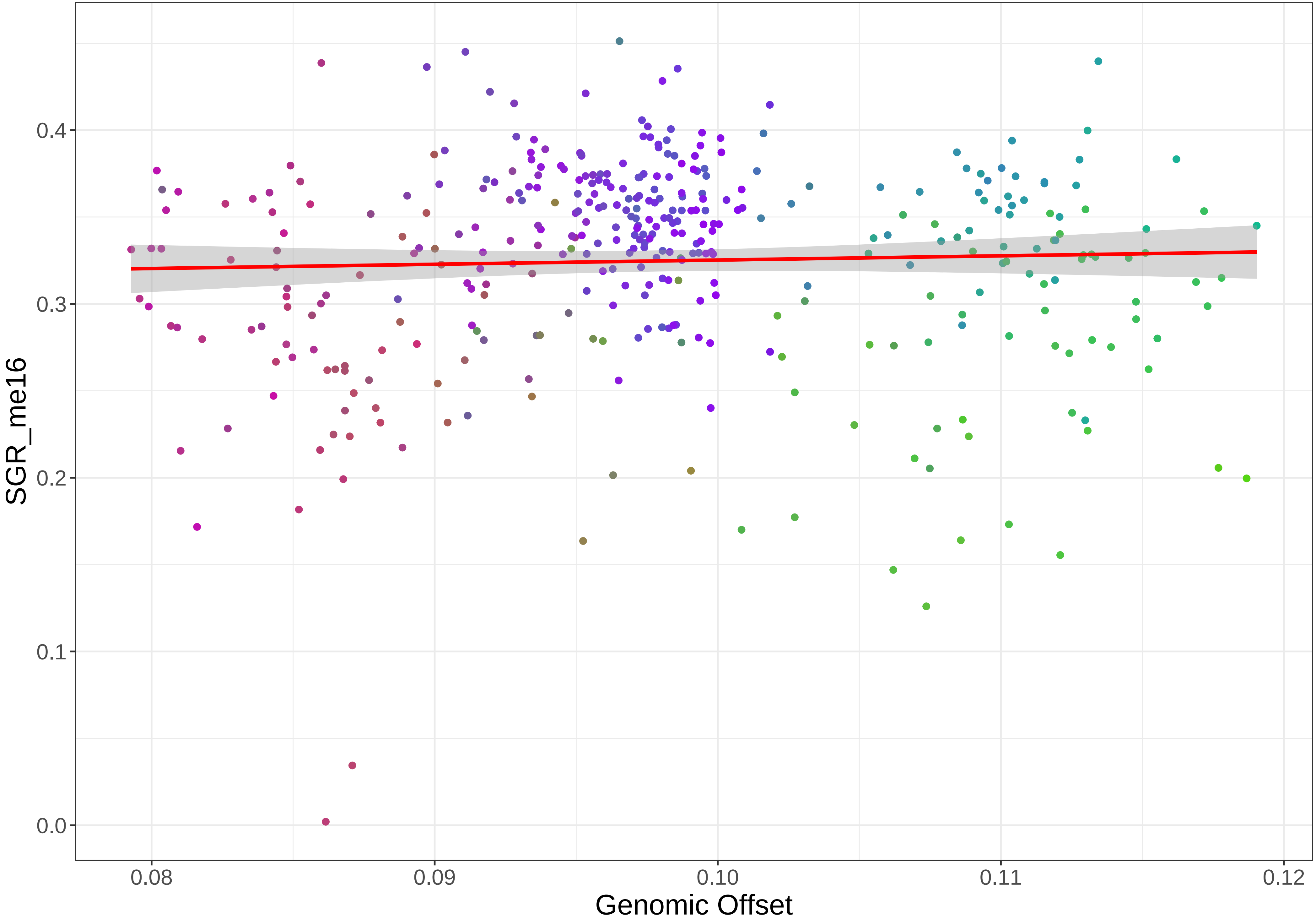

SGR_me16
0.0
0.1
0.2
0.3
0.4
0.08
0.09
0.10
0.11
0.12
Genomic Offset

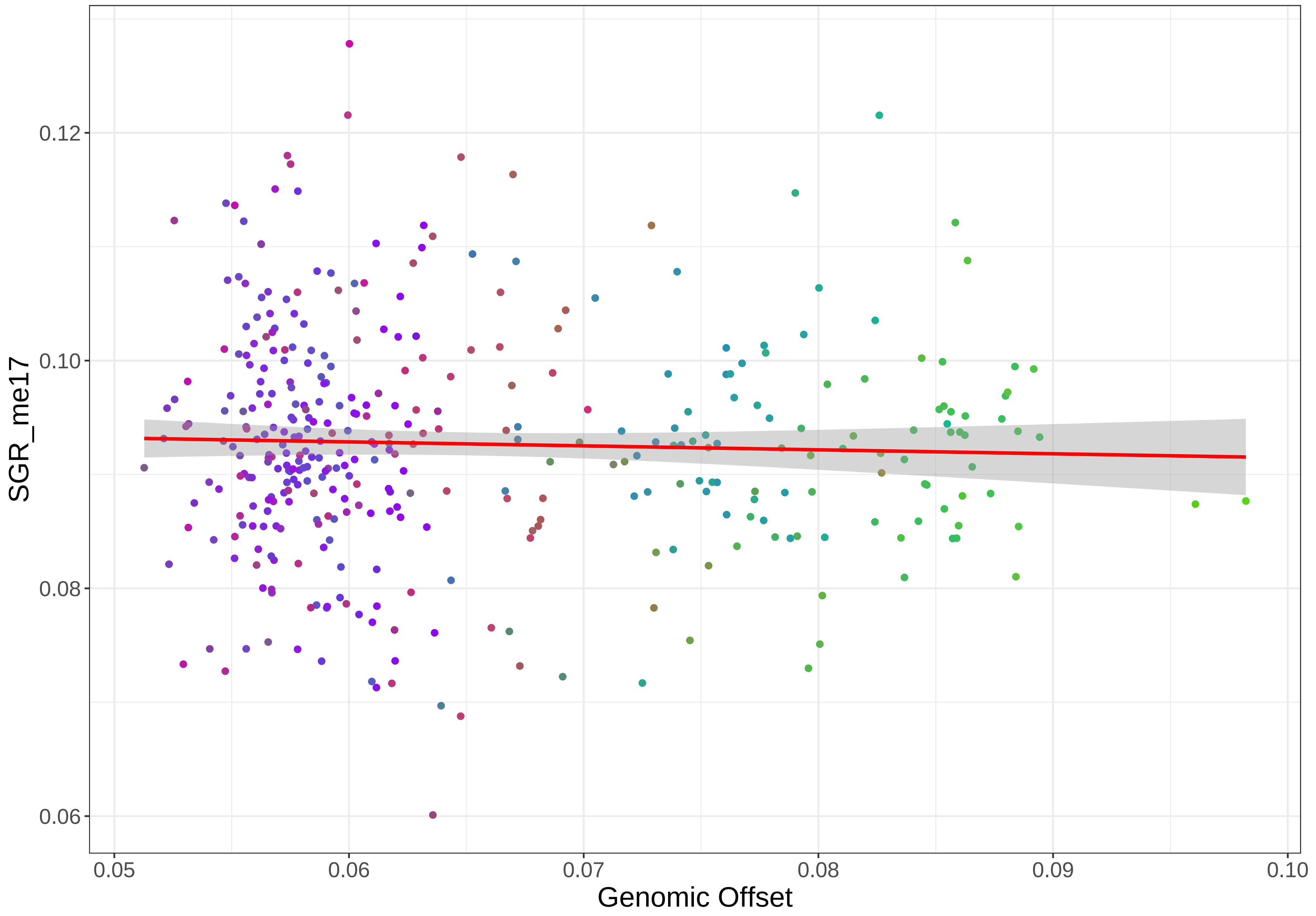

SGR_me17
0.12
0.10
0.08
0.06
0.05
0.06
0.07
0.08
0.09
0.10
Genomic Offset

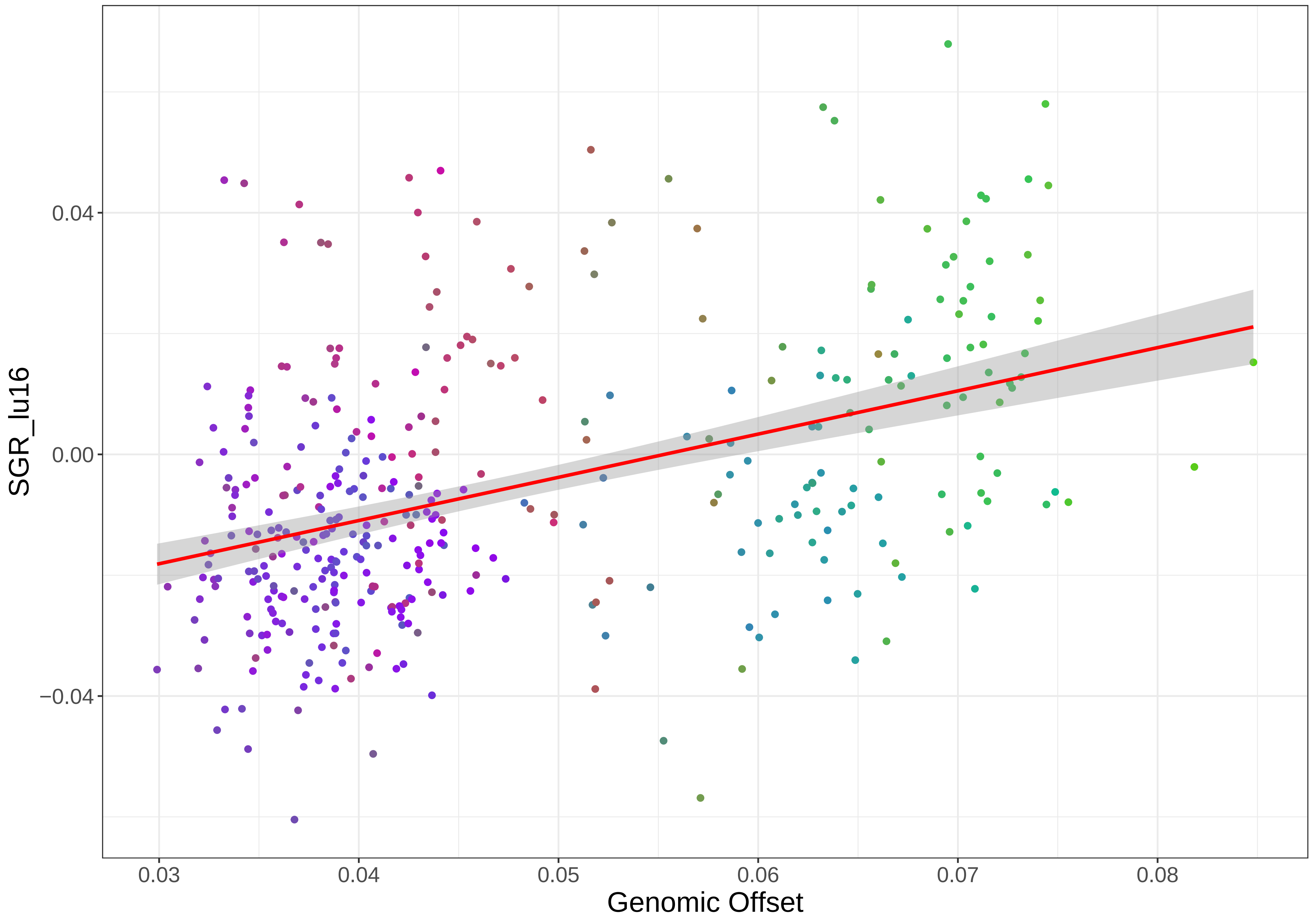

SGR_lu16
0.04
0.00
−0.04
0.03
0.04
0.05
0.06
0.07
0.08
Genomic Offset

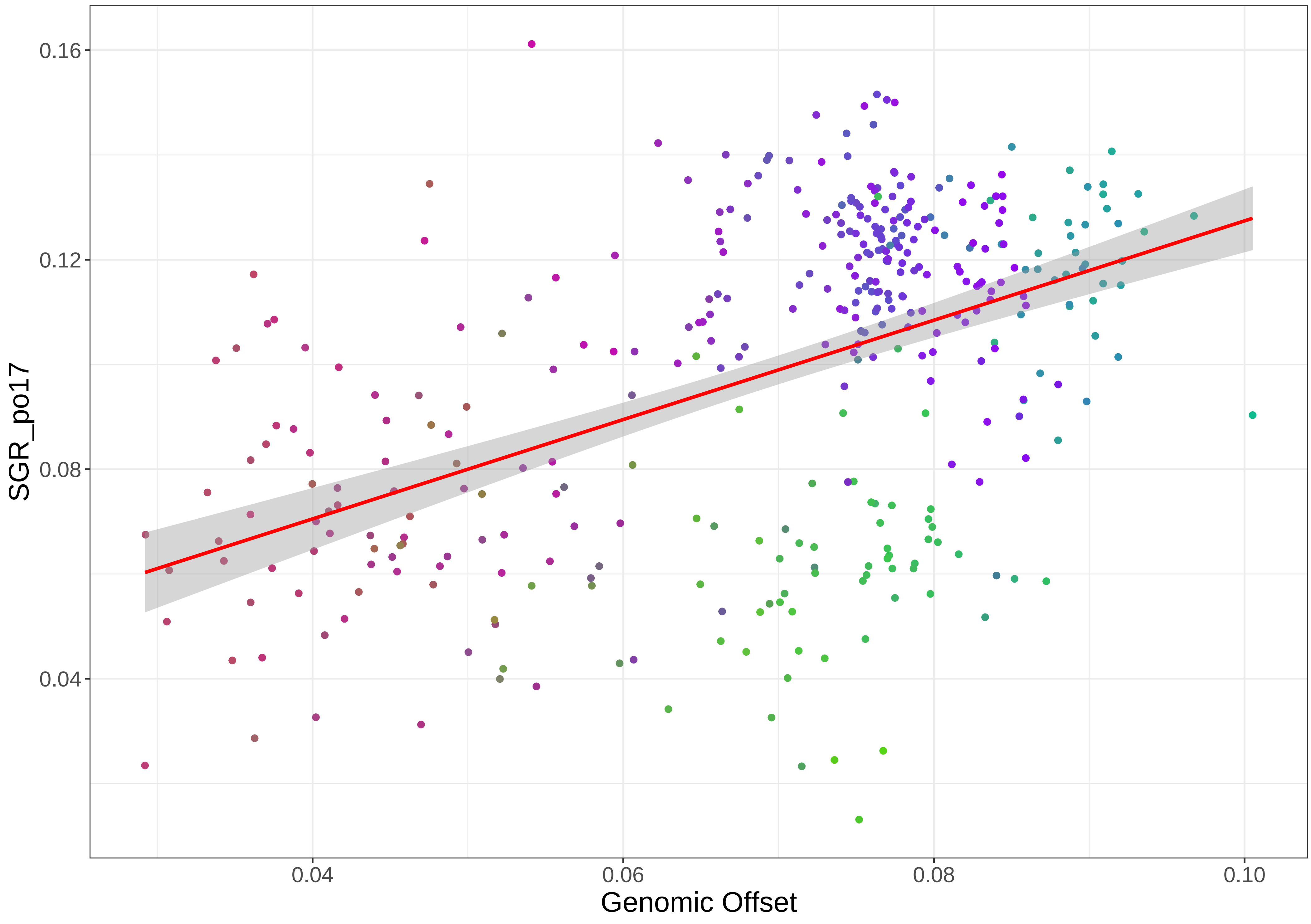

SGR_po17
0.16
0.12
0.08
0.04
0.04
0.06
0.08
0.10
Genomic Offset

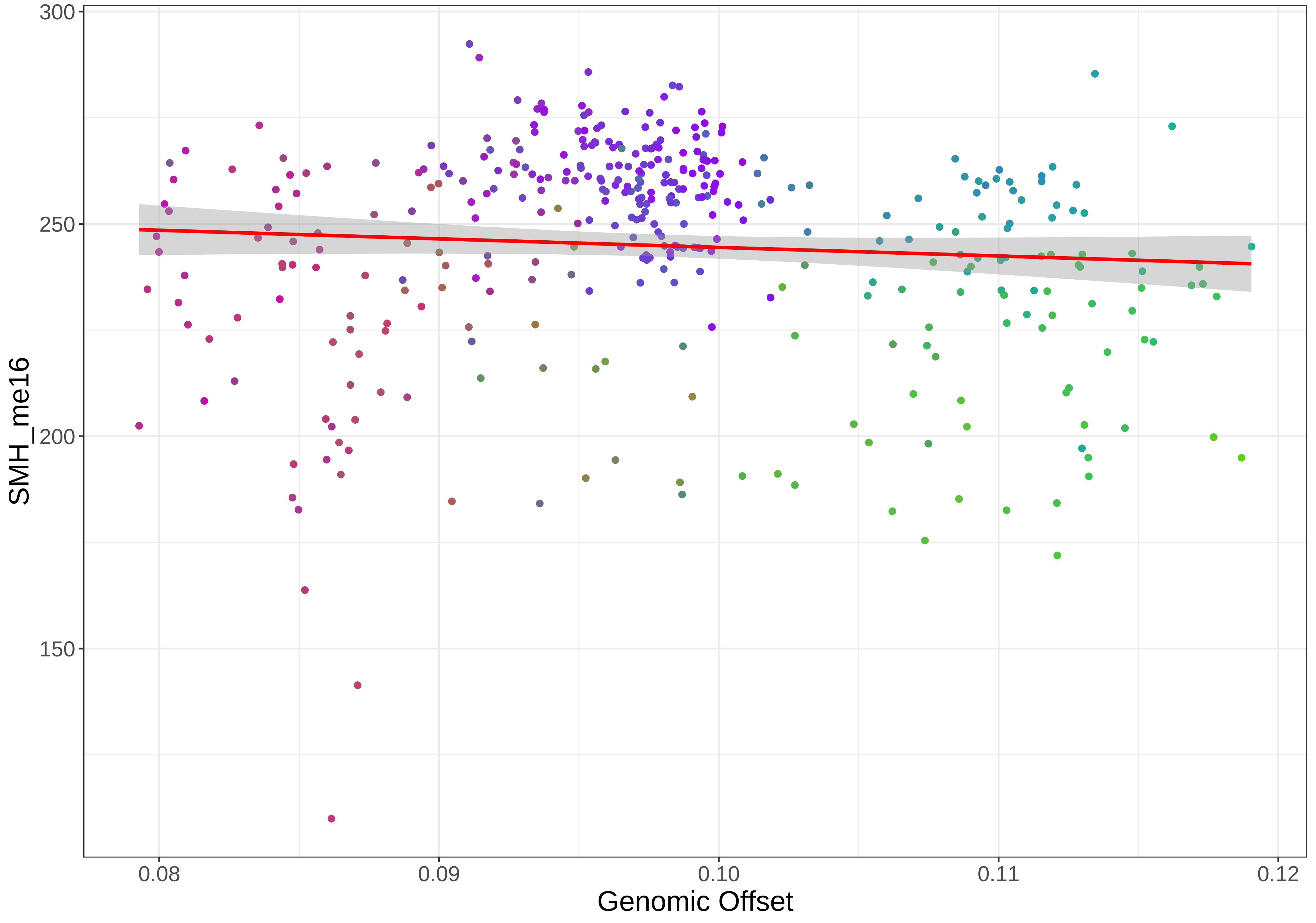

300
250
200
150
SMH_me16
0.08
0.09
0.10
0.11
0.12
Genomic Offset

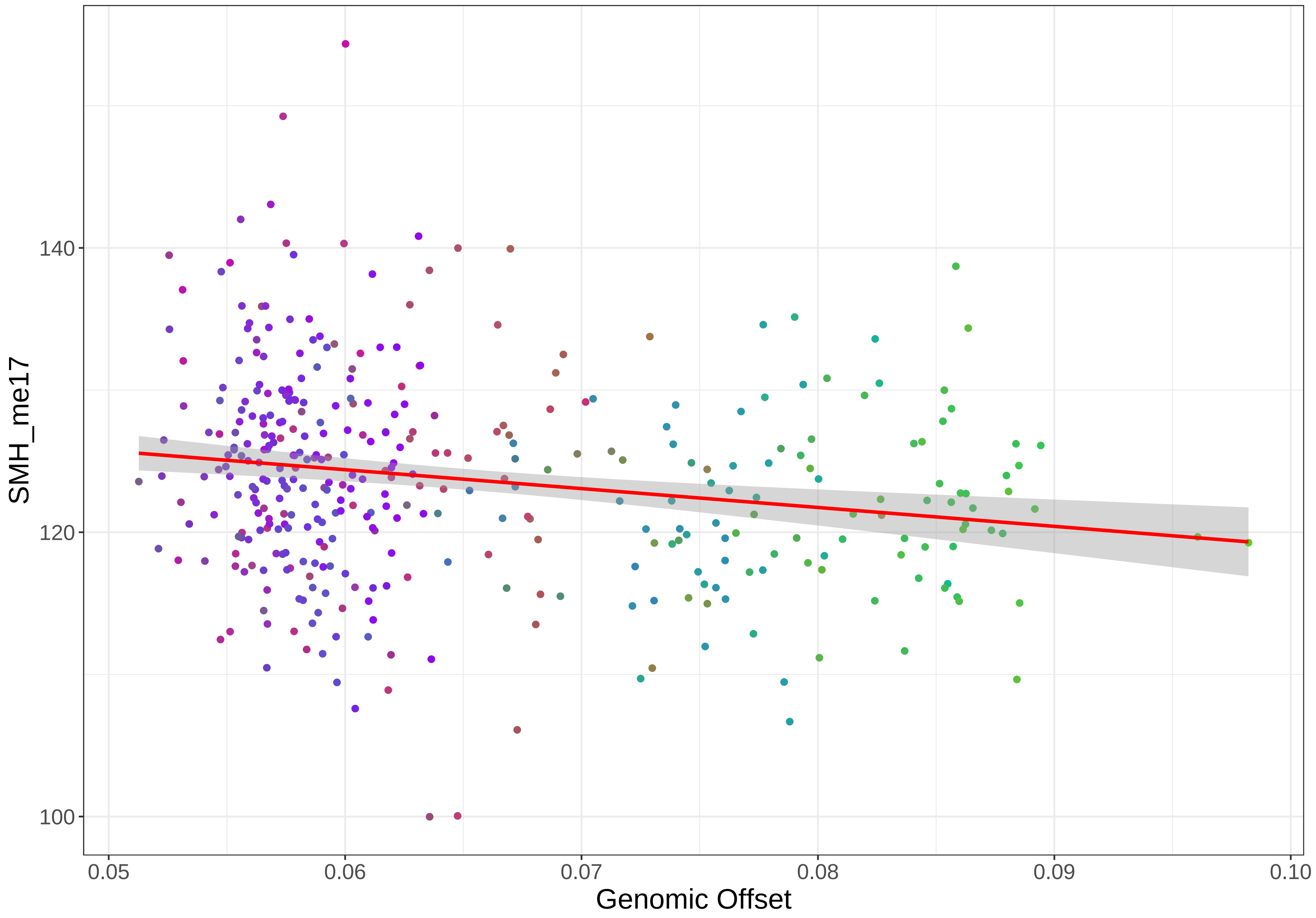

SMH_me17
140
120
100
0.05
0.06
0.07
0.08
0.09
0.10
Genomic Offset

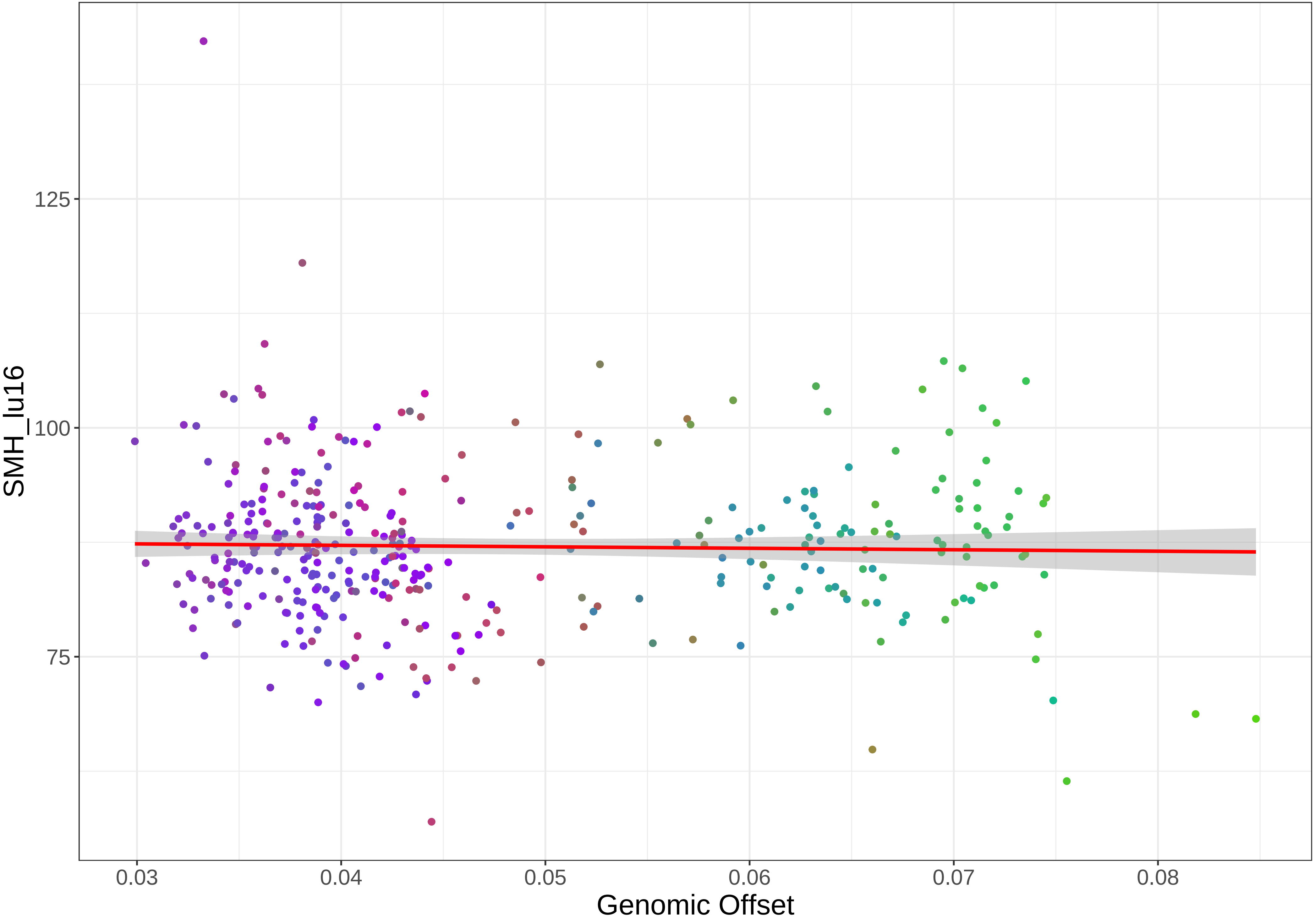

SMH_lu16
125
100
75
0.03
0.04
0.05
0.06
0.07
0.08
Genomic Offset

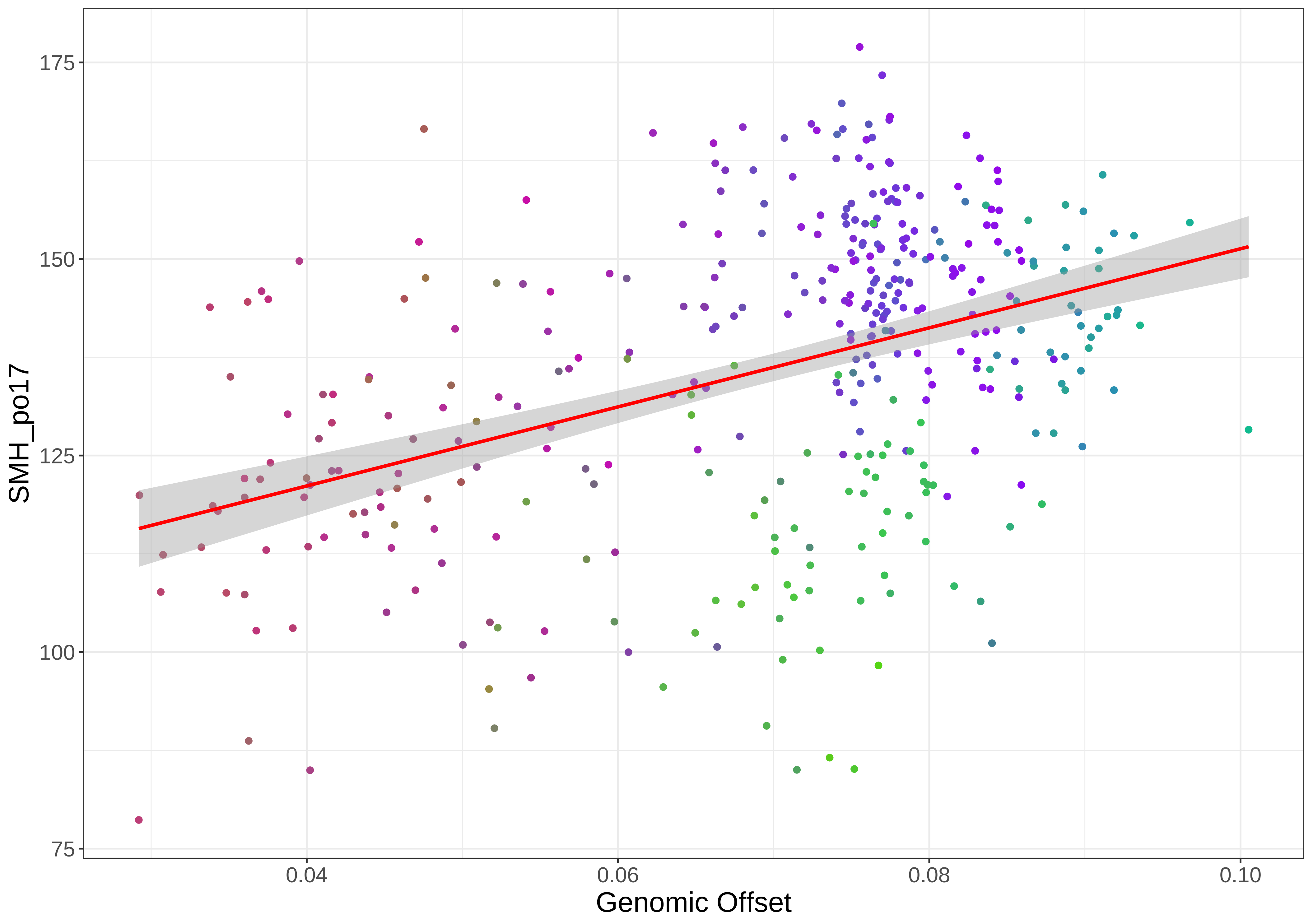

SMH_po17
175
150
125
100
75
0.04
0.06
0.08
0.10
Genomic Offset

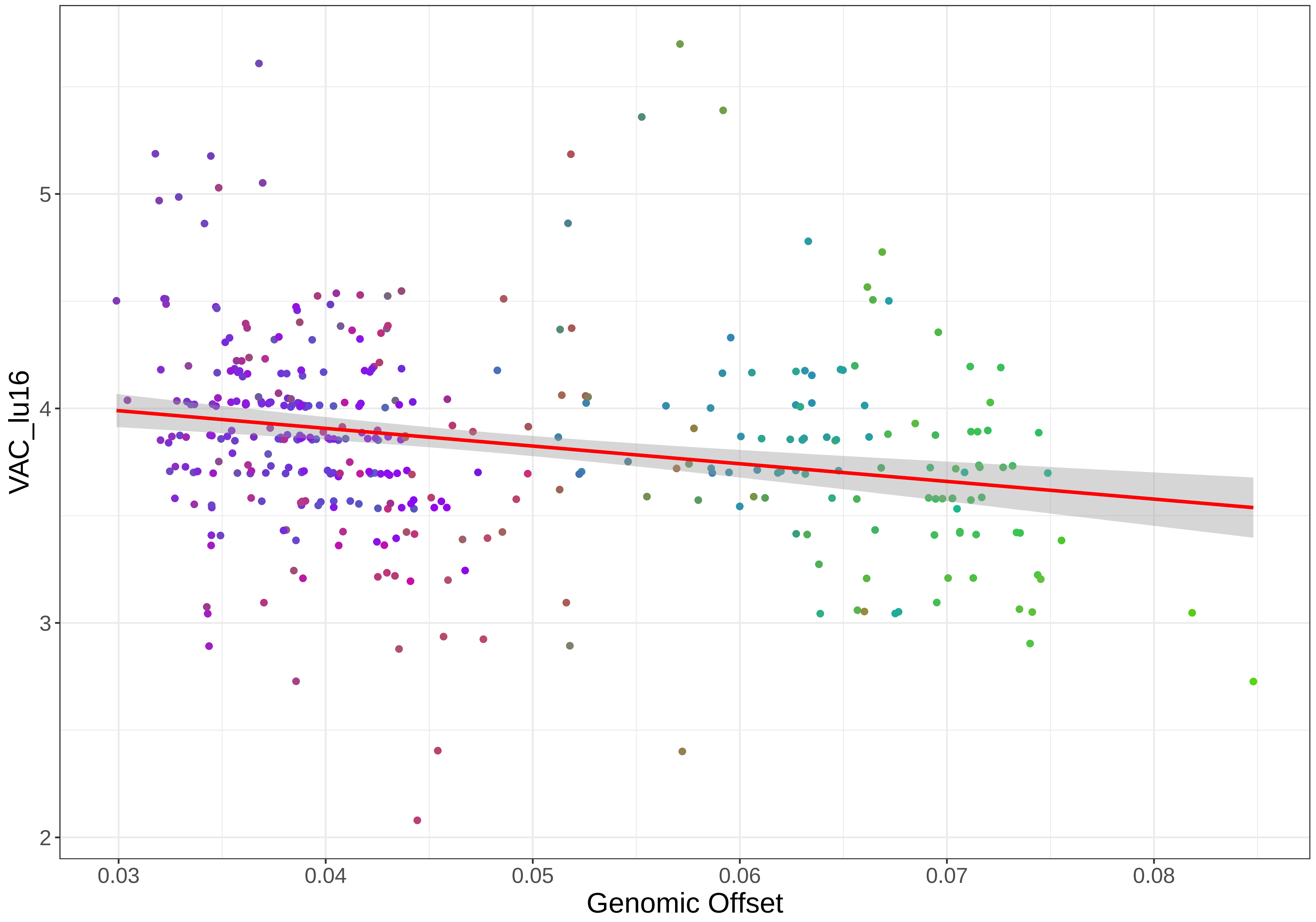

VAC_lu16
5
4
3
2
0.03
0.04
0.05
0.06
0.07
0.08
Genomic Offset

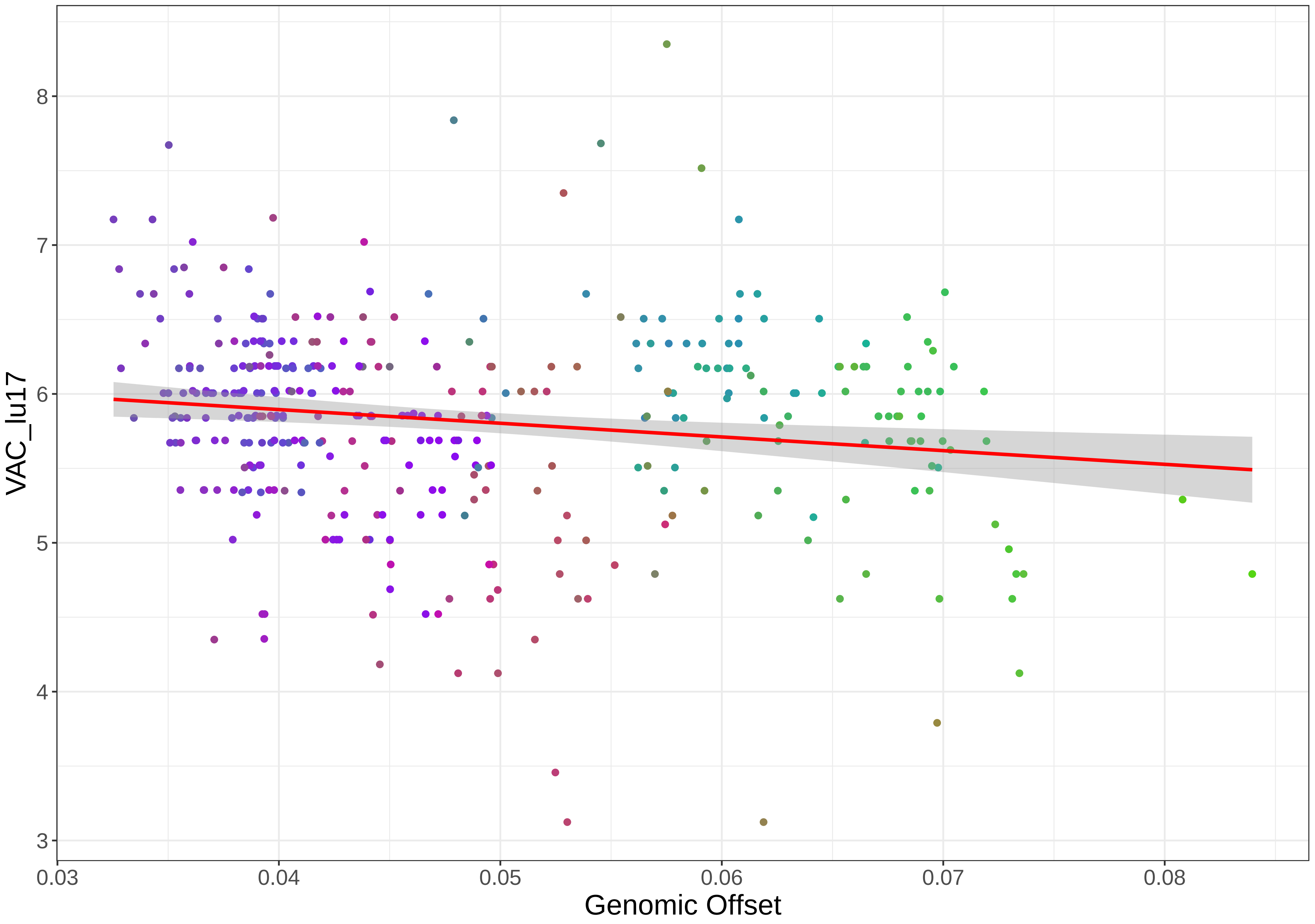

8
7
6
5
4
3
0.03
0.04
0.05
0.06
0.07
0.08
Genomic Offset
VAC_lu17

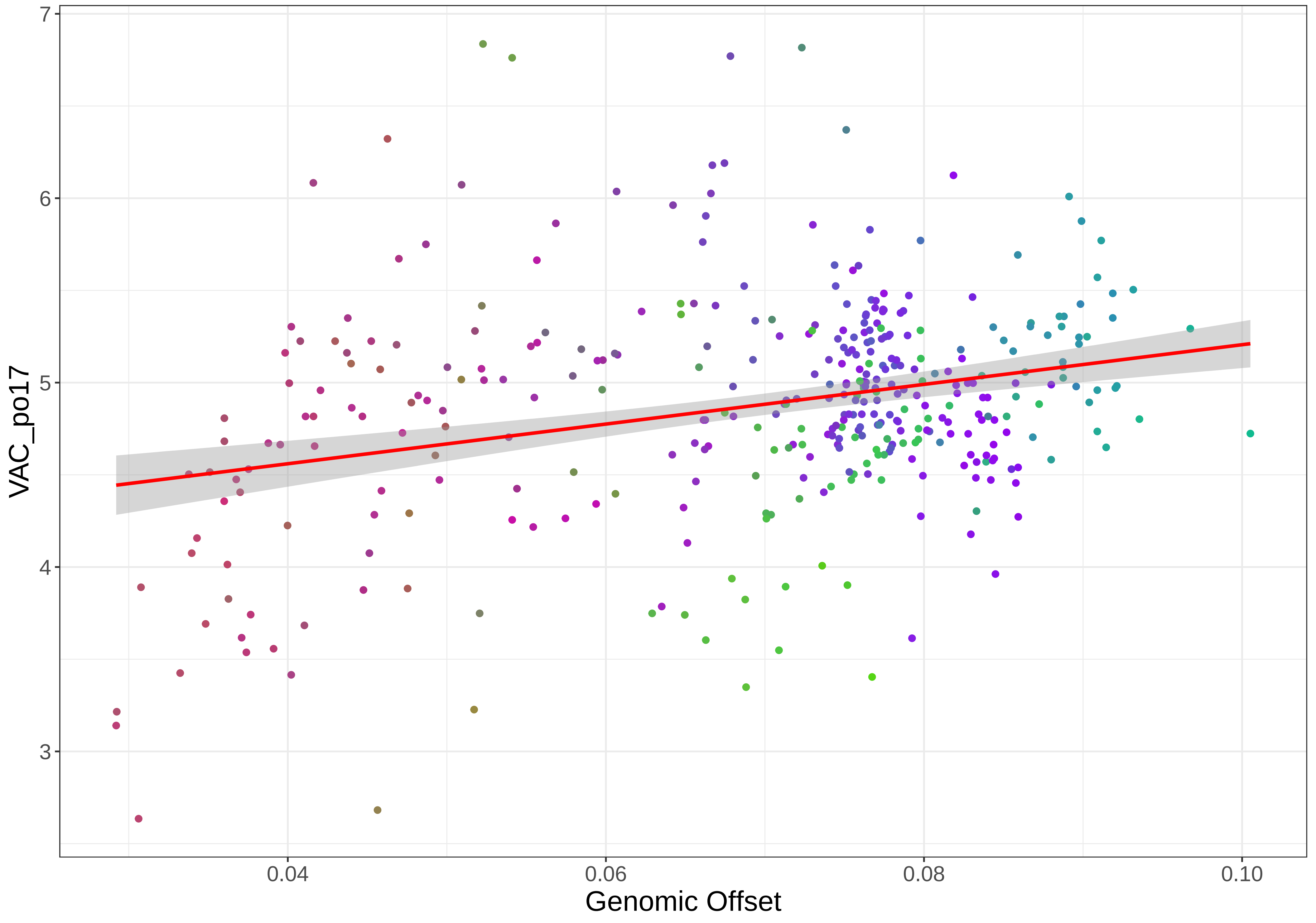

VAC_po17
Genomic Offset
7
6
5
4
3
0.04
0.06
0.08
0.10

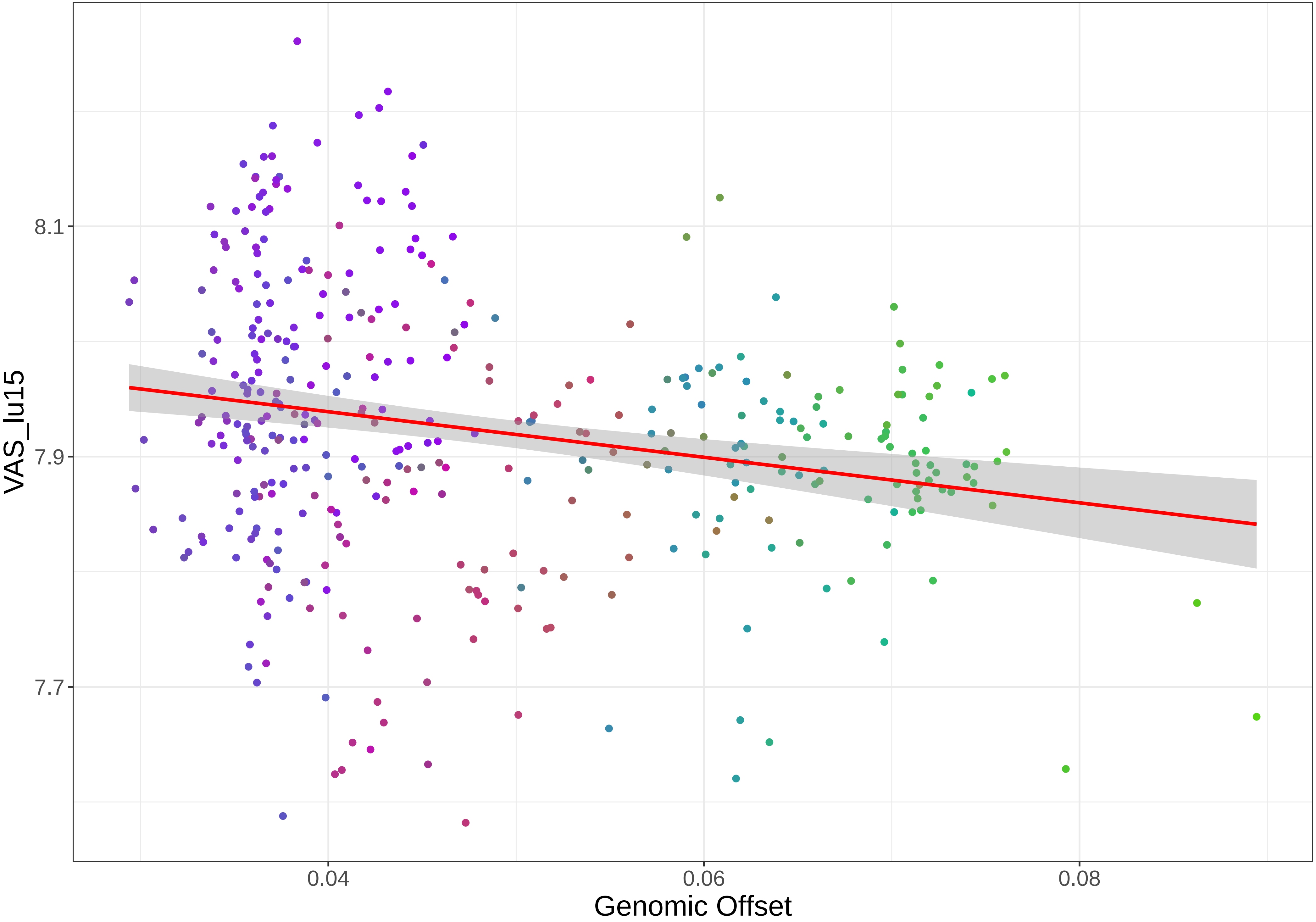
VAS_lu15
8.1
7.9
7.7
0.04
0.06
0.08
Genomic Offset

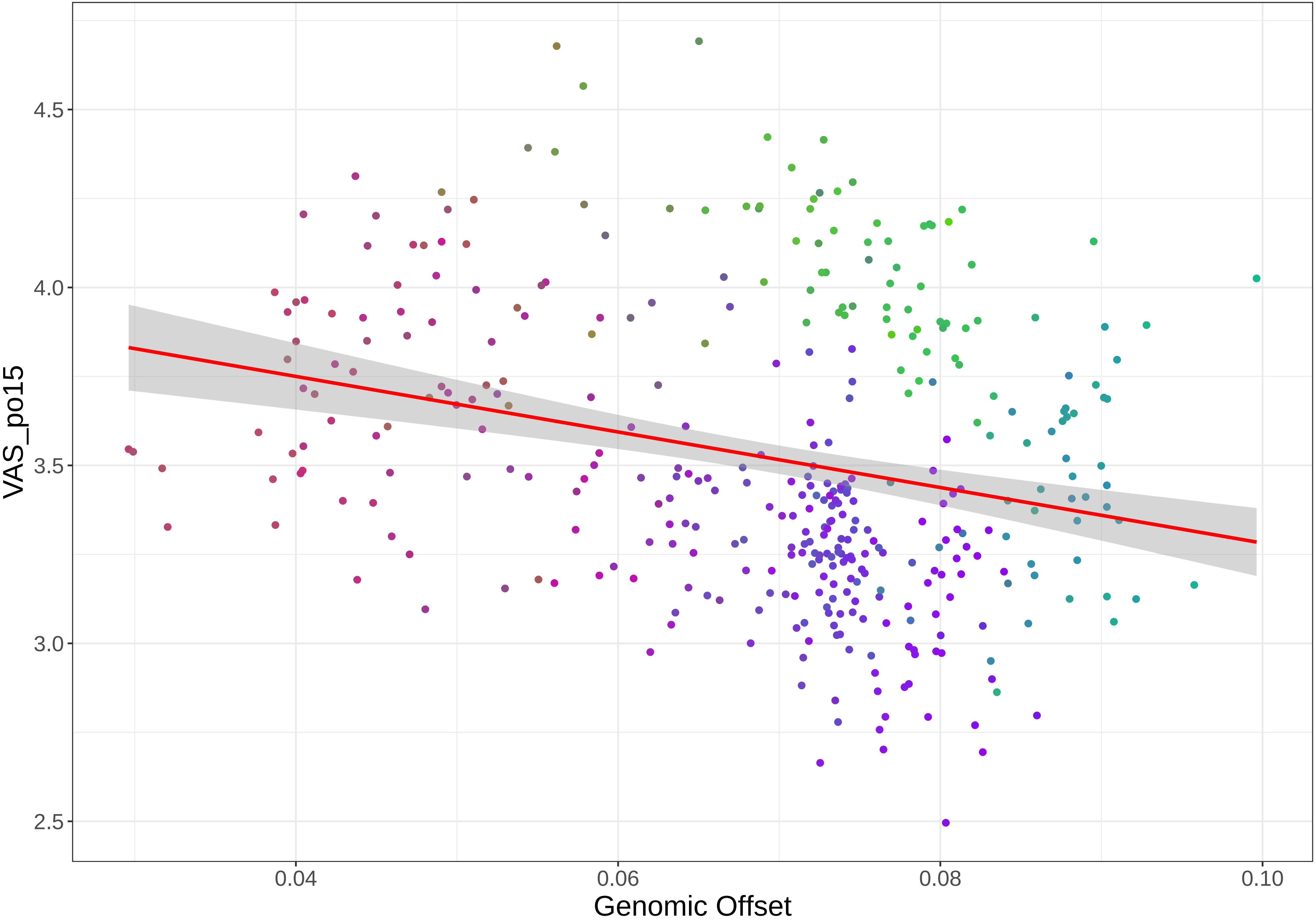

VAS_po15
4.5
4.0
3.5
3.0
2.5
0.04
0.06
0.08
0.10
Genomic Offset

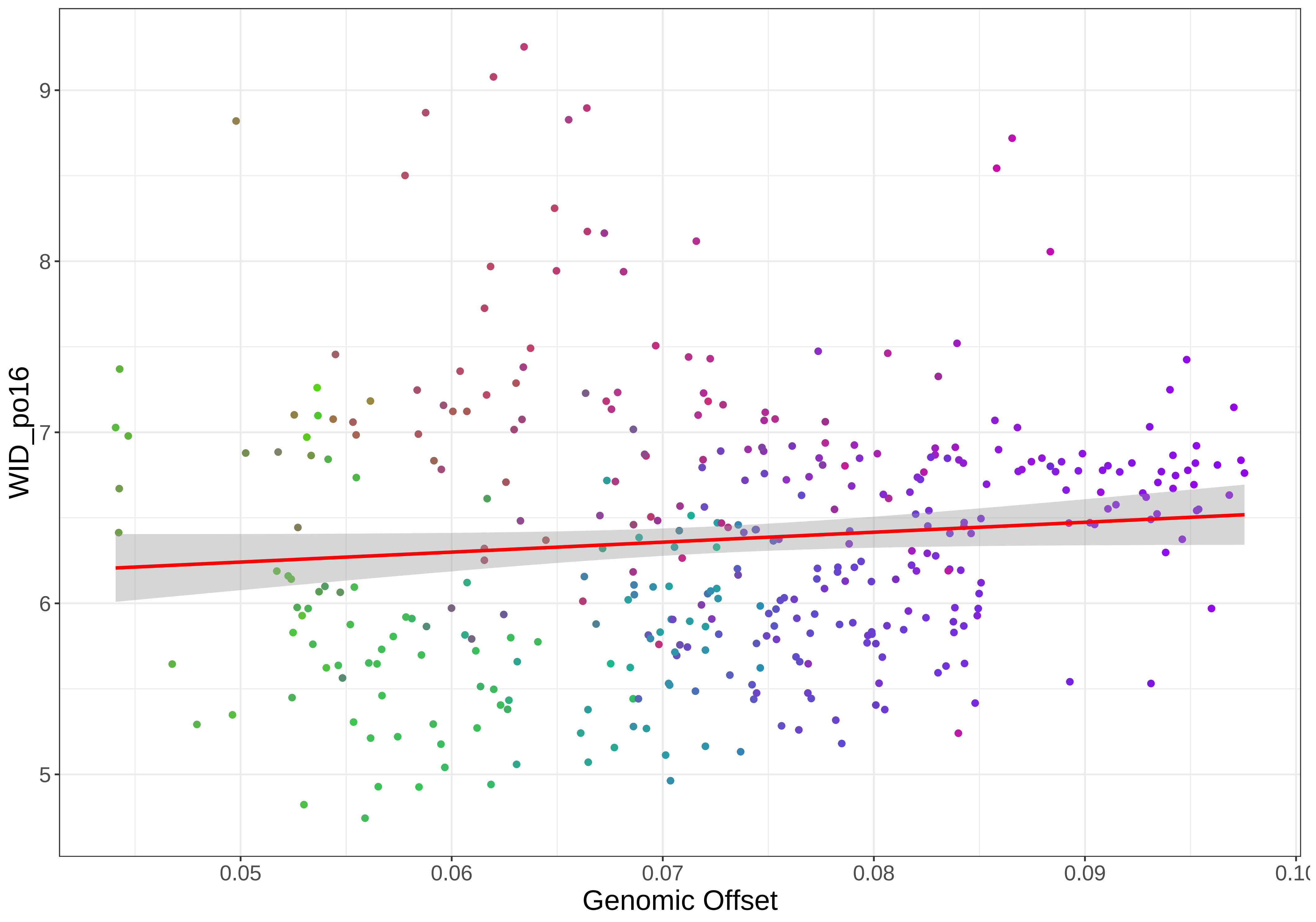

WID_po16
9
8
7
6
5
0.05
0.06
0.07
0.08
0.09
0.10
Genomic Offset

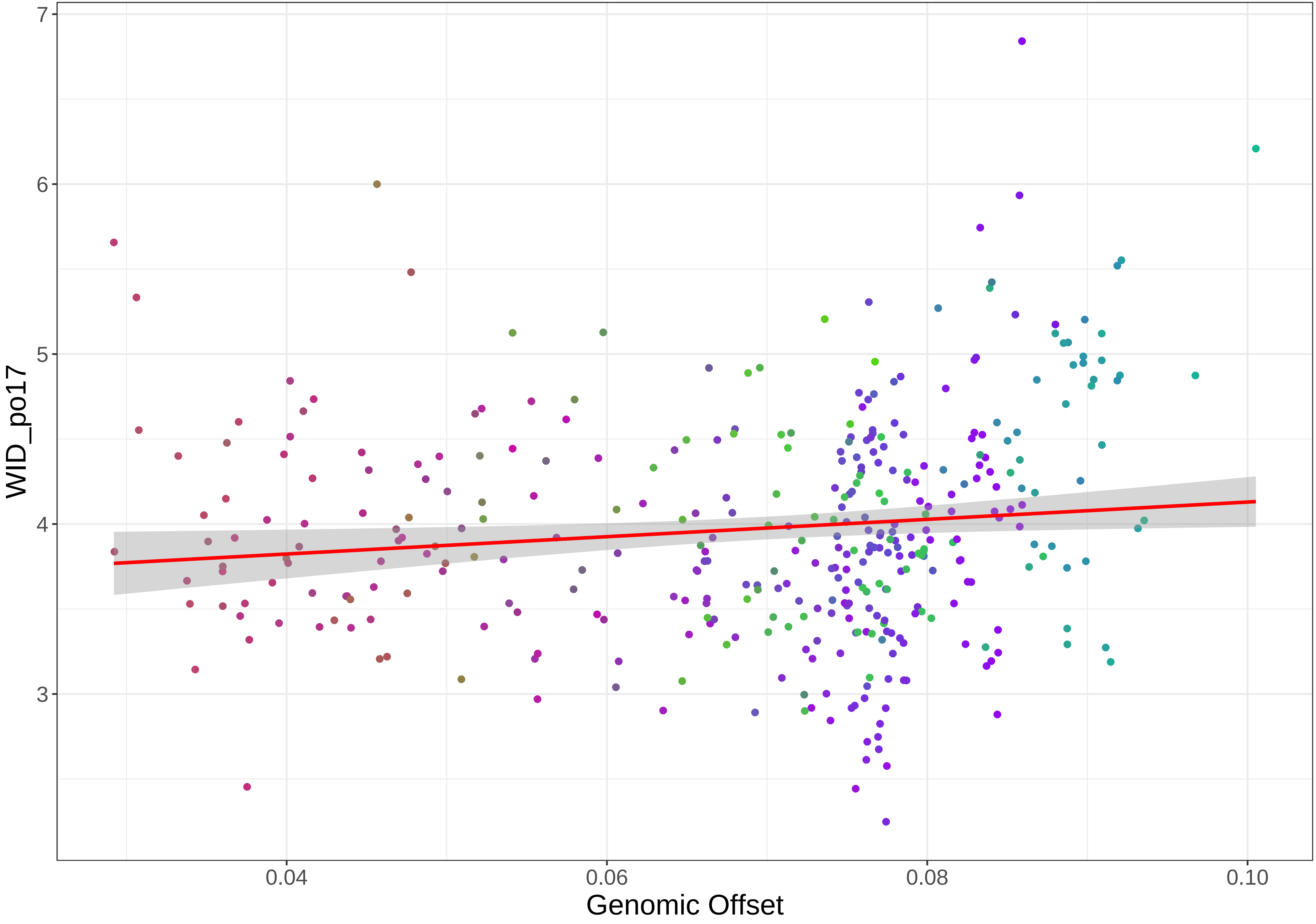

WID_po17
7
6
5
4
3
0.04
0.06
0.08
0.10
Genomic Offset

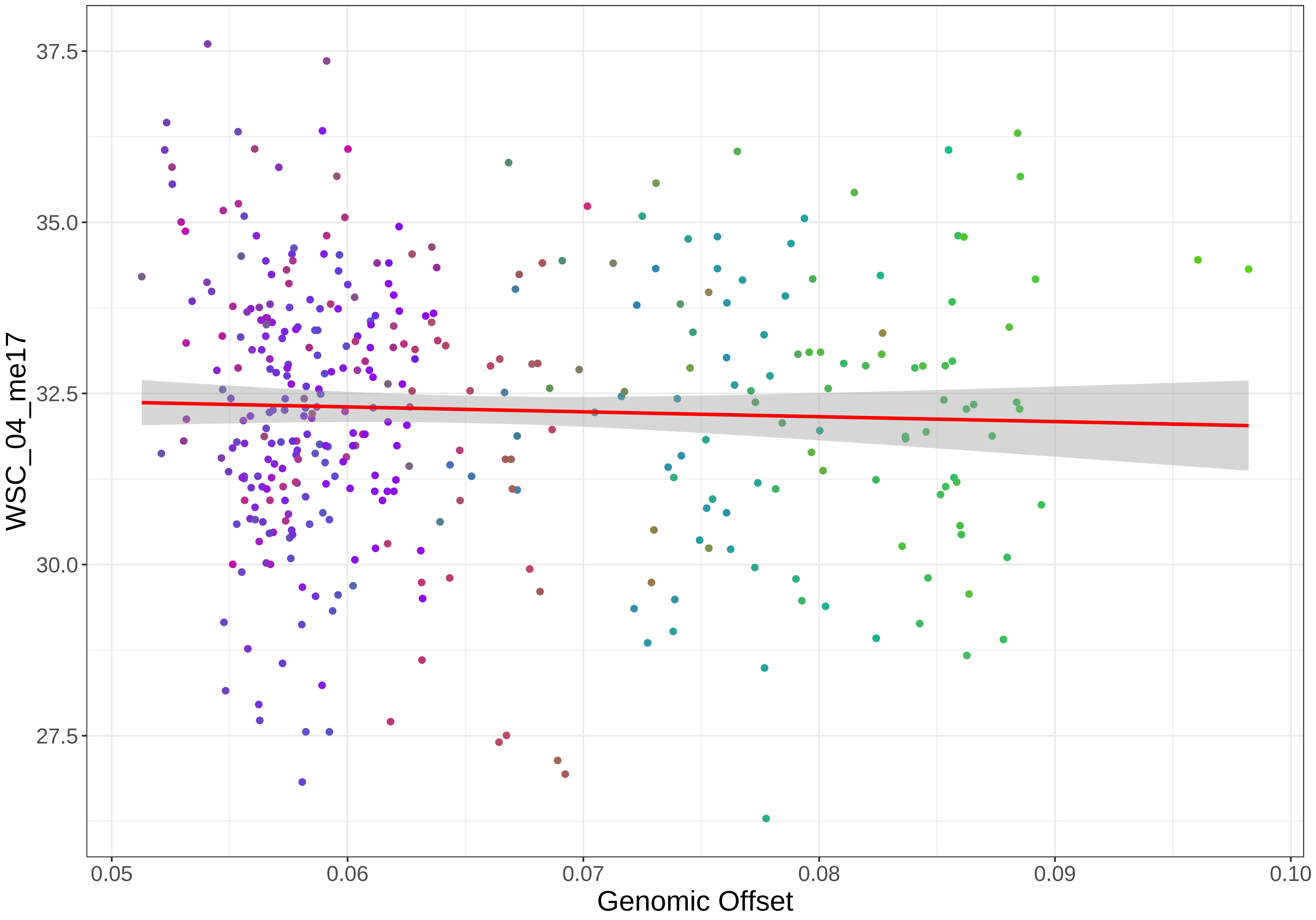

WSC_04_me17
37.5
35.0
32.5
30.0
27.5
0.05
0.06
0.07
0.08
0.09
0.10
Genomic Offset

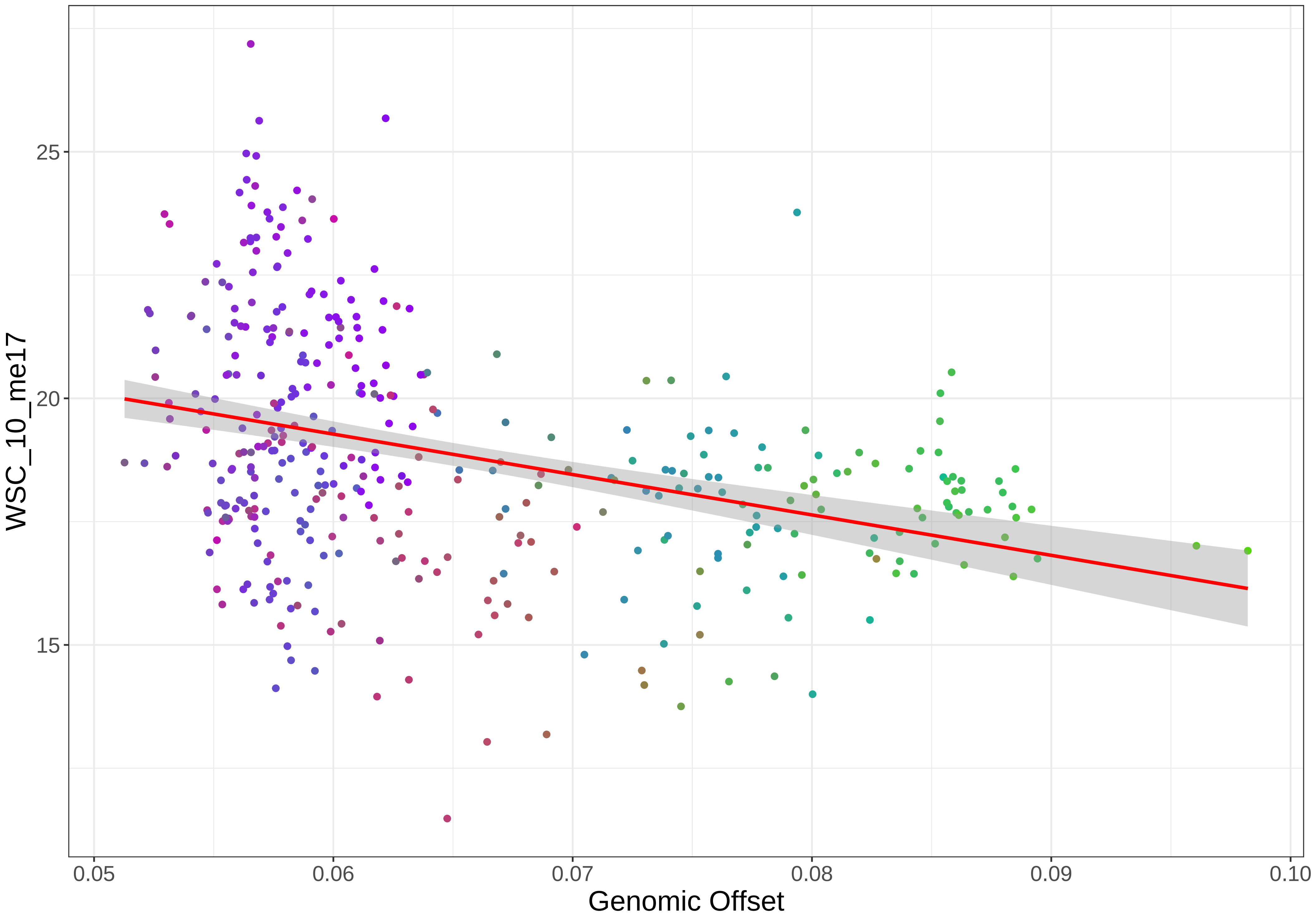

WSC_10_me17
25
20
15
0.05
0.06
0.07
0.08
0.09
0.10
Genomic Offset
